# Genome-wide analysis reveals the extent of EAV-HP integration in domestic chicken

**DOI:** 10.1186/s12864-015-1954-x

**Published:** 2015-10-14

**Authors:** David Wragg, Andrew S. Mason, Le Yu, Richard Kuo, Raman A. Lawal, Takele Taye Desta, Joram M. Mwacharo, Chang-Yeon Cho, Steve Kemp, David W. Burt, Olivier Hanotte

**Affiliations:** Ecology and Evolution, School of Life Sciences, University of Nottingham, University Park, Nottingham, UK; Institut National de la Recherche Agronomique (INRA), UMR 1338 GenPhySE, 31326 Castanet-Tolosan, France; The Roslin Institute and Royal (Dick) School of Veterinary Studies, University of Edinburgh, Midlothian, EH25 9RG Edinburgh, UK; GAIC Co. Ltd. Jing Chen Buiding, Science Park, South Street, Chao Yang District Beijing, People’s Republic Popular of China; International Centre for Agricultural Research in Dry Areas, c/o International Livestock Research Institute, Addis Ababa, Ethiopia; Animal Genetic Resources Station, National Institute of Animal Science, Namwon, Republic of Korea; International Livestock Research Institute, Naivasha Road, P.O. Box 30709, Nairobi, Kenya

**Keywords:** Retrovirus, Symbiosis, Adaptation, Immunogenetics, Genetics, NGS, *Gallus*

## Abstract

**Background:**

EAV-HP is an ancient retrovirus pre-dating *Gallus* speciation, which continues to circulate in modern chicken populations, and led to the emergence of avian leukosis virus subgroup J causing significant economic losses to the poultry industry. We mapped EAV-HP integration sites in Ethiopian village chickens, a Silkie, Taiwan Country chicken, red junglefowl *Gallus gallus* and several inbred experimental lines using whole-genome sequence data.

**Results:**

An average of 75.22 ± 9.52 integration sites per bird were identified, which collectively group into 279 intervals of which 5 % are common to 90 % of the genomes analysed and are suggestive of pre-domestication integration events. More than a third of intervals are specific to individual genomes, supporting active circulation of EAV-HP in modern chickens. Interval density is correlated with chromosome length (*P* < 2.31^−6^), and 27 % of intervals are located within 5 kb of a transcript. Functional annotation clustering of genes reveals enrichment for immune-related functions (*P* < 0.05).

**Conclusions:**

Our results illustrate a non-random distribution of EAV-HP in the genome, emphasising the importance it may have played in the adaptation of the species, and provide a platform from which to extend investigations on the co-evolutionary significance of endogenous retroviral genera with their hosts.

**Electronic supplementary material:**

The online version of this article (doi:10.1186/s12864-015-1954-x) contains supplementary material, which is available to authorized users.

## Background

The EAV retrovirus family likely originated from a primordial integration event prior to the evolutionary speciation of *Gallus sp*. [1], and show sequence similarity with the less ancient *ev* loci [2]. Some EAV elements might be related to the avian leukosis virus (ALV) genus [Dimcheff et al. 2000 in 2], and owing to high *env* sequence identity between EAV and ALV genomes, recombination events involving EAV-HP may have led to the emergence of the ALV subgroup J (ALV-J; [3]). Further support for recombination as the origin of ALV-J is evident in the avian retrotransposon ART-CH, where the R and U5 regions of the ART-CH long terminal repeat (LTR) are 97 % identical to the EAV-HP LTR, while the U3 region is distinct from any other retrovirus [3]. The active spread of EAV-HP in modern chickens is believed to involve a helper virus due to the numerous point mutations, deletions and insertions in EAV-HP sequences inactivating viral gene products [3], and that none of the proviruses found to date have been observed to produce infectious virions [2].

Exogenous retroviruses infect their specific target cells and integrate as a provirus into the cellular genome by transcribing their RNA genome into DNA by reverse transcription [4]. Once integrated into the germ line of a species it becomes endogenised. Parts of the endogenous virus may be eliminated or disrupted by random mutation events, preventing expression of functional viral proteins and the ability to replicate [4]. Consequently, remnants of endogenous retroviruses (ERVs) at varying states of integrity are present in vertebrate genomes and can be observed in re-sequencing data. An analysis of reference genome sequences by Stoye [5] suggests that 4–10 % of vertebrate DNA is composed of retroviral remnants.

A review of retrovirus integration site selection [6] supports the view that the integration of retroviruses at random genomic locations *in vivo* is unlikely to be true for all retroviruses. This review also suggests that integration site preference is often retrovirus but not host-specific. That is to say the same distribution of site preference has been observed in specific retroviridae across a range of mammalian and avian host species. Bolisetty *et al*. [7] identified 31 alpharetroviruses in the chicken genome; this retrovirus genus includes the EAV retroviruses. Desfarges and Ciuffi [6] indicate that alpharetroviruses display a weak preference for integration in transcription units of genes and CpG islands, which are enriched in gene-rich regions. This is consistent with the EAV-HP integration sites identified for the oocyan phenotype in chicken [8, 9], which integrates into the promoter region of *SLCO1B3*. Bolisetty et al. [7] report a near random distribution of ERV integration sites, although this may be an artefact of the preferences of different retrovirus genera. They also indicate that many ERVs are transcribed and translated, and that some are expressed in a tissue-specific manner.

The increasing awareness over the interplay and co-evolution of virus and host has led to the concept of viral symbiosis [10]. Despite being novel to the host genome, the virus is pre-evolved to interact with it at the genetic level. It may contribute genes and/or regulatory sequences to their host, thus conferring a selective advantage. For the virus, successful integration into the host genome offers the prospect of long-term survival and future transmission [11]. The infectious nature of retroviruses, and the capacity of some to become endogenous, offers extraordinary potential to generate heritable diversity in the host genome. Multiple integrations typically occur during endogenisation, and each of these events has the potential to regulate host gene expression given the appropriate integration site. As with any mutation, selection will favour integration sites that enhance the survival of the host, and select against those that impair it. The abundance of retroviruses may therefore play an important role in the adaptation and evolution of the host species [10].

**Fig. 1:**
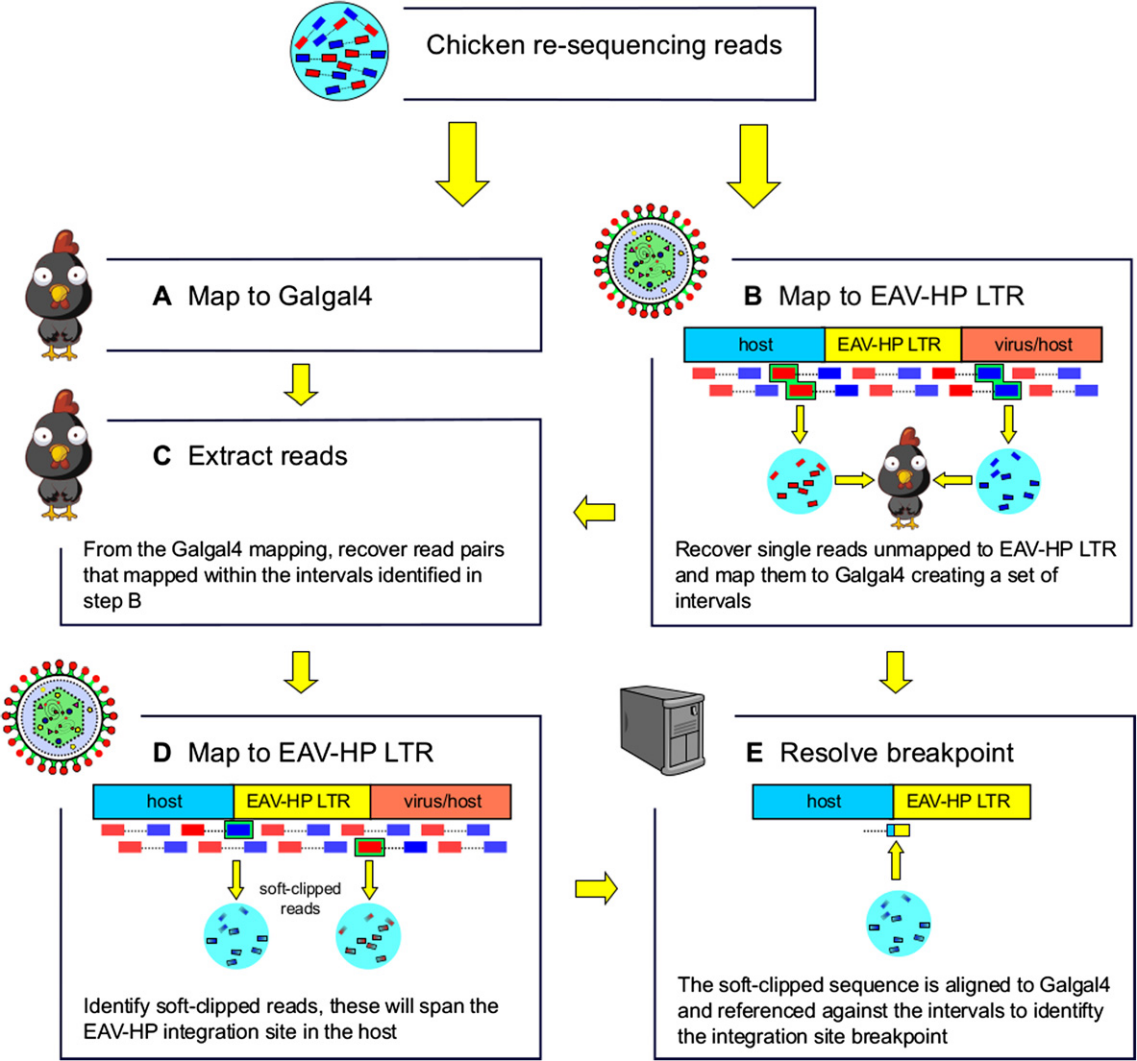
Overview of the retrovirus mapping strategy. Paired-end sequencing reads from re-sequencing of chicken genomes are mapped to both *Galgal4* (**a**) and the LTR of the EAV-HP genome (**b**). Unmapped reads from the EAV-HP LTR alignment, which may map either to the host genome or other elements of the viral sequence, are recovered and mapped to the chicken reference genome to generate a list of observed integration sites (**b**). For each integration sites, paired-end reads are retrieved from the initial mapping to *Galgal4* (**c**) and remapped to the LTR of the EAV-HP genome (**d**), and the soft-clipped sequences of mapped reads recovered (**e**). These sequences are then aligned to *Galgal4* using BLAT, and the results referenced against the interval in question to identify the integration site breakpoint

Here, we assess the prevalence of EAV-HP in chickens with different breeding histories. For this purpose we apply an innovative strategy utilising high-coverage, paired-end next-generation sequencing (NGS) data from re-sequenced chickens, to define EAV-HP integration sites (see Methods and Fig. 1). The chickens analysed included eight inbred lines of White Leghorn currently maintained at the Pirbright Institute (UK), and due to be relocated to the National Avian Research Facility (University of Edinburgh, UK), a Silkie, a Taiwan Country chicken, a red junglefowl, and village chickens from Horro (*n* = 6) and Jarso (*n* = 5) regions of Ethiopia. Specifically, we aim to contribute answers to the following questions: How common are EAV-HP integrations? Do they show a pattern of lineage specificity? What can we infer about the history of such integrations? Do the integrations occur at random across the chicken genome? and if not, is there any evolutionary significance to their distribution?

## Results and discussion

### Prevalence and distribution of EAV-HP integration sites

Alignment of pair-end reads resulted in a genome-wide mean depth of coverage against *Galgal4* of 31.14 ± 1.89 in Ethiopian birds and 15.4 ± 3.63 in Pirbright lines, whilst in the individual birds downloaded from the SRA it was 61.3 in the RJF, 26.33 in the Silkie and 32.34 in the Taiwanese bird (Additional file 1: Supplementary Material S1). Following alignment of the sequence reads to the EAV-HP LTR, and subsequence re-alignment of unmapped reads to *Galgal4*, a total of 1730 integration sites were identified (S2). The mean depth of coverage for sequence reads to the EAV-HP LTR was 12.58 ± 1.08 in Ethiopian chickens, 7.38 ± 2.33 in Pirbright lines, and 16 ± 2.83 in the SRA data, respectively. On average, 31 % of sites contained reads aligning to 100 % of the U3 region (LTR position 1:146) of the LTR sequence, 32 % of sites contained reads aligning to 100 % of the U5 region (LTR position 164:287), whilst the complete R region (LTR position 147:163) was captured in 89 % of sites. Overall, the complete LTR sequence was captured in approximately 20 % of sites.

Integration sites within and across birds and lines with less than 5 kb intervening genomic sequence were classified as a single interval. Similarly, isolated integration sites, those for which no other integration site was identified within 5 kb, were classified as an interval. This reduced the 1730 integration sites into a set of 279 intervals for comparative analyses (S3). The ratio of intervals to integrations was 0.88 in Ethiopian chickens, 0.9 Pirbright lines, and 0.89 in the SRA data, indicating an average fraction of 0.11 integration sites to be clustered. Importantly, one of the Jarso chickens (JB2A04B) was sequenced twice at the comparable genome coverage using independent libraries, and the results of each sequencing run analysed independently. Across both libraries the resulting intervals detected were identical (S3).

**Fig. 2:**
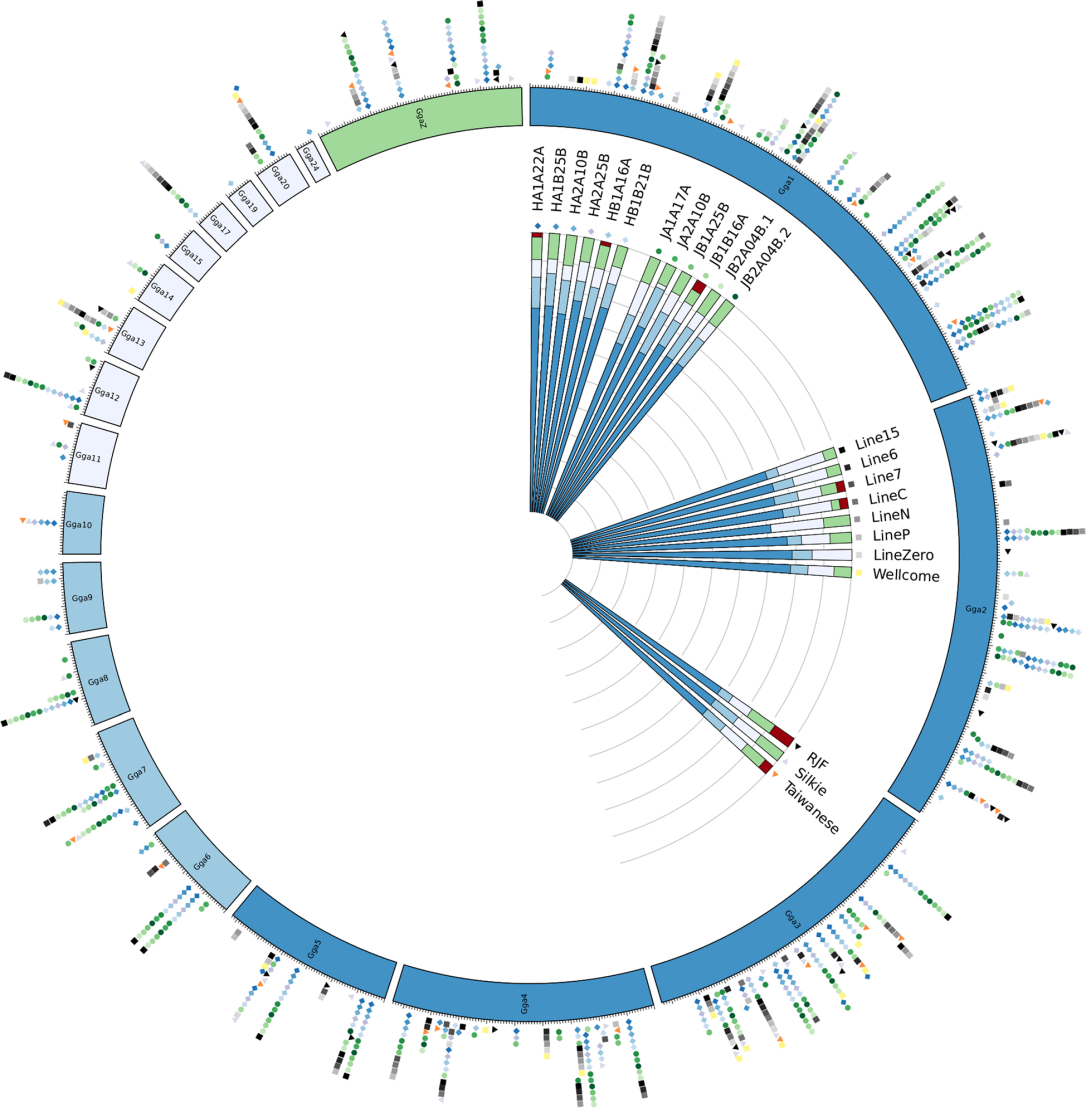
Distribution of observed EAV-HP integration sites. Outer circos plot indicates distribution of observed EAV-HP integration sites. Inner stacked histogram summarizes percentage of integration sites for each line along macro-, intermediate, and micro-chromosomes, in addition to the Z chromosome and unlocalized/unplaced contigs. The latter relates to integration sites mapped to contigs present in the reference assembly (*Galgal4)* that are either associated with a specific chromosome but unlocalized, or have not yet been associated with any chromosome

A summary of the distribution of observed EAV-HP integration site intervals for each re-sequenced genome, on the macro- (1 to 5), intermediate (6 to 10) and micro-chromosomes (11 to 28), in addition to the Z chromosome and several unplaced contigs, is presented in Fig. 2. Of the 279 intervals observed, 72 % mapped to macrochromosomes, 9 % to the intermediate-sized chromosomes, 7 % to microchromosomes and 8 % to the Z chromosome, whilst the remaining 4 % mapped to unplaced contigs (S4). A positive correlation was observed between chromosome length and interval density (*r* = 0.75, *P* < 2.31^−6^; S5), whilst a negative correlation was observed between gene and interval density (*r* = −0.74, *P* < 5.46^−6^; S5). We also observed that 27 % of intervals occur within 5 kb of transcripts (S6). This is less than might be expected by chance given that approximately 41 % of the chicken genome is transcribed. Microchromosomes are both CpG and G + C rich, and have a higher rate of recombination than macrochromosomes [12, 13], which might facilitate excision of retroviral insertions during meiosis. Furthermore, gene density in the chicken has been shown to be inversely correlated with chromosome length [12]. Assuming there to be no target-site preference for EAV-HP, the results suggests that the chicken host retains some integrations within genes or regulatory regions, possibly due to them conferring a selective advantage, whilst others might be more rapidly excised, in agreement with the concept of viral symbiosis [10].

We identify an average of 66.35 ± 7.58 intervals per bird. This is much higher than the 10–15 integrations suggested in previous studies of inbred lines and the red junglefowl based on Southern blot hybridisation with *env* [14] and LTR sequences [3]. However, it is comparable to that observed in a more recent analysis of the *Galgal3* reference genome by Bolisetty et al. [7] using RetroTector [15], in which they identified 31 alpharetroviruses in addition to an alphabeta clade which contained 76 viruses, two thirds of which were highly similar to previously described EAVs.

The precise identification of individual endogenous retroviruses can be problematic due to the extent of sequence diversity observed in mutable proviruses, the level of sequence integrity once a retrovirus becomes endogenised and subject to host recombination, and the level of sequence similarity observed in closely related retroviruses (e.g. EAV-HP and ART-CH [3]). However, EAV-HP has a distinct U3 region [3] which could be used to generate a more conservative estimate of integration sites if one wished. Taking only into consideration the 31 % of integration sites with 100 % sequence coverage of the U3 region, an average of 25.25 ± 2.07, 20.38 ± 4.78 and 27.50 ± 2.12 sites per bird are present in Ethiopian chickens, Pirbright lines and the SRA data, respectively. These values more closely resemble the previously reported Southern blot hybridisation results [14].

In total, 66 % of the EAV-HP LTR alignments to Galgal4 identified by BLAT (S7) were detected as integration sites in the RJF sample. Several filters are applied to the integrations detected during the pipeline including mapping quality (MQ) and read count (RC). An evaluation of these is presented in the Additional file 2: Supplementary Methods. Relaxing the MQ filter applied in the pipeline can result in both sensitivity and precision > 95 %, however the MQ filter is applied to reduce the risk of false positives arising from multiple mapping hits for an integration – for instance if an integration were to occur within an interspersed repetitive element such as CR1. The parameters applied herein (MQ = 20, RC = 0.25*μX*_*i*_) result in 98 % precision and 66 % sensitivity when comparing interval detection in the RJF NGS data to EAV-HP LTR BLAT alignments to *Galgal4*.

Fourteen intervals were present at high frequency (*f* ≥ 0.9) in the chicken genomes analysed, and eight of these were present in all birds (S8a). The presence of these intervals was confirmed in the chicken reference genome (*Galgal4*) following alignment of the EAV-HP LTR sequence using BLAT (S7). Furthermore, visual examination of the results of the BLAT alignment of soft-clipped sequences from each interval allowed the integration site breakpoints to be identified. The integration site breakpoints were found to be identical in each bird, suggesting that they likely occurred prior to domestication. A BLAT of the complete EAV-HP sequence (GenBank: KC632578; S8b) indicates some of these high frequency intervals in *Galgal4* to host large remnants (>1 kb) of EAV-HP sequence, although none of the alignments are complete. Additional sequencing would be require to establish the integrity of the EAV-HP sequences in these intervals for the other birds/lines. Single [16] and multiple [17–19] domestication events have been suggested for domestic chicken, which may have involved several red junglefowl subspecies and introgression from other junglefowl species [20, 21]. Whether or not such retroviral insertions might prove informative in this regard will require the genome analysis of different wild junglefowl species and subspecies.

Three of these candidate pre-domestication integration sites are localised to within 5 kb of a gene: *CNTN5* at chr1:182,832,847-182,834,082 (*f* = 0.91, absent in the N and Wellcome lines), *ATPBD4* at chr5:31,303,866-31,305,068 (*f* = 1), and *C10orf11* at chr6:13,724,843-13,726,022 (*f* = 1). Contactin 5 (*CNTN5*) is a member of the immunoglobulin super family; InnateDB [22] indicates that in mice *ATPBD4* has been demonstrated to interact with *STAT4*, which has been implicated in the innate immune system of mice and humans [23, 24]; whilst *C10orf11* is involved in melanocyte differentiation [25].

Seven intervals were found to be at high frequency (≥0.8) in chickens from either Horro or Jarso, whilst being at low frequency (≤0.2) in the other chickens (S3, S9). One of these (chr3:84,550,518–84,551,452) was present in 100 % of individuals from Jarso, whilst remaining absent in all the other birds and lines examined, including the reference genome. The soft-clipping of reads within this region suggested an integration site breakpoint at either chr3:84,550,973 or chr3:84,550,979, consistent with the size of a target site duplication during integration, which would localise such an integration event into a large intron between exons 20 and 21 of a novel protein coding gene likely to be involved in intracellular signalling (*ENSGALG00000016183*; *fibrilin*-*like precursor*). All Jarso birds indicate 100 % sequence coverage of the EAV-HP LTR at this interval. Another of the intervals (chr8:11,644,653 - 11,645,314), present in all but one of the Jarso chickens and absent in all other birds, suggests an integration site at chr8:11,645,101. This site is located within an intron between exons 8 and 9 of the *DYPD* gene, which encodes a protein involved in uracil and thymidine catabolism. In all birds possessing this interval, sequence coverage of the LTR U3 region is only 5 %, whilst the R and U5 regions ≥ 99 % coverage.

No evidence was found for different integration site breakpoints in the genome between individuals within an interval. In the event that two breakpoints were observed within an interval, they were found either to be 6 bp apart, corresponding to the size of a target-site duplication and so might be attributed to soft-clipped reads from the LTR at either end of the virus (chr2:129,140,825–129,141,707 and chr3:84,550,518–84,551,452; S9), or the integration was present in the reference genome and the breakpoints corresponded closely with the LTR alignment (chr1:182,715,565–183,138,508; S10). This is in contrast to that which has been recently observed at the oocyan locus for Chinese and South American chicken populations [8, 9].

A single interval (chr4:30,632,716–30,633,375) was found to be present in 90 % of Ethiopian birds but absent in the Pirbright lines, red junglefowl, Silkie and Taiwan chickens, in addition to the reference genome (S3). With the exception of the Ethiopian chicken (HA2A10B) in which the interval was not identified, the breakpoint was identical across all chickens at chr4:30,633,163, as was the coverage for the U3 (5 %), R (100 %) and U5 (≥98 %) regions of the LTR. A single gene is located within 5 kb of the interval, *MMAA*, which encodes a protein involved in the translocation of cobalamin into the mitochondrion for the biosynthesis of adenosylcobalamin, an active form of vitamin B_12_. The possible consequence of this integration remains speculative, with the low sequence coverage of the LTR U3 region making it unlikely to contribute a regulatory role. Also, deficiency of vitamin B_12_ would be surprising in domestic birds raised on commercial feed, or in scavenging village chicken whose diet likely includes insects and other arthropods.

**Fig. 3:**
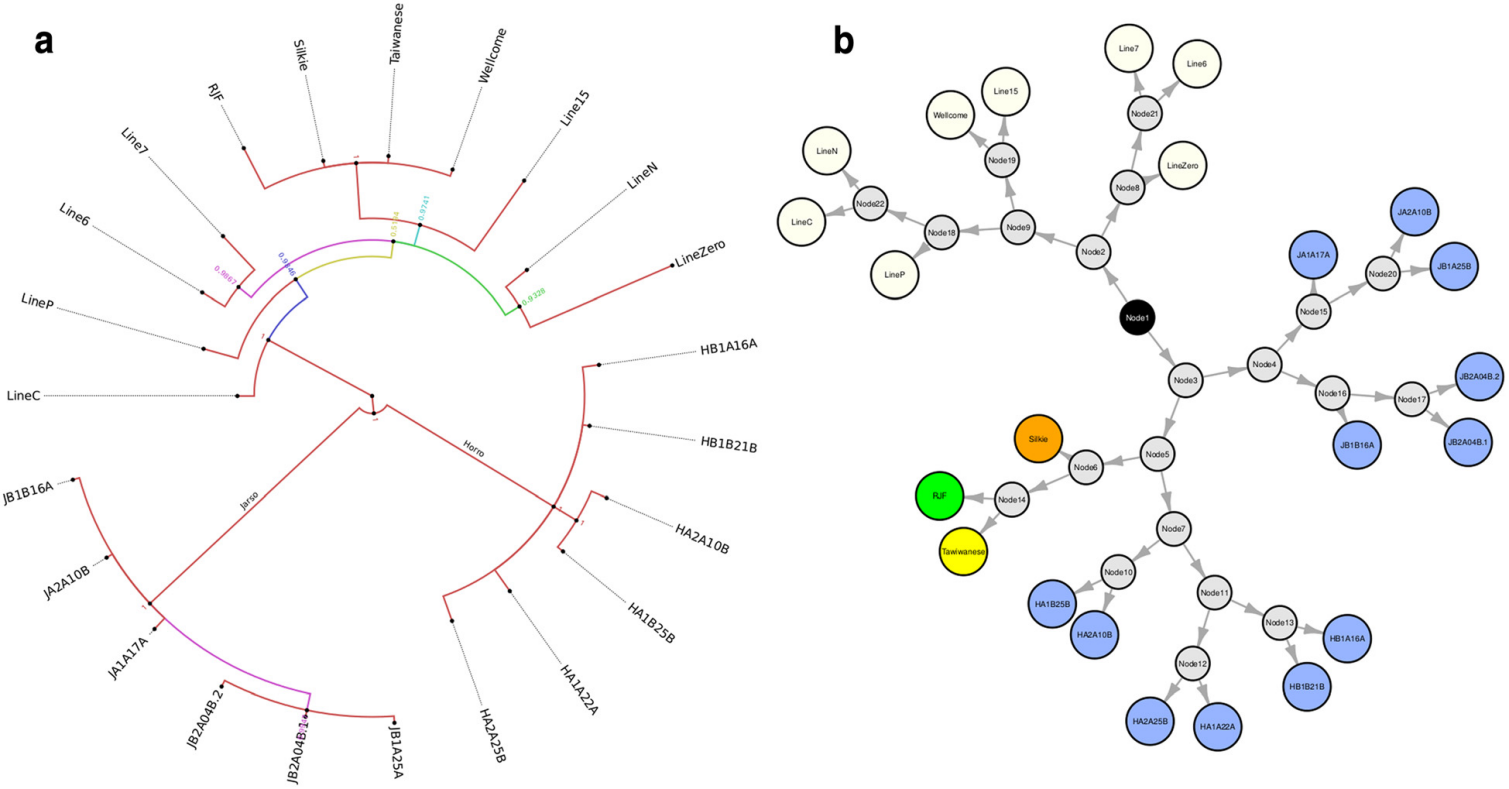
MtDNA phylogeny and network analysis of EAV-HP integration sites. Analysis of mitochondrial sequences from each bird clearly segregates birds from each Ethiopian population and the Pirbright lines, and places the Silkie and Taiwanese chickens as having close maternal ancestry to the red junglefowl (**a**). The network (**b**) from a distance matrix of integration sites present in each bird is largely consistent with this phylogeny. The colours on the phylogeny relate to branch probabilities, whilst those on the network diagram relate to breed and line

Given the heritable nature of ERVs, one would expect to observe population structure when evaluating the ERV integration sites of individuals from within and across different populations. To investigate this further a neighbour-joining tree was constructed using the mitochondrial sequence from each bird (Fig. 3a), the results of which were compared to a network analysis performed on a distance matrix of the ERV intervals present in each chicken (Fig. 3b). Both analyses clearly separate the populations of Ethiopian chickens from the Pirbright lines, and further sub-structuring is observed within the Ethiopian chickens; segregating the sub-populations of Horro and Jarso. The distinction between chickens from Horro and Jarso is not surprising given that these two indigenous populations are separated by more than 800 km and that communities from the areas have witnessed different histories, with Jarso and Horro now of predominantly of Muslim and Christian faith, respectively [26]. As a likely consequence of geographic and human history, little inter-population gene-flow might be expected, as reflected in the EAV-HP integration site network. The red junglefowl, Silkie and Taiwan chickens form an intermediary group more closely related to the Ethiopian chickens than the Pirbright lines.

### Integration sites indicate possible host-virus symbiotic advantage

Functional annotation clustering of the 79 protein-coding genes (S6) located within 5 kb of any of the 279 intervals, identify two clusters enriched for genes encoding proteins with cell adhesion properties and immune-related functions (EASE score > 1.3, *P* < 0.05; S11a). The genes identified include *CNTN1* and *CNTN5,* which encode members of the immunoglobulin (Ig) superfamily, and *TLR2-2* a member of the toll-like receptor (*TLR*) family which play a fundamental role mediating immune responses through pathogen recognition. The *TLR2* genes have been found to be polymorphic among chicken breeds, and different haplotypes may therefore contribute towards differential pathogen responses [27]. Also identified was *ROBO1*, a member of the roundabout gene family best known for their role in the development of the nervous system [28]. Polymorphisms in another member of this family, *ROBO2,* have been linked to differential immune response to Newcastle Disease virus (NDV) in chickens [29]. It should be noted however that the integration site near *ROBO1* was only present in a single chicken (JB2A04B) from Ethiopia.

Average sequence coverage for the U3 region of the LTR was considerably higher for the intervals in proximity to the *CDH7*, *CNTN1*, *CNTN5* and *LRP8* genes (97 %) than for the *FAM172A*, *LARGE*, *ROBO1* and *TLR2-2* genes (9 %). The difference however was less extreme for the R (100 % and 50 % respectively) and U5 (90 % and 79 % respectively) regions (S10). The U3 region of the LTR typically contains binding sites for cellular proteins that promote transcription initiation, and are capable of activating or enhancing the expression of nearby genes in a tissue-specific manner [30]. The conservation of the U3 sequence at some integration sites may be indicative of a possible selective advantage to the host. For instance if it were to confer superinfection resistance, through which cells prevent re-infection by a closely-related virus typically through a virus-encoded protein [31]. An example of such a mechanism is receptor-blocking by an endogenous viral *env* protein, preventing an invading virus binding to the receptor [31]. Sequencing evidence [3] suggests that the emergence of ALV subgroup J was the product of recombination between an unknown exogenous ALV and the *env* gene of EAV-HP, although the expression of EAV-HP *env* peptides was not found to confer resistance to ALV-J [32].

## Conclusions

Through the use of re-sequencing data, we have mapped EAV-HP integration sites in a number of chickens of diverse origin. For this purpose, we have presented and validated a means of detecting EAV-HP integration sites using paired-end NGS data. The sensitivity and precision of the approach was assessed using NGS data derived from the red junglefowl reference bird and comparing the results against the same genome. In addition, we have further demonstrated the reproducibility of our approach through the independent analysis of a single individual sequenced twice using independent libraries. A next step would be to validate a number of the intervals by conventional PCR, and ultimately to assess their impact on gene expression. Although we have demonstrated the method using a single endogenous retrovirus, the same general principles should be applicable for any mobile element present in a host genome for which paired-end sequence data and a reference genome are available.

Our aim was to assess how abundant EAV-HP might be in the chicken genome in the context of recent evidence demonstrating it to regulate the expression of the oocyan phenotypes in some chicken populations. A large number of integrations were found in all chickens examined consistent with historical and ongoing circulation of the virus. A small number of intervals were found to be common in all of the chickens analysed, including the red junglefowl reference genome and we propose that these might represent pre-domestication integration events. The results of this study not only provide a platform from which to extend ERV analysis across different retroviral genera in chicken using re-sequencing data, but also they reiterate the potential for ERV integrations to play a significant role in host evolution, warranting further investigation of host-virus symbioses.

## Methods

### NGS data

Paired-end reads for a Silkie and Taiwan L2 chicken, totalling 30.1 Gb and 38.3 Gb, respectively, were downloaded from NCBI's Sequence Read Archive (SRA) [33], having been made available following a study on the genome-wide patterns of genetic variation in these two birds [34] [SRA:PRJNA202483]. Sequencing for these samples was performed on the Illumina GAIIx platform. Illumina GAIIx sequencing reads for a single red junglefowl, *Gallus gallus*, totalling 67.6 Gb, were also downloaded from NCBI [SRA:SRX043655, SRA:SRX043656]. NGS data from eight inbred lines of White Leghorn from the Pirbright Institute (lines: 0, 6, 7, 15, N, C, P, and Wellcome line; http://www.narf.ac.uk/chickens/lines) was provided by the Roslin Institute, having each been sequenced on the Illumina GAIIx platform from DNA pools of 10 birds per line, averaging 12 Gb per line. Further details about the samples sequenced by the Pirbright Institute, methods of sequencing and alignment of sequence reads to reference genome can be found in Kranis et al. [35].

Village chickens from western (Horro) and eastern (Jarso) Ethiopia were sampled with the consent of farmers [26]. Blood samples were collected from the wing vein, suspended in sodium citrate and stored at −20 °C at the International Livestock Research Institute facility in Addis Ababa, Ethiopia. Ethical approval for the sampling was obtained from the University of Liverpool Committee on Research Ethics (reference RETH000410). Genomic DNA was extracted from whole blood of six Horro and five Jarso chickens following phenol-chloroform extraction [36]. For each sample, a minimum of 15 μg DNA in a total volume of 100 μl was sent to the Beijing Genomics Institute (BGI) for full genome sequencing using the Illumina HiSeq2000 platform, averaging 35 Gb per bird. One of the Jarso chickens (JB2A04B) was sequenced twice using independent libraries, and the results of each sequencing run analysed individually, providing a means of validating the strategy.

The Pirbright lines are characterised as to their major histocompatibility complex (MHC) haplotype and differential resistance to various pathogens [32, 37, 38], providing a resource for investigating bacterial, viral and parasitic infections. These lines are maintained as closed populations. The L2 strain of the Taiwan Country chicken was established in 1983 for meat and egg production and has remained a closed population ever since [39]. By contrast, the characteristic feathering of the Silkie breed was first described hundreds of years ago, and the breed is now widely popular throughout the hobbyist poultry community, although it remained unfashionable in Western cultures until the late 19th century [40]. Each of these chicken lines was artificially selected to varying extents, either for pathogen resistance, commercially desirable traits, or morphology. In contrast, Ethiopian village chicken populations are maintained by smallholder farmers and mating is uncontrolled. Consequently, high levels of phenotypic diversity are typically observed within these populations [26, 41]. Furthermore, village chickens are strongly affected by natural selection and chick mortality rates can be as high as 80-90 % within the first few weeks after hatching due to ease of predation, and cyclical disease and virus outbreaks [42].

### Sequence read mapping

Reads were first mapped to the chicken reference genome (*Galgal4*) using the Burrows-Wheeler Aligner (BWA) [43, 44] (Fig. 1a). Sequence reads were then mapped to the LTR of the EAV-HP genome [GenBank:NC_005947] (Fig. 1b). NGS data provided by the Roslin Institute (University of Edinburgh, UK) was in the format of a SAM file following mapping to the EAV-HP genome, whilst all other samples were mapped at the University of Nottingham (UK). Specifically, in the Ethiopian samples the adapter pollutions in reads were trimmed at source (BGI) along with reads containing more than 50 % low quality bases (quality < 5) bases. Both the Ethiopian dataset, and the reads downloaded from the SRA, were aligned using BWA MEM. From the EAV-HP LTR mapping, unmapped reads were extracted using SAMtools [45], converted to the fastq format using the bam2FastQ tool from the BamUtil repository (http://genome.sph.umich.edu/wiki/BamUtil), and remapped to the chicken reference genome (Fig. 1c).

### Mitochondrial analysis

Reads mapped to the mitochondrial genome were extracted from the BAM alignments for each bird using SAMtools, and consensus sequences aligned using ClustalX v2.1 [46]. A neighbour-joining tree following 1000 bootstrap replicates was constructed using MrBayes v3.22 [47] and plotted with FigTree (v1.4; http://tree.bio.ed.ac.uk/software/figtree/).

### Retroviral integration site analysis

BAM files generated from reads remapped to *Galgal4* were converted to BED format using the bamToBed tool from the BEDTools repository [48]. BED features were filtered to exclude those with a mapping quality below 20. Overlapping features were merged using the mergeBed tool from BEDTools and filtered to exclude those supported by a read count (RC) less than 25 % the mean depth of coverage for the sample (further details in Additional file 2: Supplementary Methods). The resulting list of intervals indicated putative EAV-HP integration sites for each chicken line analysed (Fig. 1c). For each interval, paired reads that were initially mapped to *Galgal4* were retrieved by name using Picard (http://broadinstitute.github.io/picard), and remapped to the LTR of the EAV-HP genome (Fig. 1d), generating an interval-specific set of paired-reads. From these reads, the soft-clipped sequences of reads identified as being clipped by ≥ 20 bases were retrieved (Fig. 1e) using a Perl script. Soft-clipped sequences were then BLAT aligned against *Galgal4* and the results referenced against the interval range using a Perl script. Manual inspection of the filtered BLAT alignments for the soft-clipped sequences allowed the potential integration site breakpoint for EAV-HP to be inferred for the intervals of interest. Sequence read coverage for each *Galgal4* interval and EAV-HP was calculated using GATK's DepthOfCoverage tool. Whilst the fraction of bases aligning to the LTR U3, R and U5 regions was calculated according to base position range of each region within the LTR, with reference to the position of these regions detailed by Sacco et al. [3].

To simplify downstream analyses, integration sites within 5 kb of one-another across different birds and lines were treated as a single interval, the start and end points of which were determined by the minimum and maximum genomic positions across sites within the interval. Likewise, integration sites for which no other integration site was detected within 5 kb were treated as a single interval. Clustering was performed in R [49]. This clustering distance was based on the 4.3 kb length of EAV-HP genome, indicating that any subsequent EAV-HP LTR alignments detected within 5 kb of one-another using BLAT on the chicken reference genome could reasonably be considered to be the result of a single integration. A binomial matrix was generated based on the presence/absence of integration site clusters in each bird using R, from which a distance matrix was computed and used as an input for hierarchical clustering using Ward's minimum variance method [50] as implemented in the hclust function of the stats package in R. A phylogeny was constructed using the as.phylo function of the APE package [51], from which a network was generated using the network package [52]. The distribution of integration sites was plotted using Circos v0.56 [53]. A correlation test for association between chromosome length and interval count was performed using Pearson's product moment correlation coefficient as implemented in the R function cor.test.

The Galgal4.78.gtf file was downloaded from Ensembl and used to reference nearest transcript for each of the intervals. This gene transfer format file includes all of the coding and non-coding transcripts annotated on Ensembl's genome browser for *Galgal4*. The rationale for selecting genes within 5 kb is that this is likely to cover the promoter region, as evidenced by the recent discovery of an EAV-HP integration site ~ 4 kb upstream of *SLCO1B3* in chickens possessing the oocyan phenotype [8, 9]. Functional annotation of genes was assessed using DAVID Bioinformatics Resources v6.7 [54], specifically the functional annotation clustering enrichment threshold for EASE was set to 1.3 with stringency set to HIGH; from the resulting clusters only those with an enrichment score ≥ 1.3 (*P* < 0.05) were retained. Functional annotation analysis was also performed using g:Profiler [55, 56], the results of which are available in the Additional file 1: Supplementary Material (S11b), in addition to REVIGO [57] summaries of GO terms for biological process (S12a, SF1), cellular component (S12b, SF2) and molecular function (S12c, SF3), which for brevity have not been presented here (see Additional file 3). The fraction of chicken genome transcribed (41 %) was calculated from the sum of transcript lengths divided by genome length. This calculation was based on transcripts data downloaded from the Ensembl Genes 74 database for *Galgal4*, from which multiple transcripts per gene were excluded.

### Availability of supporting data

Data supporting the results of this article are available in the NCBI Sequence Read Archive (SRA) repository. This includes whole genome sequence data for the Silkie and Taiwan L2 chickens [SRA:PRJNA202483]; whole genome sequence data for the Red Jungle Fowl [SRA:SRX043655, SRA:SRX043656]; and BAM alignments to the LTR of the EAV-HP genome [GenBank:NC_005947] for the Pirbright lines and Ethiopian village chickens [SRA:SRP062207].

## Additional files

Additional file 1: **Supplementary material.** **S1.** Library details and depth of coverage per bird/line alignment to *Galgal4.* **S2.** Individual integrations per bird/line, sequence coverage at interval in *Galgal4*, and sequence coverage of EAV-HP LTR at the interval site. **S3.** Summary of integration site interval presence/absence per bird/line. **S4.** Summary of distribution of intervals. **S5.** Distribution of intervals relative to chromosome length. **S6.** Intervals and their nearest transcripts (Ensembl, Galgal4.78.gtf). **S7.** BLAT results of EAV-HP LTR to *Galgal4.* **S8a.** Intervals at high frequency (≥0.9) across all birds/lines. **S8b.** BLAT results of EAV-HP (GenBank:KC632578) to *Galgal4.* **S9.** Intervals at high frequency (≥0.8) in Ethiopian chickens from one region, and low frequency (≤0.2) in the chickens from the other region. **S10.** Integration site breakpoints identified near to genes in functional annotation enriched clusters. **S11a.** Functional annotation clustering of protein-coding genes within 5 kb of intervals. **S11b.** gProfiler g:GOSt analysis of genes within 5 kb of intervals. **S12a.** REVIGO Biological Process GO term summary of genes within 5 kb of intervals. **S12b.** REVIGO Cellular Component GO term summary of genes within 5 kb of intervals. **S12c.** REVIGO Molecular Function GO term summary of genes within 5 kb of intervals. (HTML 8512 kb)

Additional file 2: **Supplementary methods.** (PDF 106 kb)

Additional file 3: **Supplementary figures.** **SF1.** REVIGO GO Biological Process uniqueness treemap. **SF2.** REVIGO GO Cellular Component uniqueness treemap. **SF3.** REVIGO GO Molecular Function uniqueness treemap. (PDF 25 kb)

## Abbreviations

ALV: Avian leukosis virus
BWA: Burrows-Wheeler aligner
ERV: Endogenous retrovirus
kb: Kilobase
Gb: Gigabase
LTR: Long terminal repeats
Mb: megabase
MHC: Major histocompatibility complex
NGS: Next-generation sequencing
SRA: Sequence read archive
