# Supplementary material for: Genome-wide analysis reveals the extent of EAV-HP integration in domestic chicken
Source: BMC Genomics. 2015 Oct 14;16:784. doi: 10.1186/s12864-015-1954-x (PMC4607243; doi:10.1186/s12864-015-1954-x)
Supplement: Supplementary file 1 — Supplementary material. S1. Library details and depth of coverage per bird/line alignment to Galgal4. S2. Individual integrations per bird/line, sequence coverage at interval in Galgal4, and sequence coverage of EAV-HP LTR at the interval site. S3. Summary of integration site interval presence/absence per bird/line. S4. Summary of distribution of intervals. S5. Distribution of intervals relative to chromosome length. S6. Intervals and their nearest transcripts (Ensembl, Galgal4.78.gtf). S7. BLAT results of EAV-HP LTR to Galgal4. S8a. Intervals at high frequency (≥0.9) across all birds/lines. S8b. BLAT results of EAV-HP (GenBank:KC632578) to Galgal4. S9. Intervals at high frequency (≥0.8) in Ethiopian chickens from one region, and low frequency (≤0.2) in the chickens from the other region. S10. Integration site breakpoints identified near to genes in functional annotation enriched clusters. S11a. Functional annotation clustering of protein-coding genes within 5 kb of intervals. S11b. gProfiler g:GOSt analysis of genes within 5 kb of intervals. S12a. REVIGO Biological Process GO term summary of genes within 5 kb of intervals. S12b. REVIGO Cellular Component GO term summary of genes within 5 kb of intervals. S12c. REVIGO Molecular Function GO term summary of genes within 5 kb of intervals. (HTML 8512 kb) [file 12864_2015_1954_MOESM1_ESM.html]

---

# Supplementary Material: Genome-wide analysis reveals the extent of EAV-HP integration in domestic chicken

S1  
S2  
S3  
S4  
S5  
S6  
S7  
S8a  
S8b  
S9  
S10  
S11a  
S11b  
S12a  
S12b  
S12c

  


---

  


# Supplementary Table 1: *S1*

| S1 Library details and depth of coverage per bird/line alignment to Galgal4 | | | | | |
|  |  |  |  |  |  |
| **Bird/Line** | **Source** | **Read length** | **Insert size** | **Depth of coverage** | **Notes** |
| HA1A22A | UoN | 90 | 478 | 31.52 | Individual library |
| HA1B25B | UoN | 90 | 476 | 31.05 | Individual library |
| HA2A10B | UoN | 90 | 478 | 32.81 | Individual library |
| HA2A25B | UoN | 90 | 472 | 32.28 | Individual library |
| HB1A16A | UoN | 90 | 479 | 31.93 | Individual library |
| HB1B21B | UoN | 90 | 461 | 32.51 | Individual library |
| JA1A17A | UoN | 90 | 481 | 31.55 | Individual library |
| JA2A10B | UoN | 90 | 479 | 32.04 | Individual library |
| JB2A04B.1 | UoN | 90 | 481 | 31.85 | Individual library |
| JB2A04B.2 | UoN | 90 | 468 | 25.86 | Individual library |
| JB1A25B | UoN | 90 | 480 | 30.96 | Individual library |
| JB1B16A | UoN | 90 | 485 | 29.31 | Individual library |
| Line15 | Roslin Institute | 101 | 322 | 9.64 | Pool of 10 females |
| Line6 | Roslin Institute | 101 | 425 | 15.5 | Pool of 10 females |
| Line7 | Roslin Institute | 101 | 400 | 18.11 | Pool of 10 females |
| LineC | Roslin Institute | 101 | 400 | 16.6 | Pool of 10 females |
| LineN | Roslin Institute | 101 | 337 | 11.43 | Pool of 10 males |
| LineP | Roslin Institute | 101 | 367 | 21.13 | Pool of 10 females |
| LineZero | Roslin Institute | 101 | 426 | 14.55 | Pool of 10 females |
| Wellcome | Roslin Institute | 101 | 354 | 16.26 | Pool of 10 females |
| RJF | SRA | 100 | 204 | 61.3 | SRA Accessions (sample SAMN00216039):  SRR105787 SRR105790 SRR105791 SRR105793 SRR197983 SRR197984  SRR105788 SRR105789 SRR105792 SRR105794 SRR197985 SRR197986 |
| Silkie | SRA | 120 | 371 | 26.33 | SRA Accessions (sample SAMN02142123):  SRR867747 SRR867746 SRR867744 SRR867743 SRR867742 |
| Taiwanese | SRA | 120 | 212 | 32.34 | SRA Accessions (sample SAMN02142124):  SRR867768 SRR867767 SRR867750 SRR867749 SRR867748 |

  


---

  


# Supplementary Table 2: *S2*

| S2. Individual integrations per bird/line, sequence coverage at interval in Galgal4, and sequence coverage of EAV-HP LTR at the interval site | | | | | | | | | | | |
|  |  |  |  |  |  |  |  |  |  |  |  |
|  |  |  |  | **EAV-HP LTR** | | **EAV-HP LTR base coverage** | | | ***Galgal4*** | | |
| **Bird/Line** | **chr** | **start** | **end** | **Depth coverage** | **Base coverage** | **eavU3** | **eavR** | **eavU5** | **Interval** | **Depth of coverage** | **Base coverage** |
| HA1A22A | 1 | 31728920 | 31729569 | 13.76 | 0.52 | 0.05 | 1 | 1 | 1:31728916-31729579 | 29.21 | 1 |
| HA1A22A | 1 | 32312255 | 32313079 | 30.2 | 0.99 | 1 | 1 | 0.98 | 1:32312218-32313185 | 28.36 | 1 |
| HA1A22A | 1 | 34786290 | 34786522 | 4.8 | 0.51 | 0.05 | 1 | 0.99 | 1:34786290-34786522 | 29.05 | 1 |
| HA1A22A | 1 | 41194458 | 41195110 | 15.86 | 0.52 | 0.05 | 1 | 1 | 1:41194458-41195110 | 29.71 | 1 |
| HA1A22A | 1 | 63571457 | 63572053 | 7.11 | 0.51 | 0.05 | 1 | 0.98 | 1:63571454-63572079 | 28.51 | 1 |
| HA1A22A | 1 | 96156975 | 96157347 | 7.69 | 1 | 1 | 1 | 1 | 1:96156897-96157369 | 30.74 | 1 |
| HA1A22A | 1 | 123156471 | 123157302 | 22.91 | 1 | 1 | 1 | 1 | 1:123156456-123157321 | 25.44 | 0.98 |
| HA1A22A | 1 | 140710416 | 140710804 | 13.13 | 1 | 1 | 1 | 0.99 | 1:140710345-140715423 | 43.38 | 1 |
| HA1A22A | 1 | 140714992 | 140715405 | 14.97 | 1 | 1 | 1 | 1 | 1:140710345-140715423 | 43.38 | 1 |
| HA1A22A | 1 | 160463946 | 160464188 | 6.16 | 0.52 | 0.05 | 1 | 1 | 1:160463921-160464245 | 28.33 | 1 |
| HA1A22A | 1 | 163501549 | 163501732 | 4.51 | 0.52 | 0.05 | 1 | 1 | 1:163501535-163501830 | 24.72 | 1 |
| HA1A22A | 1 | 180746925 | 180747121 | 7.3 | 0.51 | 0.05 | 1 | 0.99 | 1:180746896-180747970 | 27.38 | 1 |
| HA1A22A | 1 | 180747699 | 180747956 | 6.18 | 0.51 | 0.05 | 1 | 0.99 | 1:180746896-180747970 | 27.38 | 1 |
| HA1A22A | 1 | 182832868 | 182834031 | 8.36 | 0.9 | 0.97 | 1 | 0.79 | 1:182832847-182834082 | 23.45 | 1 |
| HA1A22A | 1 | 188148835 | 188149712 | 13.7 | 1 | 1 | 1 | 1 | 1:188148835-188149736 | 34.88 | 1 |
| HA1A22A | 2 | 1183407 | 1184008 | 8.04 | 0.52 | 0.05 | 1 | 1 | 2:1183407-1184047 | 26.6 | 0.99 |
| HA1A22A | 2 | 50798303 | 50798931 | 14.22 | 0.51 | 0.05 | 1 | 0.98 | 2:50798296-50798951 | 29.87 | 1 |
| HA1A22A | 2 | 53111767 | 53112004 | 8.81 | 0.93 | 0.88 | 1 | 0.98 | 2:53111756-53112017 | 27.96 | 1 |
| HA1A22A | 2 | 77178330 | 77179069 | 13.88 | 1 | 1 | 1 | 0.99 | 2:77178330-77179069 | 26.26 | 1 |
| HA1A22A | 2 | 81965010 | 81965280 | 9.39 | 0.51 | 0.05 | 1 | 0.99 | 2:81965001-81966072 | 31.86 | 1 |
| HA1A22A | 2 | 81965793 | 81966043 | 16.93 | 0.51 | 0.05 | 1 | 0.99 | 2:81965001-81966072 | 31.86 | 1 |
| HA1A22A | 2 | 82574339 | 82575505 | 5.6 | 0.47 | 0.93 | 0 | 0 | 2:82574297-82575518 | 31.31 | 1 |
| HA1A22A | 2 | 92857067 | 92858202 | NA | NA | NA | NA | NA | 2:92856996-92858219 | 28.34 | 1 |
| HA1A22A | 2 | 94594289 | 94594911 | 7.41 | 0.51 | 0.05 | 1 | 0.99 | 2:94594272-94594911 | 25.18 | 1 |
| HA1A22A | 2 | 95480963 | 95481818 | 13.13 | 1 | 1 | 1 | 0.99 | 2:95480957-95481836 | 35.81 | 1 |
| HA1A22A | 2 | 142113880 | 142114473 | 13.01 | 0.51 | 0.05 | 1 | 0.98 | 2:142113866-142114543 | 26.53 | 1 |
| HA1A22A | 3 | 19821803 | 19822995 | 4.59 | 0.68 | 0.83 | 0 | 0.6 | 3:19821802-19823007 | 24.05 | 1 |
| HA1A22A | 3 | 34783135 | 34783373 | 5.9 | 0.51 | 0.05 | 1 | 0.98 | 3:34782953-34783379 | 36.16 | 1 |
| HA1A22A | 3 | 49572078 | 49572929 | 17.95 | 1 | 1 | 1 | 1 | 3:49572068-49572931 | 36.88 | 1 |
| HA1A22A | 3 | 50010952 | 50011588 | 12.03 | 0.52 | 0.05 | 1 | 1 | 3:50010952-50011588 | 28.08 | 1 |
| HA1A22A | 3 | 54250151 | 54250391 | 18.75 | 0.51 | 0.05 | 1 | 0.98 | 3:54250109-54254158 | 40.33 | 1 |
| HA1A22A | 3 | 54253875 | 54254158 | 10.69 | 0.52 | 0.05 | 1 | 1 | 3:54250109-54254158 | 40.33 | 1 |
| HA1A22A | 3 | 60621861 | 60622499 | 26.31 | 0.51 | 0.05 | 1 | 0.99 | 3:60621854-60622499 | 28.46 | 1 |
| HA1A22A | 3 | 70604586 | 70605356 | 26.23 | 0.99 | 1 | 1 | 0.98 | 3:70604555-70605387 | 22.24 | 1 |
| HA1A22A | 3 | 73561892 | 73563074 | 2.01 | 0.55 | 0.34 | 1 | 0.74 | 3:73561879-73563101 | 29.52 | 1 |
| HA1A22A | 3 | 83776919 | 83777157 | 12.69 | 0.47 | 0.05 | 1 | 0.89 | 3:83776894-83777945 | 35.14 | 1 |
| HA1A22A | 3 | 83777704 | 83777939 | 17.14 | 0.46 | 0.05 | 1 | 0.87 | 3:83776894-83777945 | 35.14 | 1 |
| HA1A22A | 3 | 86869588 | 86870248 | 32.08 | 0.52 | 0.05 | 1 | 0.99 | 3:86869588-86870258 | 31.34 | 1 |
| HA1A22A | 3 | 96957550 | 96957815 | 15.07 | 0.51 | 0.05 | 1 | 0.98 | 3:96957515-96961612 | 35.27 | 1 |
| HA1A22A | 3 | 96961308 | 96961589 | 15.89 | 0.52 | 0.05 | 1 | 1 | 3:96957515-96961612 | 35.27 | 1 |
| HA1A22A | 3 | 102777273 | 102778125 | 34.71 | 1 | 1 | 1 | 1 | 3:102777226-102778143 | 30.65 | 1 |
| HA1A22A | 4 | 11124007 | 11124375 | 14.27 | 0.99 | 1 | 1 | 0.98 | 4:11123901-11124467 | 31.7 | 1 |
| HA1A22A | 4 | 26530940 | 26531129 | 6.57 | 0.51 | 0.05 | 1 | 0.98 | 4:26530893-26531990 | 29.93 | 1 |
| HA1A22A | 4 | 26531685 | 26531917 | 10.28 | 0.51 | 0.05 | 1 | 0.98 | 4:26530893-26531990 | 29.93 | 1 |
| HA1A22A | 4 | 30632740 | 30633364 | 10.24 | 0.51 | 0.05 | 1 | 0.99 | 4:30632716-30633375 | 26.87 | 1 |
| HA1A22A | 4 | 48096959 | 48097615 | 21.23 | 0.52 | 0.05 | 1 | 1 | 4:48096947-48097615 | 28.23 | 1 |
| HA1A22A | 4 | 70551893 | 70552660 | 14.24 | 0.99 | 1 | 1 | 0.98 | 4:70551829-70552669 | 22.64 | 1 |
| HA1A22A | 4 | 86220904 | 86221807 | 13.34 | 0.99 | 0.99 | 1 | 1 | 4:86220862-86221807 | 28.75 | 1 |
| HA1A22A | 5 | 2705056 | 2706243 | 8.32 | 0.86 | 0.99 | 1 | 0.69 | 5:2705052-2706284 | 30.6 | 1 |
| HA1A22A | 5 | 6164288 | 6165413 | 2.66 | 0.16 | 0 | 0 | 0.38 | 5:6164269-6165523 | 19.21 | 1 |
| HA1A22A | 5 | 31303902 | 31305051 | 1 | 0.26 | 0.13 | 1 | 0.31 | 5:31303866-31305068 | 29.46 | 1 |
| HA1A22A | 5 | 31898704 | 31899868 | 2.34 | 0.53 | 0.55 | 1 | 0.44 | 5:31898626-31899883 | 29 | 1 |
| HA1A22A | 5 | 39163496 | 39164643 | 6.1 | 0.77 | 0.99 | 1 | 0.49 | 5:39163484-39164645 | 26.6 | 1 |
| HA1A22A | 5 | 40464006 | 40464915 | 11.9 | 0.99 | 0.99 | 1 | 0.99 | 5:40463978-40464915 | 33.82 | 1 |
| HA1A22A | 5 | 41299361 | 41300140 | 17.15 | 1 | 1 | 1 | 1 | 5:41299361-41300140 | 31.27 | 1 |
| HA1A22A | 6 | 9811217 | 9811599 | 12.22 | 0.99 | 0.99 | 1 | 1 | 6:9811191-9816444 | 29.13 | 0.99 |
| HA1A22A | 6 | 9816033 | 9816393 | 13.23 | 0.99 | 0.99 | 1 | 0.98 | 6:9811191-9816444 | 29.13 | 0.99 |
| HA1A22A | 6 | 13724884 | 13726008 | 2.98 | 0.59 | 0.55 | 0.41 | 0.66 | 6:13724843-13726022 | 31.64 | 1 |
| HA1A22A | 7 | 9022771 | 9023423 | 8.45 | 0.52 | 0.05 | 1 | 1 | 7:9022731-9023457 | 30.13 | 1 |
| HA1A22A | 7 | 17708094 | 17708698 | 21.11 | 0.5 | 0.03 | 1 | 0.99 | 7:17708068-17708753 | 29.75 | 1 |
| HA1A22A | 8 | 9208418 | 9209227 | 6.24 | 1 | 1 | 1 | 1 | 8:9208394-9209273 | 17.61 | 1 |
| HA1A22A | 9 | 5602615 | 5603250 | 11.77 | 0.51 | 0.05 | 1 | 0.98 | 9:5602574-5603257 | 30.91 | 1 |
| HA1A22A | 10 | 9703202 | 9703820 | 27.29 | 0.51 | 0.05 | 1 | 0.98 | 10:9703183-9703845 | 31.12 | 1 |
| HA1A22A | 12 | 3240724 | 3240870 | 38.43 | 0.55 | 1 | 0.76 | 0 | 12:3240697-3240898 | 203.09 | 1 |
| HA1A22A | 20 | 7661925 | 7662753 | 31.76 | 1 | 1 | 1 | 1 | 20:7661897-7662786 | 30.32 | 1 |
| HA1A22A | 20 | 9408955 | 9409567 | 14.12 | 0.51 | 0.05 | 1 | 0.98 | 20:9408955-9409567 | 24.8 | 1 |
| HA1A22A | AADN03009901.1 | 934 | 1158 | 7.64 | 0.51 | 0.05 | 1 | 0.99 | AADN03009901.1:851-1161 | 32.98 | 1 |
| HA1A22A | JH375157.1 | 367 | 515 | 10.32 | 0.52 | 0.05 | 1 | 1 | JH375157.1:356-536 | 20.28 | 1 |
| HA1A22A | JH375968.1 | 8535 | 8818 | 5.54 | 0.94 | 0.88 | 1 | 1 | JH375968.1:8535-8818 | 157.5 | 1 |
| HA1A22A | JH376323.1 | 928 | 1072 | 4.53 | 0.55 | 1 | 0.76 | 0 | JH376323.1:928-1072 | 114.68 | 1 |
| HA1A22A | Z | 18808046 | 18808308 | 12.23 | 0.62 | 0.47 | 1 | 0.73 | Z:18807991-18809115 | 32.44 | 1 |
| HA1A22A | Z | 18808822 | 18809077 | 14.79 | 0.63 | 0.49 | 1 | 0.73 | Z:18807991-18809115 | 32.44 | 1 |
| HA1A22A | Z | 32081175 | 32081586 | 11.8 | 1 | 1 | 1 | 1 | Z:32081154-32086293 | 55.12 | 1 |
| HA1A22A | Z | 32085876 | 32086269 | 15.56 | 1 | 1 | 1 | 0.99 | Z:32081154-32086293 | 55.12 | 1 |
| HA1A22A | Z | 50872687 | 50873298 | 32.7 | 0.52 | 0.05 | 1 | 1 | Z:50872653-50873337 | 33.99 | 1 |
| HA1A22A | Z | 61304843 | 61305998 | 7.84 | 0.7 | 0.43 | 1 | 0.97 | Z:61304819-61306021 | 34.03 | 1 |
| HA1A22A | Z | 79945553 | 79946417 | 29.28 | 1 | 1 | 1 | 1 | Z:79945501-79946432 | 27.28 | 1 |
| HA1B25B | 1 | 8127812 | 8128538 | 14.23 | 1 | 1 | 1 | 1 | 1:8127806-8128614 | 23.47 | 1 |
| HA1B25B | 1 | 32312274 | 32313047 | 11.52 | 0.99 | 1 | 1 | 0.98 | 1:32312218-32313185 | 26.21 | 1 |
| HA1B25B | 1 | 42541935 | 42542576 | 13.52 | 0.51 | 0.05 | 1 | 0.99 | 1:42541895-42542614 | 32.01 | 1 |
| HA1B25B | 1 | 99050593 | 99051176 | 13.26 | 0.52 | 0.05 | 1 | 1 | 1:99050515-99051202 | 30.13 | 1 |
| HA1B25B | 1 | 99136316 | 99136927 | 15.5 | 0.46 | 0.05 | 1 | 0.87 | 1:99136310-99136953 | 25.25 | 1 |
| HA1B25B | 1 | 101333516 | 101334373 | 17.48 | 1 | 1 | 1 | 1 | 1:101333491-101334404 | 29 | 1 |
| HA1B25B | 1 | 113945493 | 113946354 | 7.34 | 0.36 | 0 | 0 | 0.83 | 1:113945460-113946362 | 31.04 | 1 |
| HA1B25B | 1 | 117166643 | 117167523 | 18.42 | 1 | 1 | 1 | 1 | 1:117166631-117167523 | 31.17 | 1 |
| HA1B25B | 1 | 140710374 | 140710793 | 16.57 | 1 | 1 | 1 | 0.99 | 1:140710345-140715423 | 42.42 | 1 |
| HA1B25B | 1 | 140715019 | 140715412 | 17.24 | 1 | 1 | 1 | 1 | 1:140710345-140715423 | 42.42 | 1 |
| HA1B25B | 1 | 146731919 | 146732820 | 29.45 | 1 | 1 | 1 | 1 | 1:146731910-146732833 | 28.61 | 1 |
| HA1B25B | 1 | 146761564 | 146762190 | 10.06 | 0.52 | 0.05 | 1 | 1 | 1:146761527-146762190 | 29.75 | 1 |
| HA1B25B | 1 | 149162770 | 149163187 | 7.01 | 1 | 1 | 1 | 0.99 | 1:149162760-149166227 | 47.75 | 1 |
| HA1B25B | 1 | 149165143 | 149166227 | 5.22 | 0.16 | 0.32 | 0 | 0 | 1:149162760-149166227 | 47.75 | 1 |
| HA1B25B | 1 | 149501163 | 149502040 | 13.64 | 1 | 1 | 1 | 1 | 1:149501163-149502040 | 31.85 | 1 |
| HA1B25B | 1 | 149935105 | 149935762 | 11.78 | 0.52 | 0.05 | 1 | 1 | 1:149935105-149935762 | 28.68 | 1 |
| HA1B25B | 1 | 151384845 | 151385716 | 15.64 | 1 | 1 | 1 | 1 | 1:151384845-151385716 | 31.1 | 1 |
| HA1B25B | 1 | 159625843 | 159626482 | 10.85 | 0.52 | 0.05 | 1 | 1 | 1:159625843-159626482 | 29.05 | 1 |
| HA1B25B | 1 | 160455253 | 160455504 | 5.62 | 0.51 | 0.05 | 1 | 0.98 | 1:160455218-160455708 | 27.08 | 1 |
| HA1B25B | 1 | 160463989 | 160464210 | 3.72 | 0.52 | 0.05 | 1 | 1 | 1:160463921-160464245 | 26.36 | 1 |
| HA1B25B | 1 | 163501560 | 163501742 | 7.53 | 0.52 | 0.05 | 1 | 1 | 1:163501535-163501830 | 20.79 | 1 |
| HA1B25B | 1 | 163906799 | 163907066 | 5.95 | 0.52 | 0.05 | 1 | 1 | 1:163906737-163910850 | 27.66 | 1 |
| HA1B25B | 1 | 163910553 | 163910802 | 6.3 | 0.51 | 0.05 | 1 | 0.98 | 1:163906737-163910850 | 27.66 | 1 |
| HA1B25B | 1 | 180746919 | 180747134 | 21.84 | 0.51 | 0.05 | 1 | 0.99 | 1:180746896-180747970 | 38.09 | 1 |
| HA1B25B | 1 | 180747700 | 180747961 | 18.56 | 0.52 | 0.05 | 1 | 1 | 1:180746896-180747970 | 38.09 | 1 |
| HA1B25B | 1 | 182832894 | 182834053 | 3.04 | 0.93 | 0.88 | 1 | 0.98 | 1:182832847-182834082 | 23.81 | 1 |
| HA1B25B | 1 | 186846427 | 186847004 | 16.64 | 0.52 | 0.05 | 1 | 1 | 1:186846427-186847073 | 30.91 | 1 |
| HA1B25B | 1 | 188148836 | 188149736 | 30.39 | 1 | 1 | 1 | 1 | 1:188148835-188149736 | 28.31 | 1 |
| HA1B25B | 2 | 3491413 | 3492279 | 15.93 | 1 | 1 | 1 | 1 | 2:3491394-3492279 | 32.09 | 1 |
| HA1B25B | 2 | 10512601 | 10512826 | 12.03 | 0.52 | 0.05 | 1 | 0.99 | 2:10512425-10512887 | 27.64 | 1 |
| HA1B25B | 2 | 20971543 | 20972430 | 10.56 | 0.62 | 0.27 | 1 | 0.98 | 2:20971534-20972437 | 30.84 | 1 |
| HA1B25B | 2 | 53111795 | 53111995 | 4.64 | 0.91 | 0.83 | 1 | 0.98 | 2:53111756-53112017 | 27.24 | 1 |
| HA1B25B | 2 | 80333585 | 80334238 | 12.99 | 0.51 | 0.05 | 1 | 0.98 | 2:80333585-80334273 | 35.1 | 0.99 |
| HA1B25B | 2 | 81965031 | 81966058 | 7.72 | 0.8 | 0.84 | 0 | 0.87 | 2:81965001-81966072 | 28.73 | 1 |
| HA1B25B | 2 | 82574333 | 82575487 | 5.63 | 0.47 | 0.9 | 0.18 | 0 | 2:82574297-82575518 | 27.11 | 1 |
| HA1B25B | 2 | 92857033 | 92858179 | 1 | 0.51 | 0.26 | 1 | 0.74 | 2:92856996-92858219 | 29.91 | 1 |
| HA1B25B | 2 | 129140825 | 129141694 | 15.16 | 1 | 0.99 | 1 | 1 | 2:129140825-129141707 | 29.06 | 1 |
| HA1B25B | 2 | 131563813 | 131564031 | 7.26 | 0.51 | 0.05 | 1 | 0.98 | 2:131563757-131567794 | 38.94 | 1 |
| HA1B25B | 2 | 131567527 | 131567763 | 7.15 | 0.52 | 0.05 | 1 | 1 | 2:131563757-131567794 | 38.94 | 1 |
| HA1B25B | 3 | 19821838 | 19822981 | 3.47 | 0.78 | 0.97 | 0.12 | 0.65 | 3:19821802-19823007 | 26.6 | 1 |
| HA1B25B | 3 | 44140640 | 44141384 | 10.26 | 1 | 1 | 1 | 1 | 3:44140622-44141485 | 29.16 | 1 |
| HA1B25B | 3 | 49572072 | 49572927 | 17.28 | 1 | 1 | 1 | 1 | 3:49572068-49572931 | 34.7 | 1 |
| HA1B25B | 3 | 54250109 | 54250380 | 13.02 | 0.51 | 0.05 | 1 | 0.98 | 3:54250109-54254158 | 42.3 | 1 |
| HA1B25B | 3 | 54253894 | 54254155 | 13.17 | 0.52 | 0.05 | 1 | 1 | 3:54250109-54254158 | 42.3 | 1 |
| HA1B25B | 3 | 59937547 | 59938182 | 14.48 | 0.51 | 0.05 | 1 | 0.98 | 3:59937540-59938220 | 27.7 | 1 |
| HA1B25B | 3 | 70604561 | 70605387 | 25.88 | 1 | 1 | 1 | 0.99 | 3:70604555-70605387 | 22.51 | 1 |
| HA1B25B | 3 | 73561881 | 73563062 | 2.41 | 0.77 | 0.99 | 0.41 | 0.57 | 3:73561879-73563101 | 29.85 | 1 |
| HA1B25B | 3 | 83776927 | 83777152 | 9.13 | 0.47 | 0.05 | 1 | 0.88 | 3:83776894-83777945 | 34.2 | 1 |
| HA1B25B | 3 | 83777689 | 83777935 | 13.17 | 0.52 | 0.05 | 1 | 1 | 3:83776894-83777945 | 34.2 | 1 |
| HA1B25B | 3 | 88244141 | 88245055 | 12.38 | 1 | 1 | 1 | 1 | 3:88244141-88245055 | 30.2 | 1 |
| HA1B25B | 4 | 11124004 | 11124362 | 4.13 | 0.99 | 1 | 1 | 0.98 | 4:11123901-11124467 | 25.59 | 1 |
| HA1B25B | 4 | 22222537 | 22223172 | 9.93 | 0.51 | 0.05 | 1 | 0.99 | 4:22222499-22223210 | 23.95 | 1 |
| HA1B25B | 4 | 26530949 | 26531153 | 7.6 | 0.51 | 0.05 | 1 | 0.98 | 4:26530893-26531990 | 29.53 | 1 |
| HA1B25B | 4 | 26531697 | 26531909 | 9.51 | 0.51 | 0.05 | 1 | 0.98 | 4:26530893-26531990 | 29.53 | 1 |
| HA1B25B | 4 | 29234085 | 29234722 | 15.64 | 0.52 | 0.05 | 1 | 1 | 4:29234085-29234741 | 28.49 | 1 |
| HA1B25B | 4 | 30632750 | 30633328 | 10.36 | 0.51 | 0.05 | 1 | 0.98 | 4:30632716-30633375 | 28.76 | 1 |
| HA1B25B | 4 | 68033286 | 68033906 | 18.01 | 0.52 | 0.05 | 1 | 1 | 4:68033286-68033906 | 30.42 | 1 |
| HA1B25B | 4 | 75154549 | 75155039 | 6.34 | 1 | 1 | 1 | 1 | 4:75154549-75155039 | 31.33 | 1 |
| HA1B25B | 4 | 86220862 | 86221793 | 16.14 | 0.99 | 0.99 | 1 | 1 | 4:86220862-86221807 | 32.66 | 1 |
| HA1B25B | 5 | 2705078 | 2706253 | 3.93 | 0.68 | 0.5 | 1 | 0.85 | 5:2705052-2706284 | 30.97 | 1 |
| HA1B25B | 5 | 4202332 | 4203178 | 15.76 | 1 | 1 | 1 | 1 | 5:4202332-4203178 | 33.41 | 1 |
| HA1B25B | 5 | 6164275 | 6165488 | 3.59 | 0.57 | 0.42 | 0 | 0.83 | 5:6164269-6165523 | 19.66 | 1 |
| HA1B25B | 5 | 31303880 | 31305059 | NA | NA | NA | NA | NA | 5:31303866-31305068 | 31.33 | 1 |
| HA1B25B | 5 | 31525368 | 31526240 | 16.02 | 1 | 1 | 1 | 1 | 5:31525368-31526271 | 30.82 | 1 |
| HA1B25B | 5 | 31898687 | 31899859 | 3.76 | 0.61 | 0.73 | 1 | 0.42 | 5:31898626-31899883 | 27.68 | 1 |
| HA1B25B | 5 | 31998157 | 31999015 | 16.3 | 1 | 1 | 1 | 1 | 5:31998141-31999034 | 35.91 | 1 |
| HA1B25B | 6 | 9811209 | 9811604 | 12.33 | 1 | 0.99 | 1 | 1 | 6:9811191-9816444 | 28.6 | 0.99 |
| HA1B25B | 6 | 9816037 | 9816393 | 11.1 | 0.99 | 0.99 | 1 | 0.98 | 6:9811191-9816444 | 28.6 | 0.99 |
| HA1B25B | 6 | 13724874 | 13726011 | NA | NA | NA | NA | NA | 6:13724843-13726022 | 30.74 | 1 |
| HA1B25B | 6 | 33847095 | 33847687 | 18.7 | 0.52 | 0.05 | 1 | 1 | 6:33847042-33847694 | 33.68 | 1 |
| HA1B25B | 7 | 2507955 | 2508579 | 6.45 | 0.52 | 0.05 | 1 | 1 | 7:2507955-2508579 | 31.35 | 1 |
| HA1B25B | 7 | 15504253 | 15504885 | 12.78 | 0.52 | 0.05 | 1 | 1 | 7:15504223-15504887 | 35.05 | 1 |
| HA1B25B | 7 | 17708156 | 17708712 | 21.58 | 0.51 | 0.05 | 1 | 0.98 | 7:17708068-17708753 | 27.55 | 1 |
| HA1B25B | 9 | 1710352 | 1710900 | 19.87 | 0.33 | 0 | 0.24 | 0.73 | 9:1710352-1710900 | 26.77 | 1 |
| HA1B25B | 9 | 21346070 | 21346899 | 13.81 | 1 | 1 | 1 | 1 | 9:21346066-21346914 | 26.96 | 1 |
| HA1B25B | 10 | 9703209 | 9703845 | 18.24 | 0.51 | 0.05 | 1 | 0.98 | 10:9703183-9703845 | 31.69 | 1 |
| HA1B25B | 11 | 7345413 | 7345603 | 5.92 | 0.51 | 0.05 | 1 | 0.98 | 11:7345413-7345603 | 30.7 | 1 |
| HA1B25B | 12 | 3240726 | 3240870 | 16.01 | 0.52 | 1 | 0.24 | 0 | 12:3240697-3240898 | 163.91 | 1 |
| HA1B25B | 15 | 1590642 | 1591244 | 8.17 | 0.52 | 0.05 | 1 | 1 | 15:1590630-1591272 | 27.07 | 1 |
| HA1B25B | 20 | 7661959 | 7662760 | 7.31 | 1 | 1 | 1 | 1 | 20:7661897-7662786 | 30.13 | 1 |
| HA1B25B | JH376323.1 | 17977 | 18119 | 8.24 | 0.28 | 0 | 0 | 0.65 | JH376323.1:17955-18124 | 87.56 | 1 |
| HA1B25B | Z | 18808044 | 18808303 | 13.04 | 0.63 | 0.49 | 1 | 0.75 | Z:18807991-18809115 | 33.35 | 1 |
| HA1B25B | Z | 18808854 | 18809064 | 10.96 | 0.63 | 0.5 | 1 | 0.73 | Z:18807991-18809115 | 33.35 | 1 |
| HA1B25B | Z | 30996774 | 30996888 | 16.67 | 0.56 | 1 | 0.94 | 0 | Z:30996146-31000811 | 23.72 | 0.98 |
| HA1B25B | Z | 32081181 | 32081585 | 11.69 | 1 | 1 | 1 | 1 | Z:32081154-32086293 | 53.28 | 1 |
| HA1B25B | Z | 32085903 | 32086284 | 13.23 | 0.99 | 1 | 1 | 0.98 | Z:32081154-32086293 | 53.28 | 1 |
| HA1B25B | Z | 50872654 | 50873335 | 30.78 | 0.52 | 0.05 | 1 | 1 | Z:50872653-50873337 | 33.65 | 1 |
| HA1B25B | Z | 61304836 | 61306015 | 6.7 | 0.65 | 0.35 | 0.94 | 0.96 | Z:61304819-61306021 | 34.44 | 1 |
| HA1B25B | Z | 78844875 | 78846029 | 19.04 | 0.82 | 1 | 1 | 0.59 | Z:78844863-78846057 | 25.79 | 1 |
| HA1B25B | Z | 79945542 | 79946391 | 13.09 | 1 | 1 | 1 | 1 | Z:79945501-79946432 | 28.95 | 1 |
| HA2A10B | 1 | 8127806 | 8128614 | 14.54 | 1 | 1 | 1 | 1 | 1:8127806-8128614 | 31.33 | 1 |
| HA2A10B | 1 | 31728916 | 31729579 | 9.47 | 0.51 | 0.05 | 1 | 0.98 | 1:31728916-31729579 | 29.53 | 1 |
| HA2A10B | 1 | 32312251 | 32313076 | 28.75 | 1 | 1 | 1 | 0.99 | 1:32312218-32313185 | 28.71 | 1 |
| HA2A10B | 1 | 41318916 | 41319777 | 16.75 | 0.99 | 1 | 1 | 0.98 | 1:41318900-41319777 | 34.4 | 1 |
| HA2A10B | 1 | 42541919 | 42542554 | 27.08 | 0.51 | 0.05 | 1 | 0.98 | 1:42541895-42542614 | 32.17 | 1 |
| HA2A10B | 1 | 43882665 | 43883798 | 34.93 | 0.86 | 0.81 | 1 | 0.91 | 1:43882636-43883815 | 35.97 | 1 |
| HA2A10B | 1 | 47364771 | 47365611 | 13.3 | 1 | 1 | 1 | 1 | 1:47364771-47365611 | 30.25 | 1 |
| HA2A10B | 1 | 99136311 | 99136930 | 12.41 | 0.51 | 0.05 | 1 | 0.98 | 1:99136310-99136953 | 37.5 | 1 |
| HA2A10B | 1 | 113945483 | 113946359 | 10.61 | 0.36 | 0 | 0 | 0.83 | 1:113945460-113946362 | 37.16 | 1 |
| HA2A10B | 1 | 123156456 | 123157316 | 12.03 | 1 | 1 | 1 | 1 | 1:123156456-123157321 | 28.48 | 1 |
| HA2A10B | 1 | 140710397 | 140710773 | 17.87 | 1 | 1 | 1 | 0.99 | 1:140710345-140715423 | 45.32 | 1 |
| HA2A10B | 1 | 140714978 | 140715412 | 15.49 | 1 | 1 | 1 | 1 | 1:140710345-140715423 | 45.32 | 1 |
| HA2A10B | 1 | 146731934 | 146732813 | 26.15 | 0.99 | 1 | 1 | 0.98 | 1:146731910-146732833 | 30.98 | 1 |
| HA2A10B | 1 | 149162810 | 149163191 | 7.13 | 1 | 1 | 1 | 0.99 | 1:149162760-149166227 | 41.72 | 1 |
| HA2A10B | 1 | 149165159 | 149166197 | 12.35 | 0.64 | 0.38 | 0.18 | 1 | 1:149162760-149166227 | 41.72 | 1 |
| HA2A10B | 1 | 160455250 | 160455464 | 8.14 | 0.51 | 0.05 | 1 | 0.98 | 1:160455218-160455708 | 27.13 | 1 |
| HA2A10B | 1 | 160463969 | 160464207 | 12.79 | 0.52 | 0.05 | 1 | 1 | 1:160463921-160464245 | 36.38 | 1 |
| HA2A10B | 1 | 179728775 | 179729463 | 14.21 | 0.52 | 0.05 | 1 | 1 | 1:179728775-179729463 | 27.5 | 1 |
| HA2A10B | 1 | 180746903 | 180747133 | 15.86 | 0.52 | 0.05 | 1 | 1 | 1:180746896-180747970 | 33.62 | 1 |
| HA2A10B | 1 | 180747695 | 180747941 | 16.93 | 0.52 | 0.05 | 1 | 1 | 1:180746896-180747970 | 33.62 | 1 |
| HA2A10B | 1 | 182832922 | 182834040 | 5.43 | 0.92 | 0.88 | 1 | 0.96 | 1:182832847-182834082 | 26.51 | 1 |
| HA2A10B | 1 | 186846435 | 186847073 | 12.9 | 0.52 | 0.05 | 1 | 1 | 1:186846427-186847073 | 33.27 | 1 |
| HA2A10B | 2 | 10512425 | 10512873 | 6.88 | 0.51 | 0.05 | 1 | 0.99 | 2:10512425-10512887 | 30.1 | 1 |
| HA2A10B | 2 | 11018857 | 11019506 | 12.06 | 0.51 | 0.05 | 1 | 0.98 | 2:11018857-11019506 | 36.63 | 1 |
| HA2A10B | 2 | 82574341 | 82575507 | 1.39 | 0.79 | 0.95 | 0 | 0.73 | 2:82574297-82575518 | 32.68 | 1 |
| HA2A10B | 2 | 92070857 | 92071730 | 13.85 | 1 | 1 | 1 | 1 | 2:92070857-92071755 | 31.48 | 1 |
| HA2A10B | 2 | 92857013 | 92858199 | 1.54 | 0.61 | 0.51 | 1 | 0.67 | 2:92856996-92858219 | 29.58 | 1 |
| HA2A10B | 2 | 94594272 | 94594905 | 14.22 | 0.51 | 0.05 | 1 | 0.99 | 2:94594272-94594911 | 29.45 | 1 |
| HA2A10B | 2 | 95480964 | 95481785 | 15.1 | 1 | 1 | 1 | 0.99 | 2:95480957-95481836 | 32.96 | 1 |
| HA2A10B | 2 | 129140834 | 129141707 | 12.67 | 1 | 0.99 | 1 | 1 | 2:129140825-129141707 | 26.32 | 1 |
| HA2A10B | 2 | 131563810 | 131564055 | 12.9 | 0.51 | 0.05 | 1 | 0.98 | 2:131563757-131567794 | 39.61 | 1 |
| HA2A10B | 2 | 131567520 | 131567760 | 17.03 | 0.52 | 0.05 | 1 | 1 | 2:131563757-131567794 | 39.61 | 1 |
| HA2A10B | 2 | 142113885 | 142114521 | 14.08 | 0.51 | 0.05 | 1 | 0.98 | 2:142113866-142114543 | 29.29 | 1 |
| HA2A10B | 3 | 19821848 | 19823007 | 2.33 | 0.95 | 0.9 | 1 | 1 | 3:19821802-19823007 | 27.49 | 1 |
| HA2A10B | 3 | 44140622 | 44141452 | 17.54 | 1 | 1 | 1 | 1 | 3:44140622-44141485 | 31.06 | 1 |
| HA2A10B | 3 | 49572081 | 49572931 | 18.2 | 1 | 1 | 1 | 1 | 3:49572068-49572931 | 36.43 | 1 |
| HA2A10B | 3 | 59937543 | 59938218 | 14.05 | 0.51 | 0.05 | 1 | 0.98 | 3:59937540-59938220 | 32.88 | 1 |
| HA2A10B | 3 | 70604555 | 70605329 | 14.93 | 0.99 | 1 | 1 | 0.98 | 3:70604555-70605387 | 24.06 | 1 |
| HA2A10B | 3 | 73561906 | 73563036 | 1.75 | 0.75 | 0.52 | 1 | 0.99 | 3:73561879-73563101 | 33.08 | 1 |
| HA2A10B | 3 | 79282754 | 79283583 | 15.04 | 0.99 | 1 | 1 | 0.98 | 3:79282754-79283588 | 32.18 | 1 |
| HA2A10B | 3 | 83776922 | 83777130 | 5.61 | 0.46 | 0.05 | 1 | 0.87 | 3:83776894-83777945 | 33.81 | 1 |
| HA2A10B | 3 | 83777691 | 83777927 | 16.07 | 0.46 | 0.05 | 1 | 0.87 | 3:83776894-83777945 | 33.81 | 1 |
| HA2A10B | 3 | 93697707 | 93698548 | 14.62 | 0.99 | 1 | 1 | 0.98 | 3:93697657-93698572 | 31.14 | 1 |
| HA2A10B | 3 | 96957568 | 96957879 | 8.1 | 0.51 | 0.05 | 1 | 0.98 | 3:96957515-96961612 | 28.66 | 1 |
| HA2A10B | 3 | 96961320 | 96961548 | 5.19 | 0.52 | 0.05 | 1 | 1 | 3:96957515-96961612 | 28.66 | 1 |
| HA2A10B | 3 | 102777238 | 102778134 | 16.01 | 1 | 1 | 1 | 1 | 3:102777226-102778143 | 31.84 | 1 |
| HA2A10B | 4 | 11124000 | 11124387 | 12.78 | 0.99 | 1 | 1 | 0.98 | 4:11123901-11124467 | 29.71 | 1 |
| HA2A10B | 4 | 26530893 | 26531127 | 8.52 | 0.51 | 0.05 | 1 | 0.98 | 4:26530893-26531990 | 28.36 | 1 |
| HA2A10B | 4 | 26531714 | 26531923 | 7.64 | 0.51 | 0.05 | 1 | 0.98 | 4:26530893-26531990 | 28.36 | 1 |
| HA2A10B | 4 | 71535538 | 71536391 | 12 | 0.99 | 1 | 1 | 0.98 | 4:71535538-71536391 | 29.14 | 1 |
| HA2A10B | 4 | 72705264 | 72705454 | 6.11 | 0.52 | 0.05 | 1 | 1 | 4:72705264-72705454 | 34.7 | 1 |
| HA2A10B | 4 | 86220873 | 86221790 | 12.25 | 1 | 1 | 1 | 1 | 4:86220862-86221807 | 32.13 | 1 |
| HA2A10B | 5 | 2705077 | 2706260 | 3.63 | 0.78 | 0.88 | 0.41 | 0.71 | 5:2705052-2706284 | 30.8 | 1 |
| HA2A10B | 5 | 4202359 | 4203140 | 16.77 | 1 | 1 | 1 | 1 | 5:4202332-4203178 | 34.26 | 1 |
| HA2A10B | 5 | 6164290 | 6165475 | 2.34 | 0.25 | 0 | 0 | 0.59 | 5:6164269-6165523 | 20.22 | 1 |
| HA2A10B | 5 | 31303895 | 31305052 | 3.38 | 0.44 | 0.4 | 1 | 0.41 | 5:31303866-31305068 | 33.51 | 1 |
| HA2A10B | 5 | 31525401 | 31526271 | 14.32 | 1 | 1 | 1 | 1 | 5:31525368-31526271 | 31.49 | 1 |
| HA2A10B | 5 | 31898704 | 31899853 | 11.02 | 0.75 | 0.88 | 1 | 0.56 | 5:31898626-31899883 | 31.88 | 1 |
| HA2A10B | 5 | 31998148 | 31999028 | 14.77 | 1 | 1 | 1 | 1 | 5:31998141-31999034 | 29.55 | 1 |
| HA2A10B | 5 | 40464023 | 40464894 | 12.23 | 1 | 1 | 1 | 0.99 | 5:40463978-40464915 | 35.65 | 1 |
| HA2A10B | 6 | 9811204 | 9811625 | 14.81 | 0.99 | 0.99 | 1 | 1 | 6:9811191-9816444 | 28.61 | 0.98 |
| HA2A10B | 6 | 9816042 | 9816404 | 12.49 | 0.99 | 0.99 | 1 | 0.98 | 6:9811191-9816444 | 28.61 | 0.98 |
| HA2A10B | 6 | 13724866 | 13726002 | 3.47 | 0.6 | 0.51 | 0.82 | 0.68 | 6:13724843-13726022 | 33.6 | 1 |
| HA2A10B | 6 | 33847042 | 33847694 | 30.66 | 0.52 | 0.05 | 1 | 1 | 6:33847042-33847694 | 33.27 | 1 |
| HA2A10B | 7 | 9022757 | 9023414 | 17.1 | 0.52 | 0.05 | 1 | 1 | 7:9022731-9023457 | 30.83 | 1 |
| HA2A10B | 7 | 17708102 | 17708734 | 26.06 | 0.48 | 0 | 1 | 0.98 | 7:17708068-17708753 | 31.15 | 1 |
| HA2A10B | 8 | 9208402 | 9209273 | 8.84 | 1 | 1 | 1 | 0.99 | 8:9208394-9209273 | 22.67 | 1 |
| HA2A10B | 9 | 18008576 | 18009249 | 12.05 | 0.52 | 0.05 | 1 | 1 | 9:18008576-18009249 | 24.27 | 1 |
| HA2A10B | 9 | 21346070 | 21346910 | 12.54 | 1 | 1 | 1 | 0.99 | 9:21346066-21346914 | 31.57 | 1 |
| HA2A10B | 10 | 9703183 | 9703821 | 15.51 | 0.51 | 0.05 | 1 | 0.98 | 10:9703183-9703845 | 33.96 | 1 |
| HA2A10B | 12 | 3240723 | 3240867 | 31.06 | 0.56 | 1 | 0.82 | 0 | 12:3240697-3240898 | 251.69 | 1 |
| HA2A10B | 20 | 7661913 | 7662758 | 15.74 | 1 | 1 | 1 | 1 | 20:7661897-7662786 | 33.44 | 1 |
| HA2A10B | 24 | 5789018 | 5789217 | 7.7 | 0.52 | 0.05 | 1 | 1 | 24:5789018-5789217 | 36.63 | 1 |
| HA2A10B | AADN03009901.1 | 868 | 1149 | 14.69 | 0.51 | 0.05 | 1 | 0.99 | AADN03009901.1:851-1161 | 32.57 | 1 |
| HA2A10B | Z | 18808038 | 18808302 | 9.43 | 0.66 | 0.55 | 1 | 0.75 | Z:18807991-18809115 | 30.03 | 1 |
| HA2A10B | Z | 18808834 | 18809073 | 10.63 | 0.63 | 0.49 | 1 | 0.75 | Z:18807991-18809115 | 30.03 | 1 |
| HA2A10B | Z | 30996687 | 30997223 | 26.82 | 1 | 1 | 1 | 1 | Z:30996146-31000811 | 30.7 | 1 |
| HA2A10B | Z | 32081158 | 32081565 | 15.88 | 1 | 1 | 1 | 1 | Z:32081154-32086293 | 53.58 | 1 |
| HA2A10B | Z | 32085877 | 32086289 | 15.73 | 0.99 | 1 | 1 | 0.98 | Z:32081154-32086293 | 53.58 | 1 |
| HA2A10B | Z | 50872653 | 50873303 | 16.39 | 0.52 | 0.05 | 1 | 1 | Z:50872653-50873337 | 40.33 | 1 |
| HA2A10B | Z | 61304831 | 61306021 | 5.03 | 0.83 | 0.68 | 1 | 0.98 | Z:61304819-61306021 | 36.57 | 1 |
| HA2A10B | Z | 65689607 | 65690259 | 17.58 | 0.52 | 0.05 | 1 | 1 | Z:65689607-65690259 | 35.12 | 1 |
| HA2A10B | Z | 78844878 | 78846035 | 31.01 | 0.83 | 1 | 1 | 0.6 | Z:78844863-78846057 | 31.6 | 0.99 |
| HA2A10B | Z | 79945542 | 79946402 | 12.85 | 1 | 1 | 1 | 1 | Z:79945501-79946432 | 27.05 | 1 |
| HA2A25B | 1 | 8127816 | 8128600 | 17.69 | 1 | 1 | 1 | 1 | 1:8127806-8128614 | 31.07 | 1 |
| HA2A25B | 1 | 32312230 | 32313076 | 36.61 | 0.99 | 1 | 1 | 0.98 | 1:32312218-32313185 | 30 | 1 |
| HA2A25B | 1 | 41318904 | 41319755 | 22.56 | 0.99 | 1 | 1 | 0.98 | 1:41318900-41319777 | 34.54 | 1 |
| HA2A25B | 1 | 89899759 | 89900538 | 17.16 | 0.99 | 1 | 1 | 0.98 | 1:89899733-89900560 | 32.27 | 1 |
| HA2A25B | 1 | 99136345 | 99136906 | 12.69 | 0.5 | 0.05 | 1 | 0.97 | 1:99136310-99136953 | 34.03 | 1 |
| HA2A25B | 1 | 113945460 | 113946331 | 25.06 | 0.36 | 0 | 0 | 0.83 | 1:113945460-113946362 | 34.32 | 1 |
| HA2A25B | 1 | 123156466 | 123157321 | 14.49 | 1 | 1 | 1 | 1 | 1:123156456-123157321 | 31.01 | 1 |
| HA2A25B | 1 | 140037593 | 140038154 | 13.58 | 0.51 | 0.05 | 1 | 0.99 | 1:140037593-140038154 | 32.08 | 1 |
| HA2A25B | 1 | 140710415 | 140710777 | 16.24 | 1 | 1 | 1 | 0.99 | 1:140710345-140715423 | 48.92 | 1 |
| HA2A25B | 1 | 140714961 | 140715423 | 15.63 | 1 | 1 | 1 | 1 | 1:140710345-140715423 | 48.92 | 1 |
| HA2A25B | 1 | 141097681 | 141098528 | 25.12 | 0.51 | 0.05 | 1 | 0.99 | 1:141097673-141098554 | 32.57 | 1 |
| HA2A25B | 1 | 146731918 | 146732807 | 18.12 | 0.99 | 1 | 1 | 0.98 | 1:146731910-146732833 | 31.09 | 1 |
| HA2A25B | 1 | 160455232 | 160455499 | 7.74 | 0.51 | 0.05 | 1 | 0.98 | 1:160455218-160455708 | 25.7 | 1 |
| HA2A25B | 1 | 160463963 | 160464230 | 11.5 | 0.52 | 0.05 | 1 | 1 | 1:160463921-160464245 | 33.48 | 1 |
| HA2A25B | 1 | 180746902 | 180747131 | 16.65 | 0.51 | 0.05 | 1 | 0.99 | 1:180746896-180747970 | 34.5 | 1 |
| HA2A25B | 1 | 180747681 | 180747927 | 13.48 | 0.52 | 0.05 | 1 | 1 | 1:180746896-180747970 | 34.5 | 1 |
| HA2A25B | 1 | 182832896 | 182834057 | 7.16 | 0.87 | 0.95 | 1 | 0.77 | 1:182832847-182834082 | 31.13 | 1 |
| HA2A25B | 1 | 185856858 | 185857444 | 12.47 | 0.51 | 0.05 | 1 | 0.98 | 1:185856831-185857511 | 30.93 | 1 |
| HA2A25B | 1 | 188148838 | 188149727 | 9.25 | 1 | 1 | 1 | 1 | 1:188148835-188149736 | 28.18 | 1 |
| HA2A25B | 2 | 3491426 | 3492255 | 15.82 | 1 | 1 | 1 | 1 | 2:3491394-3492279 | 32.03 | 1 |
| HA2A25B | 2 | 23631823 | 23632481 | 8.08 | 0.52 | 0.05 | 1 | 1 | 2:23631823-23632481 | 29.8 | 1 |
| HA2A25B | 2 | 50798333 | 50798914 | 14.16 | 0.51 | 0.05 | 1 | 0.98 | 2:50798296-50798951 | 35.82 | 1 |
| HA2A25B | 2 | 53111795 | 53112004 | 6.45 | 0.8 | 0.63 | 1 | 0.98 | 2:53111756-53112017 | 32.03 | 1 |
| HA2A25B | 2 | 80333672 | 80334224 | 11.92 | 0.51 | 0.05 | 1 | 0.98 | 2:80333585-80334273 | 33.44 | 1 |
| HA2A25B | 2 | 82574337 | 82575492 | 1.83 | 0.44 | 0.86 | 0 | 0 | 2:82574297-82575518 | 31.44 | 1 |
| HA2A25B | 2 | 92857074 | 92858171 | 1 | 0.4 | 0.72 | 0.65 | 0 | 2:92856996-92858219 | 27.19 | 1 |
| HA2A25B | 2 | 93341773 | 93342126 | 9.82 | 1 | 1 | 1 | 1 | 2:93341773-93342126 | 31.2 | 1 |
| HA2A25B | 2 | 95481033 | 95481781 | 14.09 | 1 | 1 | 1 | 0.99 | 2:95480957-95481836 | 36.06 | 1 |
| HA2A25B | 2 | 129140856 | 129141690 | 16.18 | 1 | 0.99 | 1 | 1 | 2:129140825-129141707 | 29.17 | 1 |
| HA2A25B | 2 | 131563781 | 131564030 | 18.42 | 0.51 | 0.05 | 1 | 0.98 | 2:131563757-131567794 | 33.69 | 1 |
| HA2A25B | 2 | 131567503 | 131567781 | 11.96 | 0.52 | 0.05 | 1 | 1 | 2:131563757-131567794 | 33.69 | 1 |
| HA2A25B | 2 | 142113883 | 142114518 | 11.28 | 0.51 | 0.05 | 1 | 0.98 | 2:142113866-142114543 | 32.07 | 1 |
| HA2A25B | 2 | 142512904 | 142513144 | 4.17 | 0.51 | 0.05 | 1 | 0.98 | 2:142512897-142513907 | 26.7 | 1 |
| HA2A25B | 2 | 142513664 | 142513907 | 11.77 | 0.51 | 0.05 | 1 | 0.98 | 2:142512897-142513907 | 26.7 | 1 |
| HA2A25B | 3 | 19821832 | 19822962 | 2.33 | 0.68 | 0.72 | 0 | 0.73 | 3:19821802-19823007 | 24.79 | 1 |
| HA2A25B | 3 | 34783123 | 34783362 | 4.45 | 0.51 | 0.05 | 1 | 0.98 | 3:34782953-34783379 | 36.23 | 1 |
| HA2A25B | 3 | 59937551 | 59938183 | 11.61 | 0.51 | 0.05 | 1 | 0.98 | 3:59937540-59938220 | 30.23 | 1 |
| HA2A25B | 3 | 63745335 | 63745967 | 37.49 | 0.52 | 0.05 | 1 | 1 | 3:63745322-63745967 | 34.05 | 1 |
| HA2A25B | 3 | 70604586 | 70605368 | 10.99 | 0.99 | 1 | 1 | 0.97 | 3:70604555-70605387 | 22.01 | 0.98 |
| HA2A25B | 3 | 73561894 | 73563062 | 1.88 | 0.73 | 0.47 | 1 | 0.99 | 3:73561879-73563101 | 35.76 | 1 |
| HA2A25B | 3 | 83407675 | 83408482 | 24.98 | 1 | 1 | 1 | 1 | 3:83407619-83408514 | 24.18 | 0.83 |
| HA2A25B | 3 | 83776948 | 83777144 | 12.65 | 0.46 | 0.05 | 1 | 0.87 | 3:83776894-83777945 | 34.48 | 1 |
| HA2A25B | 3 | 83777672 | 83777916 | 12.84 | 0.46 | 0.05 | 1 | 0.87 | 3:83776894-83777945 | 34.48 | 1 |
| HA2A25B | 3 | 86869634 | 86870258 | 10.32 | 0.52 | 0.05 | 1 | 0.99 | 3:86869588-86870258 | 30.93 | 1 |
| HA2A25B | 3 | 102777298 | 102778102 | 14.05 | 1 | 1 | 1 | 1 | 3:102777226-102778143 | 29.87 | 1 |
| HA2A25B | 4 | 22222591 | 22223156 | 12.95 | 0.51 | 0.05 | 1 | 0.99 | 4:22222499-22223210 | 28.32 | 1 |
| HA2A25B | 4 | 26530946 | 26531149 | 8.88 | 0.51 | 0.05 | 1 | 0.98 | 4:26530893-26531990 | 30.29 | 1 |
| HA2A25B | 4 | 26531696 | 26531907 | 9.55 | 0.51 | 0.05 | 1 | 0.98 | 4:26530893-26531990 | 30.29 | 1 |
| HA2A25B | 4 | 27394587 | 27395209 | 14.56 | 0.52 | 0.05 | 1 | 1 | 4:27394572-27395212 | 33.11 | 1 |
| HA2A25B | 4 | 30632752 | 30633336 | 12.69 | 0.51 | 0.05 | 1 | 0.98 | 4:30632716-30633375 | 32.97 | 1 |
| HA2A25B | 4 | 48096947 | 48097589 | 17.51 | 0.52 | 0.05 | 1 | 1 | 4:48096947-48097615 | 30.15 | 1 |
| HA2A25B | 4 | 70463813 | 70464439 | 16.47 | 0.52 | 0.05 | 1 | 1 | 4:70463742-70464439 | 40.03 | 1 |
| HA2A25B | 4 | 70551829 | 70552625 | 12.82 | 0.99 | 1 | 1 | 0.98 | 4:70551829-70552669 | 25.96 | 1 |
| HA2A25B | 5 | 2705099 | 2706262 | 5.08 | 0.85 | 0.99 | 1 | 0.67 | 5:2705052-2706284 | 30.93 | 1 |
| HA2A25B | 5 | 6164294 | 6165476 | 2.43 | 0.24 | 0 | 0 | 0.56 | 5:6164269-6165523 | 23.87 | 1 |
| HA2A25B | 5 | 31303897 | 31305051 | 1.55 | 0.57 | 0.16 | 1 | 1 | 5:31303866-31305068 | 34.43 | 1 |
| HA2A25B | 5 | 31898741 | 31899850 | 13.4 | 0.74 | 0.86 | 1 | 0.57 | 5:31898626-31899883 | 31.25 | 1 |
| HA2A25B | 5 | 31998162 | 31999017 | 15.52 | 1 | 1 | 1 | 1 | 5:31998141-31999034 | 29.33 | 1 |
| HA2A25B | 5 | 39163523 | 39164645 | 7.95 | 0.77 | 0.99 | 1 | 0.49 | 5:39163484-39164645 | 28.99 | 1 |
| HA2A25B | 5 | 40464016 | 40464872 | 12.83 | 0.99 | 0.99 | 1 | 0.99 | 5:40463978-40464915 | 30.58 | 1 |
| HA2A25B | 6 | 9811217 | 9811598 | 14.18 | 1 | 0.99 | 1 | 1 | 6:9811191-9816444 | 29.76 | 0.99 |
| HA2A25B | 6 | 9816035 | 9816432 | 15.62 | 0.99 | 0.99 | 1 | 0.98 | 6:9811191-9816444 | 29.76 | 0.99 |
| HA2A25B | 6 | 13724847 | 13726003 | 3.04 | 0.78 | 0.96 | 0.41 | 0.62 | 6:13724843-13726022 | 33.33 | 1 |
| HA2A25B | 7 | 15504266 | 15504826 | 15.86 | 0.52 | 0.05 | 1 | 1 | 7:15504223-15504887 | 35.92 | 1 |
| HA2A25B | 8 | 9208413 | 9209260 | 5.18 | 1 | 1 | 1 | 0.99 | 8:9208394-9209273 | 20.04 | 1 |
| HA2A25B | 10 | 9703203 | 9703817 | 16.6 | 0.51 | 0.05 | 1 | 0.98 | 10:9703183-9703845 | 31.75 | 1 |
| HA2A25B | 11 | 11174348 | 11174689 | 7.05 | 0.99 | 1 | 1 | 0.98 | 11:11174286-11174756 | 33.07 | 1 |
| HA2A25B | 12 | 3240723 | 3240866 | 47.77 | 0.58 | 1 | 1 | 0.02 | 12:3240697-3240898 | 151.86 | 1 |
| HA2A25B | 13 | 1868296 | 1868928 | 11.22 | 0.51 | 0.05 | 1 | 0.98 | 13:1868296-1868957 | 31.22 | 1 |
| HA2A25B | 15 | 1590630 | 1591228 | 14.05 | 0.52 | 0.05 | 1 | 1 | 15:1590630-1591272 | 28.48 | 1 |
| HA2A25B | AADN03017712.1 | 157 | 1286 | 14.95 | 1 | 1 | 1 | 1 | AADN03017712.1:157-1286 | 10694.45 | 1 |
| HA2A25B | Z | 18808042 | 18808297 | 14.89 | 0.52 | 0.28 | 1 | 0.73 | Z:18807991-18809115 | 34.98 | 1 |
| HA2A25B | Z | 18808811 | 18809066 | 17.19 | 0.63 | 0.49 | 1 | 0.73 | Z:18807991-18809115 | 34.98 | 1 |
| HA2A25B | Z | 32081159 | 32081599 | 10.52 | 1 | 1 | 1 | 1 | Z:32081154-32086293 | 51.34 | 1 |
| HA2A25B | Z | 32085872 | 32086277 | 16.62 | 0.99 | 1 | 1 | 0.98 | Z:32081154-32086293 | 51.34 | 1 |
| HA2A25B | Z | 47830813 | 47831469 | 12.26 | 0.51 | 0.05 | 1 | 0.99 | Z:47830813-47831469 | 31.12 | 1 |
| HA2A25B | Z | 50872673 | 50873303 | 31.49 | 0.52 | 0.05 | 1 | 1 | Z:50872653-50873337 | 35.28 | 1 |
| HA2A25B | Z | 61304862 | 61305993 | 6.26 | 0.75 | 0.52 | 1 | 0.98 | Z:61304819-61306021 | 33.81 | 1 |
| HA2A25B | Z | 79945552 | 79946385 | 24.21 | 1 | 1 | 1 | 1 | Z:79945501-79946432 | 28.92 | 1 |
| HB1A16A | 1 | 37362031 | 37362635 | 15.78 | 0.52 | 0.05 | 1 | 1 | 1:37362031-37362653 | 31 | 1 |
| HB1A16A | 1 | 42541956 | 42542577 | 13.35 | 0.52 | 0.05 | 1 | 1 | 1:42541895-42542614 | 29.85 | 1 |
| HB1A16A | 1 | 99136332 | 99136934 | 31.79 | 0.51 | 0.05 | 1 | 0.98 | 1:99136310-99136953 | 35.3 | 1 |
| HB1A16A | 1 | 113945518 | 113946344 | 12.18 | 0.36 | 0 | 0 | 0.83 | 1:113945460-113946362 | 35.81 | 1 |
| HB1A16A | 1 | 140710382 | 140710794 | 12.88 | 1 | 1 | 1 | 0.99 | 1:140710345-140715423 | 52.86 | 1 |
| HB1A16A | 1 | 140715007 | 140715385 | 10.47 | 1 | 1 | 1 | 1 | 1:140710345-140715423 | 52.86 | 1 |
| HB1A16A | 1 | 142068256 | 142068910 | 13 | 0.51 | 0.05 | 1 | 0.98 | 1:142068256-142068910 | 30.67 | 1 |
| HB1A16A | 1 | 146731933 | 146732792 | 15.87 | 0.99 | 1 | 1 | 0.98 | 1:146731910-146732833 | 31.29 | 1 |
| HB1A16A | 1 | 148564593 | 148564847 | 9.2 | 0.52 | 0.05 | 1 | 1 | 1:148564562-148568615 | 51.66 | 1 |
| HB1A16A | 1 | 148568370 | 148568589 | 8.02 | 0.51 | 0.05 | 1 | 0.98 | 1:148564562-148568615 | 51.66 | 1 |
| HB1A16A | 1 | 153995978 | 153996637 | 10.11 | 0.51 | 0.05 | 1 | 0.98 | 1:153995978-153996637 | 31.3 | 1 |
| HB1A16A | 1 | 160455234 | 160455488 | 12 | 0.51 | 0.05 | 1 | 0.98 | 1:160455218-160455708 | 31.53 | 1 |
| HB1A16A | 1 | 160463987 | 160464219 | 12.95 | 0.52 | 0.05 | 1 | 1 | 1:160463921-160464245 | 30.56 | 1 |
| HB1A16A | 1 | 163501551 | 163501792 | 12.01 | 0.52 | 0.05 | 1 | 1 | 1:163501535-163501830 | 29.57 | 1 |
| HB1A16A | 1 | 180746920 | 180747133 | 8.01 | 0.51 | 0.05 | 1 | 0.99 | 1:180746896-180747970 | 24.74 | 1 |
| HB1A16A | 1 | 180747696 | 180747920 | 9.21 | 0.52 | 0.05 | 1 | 1 | 1:180746896-180747970 | 24.74 | 1 |
| HB1A16A | 1 | 182832886 | 182834039 | 15.39 | 0.91 | 0.99 | 1 | 0.81 | 1:182832847-182834082 | 29.01 | 1 |
| HB1A16A | 1 | 184347927 | 184348781 | 9.13 | 1 | 1 | 1 | 1 | 1:184347893-184348804 | 27.44 | 1 |
| HB1A16A | 1 | 185856848 | 185857469 | 14.97 | 0.51 | 0.05 | 1 | 0.98 | 1:185856831-185857511 | 34.14 | 1 |
| HB1A16A | 2 | 1183419 | 1184047 | 9.54 | 0.52 | 0.05 | 1 | 1 | 2:1183407-1184047 | 29.23 | 1 |
| HB1A16A | 2 | 10512610 | 10512779 | 7.81 | 0.4 | 0.05 | 1 | 0.73 | 2:10512425-10512887 | 28.01 | 1 |
| HB1A16A | 2 | 53111781 | 53111980 | 9.11 | 0.82 | 0.66 | 1 | 0.98 | 2:53111756-53112017 | 28.03 | 1 |
| HB1A16A | 2 | 65341363 | 65341959 | 14.21 | 0.51 | 0.05 | 1 | 0.98 | 2:65341352-65341989 | 32.96 | 1 |
| HB1A16A | 2 | 75447503 | 75448357 | 16.33 | 1 | 1 | 1 | 1 | 2:75447503-75448357 | 30.54 | 1 |
| HB1A16A | 2 | 80333602 | 80334249 | 10.74 | 0.51 | 0.05 | 1 | 0.98 | 2:80333585-80334273 | 26.67 | 0.99 |
| HB1A16A | 2 | 81965001 | 81965255 | 7.36 | 0.51 | 0.05 | 1 | 0.99 | 2:81965001-81966072 | 29.26 | 1 |
| HB1A16A | 2 | 81965803 | 81966022 | 10.21 | 0.52 | 0.05 | 1 | 1 | 2:81965001-81966072 | 29.26 | 1 |
| HB1A16A | 2 | 82574341 | 82575508 | 3.74 | 0.76 | 0.93 | 1 | 0.52 | 2:82574297-82575518 | 32.4 | 1 |
| HB1A16A | 2 | 92070917 | 92071723 | 14.79 | 1 | 1 | 1 | 1 | 2:92070857-92071755 | 29.98 | 1 |
| HB1A16A | 2 | 104297204 | 104297861 | 19.05 | 0.52 | 0.05 | 1 | 1 | 2:104297204-104297861 | 32.52 | 1 |
| HB1A16A | 2 | 124765119 | 124765679 | 16.3 | 0.52 | 0.05 | 1 | 1 | 2:124765081-124765718 | 29.55 | 1 |
| HB1A16A | 2 | 129140835 | 129141696 | 14.09 | 1 | 0.99 | 1 | 1 | 2:129140825-129141707 | 28.9 | 1 |
| HB1A16A | 2 | 147548249 | 147548775 | 5.46 | 0.52 | 0.05 | 1 | 1 | 2:147548249-147548775 | 21.72 | 1 |
| HB1A16A | 3 | 19821846 | 19822977 | 8.96 | 0.98 | 0.98 | 1 | 0.98 | 3:19821802-19823007 | 31.01 | 1 |
| HB1A16A | 3 | 34783136 | 34783379 | 9.47 | 0.51 | 0.05 | 1 | 0.98 | 3:34782953-34783379 | 41.04 | 1 |
| HB1A16A | 3 | 44140627 | 44141484 | 16.13 | 1 | 1 | 1 | 1 | 3:44140622-44141485 | 29.84 | 1 |
| HB1A16A | 3 | 49572069 | 49572917 | 14.29 | 1 | 1 | 1 | 1 | 3:49572068-49572931 | 33 | 1 |
| HB1A16A | 3 | 59937552 | 59938220 | 8.75 | 0.51 | 0.05 | 1 | 0.98 | 3:59937540-59938220 | 32.34 | 1 |
| HB1A16A | 3 | 72558723 | 72559573 | 15.91 | 1 | 1 | 1 | 1 | 3:72558723-72559573 | 30 | 1 |
| HB1A16A | 3 | 73561893 | 73563049 | 2.96 | 0.63 | 0.64 | 0.18 | 0.68 | 3:73561879-73563101 | 31.02 | 1 |
| HB1A16A | 3 | 83407692 | 83408446 | 26.95 | 1 | 1 | 1 | 1 | 3:83407619-83408514 | 25.36 | 0.82 |
| HB1A16A | 3 | 83776930 | 83777137 | 10.14 | 0.48 | 0.05 | 1 | 0.93 | 3:83776894-83777945 | 31.08 | 1 |
| HB1A16A | 3 | 83777711 | 83777923 | 12.53 | 0.51 | 0.05 | 1 | 0.98 | 3:83776894-83777945 | 31.08 | 1 |
| HB1A16A | 3 | 84889113 | 84889407 | 9.47 | 0.52 | 0.05 | 1 | 1 | 3:84889113-84893120 | 33.07 | 1 |
| HB1A16A | 3 | 84892910 | 84893120 | 8.34 | 0.51 | 0.05 | 1 | 0.98 | 3:84889113-84893120 | 33.07 | 1 |
| HB1A16A | 3 | 96957539 | 96957806 | 8.98 | 0.51 | 0.05 | 1 | 0.98 | 3:96957515-96961612 | 31.02 | 1 |
| HB1A16A | 3 | 96961337 | 96961579 | 5.56 | 0.52 | 0.05 | 1 | 1 | 3:96957515-96961612 | 31.02 | 1 |
| HB1A16A | 3 | 102777236 | 102778143 | 27.43 | 1 | 1 | 1 | 1 | 3:102777226-102778143 | 28.95 | 1 |
| HB1A16A | 4 | 11124001 | 11124385 | 4.96 | 0.99 | 1 | 1 | 0.98 | 4:11123901-11124467 | 24.05 | 1 |
| HB1A16A | 4 | 22222545 | 22223158 | 9.59 | 0.51 | 0.05 | 1 | 0.99 | 4:22222499-22223210 | 30.01 | 1 |
| HB1A16A | 4 | 26531686 | 26531924 | 5.48 | 0.51 | 0.05 | 1 | 0.98 | 4:26530893-26531990 | 24.95 | 1 |
| HB1A16A | 4 | 30632765 | 30633373 | 17.16 | 0.51 | 0.05 | 1 | 0.98 | 4:30632716-30633375 | 33.06 | 1 |
| HB1A16A | 4 | 62324172 | 62324557 | 4.96 | 1 | 1 | 1 | 0.99 | 4:62324172-62324557 | 33.06 | 1 |
| HB1A16A | 4 | 70551922 | 70552588 | 6.96 | 0.99 | 1 | 1 | 0.98 | 4:70551829-70552669 | 18.52 | 1 |
| HB1A16A | 5 | 2705063 | 2706260 | 4.78 | 0.8 | 0.88 | 1 | 0.69 | 5:2705052-2706284 | 29.81 | 1 |
| HB1A16A | 5 | 31303906 | 31305068 | NA | NA | NA | NA | NA | 5:31303866-31305068 | 33.42 | 1 |
| HB1A16A | 5 | 31898737 | 31899847 | 18.66 | 0.82 | 0.99 | 1 | 0.58 | 5:31898626-31899883 | 33.52 | 1 |
| HB1A16A | 5 | 31998164 | 31999007 | 15.07 | 1 | 1 | 1 | 1 | 5:31998141-31999034 | 32.87 | 1 |
| HB1A16A | 6 | 9811223 | 9811600 | 13.42 | 1 | 0.99 | 1 | 1 | 6:9811191-9816444 | 28.26 | 0.99 |
| HB1A16A | 6 | 9816033 | 9816382 | 9.8 | 0.97 | 0.99 | 1 | 0.94 | 6:9811191-9816444 | 28.26 | 0.99 |
| HB1A16A | 6 | 13724843 | 13726014 | 1 | 0.28 | 0.55 | 0 | 0 | 6:13724843-13726022 | 35.02 | 1 |
| HB1A16A | 7 | 8070564 | 8071157 | 13.25 | 0.52 | 0.05 | 1 | 1 | 7:8070564-8071157 | 31.1 | 1 |
| HB1A16A | 7 | 17708090 | 17708738 | 25.34 | 0.51 | 0.04 | 1 | 1 | 7:17708068-17708753 | 31.78 | 1 |
| HB1A16A | 7 | 29830070 | 29830722 | 13.37 | 0.51 | 0.05 | 1 | 0.98 | 7:29830070-29830722 | 31.63 | 1 |
| HB1A16A | 8 | 9208412 | 9209211 | 13.07 | 1 | 1 | 1 | 1 | 8:9208394-9209273 | 23.88 | 1 |
| HB1A16A | 9 | 18008612 | 18009213 | 16.69 | 0.52 | 0.05 | 1 | 1 | 9:18008576-18009249 | 23.63 | 1 |
| HB1A16A | 9 | 21346066 | 21346914 | 14.48 | 1 | 1 | 1 | 1 | 9:21346066-21346914 | 29.49 | 1 |
| HB1A16A | 12 | 3240724 | 3240869 | 36.09 | 0.58 | 1 | 1 | 0.03 | 12:3240697-3240898 | 210.81 | 1 |
| HB1A16A | 13 | 1868364 | 1868936 | 11.98 | 0.51 | 0.05 | 1 | 0.99 | 13:1868296-1868957 | 28.5 | 1 |
| HB1A16A | 17 | 180674 | 181540 | 25.19 | 0.99 | 0.99 | 1 | 0.98 | 17:180636-181570 | 25.86 | 1 |
| HB1A16A | 19 | 2694054 | 2694923 | 13.56 | 0.99 | 1 | 1 | 0.98 | 19:2694054-2694923 | 32.4 | 1 |
| HB1A16A | AADN03009901.1 | 851 | 1135 | 5.02 | 0.51 | 0.05 | 1 | 0.99 | AADN03009901.1:851-1161 | 26.83 | 1 |
| HB1A16A | JH375157.1 | 382 | 499 | 14.46 | 0.52 | 0.05 | 1 | 1 | JH375157.1:356-536 | 26.52 | 1 |
| HB1A16A | JH376323.1 | 928 | 1037 | 5.25 | 0.46 | 0.9 | 0 | 0 | JH376323.1:928-1072 | 97.73 | 1 |
| HB1A16A | Z | 15470956 | 15471752 | 8.99 | 1 | 1 | 1 | 1 | Z:15470956-15471752 | 24.01 | 1 |
| HB1A16A | Z | 18808020 | 18809082 | NA | NA | NA | NA | NA | Z:18807991-18809115 | 30.11 | 1 |
| HB1A16A | Z | 32081179 | 32081570 | 15.6 | 1 | 1 | 1 | 1 | Z:32081154-32086293 | 68.62 | 1 |
| HB1A16A | Z | 32085901 | 32086265 | 15.98 | 0.99 | 1 | 1 | 0.98 | Z:32081154-32086293 | 68.62 | 1 |
| HB1A16A | Z | 50872717 | 50873337 | 15.11 | 0.52 | 0.05 | 1 | 1 | Z:50872653-50873337 | 35.08 | 1 |
| HB1A16A | Z | 61304833 | 61305996 | 7.9 | 0.78 | 0.64 | 0.94 | 0.91 | Z:61304819-61306021 | 34.38 | 1 |
| HB1A16A | Z | 78844863 | 78846045 | 11.81 | 0.95 | 1 | 0.35 | 0.98 | Z:78844863-78846057 | 21.38 | 0.99 |
| HB1B21B | 1 | 28836094 | 28836942 | 31.25 | 1 | 1 | 1 | 1 | 1:28836094-28836942 | 46.62 | 1 |
| HB1B21B | 1 | 32312250 | 32313072 | 31.07 | 0.99 | 1 | 1 | 0.98 | 1:32312218-32313185 | 25.59 | 1 |
| HB1B21B | 1 | 42541962 | 42542564 | 11.45 | 0.51 | 0.05 | 1 | 0.98 | 1:42541895-42542614 | 34.25 | 1 |
| HB1B21B | 1 | 63571454 | 63572041 | 18.83 | 0.52 | 0.05 | 1 | 1 | 1:63571454-63572079 | 34.2 | 1 |
| HB1B21B | 1 | 67550483 | 67551078 | 11.76 | 1 | 1 | 1 | 1 | 1:67550436-67551166 | 32.94 | 1 |
| HB1B21B | 1 | 89899737 | 89900560 | 15.13 | 1 | 1 | 1 | 0.99 | 1:89899733-89900560 | 29.4 | 1 |
| HB1B21B | 1 | 96182405 | 96182609 | 6.64 | 0.52 | 0.05 | 1 | 1 | 1:96182372-96182610 | 33.65 | 1 |
| HB1B21B | 1 | 140710390 | 140710814 | 17.16 | 1 | 1 | 1 | 0.99 | 1:140710345-140715423 | 49.22 | 1 |
| HB1B21B | 1 | 140714976 | 140715384 | 13.41 | 1 | 1 | 1 | 1 | 1:140710345-140715423 | 49.22 | 1 |
| HB1B21B | 1 | 146731923 | 146732773 | 12.44 | 0.99 | 1 | 1 | 0.98 | 1:146731910-146732833 | 33.79 | 1 |
| HB1B21B | 1 | 160455269 | 160455520 | 7.98 | 0.51 | 0.05 | 1 | 0.98 | 1:160455218-160455708 | 25.45 | 1 |
| HB1B21B | 1 | 160463944 | 160464214 | 12.41 | 0.52 | 0.05 | 1 | 1 | 1:160463921-160464245 | 35.83 | 1 |
| HB1B21B | 1 | 163906829 | 163907037 | 8.47 | 0.52 | 0.05 | 1 | 1 | 1:163906737-163910850 | 27.03 | 1 |
| HB1B21B | 1 | 164415013 | 164415258 | 4.92 | 0.51 | 0.05 | 1 | 0.98 | 1:164414998-164416037 | 29.98 | 1 |
| HB1B21B | 1 | 164415771 | 164416008 | 8.85 | 0.51 | 0.05 | 1 | 0.98 | 1:164414998-164416037 | 29.98 | 1 |
| HB1B21B | 1 | 171152000 | 171152790 | 15.25 | 0.99 | 1 | 1 | 0.98 | 1:171152000-171152790 | 33.91 | 1 |
| HB1B21B | 1 | 180746931 | 180747916 | 8.53 | 0.51 | 0.03 | 1 | 1 | 1:180746896-180747970 | 33.59 | 1 |
| HB1B21B | 1 | 182832947 | 182834029 | 9.96 | 0.96 | 0.94 | 1 | 0.98 | 1:182832847-182834082 | 29.36 | 1 |
| HB1B21B | 1 | 184347959 | 184348771 | 11.7 | 1 | 1 | 1 | 1 | 1:184347893-184348804 | 28.36 | 1 |
| HB1B21B | 1 | 186846436 | 186847037 | 10.55 | 0.52 | 0.05 | 1 | 1 | 1:186846427-186847073 | 27.53 | 1 |
| HB1B21B | 2 | 7818049 | 7818297 | 4.93 | 0.51 | 0.05 | 1 | 0.99 | 2:7818049-7818465 | 29.02 | 1 |
| HB1B21B | 2 | 16258680 | 16259495 | 13.76 | 1 | 1 | 1 | 1 | 2:16258680-16259495 | 34.27 | 1 |
| HB1B21B | 2 | 50798308 | 50798930 | 12.96 | 0.51 | 0.05 | 1 | 0.98 | 2:50798296-50798951 | 37.35 | 1 |
| HB1B21B | 2 | 53111792 | 53112001 | 5.83 | 0.77 | 0.55 | 1 | 1 | 2:53111756-53112017 | 29.3 | 1 |
| HB1B21B | 2 | 65341376 | 65341973 | 11.63 | 0.51 | 0.05 | 1 | 0.98 | 2:65341352-65341989 | 35.19 | 1 |
| HB1B21B | 2 | 80333665 | 80334229 | 11.34 | 0.51 | 0.05 | 1 | 0.98 | 2:80333585-80334273 | 29.71 | 1 |
| HB1B21B | 2 | 81965026 | 81966025 | 1 | 0.31 | 0 | 0 | 0.73 | 2:81965001-81966072 | 26.96 | 1 |
| HB1B21B | 2 | 82574343 | 82575494 | 7.1 | 0.47 | 0.9 | 0.18 | 0 | 2:82574297-82575518 | 33.42 | 1 |
| HB1B21B | 2 | 92857041 | 92858193 | 4.98 | 0.75 | 0.53 | 1 | 0.98 | 2:92856996-92858219 | 35.34 | 1 |
| HB1B21B | 2 | 95480991 | 95481786 | 12.46 | 1 | 1 | 1 | 1 | 2:95480957-95481836 | 36.03 | 1 |
| HB1B21B | 2 | 124765101 | 124765718 | 17.62 | 0.52 | 0.05 | 1 | 1 | 2:124765081-124765718 | 35.05 | 1 |
| HB1B21B | 2 | 129140842 | 129141689 | 14.52 | 1 | 0.99 | 1 | 1 | 2:129140825-129141707 | 31.96 | 1 |
| HB1B21B | 2 | 131563803 | 131564046 | 5.88 | 0.51 | 0.05 | 1 | 0.98 | 2:131563757-131567794 | 32.15 | 1 |
| HB1B21B | 2 | 131567504 | 131567736 | 12.91 | 0.52 | 0.05 | 1 | 1 | 2:131563757-131567794 | 32.15 | 1 |
| HB1B21B | 3 | 19821858 | 19822972 | 5.56 | 0.95 | 0.91 | 1 | 0.98 | 3:19821802-19823007 | 24.72 | 1 |
| HB1B21B | 3 | 44140659 | 44141485 | 11.11 | 0.99 | 1 | 1 | 0.98 | 3:44140622-44141485 | 29.8 | 1 |
| HB1B21B | 3 | 49572136 | 49572911 | 11.81 | 1 | 1 | 1 | 1 | 3:49572068-49572931 | 35.8 | 1 |
| HB1B21B | 3 | 54250152 | 54250363 | 7.41 | 0.51 | 0.05 | 1 | 0.98 | 3:54250109-54254158 | 35.09 | 1 |
| HB1B21B | 3 | 54253888 | 54254157 | 6.56 | 0.52 | 0.05 | 1 | 1 | 3:54250109-54254158 | 35.09 | 1 |
| HB1B21B | 3 | 59937607 | 59938167 | 14.08 | 0.51 | 0.05 | 1 | 0.98 | 3:59937540-59938220 | 31.38 | 1 |
| HB1B21B | 3 | 70604588 | 70605355 | 16.42 | 0.99 | 1 | 1 | 0.98 | 3:70604555-70605387 | 22.73 | 1 |
| HB1B21B | 3 | 73561909 | 73563055 | 5.79 | 0.3 | 0 | 0 | 0.7 | 3:73561879-73563101 | 32.76 | 1 |
| HB1B21B | 3 | 83407710 | 83408470 | 24.98 | 1 | 1 | 1 | 1 | 3:83407619-83408514 | 27.84 | 0.82 |
| HB1B21B | 3 | 83776940 | 83777906 | 2.73 | 0.36 | 0 | 0 | 0.82 | 3:83776894-83777945 | 32.31 | 1 |
| HB1B21B | 3 | 84892879 | 84893097 | 7.97 | 0.51 | 0.05 | 1 | 0.98 | 3:84889113-84893120 | 31.12 | 1 |
| HB1B21B | 3 | 93697700 | 93698488 | 15.96 | 0.99 | 1 | 1 | 0.98 | 3:93697657-93698572 | 31.57 | 1 |
| HB1B21B | 3 | 96957554 | 96957780 | 6.36 | 0.51 | 0.05 | 1 | 0.98 | 3:96957515-96961612 | 31.69 | 1 |
| HB1B21B | 3 | 96961319 | 96961554 | 6.62 | 0.52 | 0.05 | 1 | 1 | 3:96957515-96961612 | 31.69 | 1 |
| HB1B21B | 3 | 102777268 | 102778110 | 28.91 | 1 | 1 | 1 | 1 | 3:102777226-102778143 | 30.31 | 1 |
| HB1B21B | 4 | 11124000 | 11124360 | 7.06 | 0.99 | 1 | 1 | 0.98 | 4:11123901-11124467 | 28.05 | 1 |
| HB1B21B | 4 | 20401588 | 20402427 | 13.45 | 1 | 1 | 1 | 1 | 4:20401588-20402427 | 33.43 | 0.99 |
| HB1B21B | 4 | 22222550 | 22223167 | 21.69 | 0.52 | 0.05 | 1 | 1 | 4:22222499-22223210 | 29.58 | 1 |
| HB1B21B | 4 | 26530911 | 26531159 | 8.68 | 0.51 | 0.05 | 1 | 0.98 | 4:26530893-26531990 | 30.09 | 1 |
| HB1B21B | 4 | 26531680 | 26531900 | 14.52 | 0.51 | 0.05 | 1 | 0.98 | 4:26530893-26531990 | 30.09 | 1 |
| HB1B21B | 4 | 27394572 | 27395191 | 14.55 | 0.52 | 0.05 | 1 | 1 | 4:27394572-27395212 | 33.72 | 1 |
| HB1B21B | 4 | 29234124 | 29234712 | 13.3 | 0.52 | 0.05 | 1 | 1 | 4:29234085-29234741 | 26.25 | 1 |
| HB1B21B | 4 | 30632767 | 30633319 | 9.93 | 0.51 | 0.05 | 1 | 0.98 | 4:30632716-30633375 | 30.91 | 1 |
| HB1B21B | 4 | 69679128 | 69679960 | 8.38 | 0.32 | 0 | 0 | 0.75 | 4:69679128-69679960 | 29.76 | 1 |
| HB1B21B | 4 | 70552013 | 70552638 | 18.18 | 0.99 | 1 | 1 | 0.98 | 4:70551829-70552669 | 23.92 | 1 |
| HB1B21B | 4 | 86220929 | 86221699 | 11.1 | 1 | 1 | 1 | 1 | 4:86220862-86221807 | 34.49 | 1 |
| HB1B21B | 5 | 2705106 | 2706244 | 6.54 | 0.86 | 0.99 | 1 | 0.68 | 5:2705052-2706284 | 30.96 | 1 |
| HB1B21B | 5 | 6164331 | 6165469 | 3.43 | 0.49 | 0.3 | 0.71 | 0.68 | 5:6164269-6165523 | 20.77 | 1 |
| HB1B21B | 5 | 31303919 | 31305049 | 2.91 | 0.11 | 0.23 | 0 | 0 | 5:31303866-31305068 | 35.9 | 1 |
| HB1B21B | 5 | 31525409 | 31526225 | 15.55 | 1 | 1 | 1 | 1 | 5:31525368-31526271 | 32.62 | 1 |
| HB1B21B | 5 | 31898714 | 31899817 | 31.62 | 0.82 | 1 | 1 | 0.57 | 5:31898626-31899883 | 39.06 | 1 |
| HB1B21B | 5 | 31998202 | 31999006 | 14.64 | 1 | 1 | 1 | 1 | 5:31998141-31999034 | 29.23 | 1 |
| HB1B21B | 5 | 40464052 | 40464868 | 13 | 0.99 | 0.99 | 1 | 0.99 | 5:40463978-40464915 | 31.83 | 1 |
| HB1B21B | 6 | 9811242 | 9811624 | 15.13 | 0.99 | 0.99 | 1 | 1 | 6:9811191-9816444 | 29.95 | 0.95 |
| HB1B21B | 6 | 9816018 | 9816427 | 14.23 | 0.99 | 0.99 | 1 | 0.98 | 6:9811191-9816444 | 29.95 | 0.95 |
| HB1B21B | 6 | 13724880 | 13725999 | 4.33 | 0.57 | 0.51 | 0.88 | 0.61 | 6:13724843-13726022 | 33.66 | 1 |
| HB1B21B | 7 | 17708105 | 17708703 | 27.28 | 0.48 | 0 | 1 | 0.98 | 7:17708068-17708753 | 32.44 | 1 |
| HB1B21B | 7 | 26115689 | 26116320 | 13.32 | 0.52 | 0.05 | 1 | 1 | 7:26115687-26116356 | 31.23 | 0.99 |
| HB1B21B | 8 | 9208412 | 9209122 | 3.27 | 1 | 1 | 1 | 0.99 | 8:9208394-9209273 | 16 | 1 |
| HB1B21B | 9 | 1710368 | 1710893 | 7.22 | 0.41 | 0.08 | 1 | 0.72 | 9:1710352-1710900 | 20.82 | 1 |
| HB1B21B | 9 | 5602599 | 5603206 | 29.13 | 0.51 | 0.05 | 1 | 0.98 | 9:5602574-5603257 | 30.45 | 1 |
| HB1B21B | 12 | 3240727 | 3240865 | 17.78 | 0.58 | 1 | 1 | 0.03 | 12:3240697-3240898 | 150.94 | 1 |
| HB1B21B | 13 | 1868321 | 1868923 | 18 | 0.51 | 0.05 | 1 | 0.99 | 13:1868296-1868957 | 28.04 | 1 |
| HB1B21B | 13 | 6484804 | 6485555 | 13.6 | 1 | 1 | 1 | 1 | 13:6484735-6485598 | 27.07 | 1 |
| HB1B21B | 17 | 180703 | 181502 | 12.99 | 0.99 | 0.99 | 1 | 0.98 | 17:180636-181570 | 30.01 | 1 |
| HB1B21B | AADN03009901.1 | 877 | 1125 | 8.63 | 0.51 | 0.05 | 1 | 0.99 | AADN03009901.1:851-1161 | 31.38 | 1 |
| HB1B21B | AADN03018735.1 | 299 | 455 | 6.77 | 0.91 | 0.99 | 1 | 0.81 | AADN03018735.1:281-1293 | 28.21 | 1 |
| HB1B21B | Z | 18808068 | 18808289 | 5.15 | 0.63 | 0.5 | 1 | 0.73 | Z:18807991-18809115 | 27.83 | 1 |
| HB1B21B | Z | 18808804 | 18809050 | 12.54 | 0.63 | 0.49 | 1 | 0.75 | Z:18807991-18809115 | 27.83 | 1 |
| HB1B21B | Z | 30996774 | 30996890 | 23.96 | 0.65 | 1 | 1 | 0.19 | Z:30996146-31000811 | 30.77 | 1 |
| HB1B21B | Z | 32081184 | 32081585 | 14.21 | 1 | 1 | 1 | 1 | Z:32081154-32086293 | 70.1 | 1 |
| HB1B21B | Z | 32085870 | 32086246 | 11.51 | 1 | 1 | 1 | 0.99 | Z:32081154-32086293 | 70.1 | 1 |
| HB1B21B | Z | 50872673 | 50873314 | 33.03 | 0.52 | 0.05 | 1 | 1 | Z:50872653-50873337 | 32.67 | 1 |
| HB1B21B | Z | 61304867 | 61305995 | 5.08 | 0.43 | 0 | 0.53 | 0.93 | Z:61304819-61306021 | 35.4 | 1 |
| HB1B21B | Z | 79945539 | 79946370 | 33.92 | 1 | 1 | 1 | 1 | Z:79945501-79946432 | 30.2 | 1 |
| JA1A17A | 1 | 32312250 | 32313090 | 25.65 | 0.99 | 1 | 1 | 0.98 | 1:32312218-32313185 | 28.95 | 1 |
| JA1A17A | 1 | 41318903 | 41319751 | 14.38 | 0.99 | 1 | 1 | 0.98 | 1:41318900-41319777 | 32.99 | 1 |
| JA1A17A | 1 | 140710345 | 140710822 | 12.57 | 1 | 1 | 1 | 0.99 | 1:140710345-140715423 | 41.69 | 1 |
| JA1A17A | 1 | 140715022 | 140715413 | 12.01 | 1 | 1 | 1 | 1 | 1:140710345-140715423 | 41.69 | 1 |
| JA1A17A | 1 | 140995796 | 140996700 | 9.82 | 0.97 | 1 | 1 | 0.93 | 1:140995796-140996700 | 22.3 | 1 |
| JA1A17A | 1 | 146731917 | 146732782 | 27.68 | 1 | 1 | 1 | 0.99 | 1:146731910-146732833 | 28.9 | 1 |
| JA1A17A | 1 | 148564568 | 148564822 | 7.23 | 0.51 | 0.05 | 1 | 0.98 | 1:148564562-148568615 | 31.35 | 0.97 |
| JA1A17A | 1 | 148568346 | 148568615 | 8.3 | 0.51 | 0.05 | 1 | 0.99 | 1:148564562-148568615 | 31.35 | 0.97 |
| JA1A17A | 1 | 163694756 | 163695363 | 9.45 | 0.52 | 0.05 | 1 | 1 | 1:163694748-163695372 | 17.25 | 1 |
| JA1A17A | 1 | 163906823 | 163907066 | 5.64 | 0.52 | 0.05 | 1 | 1 | 1:163906737-163910850 | 23.49 | 1 |
| JA1A17A | 1 | 180746907 | 180747112 | 7.11 | 0.51 | 0.05 | 1 | 0.99 | 1:180746896-180747970 | 24.21 | 1 |
| JA1A17A | 1 | 180747697 | 180747970 | 7.55 | 0.51 | 0.05 | 1 | 0.99 | 1:180746896-180747970 | 24.21 | 1 |
| JA1A17A | 1 | 182832884 | 182834049 | 12.27 | 0.99 | 0.99 | 1 | 0.98 | 1:182832847-182834082 | 30.14 | 1 |
| JA1A17A | 1 | 184347945 | 184348789 | 11.96 | 1 | 1 | 1 | 1 | 1:184347893-184348804 | 25.49 | 1 |
| JA1A17A | 1 | 185856868 | 185857484 | 18.14 | 0.51 | 0.05 | 1 | 0.99 | 1:185856831-185857511 | 31.19 | 1 |
| JA1A17A | 2 | 51801067 | 51801954 | 15.71 | 0.99 | 0.99 | 1 | 0.99 | 2:51801055-51801954 | 33.08 | 1 |
| JA1A17A | 2 | 53111789 | 53112000 | 6.63 | 0.84 | 0.69 | 1 | 0.98 | 2:53111756-53112017 | 27.06 | 1 |
| JA1A17A | 2 | 82574335 | 82575481 | 1.32 | 0.46 | 0.91 | 0 | 0 | 2:82574297-82575518 | 26.55 | 1 |
| JA1A17A | 2 | 87444008 | 87444641 | 12.81 | 0.51 | 0.05 | 1 | 0.98 | 2:87444008-87444641 | 35.5 | 1 |
| JA1A17A | 2 | 92857008 | 92858198 | 9.39 | 0.48 | 0.45 | 1 | 0.45 | 2:92856996-92858219 | 30.67 | 1 |
| JA1A17A | 2 | 95480957 | 95481781 | 11.63 | 1 | 1 | 1 | 0.99 | 2:95480957-95481836 | 36.7 | 1 |
| JA1A17A | 2 | 123095199 | 123095828 | 15.95 | 0.51 | 0.05 | 1 | 0.98 | 2:123095199-123095828 | 26.9 | 1 |
| JA1A17A | 2 | 123945004 | 123945201 | 7.6 | 0.46 | 0.05 | 1 | 0.87 | 2:123945004-123945201 | 43.32 | 1 |
| JA1A17A | 2 | 131563775 | 131564019 | 14.22 | 0.51 | 0.05 | 1 | 0.98 | 2:131563757-131567794 | 28.66 | 1 |
| JA1A17A | 2 | 131567510 | 131567790 | 17.39 | 0.52 | 0.05 | 1 | 1 | 2:131563757-131567794 | 28.66 | 1 |
| JA1A17A | 2 | 142113889 | 142114516 | 14.75 | 0.51 | 0.05 | 1 | 0.98 | 2:142113866-142114543 | 29.05 | 1 |
| JA1A17A | 3 | 19821850 | 19822982 | 6.72 | 0.99 | 0.99 | 1 | 0.98 | 3:19821802-19823007 | 22.93 | 1 |
| JA1A17A | 3 | 40908304 | 40908808 | 7.38 | 0.46 | 0.03 | 1 | 0.89 | 3:40908270-40908901 | 30.96 | 1 |
| JA1A17A | 3 | 44140631 | 44141459 | 9.9 | 1 | 1 | 1 | 1 | 3:44140622-44141485 | 30.79 | 1 |
| JA1A17A | 3 | 59937554 | 59938186 | 8.55 | 0.51 | 0.05 | 1 | 0.98 | 3:59937540-59938220 | 28.59 | 1 |
| JA1A17A | 3 | 73561879 | 73563061 | 1 | 0.19 | 0 | 0 | 0.44 | 3:73561879-73563101 | 27.13 | 1 |
| JA1A17A | 3 | 79282766 | 79283588 | 16.8 | 0.99 | 1 | 1 | 0.98 | 3:79282754-79283588 | 29.62 | 1 |
| JA1A17A | 3 | 83407747 | 83408422 | 7.82 | 1 | 1 | 1 | 1 | 3:83407619-83408514 | 27.56 | 1 |
| JA1A17A | 3 | 83776894 | 83777145 | 7.79 | 0.48 | 0.05 | 1 | 0.93 | 3:83776894-83777945 | 32.03 | 1 |
| JA1A17A | 3 | 83777720 | 83777917 | 9.69 | 0.51 | 0.05 | 1 | 0.98 | 3:83776894-83777945 | 32.03 | 1 |
| JA1A17A | 3 | 84550564 | 84551413 | 14.93 | 1 | 1 | 1 | 1 | 3:84550518-84551452 | 33.42 | 1 |
| JA1A17A | 3 | 93697695 | 93698546 | 29.29 | 0.99 | 1 | 1 | 0.98 | 3:93697657-93698572 | 27.19 | 1 |
| JA1A17A | 3 | 96957545 | 96957745 | 6.72 | 0.51 | 0.05 | 1 | 0.98 | 3:96957515-96961612 | 22.62 | 1 |
| JA1A17A | 4 | 11124010 | 11124401 | 12.41 | 0.99 | 1 | 1 | 0.98 | 4:11123901-11124467 | 23.99 | 1 |
| JA1A17A | 4 | 26530913 | 26531138 | 9.08 | 0.51 | 0.05 | 1 | 0.98 | 4:26530893-26531990 | 30.38 | 1 |
| JA1A17A | 4 | 26531680 | 26531913 | 12.05 | 0.51 | 0.05 | 1 | 0.98 | 4:26530893-26531990 | 30.38 | 1 |
| JA1A17A | 4 | 30595679 | 30596522 | 13.52 | 1 | 1 | 1 | 1 | 4:30595679-30596557 | 31.75 | 1 |
| JA1A17A | 4 | 30632771 | 30633359 | 9.92 | 0.51 | 0.05 | 1 | 0.98 | 4:30632716-30633375 | 26.31 | 1 |
| JA1A17A | 4 | 35886029 | 35886661 | 14.66 | 0.52 | 0.05 | 1 | 1 | 4:35886007-35886673 | 35.14 | 1 |
| JA1A17A | 4 | 86220868 | 86221788 | 28.16 | 1 | 1 | 1 | 1 | 4:86220862-86221807 | 28.14 | 0.91 |
| JA1A17A | 5 | 2705084 | 2706251 | 3.05 | 0.69 | 0.62 | 1 | 0.73 | 5:2705052-2706284 | 32.16 | 1 |
| JA1A17A | 5 | 31303903 | 31305053 | NA | NA | NA | NA | NA | 5:31303866-31305068 | 34.09 | 1 |
| JA1A17A | 5 | 31898722 | 31899870 | 12.34 | 0.61 | 0.6 | 1 | 0.56 | 5:31898626-31899883 | 29.47 | 1 |
| JA1A17A | 6 | 9811200 | 9811599 | 9.12 | 0.99 | 0.99 | 1 | 1 | 6:9811191-9816444 | 24.86 | 0.99 |
| JA1A17A | 6 | 9816096 | 9816368 | 7.97 | 0.99 | 0.99 | 1 | 0.98 | 6:9811191-9816444 | 24.86 | 0.99 |
| JA1A17A | 6 | 13724883 | 13725995 | NA | NA | NA | NA | NA | 6:13724843-13726022 | 31.97 | 1 |
| JA1A17A | 7 | 9022789 | 9023419 | 10.07 | 0.52 | 0.05 | 1 | 1 | 7:9022731-9023457 | 30.55 | 1 |
| JA1A17A | 7 | 15504227 | 15504882 | 18.52 | 0.52 | 0.05 | 1 | 1 | 7:15504223-15504887 | 34.22 | 1 |
| JA1A17A | 7 | 17708102 | 17708707 | 23.08 | 0.48 | 0.05 | 1 | 0.93 | 7:17708068-17708753 | 29.12 | 1 |
| JA1A17A | 8 | 9208438 | 9209088 | 4.94 | 1 | 1 | 1 | 0.99 | 8:9208394-9209273 | 19.77 | 1 |
| JA1A17A | 8 | 17696796 | 17697631 | 14.85 | 0.99 | 1 | 1 | 0.98 | 8:17696796-17697651 | 34.75 | 1 |
| JA1A17A | 11 | 11174321 | 11174690 | 6.35 | 0.99 | 0.99 | 1 | 0.98 | 11:11174286-11174756 | 31.87 | 1 |
| JA1A17A | 12 | 335263 | 335887 | 9.2 | 0.52 | 0.05 | 1 | 1 | 12:335263-335965 | 23.76 | 1 |
| JA1A17A | 12 | 3240727 | 3240871 | 38.61 | 0.59 | 1 | 1 | 0.06 | 12:3240697-3240898 | 183.72 | 1 |
| JA1A17A | 13 | 6484735 | 6485598 | 15.03 | 1 | 1 | 1 | 1 | 13:6484735-6485598 | 22.95 | 1 |
| JA1A17A | 15 | 1590631 | 1591272 | 18.34 | 0.52 | 0.05 | 1 | 1 | 15:1590630-1591272 | 27.48 | 1 |
| JA1A17A | 17 | 180680 | 181542 | 13.3 | 0.99 | 0.99 | 1 | 0.99 | 17:180636-181570 | 27.86 | 1 |
| JA1A17A | 20 | 7661907 | 7662749 | 16.36 | 1 | 1 | 1 | 1 | 20:7661897-7662786 | 32.38 | 1 |
| JA1A17A | AADN03009901.1 | 871 | 1139 | 10.08 | 0.51 | 0.05 | 1 | 0.99 | AADN03009901.1:851-1161 | 24.7 | 1 |
| JA1A17A | Z | 18808052 | 18809079 | NA | NA | NA | NA | NA | Z:18807991-18809115 | 30.59 | 1 |
| JA1A17A | Z | 32081196 | 32081578 | 13.26 | 1 | 1 | 1 | 1 | Z:32081154-32086293 | 39.69 | 1 |
| JA1A17A | Z | 32085904 | 32086277 | 13.29 | 0.99 | 1 | 1 | 0.98 | Z:32081154-32086293 | 39.69 | 1 |
| JA1A17A | Z | 50872704 | 50873279 | 18.65 | 0.52 | 0.05 | 1 | 1 | Z:50872653-50873337 | 37.71 | 1 |
| JA1A17A | Z | 61304849 | 61306008 | 6.37 | 0.91 | 0.86 | 1 | 0.97 | Z:61304819-61306021 | 32.41 | 1 |
| JA1A17A | Z | 78844863 | 78845991 | 35.01 | 0.65 | 1 | 0.18 | 0.31 | Z:78844863-78846057 | 30.59 | 1 |
| JA1A17A | Z | 79945521 | 79946397 | 18.21 | 1 | 1 | 1 | 1 | Z:79945501-79946432 | 25.96 | 1 |
| JA2A10B | 1 | 7414982 | 7415621 | 12.94 | 0.52 | 0.05 | 1 | 1 | 1:7414982-7415621 | 28.68 | 1 |
| JA2A10B | 1 | 32312264 | 32313082 | 27.59 | 0.99 | 1 | 1 | 0.98 | 1:32312218-32313185 | 28.7 | 1 |
| JA2A10B | 1 | 32508430 | 32508703 | 9.31 | 0.51 | 0.05 | 1 | 0.99 | 1:32508365-32508761 | 35.42 | 1 |
| JA2A10B | 1 | 41318910 | 41319747 | 17.02 | 0.99 | 1 | 1 | 0.98 | 1:41318900-41319777 | 30.61 | 1 |
| JA2A10B | 1 | 67550462 | 67551091 | 15.1 | 1 | 1 | 1 | 1 | 1:67550436-67551166 | 38.79 | 1 |
| JA2A10B | 1 | 84555430 | 84556039 | 14.55 | 0.51 | 0.05 | 1 | 0.98 | 1:84555430-84556039 | 32.23 | 1 |
| JA2A10B | 1 | 87425199 | 87425787 | 11.64 | 0.52 | 0.05 | 1 | 1 | 1:87425101-87425826 | 32.9 | 1 |
| JA2A10B | 1 | 101333523 | 101334378 | 15.1 | 1 | 1 | 1 | 1 | 1:101333491-101334404 | 30.28 | 1 |
| JA2A10B | 1 | 117166631 | 117167501 | 14.05 | 1 | 1 | 1 | 0.99 | 1:117166631-117167523 | 31.8 | 1 |
| JA2A10B | 1 | 131477317 | 131477931 | 8.92 | 0.52 | 0.05 | 1 | 1 | 1:131477317-131477931 | 30.93 | 1 |
| JA2A10B | 1 | 140710376 | 140710781 | 12.21 | 1 | 1 | 1 | 0.99 | 1:140710345-140715423 | 45.46 | 1 |
| JA2A10B | 1 | 140714992 | 140715410 | 12.2 | 1 | 1 | 1 | 1 | 1:140710345-140715423 | 45.46 | 1 |
| JA2A10B | 1 | 146731927 | 146732833 | 10.23 | 0.99 | 1 | 1 | 0.98 | 1:146731910-146732833 | 26.45 | 1 |
| JA2A10B | 1 | 148564586 | 148564829 | 7.32 | 0.52 | 0.05 | 1 | 1 | 1:148564562-148568615 | 44.68 | 1 |
| JA2A10B | 1 | 148568345 | 148568612 | 16.53 | 0.51 | 0.05 | 1 | 0.98 | 1:148564562-148568615 | 44.68 | 1 |
| JA2A10B | 1 | 149162774 | 149163200 | 5.4 | 1 | 1 | 1 | 0.99 | 1:149162760-149166227 | 38.9 | 1 |
| JA2A10B | 1 | 149165129 | 149166217 | 3.17 | 0.74 | 0.49 | 1 | 1 | 1:149162760-149166227 | 38.9 | 1 |
| JA2A10B | 1 | 160455230 | 160455474 | 7.97 | 0.51 | 0.05 | 1 | 0.98 | 1:160455218-160455708 | 25.27 | 1 |
| JA2A10B | 1 | 160463946 | 160464245 | 17.07 | 0.52 | 0.05 | 1 | 1 | 1:160463921-160464245 | 37 | 1 |
| JA2A10B | 1 | 163501541 | 163501756 | 11.01 | 0.52 | 0.05 | 1 | 1 | 1:163501535-163501830 | 29.37 | 1 |
| JA2A10B | 1 | 182832889 | 182834039 | 18.93 | 0.92 | 0.99 | 1 | 0.82 | 1:182832847-182834082 | 29.49 | 1 |
| JA2A10B | 1 | 185856873 | 185857496 | 9.07 | 0.51 | 0.05 | 1 | 0.98 | 1:185856831-185857511 | 32.88 | 1 |
| JA2A10B | 2 | 10512610 | 10512863 | 4.95 | 0.51 | 0.05 | 1 | 0.99 | 2:10512425-10512887 | 26.7 | 1 |
| JA2A10B | 2 | 20971546 | 20972437 | 18.7 | 0.62 | 0.27 | 1 | 0.98 | 2:20971534-20972437 | 37.69 | 1 |
| JA2A10B | 2 | 51801070 | 51801931 | 31.18 | 0.99 | 0.99 | 1 | 0.99 | 2:51801055-51801954 | 33.49 | 1 |
| JA2A10B | 2 | 53111756 | 53112001 | 10.01 | 0.91 | 0.84 | 1 | 0.98 | 2:53111756-53112017 | 24.88 | 1 |
| JA2A10B | 2 | 81965019 | 81965255 | 6.86 | 0.52 | 0.05 | 1 | 0.99 | 2:81965001-81966072 | 27.15 | 1 |
| JA2A10B | 2 | 81965802 | 81966064 | 6.11 | 0.51 | 0.05 | 1 | 0.99 | 2:81965001-81966072 | 27.15 | 1 |
| JA2A10B | 2 | 82574335 | 82575507 | 2.43 | 0.79 | 0.93 | 0.12 | 0.73 | 2:82574297-82575518 | 29.88 | 1 |
| JA2A10B | 2 | 92058477 | 92059335 | 14.14 | 1 | 1 | 1 | 1 | 2:92058477-92059335 | 33.95 | 1 |
| JA2A10B | 2 | 92857054 | 92858211 | NA | NA | NA | NA | NA | 2:92856996-92858219 | 34.32 | 1 |
| JA2A10B | 2 | 131563774 | 131564029 | 15.23 | 0.51 | 0.05 | 1 | 0.99 | 2:131563757-131567794 | 33.6 | 1 |
| JA2A10B | 2 | 131567513 | 131567768 | 13.09 | 0.52 | 0.05 | 1 | 1 | 2:131563757-131567794 | 33.6 | 1 |
| JA2A10B | 3 | 19821835 | 19822998 | 12.56 | 0.99 | 0.99 | 1 | 0.98 | 3:19821802-19823007 | 27.11 | 1 |
| JA2A10B | 3 | 40908278 | 40908901 | 11.13 | 0.47 | 0 | 0.76 | 0.98 | 3:40908270-40908901 | 29.59 | 1 |
| JA2A10B | 3 | 49572075 | 49572915 | 16.94 | 1 | 1 | 1 | 1 | 3:49572068-49572931 | 35.36 | 1 |
| JA2A10B | 3 | 59937561 | 59938176 | 13.26 | 0.51 | 0.05 | 1 | 0.98 | 3:59937540-59938220 | 30.48 | 1 |
| JA2A10B | 3 | 60621854 | 60622477 | 11.95 | 0.52 | 0.05 | 1 | 0.99 | 3:60621854-60622499 | 30.33 | 1 |
| JA2A10B | 3 | 70604604 | 70605364 | 10.69 | 0.99 | 1 | 1 | 0.98 | 3:70604555-70605387 | 20.99 | 1 |
| JA2A10B | 3 | 73561884 | 73563074 | 2.53 | 0.77 | 0.55 | 1 | 0.99 | 3:73561879-73563101 | 33.32 | 1 |
| JA2A10B | 3 | 83776925 | 83777147 | 13.38 | 0.5 | 0.05 | 1 | 0.97 | 3:83776894-83777945 | 35.08 | 1 |
| JA2A10B | 3 | 83777716 | 83777913 | 11.9 | 0.51 | 0.05 | 1 | 0.98 | 3:83776894-83777945 | 35.08 | 1 |
| JA2A10B | 3 | 84550520 | 84551425 | 13.3 | 1 | 1 | 1 | 1 | 3:84550518-84551452 | 32.32 | 1 |
| JA2A10B | 3 | 93697703 | 93698544 | 32.53 | 1 | 1 | 1 | 0.99 | 3:93697657-93698572 | 29.45 | 1 |
| JA2A10B | 4 | 11124018 | 11124407 | 13.73 | 0.99 | 1 | 1 | 0.98 | 4:11123901-11124467 | 26.73 | 1 |
| JA2A10B | 4 | 16267586 | 16268257 | 29.04 | 0.51 | 0.05 | 1 | 0.99 | 4:16267586-16268257 | 28.29 | 1 |
| JA2A10B | 4 | 26530933 | 26531188 | 12.74 | 0.51 | 0.05 | 1 | 0.98 | 4:26530893-26531990 | 28.05 | 1 |
| JA2A10B | 4 | 26531700 | 26531946 | 10.5 | 0.51 | 0.05 | 1 | 0.98 | 4:26530893-26531990 | 28.05 | 1 |
| JA2A10B | 4 | 30632735 | 30633344 | 23.03 | 0.51 | 0.05 | 1 | 0.98 | 4:30632716-30633375 | 30.72 | 1 |
| JA2A10B | 4 | 62460729 | 62461331 | 10.23 | 0.51 | 0.04 | 1 | 1 | 4:62460729-62461331 | 29.57 | 1 |
| JA2A10B | 4 | 78901630 | 78902494 | 16.36 | 1 | 1 | 1 | 1 | 4:78901609-78902511 | 30.76 | 1 |
| JA2A10B | 4 | 86220883 | 86221805 | 11.59 | 1 | 1 | 1 | 1 | 4:86220862-86221807 | 27.52 | 1 |
| JA2A10B | 5 | 2705086 | 2706250 | 6.21 | 0.66 | 0.64 | 1 | 0.65 | 5:2705052-2706284 | 30.37 | 1 |
| JA2A10B | 5 | 31303906 | 31305035 | NA | NA | NA | NA | NA | 5:31303866-31305068 | 34.59 | 1 |
| JA2A10B | 5 | 31898704 | 31899883 | 18.78 | 0.48 | 0.37 | 1 | 0.55 | 5:31898626-31899883 | 36.5 | 1 |
| JA2A10B | 5 | 31998154 | 31999006 | 30.64 | 1 | 1 | 1 | 1 | 5:31998141-31999034 | 30.77 | 1 |
| JA2A10B | 6 | 5291837 | 5292712 | 14.87 | 1 | 1 | 1 | 1 | 6:5291837-5292712 | 32.76 | 1 |
| JA2A10B | 6 | 9811212 | 9811554 | 14.55 | 0.99 | 0.99 | 1 | 1 | 6:9811191-9816444 | 32.03 | 1 |
| JA2A10B | 6 | 9816018 | 9816444 | 15.7 | 0.99 | 0.99 | 1 | 0.98 | 6:9811191-9816444 | 32.03 | 1 |
| JA2A10B | 6 | 13724864 | 13726016 | NA | NA | NA | NA | NA | 6:13724843-13726022 | 31.22 | 1 |
| JA2A10B | 7 | 9022790 | 9023433 | 15.78 | 0.52 | 0.05 | 1 | 1 | 7:9022731-9023457 | 33.04 | 1 |
| JA2A10B | 7 | 15504254 | 15504784 | 5.14 | 0.51 | 0.05 | 1 | 0.98 | 7:15504223-15504887 | 32.32 | 1 |
| JA2A10B | 7 | 17708086 | 17708654 | 22.38 | 0.5 | 0.05 | 1 | 0.96 | 7:17708068-17708753 | 32.21 | 1 |
| JA2A10B | 7 | 26115687 | 26116356 | 10.49 | 0.52 | 0.05 | 1 | 1 | 7:26115687-26116356 | 29.07 | 0.99 |
| JA2A10B | 8 | 9208405 | 9209240 | 9.05 | 1 | 1 | 1 | 0.99 | 8:9208394-9209273 | 21.84 | 1 |
| JA2A10B | 8 | 11644655 | 11645224 | 10.74 | 0.52 | 0.05 | 1 | 1 | 8:11644653-11645314 | 29.31 | 1 |
| JA2A10B | 8 | 23631239 | 23632111 | 13.57 | 0.99 | 1 | 1 | 0.98 | 8:23631239-23632111 | 29.12 | 1 |
| JA2A10B | 12 | 3240730 | 3240863 | 16.62 | 0.59 | 1 | 1 | 0.05 | 12:3240697-3240898 | 191.2 | 1 |
| JA2A10B | 12 | 17400816 | 17401450 | 11.34 | 0.52 | 0.05 | 1 | 1 | 12:17400816-17401479 | 31.34 | 1 |
| JA2A10B | JH376323.1 | 17975 | 18120 | 8.3 | 0.27 | 0 | 0 | 0.62 | JH376323.1:17955-18124 | 108.57 | 1 |
| JA2A10B | Z | 18808050 | 18808286 | 7.41 | 0.63 | 0.49 | 1 | 0.73 | Z:18807991-18809115 | 30.38 | 1 |
| JA2A10B | Z | 18808837 | 18809081 | 10.77 | 0.63 | 0.49 | 1 | 0.73 | Z:18807991-18809115 | 30.38 | 1 |
| JA2A10B | Z | 32081173 | 32081589 | 16.23 | 1 | 1 | 1 | 1 | Z:32081154-32086293 | 48.38 | 1 |
| JA2A10B | Z | 32085905 | 32086293 | 11.68 | 0.99 | 1 | 1 | 0.98 | Z:32081154-32086293 | 48.38 | 1 |
| JA2A10B | Z | 61304857 | 61305991 | 6.58 | 0.68 | 0.42 | 0.88 | 0.96 | Z:61304819-61306021 | 36.39 | 1 |
| JA2A10B | Z | 78844890 | 78846020 | 19.88 | 0.82 | 0.99 | 0.94 | 0.6 | Z:78844863-78846057 | 22.15 | 0.99 |
| JA2A10B | Z | 79945585 | 79946376 | 13.36 | 1 | 1 | 1 | 1 | Z:79945501-79946432 | 32.93 | 1 |
| JB2A04B.2 | 1 | 32312251 | 32313022 | 10.39 | 0.99 | 1 | 1 | 0.98 | 1:32312218-32313185 | 22.3 | 1 |
| JB2A04B.2 | 1 | 32508445 | 32508659 | 4.8 | 0.51 | 0.05 | 1 | 0.99 | 1:32508365-32508761 | 25.77 | 1 |
| JB2A04B.2 | 1 | 41318924 | 41319755 | 12.29 | 0.99 | 1 | 1 | 0.98 | 1:41318900-41319777 | 24.81 | 1 |
| JB2A04B.2 | 1 | 70454724 | 70455314 | 9.7 | 0.52 | 0.05 | 1 | 1 | 1:70454672-70455338 | 23.75 | 1 |
| JB2A04B.2 | 1 | 95854651 | 95855224 | 14.71 | 0.51 | 0.05 | 1 | 0.99 | 1:95854597-95855224 | 29.75 | 1 |
| JB2A04B.2 | 1 | 96156954 | 96157346 | 5.99 | 0.99 | 1 | 1 | 0.98 | 1:96156897-96157369 | 29.9 | 1 |
| JB2A04B.2 | 1 | 99050593 | 99051142 | 14.19 | 0.52 | 0.05 | 1 | 1 | 1:99050515-99051202 | 25.48 | 1 |
| JB2A04B.2 | 1 | 99136332 | 99136917 | 14.78 | 0.46 | 0.05 | 1 | 0.87 | 1:99136310-99136953 | 21.61 | 1 |
| JB2A04B.2 | 1 | 101333527 | 101334369 | 11.25 | 0.99 | 1 | 1 | 0.98 | 1:101333491-101334404 | 22.7 | 1 |
| JB2A04B.2 | 1 | 126794631 | 126795484 | 13.26 | 1 | 1 | 1 | 1 | 1:126794615-126795484 | 27.89 | 1 |
| JB2A04B.2 | 1 | 140710374 | 140710804 | 9.32 | 1 | 1 | 1 | 0.99 | 1:140710345-140715423 | 30.29 | 1 |
| JB2A04B.2 | 1 | 140715004 | 140715384 | 13.09 | 1 | 1 | 1 | 1 | 1:140710345-140715423 | 30.29 | 1 |
| JB2A04B.2 | 1 | 146731947 | 146732750 | 7.99 | 0.99 | 1 | 1 | 0.98 | 1:146731910-146732833 | 25.16 | 1 |
| JB2A04B.2 | 1 | 149162820 | 149163212 | 6.1 | 0.98 | 1 | 1 | 0.96 | 1:149162760-149166227 | 29.29 | 1 |
| JB2A04B.2 | 1 | 149165146 | 149166180 | 5.46 | 0.46 | 0.35 | 0 | 0.65 | 1:149162760-149166227 | 29.29 | 1 |
| JB2A04B.2 | 1 | 160455218 | 160455478 | 5.13 | 0.51 | 0.05 | 1 | 0.98 | 1:160455218-160455708 | 17.78 | 1 |
| JB2A04B.2 | 1 | 160463962 | 160464210 | 8.43 | 0.52 | 0.05 | 1 | 1 | 1:160463921-160464245 | 26.98 | 1 |
| JB2A04B.2 | 1 | 163501569 | 163501730 | 5.91 | 0.52 | 0.05 | 1 | 1 | 1:163501535-163501830 | 22.33 | 1 |
| JB2A04B.2 | 1 | 163694818 | 163695334 | 8.66 | 0.52 | 0.05 | 1 | 1 | 1:163694748-163695372 | 18.33 | 1 |
| JB2A04B.2 | 1 | 180746924 | 180747132 | 10.52 | 0.51 | 0.05 | 1 | 0.99 | 1:180746896-180747970 | 23.93 | 1 |
| JB2A04B.2 | 1 | 180747688 | 180747914 | 9.73 | 0.52 | 0.05 | 1 | 1 | 1:180746896-180747970 | 23.93 | 1 |
| JB2A04B.2 | 1 | 182832887 | 182834032 | 9.76 | 0.99 | 0.99 | 1 | 0.98 | 1:182832847-182834082 | 20.89 | 1 |
| JB2A04B.2 | 1 | 187340689 | 187340886 | 4.33 | 0.51 | 0.05 | 1 | 0.99 | 1:187340668-187342356 | 18.23 | 1 |
| JB2A04B.2 | 1 | 187341956 | 187342343 | 6.51 | 0.51 | 0.05 | 1 | 0.98 | 1:187340668-187342356 | 18.23 | 1 |
| JB2A04B.2 | 2 | 51801109 | 51801932 | 16.7 | 0.99 | 0.99 | 1 | 0.99 | 2:51801055-51801954 | 24.6 | 1 |
| JB2A04B.2 | 2 | 53111795 | 53112001 | 8.65 | 0.85 | 0.71 | 1 | 0.98 | 2:53111756-53112017 | 23.18 | 1 |
| JB2A04B.2 | 2 | 82574322 | 82575490 | 4.86 | 0.79 | 0.92 | 0.12 | 0.72 | 2:82574297-82575518 | 24.15 | 1 |
| JB2A04B.2 | 2 | 92857033 | 92858123 | 7.02 | 0.49 | 0.45 | 1 | 0.48 | 2:92856996-92858219 | 24.2 | 1 |
| JB2A04B.2 | 2 | 94577747 | 94577897 | 6.01 | 0.39 | 0.05 | 1 | 0.71 | 2:94577735-94578007 | 24.71 | 1 |
| JB2A04B.2 | 2 | 95480992 | 95481797 | 14 | 1 | 1 | 1 | 1 | 2:95480957-95481836 | 29.31 | 1 |
| JB2A04B.2 | 2 | 131563789 | 131564033 | 9.48 | 0.51 | 0.05 | 1 | 0.98 | 2:131563757-131567794 | 25.4 | 1 |
| JB2A04B.2 | 2 | 131567498 | 131567752 | 4.87 | 0.52 | 0.05 | 1 | 1 | 2:131563757-131567794 | 25.4 | 1 |
| JB2A04B.2 | 3 | 19821892 | 19822973 | 12.35 | 0.78 | 0.92 | 0 | 0.73 | 3:19821802-19823007 | 20.24 | 1 |
| JB2A04B.2 | 3 | 34783119 | 34783365 | 13.88 | 0.51 | 0.05 | 1 | 0.98 | 3:34782953-34783379 | 35.66 | 1 |
| JB2A04B.2 | 3 | 59937569 | 59938188 | 15.45 | 0.51 | 0.05 | 1 | 0.98 | 3:59937540-59938220 | 24.32 | 1 |
| JB2A04B.2 | 3 | 70604595 | 70605339 | 9.28 | 0.99 | 1 | 1 | 0.98 | 3:70604555-70605387 | 19.59 | 0.97 |
| JB2A04B.2 | 3 | 73561952 | 73563056 | 1 | 0.31 | 0.62 | 0 | 0 | 3:73561879-73563101 | 23.15 | 1 |
| JB2A04B.2 | 3 | 83776928 | 83777140 | 10.14 | 0.51 | 0.05 | 1 | 0.98 | 3:83776894-83777945 | 28.36 | 1 |
| JB2A04B.2 | 3 | 83777683 | 83777904 | 9.07 | 0.46 | 0.05 | 1 | 0.88 | 3:83776894-83777945 | 28.36 | 1 |
| JB2A04B.2 | 3 | 84550542 | 84551403 | 13.7 | 1 | 1 | 1 | 1 | 3:84550518-84551452 | 29.89 | 1 |
| JB2A04B.2 | 3 | 93697707 | 93698497 | 21.16 | 1 | 1 | 1 | 1 | 3:93697657-93698572 | 19.2 | 1 |
| JB2A04B.2 | 4 | 11124013 | 11124383 | 9.1 | 0.99 | 1 | 1 | 0.98 | 4:11123901-11124467 | 22.84 | 1 |
| JB2A04B.2 | 4 | 26530926 | 26531164 | 10.05 | 0.51 | 0.05 | 1 | 0.98 | 4:26530893-26531990 | 23.32 | 1 |
| JB2A04B.2 | 4 | 26531699 | 26531913 | 8.29 | 0.51 | 0.05 | 1 | 0.98 | 4:26530893-26531990 | 23.32 | 1 |
| JB2A04B.2 | 4 | 30632752 | 30633339 | 24.92 | 0.51 | 0.05 | 1 | 0.98 | 4:30632716-30633375 | 24.43 | 1 |
| JB2A04B.2 | 4 | 78901641 | 78902485 | 23.8 | 1 | 1 | 1 | 1 | 4:78901609-78902511 | 25.04 | 1 |
| JB2A04B.2 | 5 | 2705110 | 2706181 | 5.87 | 0.67 | 0.6 | 1 | 0.71 | 5:2705052-2706284 | 23.05 | 1 |
| JB2A04B.2 | 5 | 6164297 | 6165482 | 1.86 | 0.27 | 0 | 0 | 0.62 | 5:6164269-6165523 | 21.82 | 1 |
| JB2A04B.2 | 5 | 31303912 | 31305035 | NA | NA | NA | NA | NA | 5:31303866-31305068 | 25.99 | 1 |
| JB2A04B.2 | 5 | 31898710 | 31899849 | 9.12 | 0.76 | 0.9 | 1 | 0.57 | 5:31898626-31899883 | 29.92 | 1 |
| JB2A04B.2 | 5 | 31998141 | 31998991 | 10.68 | 1 | 1 | 1 | 1 | 5:31998141-31999034 | 28.51 | 1 |
| JB2A04B.2 | 6 | 9811219 | 9811596 | 13.12 | 0.99 | 0.99 | 1 | 1 | 6:9811191-9816444 | 23.45 | 0.99 |
| JB2A04B.2 | 6 | 9816033 | 9816420 | 8.16 | 0.99 | 0.99 | 1 | 0.99 | 6:9811191-9816444 | 23.45 | 0.99 |
| JB2A04B.2 | 6 | 13724897 | 13725999 | NA | NA | NA | NA | NA | 6:13724843-13726022 | 28.61 | 1 |
| JB2A04B.2 | 7 | 9022781 | 9023403 | 7.75 | 0.52 | 0.05 | 1 | 1 | 7:9022731-9023457 | 23.86 | 1 |
| JB2A04B.2 | 7 | 15504249 | 15504832 | 6.45 | 0.52 | 0.05 | 1 | 1 | 7:15504223-15504887 | 21.51 | 1 |
| JB2A04B.2 | 7 | 17708099 | 17708705 | 17.78 | 0.5 | 0.04 | 1 | 0.97 | 7:17708068-17708753 | 27.63 | 1 |
| JB2A04B.2 | 8 | 9208438 | 9209158 | 2.63 | 0.82 | 1 | 1 | 0.57 | 8:9208394-9209273 | 11.53 | 0.97 |
| JB2A04B.2 | 8 | 11644677 | 11645263 | 9.9 | 0.51 | 0.05 | 1 | 0.99 | 8:11644653-11645314 | 18.44 | 1 |
| JB2A04B.2 | 9 | 5602574 | 5603215 | 10.67 | 0.51 | 0.05 | 1 | 0.99 | 9:5602574-5603257 | 26.76 | 1 |
| JB2A04B.2 | 12 | 3240729 | 3240870 | 22.91 | 0.5 | 0.99 | 0 | 0 | 12:3240697-3240898 | 80.62 | 1 |
| JB2A04B.2 | 13 | 2970519 | 2971350 | 8.63 | 1 | 1 | 1 | 1 | 13:2970495-2971368 | 24.77 | 1 |
| JB2A04B.2 | 14 | 7965914 | 7966732 | 10.18 | 0.99 | 1 | 1 | 0.98 | 14:7965871-7966753 | 26.43 | 1 |
| JB2A04B.2 | 17 | 180669 | 181491 | 14.67 | 0.99 | 0.99 | 1 | 0.98 | 17:180636-181570 | 24.64 | 1 |
| JB2A04B.2 | Z | 18808095 | 18808297 | 8.8 | 0.51 | 0.27 | 1 | 0.73 | Z:18807991-18809115 | 24.97 | 1 |
| JB2A04B.2 | Z | 18808813 | 18809070 | 7.75 | 0.63 | 0.49 | 1 | 0.73 | Z:18807991-18809115 | 24.97 | 1 |
| JB2A04B.2 | Z | 32081170 | 32081566 | 10.4 | 1 | 1 | 1 | 1 | Z:32081154-32086293 | 30.05 | 1 |
| JB2A04B.2 | Z | 32085864 | 32086268 | 8.42 | 0.99 | 1 | 1 | 0.98 | Z:32081154-32086293 | 30.05 | 1 |
| JB2A04B.2 | Z | 50801195 | 50801772 | 14.89 | 0.52 | 0.05 | 1 | 1 | Z:50801143-50801818 | 19.16 | 1 |
| JB2A04B.2 | Z | 61304848 | 61306005 | 9.29 | 0.44 | 0 | 0.53 | 0.94 | Z:61304819-61306021 | 24.04 | 1 |
| JB2A04B.2 | Z | 78844879 | 78846057 | 6.53 | 0.79 | 0.95 | 1 | 0.59 | Z:78844863-78846057 | 21.07 | 1 |
| JB2A04B.2 | Z | 79945566 | 79946386 | 17.46 | 1 | 1 | 1 | 1 | Z:79945501-79946432 | 20.16 | 1 |
| JB1A25B | 1 | 31728941 | 31729551 | 12.45 | 0.52 | 0.05 | 1 | 1 | 1:31728916-31729579 | 29.66 | 1 |
| JB1A25B | 1 | 32312227 | 32313105 | 31.29 | 0.99 | 1 | 1 | 0.98 | 1:32312218-32313185 | 26.08 | 1 |
| JB1A25B | 1 | 44606699 | 44607552 | 10.71 | 1 | 1 | 1 | 1 | 1:44606699-44607552 | 29.25 | 1 |
| JB1A25B | 1 | 99136342 | 99136953 | 11.86 | 0.46 | 0.05 | 1 | 0.87 | 1:99136310-99136953 | 31.1 | 1 |
| JB1A25B | 1 | 113945462 | 113946354 | 16.96 | 0.36 | 0 | 0 | 0.83 | 1:113945460-113946362 | 30.9 | 1 |
| JB1A25B | 1 | 140710423 | 140710802 | 14.85 | 1 | 1 | 1 | 0.99 | 1:140710345-140715423 | 45.08 | 1 |
| JB1A25B | 1 | 140715001 | 140715408 | 13.41 | 1 | 1 | 1 | 1 | 1:140710345-140715423 | 45.08 | 1 |
| JB1A25B | 1 | 141097693 | 141098509 | 10.02 | 0.51 | 0.05 | 1 | 0.99 | 1:141097673-141098554 | 35.82 | 1 |
| JB1A25B | 1 | 146731925 | 146732815 | 16.95 | 0.99 | 1 | 1 | 0.98 | 1:146731910-146732833 | 29.79 | 1 |
| JB1A25B | 1 | 146761537 | 146762173 | 19.64 | 0.52 | 0.05 | 1 | 1 | 1:146761527-146762190 | 32.66 | 1 |
| JB1A25B | 1 | 151407118 | 151407227 | 5.08 | 0.33 | 0.23 | 0 | 0.5 | 1:151407118-151410307 | 16.92 | 0.91 |
| JB1A25B | 1 | 157698914 | 157699764 | 13.82 | 1 | 1 | 1 | 1 | 1:157698899-157699774 | 30.1 | 1 |
| JB1A25B | 1 | 160455239 | 160455469 | 5.96 | 0.51 | 0.05 | 1 | 0.98 | 1:160455218-160455708 | 20.88 | 1 |
| JB1A25B | 1 | 160463974 | 160464207 | 8.59 | 0.52 | 0.05 | 1 | 1 | 1:160463921-160464245 | 27.46 | 1 |
| JB1A25B | 1 | 163501540 | 163501765 | 11.33 | 0.52 | 0.05 | 1 | 1 | 1:163501535-163501830 | 25.92 | 1 |
| JB1A25B | 1 | 163694748 | 163695372 | 26.59 | 0.52 | 0.05 | 1 | 1 | 1:163694748-163695372 | 19.71 | 1 |
| JB1A25B | 1 | 180746938 | 180747122 | 4.89 | 0.51 | 0.05 | 1 | 0.99 | 1:180746896-180747970 | 26.88 | 1 |
| JB1A25B | 1 | 180747716 | 180747947 | 4.46 | 0.51 | 0.05 | 1 | 0.99 | 1:180746896-180747970 | 26.88 | 1 |
| JB1A25B | 1 | 182832884 | 182834061 | 7.84 | 0.92 | 0.99 | 1 | 0.82 | 1:182832847-182834082 | 24.81 | 1 |
| JB1A25B | 2 | 10512610 | 10512864 | 8.41 | 0.51 | 0.05 | 1 | 0.99 | 2:10512425-10512887 | 33.9 | 1 |
| JB1A25B | 2 | 51801071 | 51801937 | 11.43 | 0.99 | 0.99 | 1 | 0.99 | 2:51801055-51801954 | 33.88 | 1 |
| JB1A25B | 2 | 53111805 | 53112007 | 5.74 | 0.87 | 0.75 | 1 | 0.98 | 2:53111756-53112017 | 26.17 | 1 |
| JB1A25B | 2 | 82574333 | 82575495 | 8.62 | 0.51 | 0.99 | 0.12 | 0 | 2:82574297-82575518 | 31.03 | 1 |
| JB1A25B | 2 | 92070863 | 92071755 | 12.23 | 1 | 1 | 1 | 1 | 2:92070857-92071755 | 26.75 | 1 |
| JB1A25B | 2 | 92857023 | 92858184 | 1 | 0.31 | 0.49 | 1 | 0.01 | 2:92856996-92858219 | 27.48 | 1 |
| JB1A25B | 2 | 131563761 | 131564032 | 20.65 | 0.51 | 0.05 | 1 | 0.99 | 2:131563757-131567794 | 38.7 | 1 |
| JB1A25B | 2 | 131567527 | 131567794 | 11.76 | 0.52 | 0.05 | 1 | 1 | 2:131563757-131567794 | 38.7 | 1 |
| JB1A25B | 3 | 19821831 | 19822965 | 7.27 | 0.99 | 0.99 | 1 | 0.98 | 3:19821802-19823007 | 23.11 | 1 |
| JB1A25B | 3 | 40908270 | 40908891 | 15.01 | 0.47 | 0 | 0.94 | 0.97 | 3:40908270-40908901 | 30.54 | 1 |
| JB1A25B | 3 | 73561904 | 73563074 | 3.04 | 0.7 | 0.7 | 1 | 0.67 | 3:73561879-73563101 | 26.12 | 1 |
| JB1A25B | 3 | 83776908 | 83777125 | 13.72 | 0.5 | 0.05 | 1 | 0.97 | 3:83776894-83777945 | 36.48 | 1 |
| JB1A25B | 3 | 83777693 | 83777941 | 19.78 | 0.48 | 0.05 | 1 | 0.92 | 3:83776894-83777945 | 36.48 | 1 |
| JB1A25B | 3 | 84550525 | 84551408 | 31.1 | 1 | 1 | 1 | 1 | 3:84550518-84551452 | 28.01 | 1 |
| JB1A25B | 3 | 93697688 | 93698572 | 33.36 | 1 | 1 | 1 | 1 | 3:93697657-93698572 | 31.94 | 1 |
| JB1A25B | 3 | 95722787 | 95723662 | 13.84 | 0.99 | 0.99 | 1 | 0.99 | 3:95722787-95723662 | 33.05 | 1 |
| JB1A25B | 3 | 102777226 | 102778117 | 13.31 | 1 | 1 | 1 | 1 | 3:102777226-102778143 | 28.08 | 1 |
| JB1A25B | 4 | 16267586 | 16268207 | 8.99 | 0.51 | 0.05 | 1 | 0.98 | 4:16267586-16268257 | 27.6 | 1 |
| JB1A25B | 4 | 26530901 | 26531141 | 9.09 | 0.51 | 0.05 | 1 | 0.98 | 4:26530893-26531990 | 26.71 | 1 |
| JB1A25B | 4 | 26531695 | 26531920 | 8.11 | 0.51 | 0.05 | 1 | 0.98 | 4:26530893-26531990 | 26.71 | 1 |
| JB1A25B | 4 | 29234088 | 29234741 | 15.78 | 0.52 | 0.05 | 1 | 1 | 4:29234085-29234741 | 26.24 | 1 |
| JB1A25B | 4 | 30632716 | 30633375 | 29.75 | 0.51 | 0.05 | 1 | 0.98 | 4:30632716-30633375 | 29.36 | 1 |
| JB1A25B | 4 | 35886007 | 35886673 | 30.3 | 0.52 | 0.05 | 1 | 1 | 4:35886007-35886673 | 31.34 | 1 |
| JB1A25B | 4 | 48096968 | 48097609 | 8.8 | 0.52 | 0.05 | 1 | 1 | 4:48096947-48097615 | 30.71 | 1 |
| JB1A25B | 4 | 86220888 | 86221799 | 16.14 | 1 | 1 | 1 | 1 | 4:86220862-86221807 | 31.46 | 1 |
| JB1A25B | 5 | 2705114 | 2706269 | 5.94 | 0.62 | 0.64 | 1 | 0.53 | 5:2705052-2706284 | 26.01 | 1 |
| JB1A25B | 5 | 31303901 | 31305058 | NA | NA | NA | NA | NA | 5:31303866-31305068 | 34.32 | 1 |
| JB1A25B | 5 | 31898676 | 31899873 | 13.65 | 0.72 | 0.6 | 1 | 0.83 | 5:31898626-31899883 | 32.34 | 1 |
| JB1A25B | 5 | 31998163 | 31999034 | 12.15 | 1 | 1 | 1 | 1 | 5:31998141-31999034 | 31.6 | 1 |
| JB1A25B | 5 | 39163484 | 39164641 | 1.97 | 0.11 | 0.23 | 0 | 0 | 5:39163484-39164645 | 26.68 | 1 |
| JB1A25B | 6 | 5291852 | 5292695 | 17.28 | 1 | 1 | 1 | 1 | 6:5291837-5292712 | 31.42 | 1 |
| JB1A25B | 6 | 9811235 | 9811589 | 13.3 | 1 | 0.99 | 1 | 1 | 6:9811191-9816444 | 24.67 | 0.96 |
| JB1A25B | 6 | 9816029 | 9816399 | 9.63 | 0.99 | 0.99 | 1 | 0.98 | 6:9811191-9816444 | 24.67 | 0.96 |
| JB1A25B | 6 | 13724901 | 13726003 | NA | NA | NA | NA | NA | 6:13724843-13726022 | 31.45 | 1 |
| JB1A25B | 6 | 33482245 | 33483060 | 14.68 | 1 | 1 | 1 | 1 | 6:33482245-33483060 | 28.79 | 1 |
| JB1A25B | 7 | 9022769 | 9023419 | 15.55 | 0.52 | 0.05 | 1 | 1 | 7:9022731-9023457 | 33.43 | 1 |
| JB1A25B | 7 | 9139826 | 9140674 | 12.43 | 0.52 | 0.05 | 1 | 1 | 7:9139826-9140674 | 29.49 | 1 |
| JB1A25B | 7 | 17708120 | 17708720 | 22.93 | 0.46 | 0.05 | 1 | 0.87 | 7:17708068-17708753 | 31.14 | 1 |
| JB1A25B | 8 | 9208445 | 9209129 | 4.49 | 1 | 1 | 1 | 0.99 | 8:9208394-9209273 | 16.25 | 1 |
| JB1A25B | 8 | 11644671 | 11645291 | 9.69 | 0.52 | 0.05 | 1 | 0.99 | 8:11644653-11645314 | 24.16 | 1 |
| JB1A25B | 9 | 5602587 | 5603245 | 23.68 | 0.51 | 0.05 | 1 | 0.98 | 9:5602574-5603257 | 33.27 | 1 |
| JB1A25B | 13 | 10909806 | 10910958 | 7.39 | 0.61 | 1 | 1 | 0.09 | 13:10909806-10910958 | 26.15 | 1 |
| JB1A25B | 14 | 7965871 | 7966740 | 12.28 | 0.99 | 1 | 1 | 0.98 | 14:7965871-7966753 | 26.21 | 1 |
| JB1A25B | 20 | 2428746 | 2429608 | 14.62 | 1 | 1 | 1 | 1 | 20:2428746-2429684 | 31.62 | 1 |
| JB1A25B | AADN03009901.1 | 903 | 1161 | 13.27 | 0.51 | 0.05 | 1 | 0.99 | AADN03009901.1:851-1161 | 28.68 | 1 |
| JB1A25B | AADN03018735.1 | 1077 | 1230 | 9.79 | 0.92 | 0.99 | 1 | 0.83 | AADN03018735.1:281-1293 | 17.63 | 1 |
| JB1A25B | Z | 18808052 | 18808272 | 7.02 | 0.66 | 0.55 | 1 | 0.73 | Z:18807991-18809115 | 31.38 | 1 |
| JB1A25B | Z | 18808832 | 18809063 | 8.83 | 0.63 | 0.49 | 1 | 0.73 | Z:18807991-18809115 | 31.38 | 1 |
| JB1A25B | Z | 32081164 | 32081598 | 11.48 | 1 | 1 | 1 | 1 | Z:32081154-32086293 | 42.22 | 1 |
| JB1A25B | Z | 32085873 | 32086269 | 16.95 | 0.99 | 1 | 1 | 0.98 | Z:32081154-32086293 | 42.22 | 1 |
| JB1A25B | Z | 50801192 | 50801818 | 14.82 | 0.52 | 0.05 | 1 | 1 | Z:50801143-50801818 | 30.23 | 1 |
| JB1A25B | Z | 61304819 | 61306005 | 5.33 | 0.47 | 0 | 0.94 | 0.96 | Z:61304819-61306021 | 37.29 | 1 |
| JB1A25B | Z | 79945559 | 79946417 | 24.99 | 1 | 1 | 1 | 1 | Z:79945501-79946432 | 28.2 | 1 |
| JB1B16A | 1 | 32312218 | 32313105 | 28.25 | 0.99 | 1 | 1 | 0.98 | 1:32312218-32313185 | 25.21 | 1 |
| JB1B16A | 1 | 32508416 | 32508688 | 8.38 | 0.51 | 0.05 | 1 | 0.99 | 1:32508365-32508761 | 31.22 | 1 |
| JB1B16A | 1 | 42541935 | 42542569 | 10.79 | 0.51 | 0.05 | 1 | 0.98 | 1:42541895-42542614 | 34.89 | 1 |
| JB1B16A | 1 | 67550449 | 67551105 | 13.48 | 1 | 1 | 1 | 1 | 1:67550436-67551166 | 35.56 | 1 |
| JB1B16A | 1 | 70454672 | 70455338 | 15.84 | 0.52 | 0.05 | 1 | 1 | 1:70454672-70455338 | 31.31 | 1 |
| JB1B16A | 1 | 90099929 | 90100593 | 9.45 | 0.51 | 0.05 | 1 | 0.98 | 1:90099919-90100600 | 23.96 | 1 |
| JB1B16A | 1 | 96182372 | 96182609 | 6.33 | 0.52 | 0.05 | 1 | 1 | 1:96182372-96182610 | 32.13 | 1 |
| JB1B16A | 1 | 99136310 | 99136952 | 22.94 | 0.48 | 0.05 | 1 | 0.93 | 1:99136310-99136953 | 29.89 | 1 |
| JB1B16A | 1 | 113945474 | 113945672 | 9.43 | 0.36 | 0 | 0 | 0.83 | 1:113945460-113946362 | 31.68 | 1 |
| JB1B16A | 1 | 113946180 | 113946362 | 8.72 | 0.36 | 0 | 0 | 0.83 | 1:113945460-113946362 | 31.68 | 1 |
| JB1B16A | 1 | 126794620 | 126795465 | 14.77 | 1 | 1 | 1 | 1 | 1:126794615-126795484 | 30.14 | 1 |
| JB1B16A | 1 | 140710366 | 140710786 | 14.17 | 1 | 1 | 1 | 0.99 | 1:140710345-140715423 | 30.71 | 0.99 |
| JB1B16A | 1 | 140715003 | 140715398 | 14.74 | 1 | 1 | 1 | 0.99 | 1:140710345-140715423 | 30.71 | 0.99 |
| JB1B16A | 1 | 146731929 | 146732814 | 28.88 | 0.99 | 1 | 1 | 0.98 | 1:146731910-146732833 | 29.03 | 1 |
| JB1B16A | 1 | 146761527 | 146762180 | 14.46 | 0.52 | 0.05 | 1 | 1 | 1:146761527-146762190 | 29.91 | 1 |
| JB1B16A | 1 | 148564562 | 148564837 | 8.17 | 0.51 | 0.05 | 1 | 0.98 | 1:148564562-148568615 | 40.72 | 1 |
| JB1B16A | 1 | 148568367 | 148568555 | 5.25 | 0.48 | 0.05 | 1 | 0.92 | 1:148564562-148568615 | 40.72 | 1 |
| JB1B16A | 1 | 150131206 | 150132006 | 11.79 | 1 | 1 | 1 | 1 | 1:150131206-150132006 | 33.6 | 1 |
| JB1B16A | 1 | 160455274 | 160455648 | 8.46 | 0.51 | 0.05 | 1 | 0.99 | 1:160455218-160455708 | 24.06 | 1 |
| JB1B16A | 1 | 160463974 | 160464234 | 5.03 | 0.52 | 0.05 | 1 | 1 | 1:160463921-160464245 | 27.11 | 1 |
| JB1B16A | 1 | 163694750 | 163695341 | 12.97 | 0.52 | 0.05 | 1 | 1 | 1:163694748-163695372 | 20.45 | 1 |
| JB1B16A | 1 | 164414998 | 164415246 | 8.52 | 0.51 | 0.05 | 1 | 0.98 | 1:164414998-164416037 | 28.66 | 1 |
| JB1B16A | 1 | 164415796 | 164416037 | 4.29 | 0.51 | 0.05 | 1 | 0.98 | 1:164414998-164416037 | 28.66 | 1 |
| JB1B16A | 1 | 180746896 | 180747131 | 12.65 | 0.51 | 0.05 | 1 | 0.99 | 1:180746896-180747970 | 33.71 | 1 |
| JB1B16A | 1 | 180747692 | 180747964 | 16.99 | 0.52 | 0.05 | 1 | 1 | 1:180746896-180747970 | 33.71 | 1 |
| JB1B16A | 1 | 182832885 | 182834052 | 15.42 | 0.92 | 1 | 1 | 0.81 | 1:182832847-182834082 | 25.72 | 0.99 |
| JB1B16A | 2 | 21240696 | 21240948 | 8.16 | 0.51 | 0.05 | 1 | 0.98 | 2:21240687-21244651 | 41.74 | 1 |
| JB1B16A | 2 | 50798296 | 50798951 | 22.7 | 0.51 | 0.05 | 1 | 0.99 | 2:50798296-50798951 | 28.47 | 1 |
| JB1B16A | 2 | 65341357 | 65341971 | 10.96 | 0.51 | 0.05 | 1 | 0.98 | 2:65341352-65341989 | 28.75 | 1 |
| JB1B16A | 2 | 81965027 | 81965237 | 4.15 | 0.51 | 0.05 | 1 | 0.99 | 2:81965001-81966072 | 23.14 | 1 |
| JB1B16A | 2 | 81965809 | 81966029 | 8.56 | 0.51 | 0.05 | 1 | 0.99 | 2:81965001-81966072 | 23.14 | 1 |
| JB1B16A | 2 | 92857020 | 92858181 | 5.52 | 0.77 | 0.99 | 1 | 0.49 | 2:92856996-92858219 | 24.05 | 1 |
| JB1B16A | 2 | 94577762 | 94577976 | 5.29 | 0.52 | 0.05 | 1 | 0.99 | 2:94577735-94578007 | 31.22 | 1 |
| JB1B16A | 2 | 95480969 | 95481814 | 23.8 | 1 | 1 | 1 | 0.99 | 2:95480957-95481836 | 27.43 | 1 |
| JB1B16A | 2 | 104297236 | 104297857 | 10.56 | 0.52 | 0.05 | 1 | 1 | 2:104297204-104297861 | 30.81 | 1 |
| JB1B16A | 2 | 131563798 | 131564026 | 7.15 | 0.51 | 0.05 | 1 | 0.98 | 2:131563757-131567794 | 33.57 | 1 |
| JB1B16A | 2 | 131567510 | 131567780 | 8.93 | 0.52 | 0.05 | 1 | 1 | 2:131563757-131567794 | 33.57 | 1 |
| JB1B16A | 2 | 133607552 | 133608408 | 17.01 | 0.99 | 1 | 1 | 0.98 | 2:133607552-133608408 | 29.44 | 1 |
| JB1B16A | 3 | 19821802 | 19822989 | 6.48 | 0.77 | 0.9 | 0 | 0.73 | 3:19821802-19823007 | 23.13 | 1 |
| JB1B16A | 3 | 22595889 | 22596501 | 9.83 | 0.52 | 0.05 | 1 | 1 | 3:22595889-22596501 | 32.23 | 1 |
| JB1B16A | 3 | 34783087 | 34783374 | 7.44 | 0.51 | 0.05 | 1 | 0.98 | 3:34782953-34783379 | 34.11 | 1 |
| JB1B16A | 3 | 40908309 | 40908869 | 8.48 | 0.47 | 0.05 | 1 | 0.9 | 3:40908270-40908901 | 28.96 | 1 |
| JB1B16A | 3 | 49572073 | 49572929 | 17.76 | 1 | 1 | 1 | 1 | 3:49572068-49572931 | 31.57 | 1 |
| JB1B16A | 3 | 54250116 | 54250361 | 6.53 | 0.51 | 0.05 | 1 | 0.98 | 3:54250109-54254158 | 34.96 | 1 |
| JB1B16A | 3 | 54253914 | 54254146 | 8.76 | 0.52 | 0.05 | 1 | 1 | 3:54250109-54254158 | 34.96 | 1 |
| JB1B16A | 3 | 59937570 | 59938181 | 11.58 | 0.51 | 0.05 | 1 | 0.98 | 3:59937540-59938220 | 28.28 | 1 |
| JB1B16A | 3 | 70604586 | 70605370 | 9.21 | 0.99 | 1 | 1 | 0.98 | 3:70604555-70605387 | 22.75 | 1 |
| JB1B16A | 3 | 73561882 | 73563056 | 5.16 | 0.43 | 0.7 | 1 | 0.02 | 3:73561879-73563101 | 32.4 | 1 |
| JB1B16A | 3 | 83776907 | 83777146 | 11.43 | 0.46 | 0.05 | 1 | 0.87 | 3:83776894-83777945 | 31.7 | 1 |
| JB1B16A | 3 | 83777704 | 83777914 | 14.37 | 0.47 | 0.05 | 1 | 0.89 | 3:83776894-83777945 | 31.7 | 1 |
| JB1B16A | 3 | 84550518 | 84551452 | 13.23 | 1 | 1 | 1 | 1 | 3:84550518-84551452 | 32.44 | 1 |
| JB1B16A | 3 | 93697667 | 93698539 | 13.92 | 1 | 1 | 1 | 1 | 3:93697657-93698572 | 26.06 | 1 |
| JB1B16A | 4 | 11124004 | 11124404 | 12.64 | 0.99 | 1 | 1 | 0.98 | 4:11123901-11124467 | 25.73 | 1 |
| JB1B16A | 4 | 25887286 | 25887901 | 14.7 | 0.52 | 0.05 | 1 | 1 | 4:25887286-25887901 | 31.29 | 1 |
| JB1B16A | 4 | 26530919 | 26531136 | 12.64 | 0.51 | 0.05 | 1 | 0.98 | 4:26530893-26531990 | 29.71 | 1 |
| JB1B16A | 4 | 26531711 | 26531923 | 12.87 | 0.51 | 0.05 | 1 | 0.98 | 4:26530893-26531990 | 29.71 | 1 |
| JB1B16A | 4 | 27394575 | 27395212 | 16.8 | 0.52 | 0.05 | 1 | 1 | 4:27394572-27395212 | 36.02 | 1 |
| JB1B16A | 4 | 30632723 | 30633367 | 27.1 | 0.51 | 0.05 | 1 | 0.98 | 4:30632716-30633375 | 27.69 | 1 |
| JB1B16A | 4 | 70552005 | 70552666 | 4.65 | 0.99 | 1 | 1 | 0.98 | 4:70551829-70552669 | 19.4 | 1 |
| JB1B16A | 4 | 71535558 | 71536348 | 11.62 | 0.99 | 1 | 1 | 0.98 | 4:71535538-71536391 | 26.82 | 1 |
| JB1B16A | 4 | 72705269 | 72705454 | 9.08 | 0.52 | 0.05 | 1 | 1 | 4:72705264-72705454 | 27.72 | 1 |
| JB1B16A | 5 | 2705077 | 2706254 | 3.92 | 0.95 | 0.9 | 1 | 0.99 | 5:2705052-2706284 | 29.86 | 1 |
| JB1B16A | 5 | 6164288 | 6165465 | 4.36 | 0.29 | 0 | 0 | 0.68 | 5:6164269-6165523 | 20.11 | 1 |
| JB1B16A | 5 | 31303877 | 31305055 | 1 | 0.31 | 0.14 | 1 | 0.41 | 5:31303866-31305068 | 33.72 | 1 |
| JB1B16A | 5 | 31898689 | 31899877 | 7.05 | 0.68 | 0.79 | 1 | 0.51 | 5:31898626-31899883 | 32.36 | 1 |
| JB1B16A | 5 | 31998151 | 31999029 | 30.97 | 1 | 1 | 1 | 1 | 5:31998141-31999034 | 31.19 | 1 |
| JB1B16A | 5 | 40464028 | 40464903 | 14.46 | 0.99 | 0.99 | 1 | 0.99 | 5:40463978-40464915 | 35.51 | 1 |
| JB1B16A | 6 | 9811227 | 9811583 | 11.86 | 0.99 | 0.99 | 1 | 1 | 6:9811191-9816444 | 26.7 | 0.96 |
| JB1B16A | 6 | 9816012 | 9816428 | 18.81 | 0.99 | 0.99 | 1 | 0.98 | 6:9811191-9816444 | 26.7 | 0.96 |
| JB1B16A | 6 | 13724864 | 13726022 | 5.23 | 0.68 | 0.55 | 1 | 0.78 | 6:13724843-13726022 | 32.9 | 1 |
| JB1B16A | 7 | 9022763 | 9023428 | 14.59 | 0.52 | 0.05 | 1 | 1 | 7:9022731-9023457 | 31.07 | 1 |
| JB1B16A | 7 | 15504223 | 15504887 | 15.06 | 0.52 | 0.05 | 1 | 1 | 7:15504223-15504887 | 29.22 | 1 |
| JB1B16A | 7 | 17708093 | 17708707 | 24.97 | 0.51 | 0.04 | 1 | 1 | 7:17708068-17708753 | 31.44 | 1 |
| JB1B16A | 8 | 9208424 | 9209004 | 5.75 | 1 | 1 | 1 | 0.99 | 8:9208394-9209273 | 17.77 | 1 |
| JB1B16A | 8 | 11644724 | 11645314 | 13.26 | 0.51 | 0.05 | 1 | 0.99 | 8:11644653-11645314 | 29.43 | 1 |
| JB1B16A | 9 | 5602608 | 5603257 | 15.66 | 0.51 | 0.05 | 1 | 0.98 | 9:5602574-5603257 | 31.79 | 1 |
| JB1B16A | 12 | 3240723 | 3240870 | 31.87 | 0.63 | 1 | 1 | 0.14 | 12:3240697-3240898 | 143.42 | 1 |
| JB1B16A | 12 | 17400825 | 17401479 | 13.48 | 0.52 | 0.05 | 1 | 1 | 12:17400816-17401479 | 30.66 | 1 |
| JB1B16A | 17 | 180676 | 181518 | 15.78 | 0.99 | 0.99 | 1 | 0.98 | 17:180636-181570 | 27.7 | 1 |
| JB1B16A | 20 | 2428756 | 2429608 | 15.63 | 1 | 1 | 1 | 1 | 20:2428746-2429684 | 29.61 | 1 |
| JB1B16A | 20 | 7661897 | 7662775 | 14.78 | 1 | 1 | 1 | 1 | 20:7661897-7662786 | 32.79 | 1 |
| JB1B16A | AADN03024906.1 | 4129 | 4351 | 5.99 | 0.51 | 0.05 | 1 | 0.98 | AADN03024906.1:3470-4351 | 20.54 | 1 |
| JB1B16A | JH375212.1 | 37474 | 37702 | 4.65 | 0.51 | 0.05 | 1 | 0.98 | JH375212.1:37241-37702 | 12.13 | 1 |
| JB1B16A | JH375231.1 | 5524 | 5780 | 6.21 | 0.74 | 0.55 | 1 | 0.93 | JH375231.1:4846-8915 | 12.07 | 1 |
| JB1B16A | JH375231.1 | 8675 | 8915 | 3.72 | 0.73 | 0.47 | 1 | 1 | JH375231.1:4846-8915 | 12.07 | 1 |
| JB1B16A | JH375237.1 | 50517 | 50701 | 6.36 | 0.34 | 0 | 0 | 0.79 | JH375237.1:49865-50701 | 12.76 | 1 |
| JB1B16A | JH376310.1 | 6583 | 6956 | 4.97 | 0.93 | 0.99 | 1 | 0.85 | JH376310.1:6513-7054 | 12.44 | 1 |
| JB1B16A | JH376323.1 | 17955 | 18124 | 5.64 | 0.27 | 0 | 0 | 0.63 | JH376323.1:17955-18124 | 55.88 | 1 |
| JB1B16A | Z | 18808026 | 18808287 | 5.56 | 0.63 | 0.49 | 1 | 0.73 | Z:18807991-18809115 | 14.56 | 1 |
| JB1B16A | Z | 18808871 | 18809058 | 6.77 | 0.52 | 0.27 | 1 | 0.73 | Z:18807991-18809115 | 14.56 | 1 |
| JB1B16A | Z | 32081187 | 32081544 | 8.07 | 1 | 1 | 1 | 1 | Z:32081154-32086293 | 32.64 | 1 |
| JB1B16A | Z | 32085917 | 32086266 | 6.25 | 0.99 | 1 | 1 | 0.98 | Z:32081154-32086293 | 32.64 | 1 |
| JB1B16A | Z | 61304877 | 61305990 | 7.4 | 0.6 | 0.29 | 1 | 0.9 | Z:61304819-61306021 | 18.12 | 1 |
| JB1B16A | Z | 79945555 | 79946424 | 15.51 | 1 | 1 | 1 | 1 | Z:79945501-79946432 | 13.81 | 1 |
| JB2A04B.1 | 1 | 32312244 | 32313011 | 12.12 | 0.99 | 1 | 1 | 0.98 | 1:32312218-32313185 | 27.61 | 1 |
| JB2A04B.1 | 1 | 32508426 | 32508677 | 7.12 | 0.51 | 0.05 | 1 | 0.99 | 1:32508365-32508761 | 33.53 | 1 |
| JB2A04B.1 | 1 | 41318900 | 41319764 | 14.23 | 1 | 1 | 1 | 0.99 | 1:41318900-41319777 | 30.1 | 1 |
| JB2A04B.1 | 1 | 70454693 | 70455311 | 14.03 | 0.52 | 0.05 | 1 | 1 | 1:70454672-70455338 | 26.38 | 1 |
| JB2A04B.1 | 1 | 95854597 | 95855207 | 8.29 | 0.52 | 0.05 | 1 | 1 | 1:95854597-95855224 | 31.13 | 1 |
| JB2A04B.1 | 1 | 96156972 | 96157369 | 8.94 | 0.99 | 1 | 1 | 0.98 | 1:96156897-96157369 | 36.91 | 1 |
| JB2A04B.1 | 1 | 99050565 | 99051170 | 9.73 | 0.52 | 0.05 | 1 | 1 | 1:99050515-99051202 | 27.66 | 1 |
| JB2A04B.1 | 1 | 99136333 | 99136902 | 15.07 | 0.51 | 0.05 | 1 | 0.98 | 1:99136310-99136953 | 27.12 | 1 |
| JB2A04B.1 | 1 | 101333546 | 101334362 | 14.91 | 1 | 1 | 1 | 1 | 1:101333491-101334404 | 27.73 | 1 |
| JB2A04B.1 | 1 | 126794615 | 126795468 | 18.56 | 1 | 1 | 1 | 1 | 1:126794615-126795484 | 31.68 | 1 |
| JB2A04B.1 | 1 | 140710379 | 140710803 | 15.06 | 1 | 1 | 1 | 0.99 | 1:140710345-140715423 | 41.19 | 1 |
| JB2A04B.1 | 1 | 140714997 | 140715410 | 10.16 | 1 | 1 | 1 | 1 | 1:140710345-140715423 | 41.19 | 1 |
| JB2A04B.1 | 1 | 146731926 | 146732761 | 10.28 | 0.99 | 1 | 1 | 0.98 | 1:146731910-146732833 | 27.6 | 1 |
| JB2A04B.1 | 1 | 149162780 | 149163120 | 6.86 | 1 | 1 | 1 | 0.99 | 1:149162760-149166227 | 32.94 | 1 |
| JB2A04B.1 | 1 | 149165819 | 149166198 | 5.14 | 1 | 1 | 1 | 1 | 1:149162760-149166227 | 32.94 | 1 |
| JB2A04B.1 | 1 | 160455263 | 160455478 | 13.22 | 0.51 | 0.05 | 1 | 0.98 | 1:160455218-160455708 | 29.13 | 1 |
| JB2A04B.1 | 1 | 160464011 | 160464221 | 8.31 | 0.52 | 0.05 | 1 | 1 | 1:160463921-160464245 | 33.28 | 1 |
| JB2A04B.1 | 1 | 163501538 | 163501759 | 6.82 | 0.52 | 0.05 | 1 | 1 | 1:163501535-163501830 | 25.47 | 1 |
| JB2A04B.1 | 1 | 163694782 | 163695352 | 10.39 | 0.52 | 0.05 | 1 | 1 | 1:163694748-163695372 | 23.37 | 1 |
| JB2A04B.1 | 1 | 180746914 | 180747135 | 9.56 | 0.52 | 0.05 | 1 | 0.99 | 1:180746896-180747970 | 31.97 | 1 |
| JB2A04B.1 | 1 | 180747693 | 180747951 | 16.88 | 0.51 | 0.05 | 1 | 0.99 | 1:180746896-180747970 | 31.97 | 1 |
| JB2A04B.1 | 1 | 182832907 | 182834057 | 18.41 | 0.91 | 0.99 | 1 | 0.81 | 1:182832847-182834082 | 26.81 | 1 |
| JB2A04B.1 | 1 | 187340668 | 187340887 | 7.84 | 0.51 | 0.05 | 1 | 0.98 | 1:187340668-187342356 | 21.53 | 1 |
| JB2A04B.1 | 1 | 187342105 | 187342356 | 6.44 | 0.52 | 0.05 | 1 | 0.99 | 1:187340668-187342356 | 21.53 | 1 |
| JB2A04B.1 | 2 | 51801055 | 51801942 | 34.07 | 0.99 | 0.99 | 1 | 0.99 | 2:51801055-51801954 | 34.47 | 1 |
| JB2A04B.1 | 2 | 53111793 | 53112005 | 13.83 | 0.94 | 0.88 | 1 | 0.99 | 2:53111756-53112017 | 27.26 | 1 |
| JB2A04B.1 | 2 | 82574335 | 82575471 | 4.31 | 0.48 | 0.94 | 0 | 0 | 2:82574297-82575518 | 30.92 | 1 |
| JB2A04B.1 | 2 | 92857013 | 92858192 | 7.5 | 0.59 | 0.61 | 1 | 0.5 | 2:92856996-92858219 | 34.58 | 1 |
| JB2A04B.1 | 2 | 94577735 | 94578007 | 5.48 | 0.51 | 0.05 | 1 | 0.98 | 2:94577735-94578007 | 34.55 | 1 |
| JB2A04B.1 | 2 | 95480982 | 95481804 | 14.04 | 1 | 1 | 1 | 1 | 2:95480957-95481836 | 32.11 | 1 |
| JB2A04B.1 | 2 | 131563767 | 131564002 | 8.19 | 0.51 | 0.05 | 1 | 0.98 | 2:131563757-131567794 | 32 | 1 |
| JB2A04B.1 | 2 | 131567517 | 131567786 | 5.74 | 0.52 | 0.05 | 1 | 1 | 2:131563757-131567794 | 32 | 1 |
| JB2A04B.1 | 3 | 19821865 | 19822990 | 12.52 | 0.99 | 0.99 | 1 | 0.99 | 3:19821802-19823007 | 27.85 | 1 |
| JB2A04B.1 | 3 | 34783149 | 34783359 | 9.5 | 0.51 | 0.05 | 1 | 0.98 | 3:34782953-34783379 | 41.52 | 1 |
| JB2A04B.1 | 3 | 59937540 | 59938204 | 27.24 | 0.51 | 0.05 | 1 | 0.99 | 3:59937540-59938220 | 25.6 | 1 |
| JB2A04B.1 | 3 | 70604615 | 70605378 | 12.24 | 1 | 1 | 1 | 0.99 | 3:70604555-70605387 | 25.26 | 1 |
| JB2A04B.1 | 3 | 73561902 | 73563062 | 1 | 0.24 | 0.47 | 0 | 0 | 3:73561879-73563101 | 32 | 1 |
| JB2A04B.1 | 3 | 83776905 | 83777146 | 12.47 | 0.47 | 0.05 | 1 | 0.89 | 3:83776894-83777945 | 31.61 | 1 |
| JB2A04B.1 | 3 | 83777717 | 83777932 | 16.95 | 0.51 | 0.05 | 1 | 0.98 | 3:83776894-83777945 | 31.61 | 1 |
| JB2A04B.1 | 3 | 84550557 | 84551402 | 13.5 | 1 | 1 | 1 | 1 | 3:84550518-84551452 | 27.52 | 1 |
| JB2A04B.1 | 3 | 93697657 | 93698556 | 31.25 | 0.99 | 1 | 1 | 0.98 | 3:93697657-93698572 | 28.86 | 1 |
| JB2A04B.1 | 4 | 11124054 | 11124406 | 11.72 | 0.99 | 1 | 1 | 0.98 | 4:11123901-11124467 | 27.3 | 1 |
| JB2A04B.1 | 4 | 26530920 | 26531147 | 9.71 | 0.51 | 0.05 | 1 | 0.98 | 4:26530893-26531990 | 29.14 | 1 |
| JB2A04B.1 | 4 | 26531693 | 26531907 | 9.36 | 0.51 | 0.05 | 1 | 0.98 | 4:26530893-26531990 | 29.14 | 1 |
| JB2A04B.1 | 4 | 30632730 | 30633356 | 28.46 | 0.51 | 0.05 | 1 | 0.98 | 4:30632716-30633375 | 33.34 | 1 |
| JB2A04B.1 | 4 | 78901609 | 78902511 | 31.33 | 1 | 1 | 1 | 1 | 4:78901609-78902511 | 31.14 | 1 |
| JB2A04B.1 | 5 | 2705079 | 2706260 | 6.54 | 0.68 | 0.6 | 1 | 0.73 | 5:2705052-2706284 | 27 | 1 |
| JB2A04B.1 | 5 | 6164302 | 6165488 | 2.66 | 0.28 | 0 | 0 | 0.65 | 5:6164269-6165523 | 27.35 | 1 |
| JB2A04B.1 | 5 | 31303888 | 31305034 | 1 | 0.22 | 0.43 | 0 | 0 | 5:31303866-31305068 | 31.41 | 1 |
| JB2A04B.1 | 5 | 31898713 | 31899873 | 14.35 | 0.74 | 0.84 | 1 | 0.58 | 5:31898626-31899883 | 30.03 | 1 |
| JB2A04B.1 | 5 | 31998150 | 31999021 | 17.53 | 1 | 1 | 1 | 1 | 5:31998141-31999034 | 35.16 | 1 |
| JB2A04B.1 | 6 | 9811191 | 9811595 | 10.6 | 0.99 | 0.99 | 1 | 1 | 6:9811191-9816444 | 27.91 | 0.99 |
| JB2A04B.1 | 6 | 9816055 | 9816420 | 12.32 | 0.99 | 0.99 | 1 | 0.98 | 6:9811191-9816444 | 27.91 | 0.99 |
| JB2A04B.1 | 6 | 13724874 | 13726005 | NA | NA | NA | NA | NA | 6:13724843-13726022 | 30.99 | 1 |
| JB2A04B.1 | 7 | 9022748 | 9023383 | 11.37 | 0.52 | 0.05 | 1 | 1 | 7:9022731-9023457 | 31.84 | 1 |
| JB2A04B.1 | 7 | 15504258 | 15504864 | 13.6 | 0.52 | 0.05 | 1 | 1 | 7:15504223-15504887 | 35.62 | 1 |
| JB2A04B.1 | 7 | 17708102 | 17708708 | 26.62 | 0.51 | 0.04 | 1 | 0.98 | 7:17708068-17708753 | 30.32 | 1 |
| JB2A04B.1 | 8 | 9208394 | 9208772 | 9.04 | 1 | 1 | 1 | 0.99 | 8:9208394-9209273 | 21.63 | 1 |
| JB2A04B.1 | 8 | 11644653 | 11645312 | 29.33 | 0.51 | 0.05 | 1 | 0.99 | 8:11644653-11645314 | 35.31 | 1 |
| JB2A04B.1 | 9 | 5602576 | 5603172 | 15.78 | 0.51 | 0.05 | 1 | 0.99 | 9:5602574-5603257 | 34 | 1 |
| JB2A04B.1 | 12 | 3240722 | 3240870 | 40.88 | 0.57 | 1 | 1 | 0 | 12:3240697-3240898 | 141.61 | 1 |
| JB2A04B.1 | 13 | 2970495 | 2971368 | 14.42 | 1 | 1 | 1 | 1 | 13:2970495-2971368 | 25.74 | 1 |
| JB2A04B.1 | 14 | 7965897 | 7966753 | 16.59 | 0.99 | 1 | 1 | 0.98 | 14:7965871-7966753 | 26.46 | 1 |
| JB2A04B.1 | 17 | 180660 | 181544 | 16.74 | 0.99 | 0.99 | 1 | 0.98 | 17:180636-181570 | 29.25 | 1 |
| JB2A04B.1 | Z | 18808058 | 18808297 | 7.93 | 0.63 | 0.49 | 1 | 0.73 | Z:18807991-18809115 | 30.92 | 1 |
| JB2A04B.1 | Z | 18808817 | 18809070 | 12.76 | 0.63 | 0.49 | 1 | 0.73 | Z:18807991-18809115 | 30.92 | 1 |
| JB2A04B.1 | Z | 32081154 | 32081572 | 9.52 | 1 | 1 | 1 | 1 | Z:32081154-32086293 | 36.28 | 1 |
| JB2A04B.1 | Z | 32085873 | 32086266 | 18.8 | 0.99 | 1 | 1 | 0.98 | Z:32081154-32086293 | 36.28 | 1 |
| JB2A04B.1 | Z | 50801143 | 50801813 | 29.13 | 0.52 | 0.05 | 1 | 1 | Z:50801143-50801818 | 28.05 | 1 |
| JB2A04B.1 | Z | 61304819 | 61305990 | 8.81 | 0.62 | 0.26 | 1 | 0.98 | Z:61304819-61306021 | 35.48 | 1 |
| JB2A04B.1 | Z | 78844870 | 78846040 | 6.26 | 0.98 | 0.97 | 1 | 1 | Z:78844863-78846057 | 26.22 | 1 |
| JB2A04B.1 | Z | 79945547 | 79946430 | 21.66 | 1 | 1 | 1 | 1 | Z:79945501-79946432 | 26.45 | 1 |
| Line15 | 1 | 18836195 | 18836791 | 4.23 | 1 | 1 | 1 | 1 | 1:18836195-18836791 | 10.47 | 1 |
| Line15 | 1 | 32312327 | 32312977 | 8.57 | 0.99 | 1 | 1 | 0.98 | 1:32312218-32313185 | 8.16 | 1 |
| Line15 | 1 | 41319063 | 41319612 | 5.71 | 0.98 | 0.99 | 1 | 0.98 | 1:41318900-41319777 | 9.1 | 1 |
| Line15 | 1 | 63571544 | 63571952 | 2.52 | 0.45 | 0.05 | 1 | 0.84 | 1:63571454-63572079 | 5.16 | 0.95 |
| Line15 | 1 | 67550570 | 67551002 | 10.89 | 0.99 | 1 | 1 | 0.98 | 1:67550436-67551166 | 19.83 | 1 |
| Line15 | 1 | 99136437 | 99136649 | 1 | 0.35 | 0 | 0.59 | 0.73 | 1:99136310-99136953 | 13.37 | 1 |
| Line15 | 1 | 140710730 | 140710851 | 4.01 | 1 | 1 | 1 | 0.99 | 1:140710345-140715423 | 23.37 | 1 |
| Line15 | 1 | 140714972 | 140715185 | 6.96 | 1 | 1 | 1 | 1 | 1:140710345-140715423 | 23.37 | 1 |
| Line15 | 1 | 146732070 | 146732601 | 8.41 | 0.99 | 1 | 1 | 0.98 | 1:146731910-146732833 | 9.95 | 1 |
| Line15 | 1 | 163695172 | 163695275 | 1.96 | 0.36 | 0 | 0 | 0.83 | 1:163694748-163695372 | 8.13 | 1 |
| Line15 | 1 | 164415146 | 164415944 | 3.95 | 0.51 | 0.05 | 1 | 0.98 | 1:164414998-164416037 | 12.13 | 1 |
| Line15 | 1 | 182832958 | 182833933 | 6.52 | 0.91 | 0.85 | 1 | 0.96 | 1:182832847-182834082 | 17.95 | 1 |
| Line15 | 1 | 185857104 | 185857263 | 2.6 | 0.51 | 0.05 | 1 | 0.98 | 1:185856831-185857511 | 10.89 | 0.99 |
| Line15 | 2 | 3491599 | 3492119 | 3.76 | 0.92 | 0.84 | 1 | 1 | 2:3491394-3492279 | 10.25 | 1 |
| Line15 | 2 | 10512429 | 10512765 | 2.97 | 0.51 | 0.05 | 1 | 0.99 | 2:10512425-10512887 | 12.42 | 1 |
| Line15 | 2 | 21240803 | 21240942 | 1.96 | 0.47 | 0.05 | 1 | 0.9 | 2:21240687-21244651 | 12.38 | 1 |
| Line15 | 2 | 34296700 | 34297275 | 7.08 | 0.97 | 1 | 1 | 0.93 | 2:34296518-34297385 | 10.76 | 1 |
| Line15 | 2 | 51801179 | 51801817 | 5.39 | 0.91 | 0.83 | 1 | 0.99 | 2:51801055-51801954 | 9.52 | 1 |
| Line15 | 2 | 81965188 | 81965949 | 2.24 | 0.51 | 0.05 | 1 | 0.99 | 2:81965001-81966072 | 8.85 | 0.98 |
| Line15 | 2 | 82574445 | 82575400 | 5.04 | 0.89 | 0.99 | 1 | 0.77 | 2:82574297-82575518 | 11.07 | 1 |
| Line15 | 2 | 92857795 | 92858052 | 1.85 | 0.83 | 0.95 | 0 | 0.8 | 2:92856996-92858219 | 10.08 | 1 |
| Line15 | 2 | 124765235 | 124765520 | 1.92 | 0.42 | 0.05 | 1 | 0.77 | 2:124765081-124765718 | 6.41 | 1 |
| Line15 | 2 | 142114261 | 142114437 | 3.39 | 0.51 | 0.05 | 1 | 0.98 | 2:142113866-142114543 | 12.29 | 1 |
| Line15 | 3 | 19822167 | 19822895 | 1 | 0.35 | 0.68 | 0 | 0 | 3:19821802-19823007 | 7.54 | 1 |
| Line15 | 3 | 36156554 | 36156751 | 2.35 | 0.51 | 0.05 | 1 | 0.98 | 3:36156197-36156846 | 13.45 | 1 |
| Line15 | 3 | 73562001 | 73562905 | 2.11 | 0.23 | 0.45 | 0 | 0 | 3:73561879-73563101 | 8.82 | 1 |
| Line15 | 3 | 83407767 | 83408373 | 2.1 | 1 | 1 | 1 | 1 | 3:83407619-83408514 | 5.79 | 0.83 |
| Line15 | 3 | 83777062 | 83777833 | 2.18 | 0.38 | 0 | 0 | 0.87 | 3:83776894-83777945 | 11.08 | 1 |
| Line15 | 3 | 99426134 | 99426459 | 1.57 | 0.45 | 0 | 0.82 | 0.93 | 3:99426024-99426621 | 6.76 | 1 |
| Line15 | 4 | 11123990 | 11124306 | 3.6 | 0.99 | 1 | 1 | 0.98 | 4:11123901-11124467 | 11.38 | 1 |
| Line15 | 4 | 29234423 | 29234592 | 1.43 | 0.46 | 0.05 | 1 | 0.87 | 4:29234085-29234741 | 11.39 | 1 |
| Line15 | 4 | 30595812 | 30596406 | 4.01 | 0.95 | 0.93 | 1 | 0.96 | 4:30595679-30596557 | 11.57 | 1 |
| Line15 | 4 | 66767916 | 66768002 | 1.7 | 0.25 | 0 | 0 | 0.59 | 4:66767916-66768002 | 13.32 | 1 |
| Line15 | 4 | 70552132 | 70552461 | 2.02 | 0.99 | 1 | 1 | 0.98 | 4:70551829-70552669 | 5.86 | 0.99 |
| Line15 | 4 | 78859448 | 78859880 | 4.31 | 0.51 | 0.05 | 1 | 0.98 | 4:78859384-78859987 | 11.69 | 1 |
| Line15 | 5 | 2705225 | 2706072 | 6.36 | 0.65 | 0.46 | 1 | 0.83 | 5:2705052-2706284 | 14.86 | 1 |
| Line15 | 5 | 6164408 | 6165387 | 5.34 | 0.99 | 1 | 1 | 0.98 | 5:6164269-6165523 | 12.93 | 1 |
| Line15 | 5 | 31304008 | 31304349 | 5.14 | 0.94 | 0.88 | 1 | 1 | 5:31303866-31305068 | 10.65 | 1 |
| Line15 | 5 | 31898937 | 31899596 | 2.89 | 0.82 | 0.66 | 1 | 0.98 | 5:31898626-31899883 | 8.03 | 1 |
| Line15 | 5 | 41299637 | 41300040 | 5.27 | 1 | 1 | 1 | 1 | 5:41299361-41300140 | 10.96 | 1 |
| Line15 | 6 | 9811468 | 9811663 | 3.37 | 0.99 | 0.99 | 1 | 1 | 6:9811191-9816444 | 13.57 | 0.98 |
| Line15 | 6 | 9815943 | 9816222 | 6.51 | 0.99 | 0.98 | 1 | 1 | 6:9811191-9816444 | 13.57 | 0.98 |
| Line15 | 6 | 13724964 | 13725802 | 1 | 0.31 | 0 | 0 | 0.73 | 6:13724843-13726022 | 8.76 | 1 |
| Line15 | 7 | 17708190 | 17708612 | 2.55 | 0.41 | 0 | 0.59 | 0.88 | 7:17708068-17708753 | 12.57 | 1 |
| Line15 | 8 | 9208667 | 9208948 | 2.2 | 0.89 | 0.92 | 1 | 0.85 | 8:9208394-9209273 | 5.5 | 0.94 |
| Line15 | 12 | 3240753 | 3240836 | 2.12 | 0.24 | 0.47 | 0 | 0 | 12:3240697-3240898 | 66.56 | 1 |
| Line15 | 12 | 3277803 | 3278290 | 16.16 | 0.99 | 1 | 1 | 0.98 | 12:3277416-3278316 | 50.9 | 1 |
| Line15 | 13 | 6876742 | 6877190 | 6.04 | 0.93 | 0.96 | 1 | 0.88 | 13:6876434-6877337 | 8.86 | 1 |
| Line15 | 17 | 180746 | 181452 | 6.41 | 0.98 | 0.99 | 1 | 0.98 | 17:180636-181570 | 13.16 | 1 |
| Line15 | 20 | 7662107 | 7662678 | 3.44 | 0.96 | 1 | 1 | 0.9 | 20:7661897-7662786 | 8.58 | 1 |
| Line15 | AADN03016632.1 | 765 | 923 | 11.08 | 0.52 | 0.05 | 1 | 1 | AADN03016632.1:765-923 | 32.26 | 1 |
| Line15 | AADN03018735.1 | 281 | 629 | 11.54 | 0.99 | 0.99 | 1 | 0.98 | AADN03018735.1:281-1293 | 30.65 | 1 |
| Line15 | AADN03019391.1 | 477 | 583 | 2.26 | 0.47 | 0.03 | 1 | 0.9 | AADN03019391.1:477-583 | 11.09 | 1 |
| Line15 | AADN03024906.1 | 3470 | 4022 | 1.35 | 0.49 | 0.05 | 1 | 0.95 | AADN03024906.1:3470-4351 | 7.79 | 1 |
| Line15 | JH376310.1 | 6807 | 7029 | 2.35 | 0.95 | 0.92 | 1 | 0.98 | JH376310.1:6513-7054 | 3.92 | 1 |
| Line15 | Z | 49125790 | 49126200 | 2.86 | 0.51 | 0.05 | 1 | 0.98 | Z:49125790-49126200 | 3.22 | 0.9 |
| Line15 | Z | 61305580 | 61305813 | 3.04 | 0.94 | 0.9 | 1 | 0.98 | Z:61304819-61306021 | 5.15 | 1 |
| Line15 | Z | 64574244 | 64574638 | 2.67 | 0.46 | 0.05 | 1 | 0.87 | Z:64574244-64574638 | 6.55 | 1 |
| Line15 | Z | 79945647 | 79946302 | 4.31 | 1 | 1 | 1 | 1 | Z:79945501-79946432 | 7.15 | 1 |
| Line6 | 1 | 15904041 | 15904142 | 2.86 | 0.4 | 0 | 0 | 0.94 | 1:15904038-15904160 | 22.44 | 1 |
| Line6 | 1 | 32312254 | 32313105 | 10.42 | 0.97 | 1 | 1 | 0.93 | 1:32312218-32313185 | 10.35 | 1 |
| Line6 | 1 | 41318918 | 41319756 | 9.91 | 1 | 1 | 1 | 0.99 | 1:41318900-41319777 | 12.97 | 1 |
| Line6 | 1 | 42541971 | 42542567 | 3.37 | 0.42 | 0.05 | 1 | 0.77 | 1:42541895-42542614 | 15.93 | 1 |
| Line6 | 1 | 43882652 | 43883765 | 3.18 | 0.7 | 1 | 1 | 0.31 | 1:43882636-43883815 | 12.88 | 1 |
| Line6 | 1 | 67550473 | 67551045 | 2.95 | 1 | 1 | 1 | 1 | 1:67550436-67551166 | 25.26 | 1 |
| Line6 | 1 | 96182406 | 96182591 | 5.53 | 0.52 | 0.05 | 1 | 1 | 1:96182372-96182610 | 15.37 | 1 |
| Line6 | 1 | 99050545 | 99050961 | 7.86 | 0.52 | 0.05 | 1 | 1 | 1:99050515-99051202 | 14.29 | 1 |
| Line6 | 1 | 140710431 | 140710846 | 11.68 | 1 | 1 | 1 | 0.99 | 1:140710345-140715423 | 38.64 | 1 |
| Line6 | 1 | 140715058 | 140715226 | 2.14 | 0.82 | 0.86 | 0 | 0.89 | 1:140710345-140715423 | 38.64 | 1 |
| Line6 | 1 | 157450121 | 157450954 | 16.99 | 0.51 | 0.05 | 1 | 0.98 | 1:157450062-157450954 | 17.22 | 1 |
| Line6 | 1 | 157698899 | 157699774 | 12.34 | 1 | 1 | 1 | 1 | 1:157698899-157699774 | 17.03 | 1 |
| Line6 | 1 | 162222519 | 162223183 | 5.78 | 1 | 1 | 1 | 1 | 1:162222519-162223183 | 14.55 | 1 |
| Line6 | 1 | 164415036 | 164415163 | 6.12 | 0.49 | 0.05 | 1 | 0.94 | 1:164414998-164416037 | 18.09 | 1 |
| Line6 | 1 | 164415944 | 164416063 | 2.61 | 0.36 | 0 | 0 | 0.83 | 1:164414998-164416037 | 18.09 | 1 |
| Line6 | 1 | 180746939 | 180747260 | 5.82 | 0.51 | 0.05 | 1 | 0.99 | 1:180746896-180747970 | 16.07 | 1 |
| Line6 | 1 | 180747803 | 180747949 | 4.16 | 0.51 | 0.05 | 1 | 0.99 | 1:180746896-180747970 | 16.07 | 1 |
| Line6 | 1 | 182832918 | 182834042 | 17.81 | 0.98 | 0.97 | 1 | 0.98 | 1:182832847-182834082 | 25.04 | 1 |
| Line6 | 1 | 184347922 | 184348767 | 17.64 | 0.99 | 1 | 1 | 0.98 | 1:184347893-184348804 | 26.35 | 1 |
| Line6 | 1 | 185856831 | 185857467 | 12.88 | 0.51 | 0.05 | 1 | 0.98 | 1:185856831-185857511 | 16.89 | 1 |
| Line6 | 2 | 3491397 | 3492240 | 12.11 | 1 | 1 | 1 | 1 | 2:3491394-3492279 | 19.37 | 1 |
| Line6 | 2 | 10512508 | 10512851 | 6.98 | 0.51 | 0.05 | 1 | 0.99 | 2:10512425-10512887 | 16.73 | 1 |
| Line6 | 2 | 20971784 | 20972407 | 3.78 | 0.62 | 0.27 | 1 | 0.98 | 2:20971534-20972437 | 13 | 1 |
| Line6 | 2 | 51801080 | 51801932 | 15.23 | 0.99 | 0.99 | 1 | 0.99 | 2:51801055-51801954 | 18.45 | 1 |
| Line6 | 2 | 53111773 | 53111992 | 3.22 | 0.99 | 0.99 | 1 | 0.98 | 2:53111756-53112017 | 14.37 | 1 |
| Line6 | 2 | 80333619 | 80334273 | 5.72 | 0.51 | 0.05 | 1 | 0.99 | 2:80333585-80334273 | 10.84 | 0.99 |
| Line6 | 2 | 82574335 | 82575450 | 8.87 | 0.99 | 1 | 1 | 0.98 | 2:82574297-82575518 | 23.2 | 1 |
| Line6 | 2 | 92857028 | 92858147 | 4.71 | 0.99 | 0.99 | 1 | 0.98 | 2:92856996-92858219 | 12.18 | 1 |
| Line6 | 2 | 95480970 | 95481836 | 8.26 | 1 | 1 | 1 | 0.99 | 2:95480957-95481836 | 11.57 | 1 |
| Line6 | 2 | 106764059 | 106764678 | 15.42 | 0.52 | 0.05 | 1 | 1 | 2:106764059-106764678 | 18.25 | 1 |
| Line6 | 2 | 124765081 | 124765703 | 8.71 | 0.51 | 0.05 | 1 | 0.98 | 2:124765081-124765718 | 15.99 | 1 |
| Line6 | 2 | 131563769 | 131564115 | 11.84 | 0.51 | 0.05 | 1 | 0.98 | 2:131563757-131567794 | 23.32 | 1 |
| Line6 | 2 | 131567597 | 131567760 | 6.4 | 0.52 | 0.05 | 1 | 1 | 2:131563757-131567794 | 23.32 | 1 |
| Line6 | 2 | 143463257 | 143463631 | 8.17 | 0.51 | 0.05 | 1 | 0.98 | 2:143463257-143468861 | 17.5 | 0.93 |
| Line6 | 2 | 143468707 | 143468861 | 4.92 | 0.52 | 0.06 | 1 | 0.98 | 2:143463257-143468861 | 17.5 | 0.93 |
| Line6 | 3 | 19822153 | 19822916 | 5.23 | 0.46 | 0.91 | 0 | 0 | 3:19821802-19823007 | 7.56 | 0.96 |
| Line6 | 3 | 49572068 | 49572921 | 13.76 | 1 | 1 | 1 | 1 | 3:49572068-49572931 | 17.51 | 1 |
| Line6 | 3 | 54250336 | 54250466 | 2.98 | 0.51 | 0.05 | 1 | 0.98 | 3:54250109-54254158 | 21.43 | 1 |
| Line6 | 3 | 54253926 | 54254092 | 5.82 | 0.52 | 0.05 | 1 | 1 | 3:54250109-54254158 | 21.43 | 1 |
| Line6 | 3 | 63745322 | 63745883 | 7.97 | 0.52 | 0.05 | 1 | 1 | 3:63745322-63745967 | 20.16 | 1 |
| Line6 | 3 | 73562038 | 73562816 | 4.55 | 0.99 | 0.99 | 1 | 0.98 | 3:73561879-73563101 | 13.85 | 1 |
| Line6 | 3 | 83407685 | 83408482 | 7.81 | 1 | 1 | 1 | 1 | 3:83407619-83408514 | 12.31 | 0.83 |
| Line6 | 3 | 91661280 | 91662061 | 11.51 | 1 | 1 | 1 | 1 | 3:91661280-91662061 | 19.71 | 1 |
| Line6 | 3 | 96957534 | 96957908 | 8.06 | 0.51 | 0.05 | 1 | 0.98 | 3:96957515-96961612 | 23.41 | 1 |
| Line6 | 3 | 96961376 | 96961600 | 10.64 | 0.52 | 0.05 | 1 | 1 | 3:96957515-96961612 | 23.41 | 1 |
| Line6 | 4 | 11124012 | 11124392 | 5.61 | 0.99 | 1 | 1 | 0.98 | 4:11123901-11124467 | 27.19 | 1 |
| Line6 | 4 | 30595682 | 30596244 | 3.04 | 0.93 | 0.99 | 1 | 0.85 | 4:30595679-30596557 | 16.94 | 1 |
| Line6 | 4 | 40212369 | 40212953 | 9.15 | 0.52 | 0.05 | 1 | 1 | 4:40212346-40213006 | 11.45 | 1 |
| Line6 | 4 | 70552141 | 70552668 | 4.75 | 0.99 | 1 | 1 | 0.98 | 4:70551829-70552669 | 10.1 | 0.91 |
| Line6 | 4 | 78859384 | 78859987 | 9.08 | 0.51 | 0.05 | 1 | 0.98 | 4:78859384-78859987 | 15.16 | 1 |
| Line6 | 5 | 2705103 | 2706194 | 9.1 | 0.69 | 0.56 | 1 | 0.81 | 5:2705052-2706284 | 23.19 | 1 |
| Line6 | 5 | 6164277 | 6165449 | 16.19 | 0.95 | 1 | 1 | 0.88 | 5:6164269-6165523 | 26.77 | 1 |
| Line6 | 5 | 31303941 | 31304752 | 4.5 | 0.8 | 0.6 | 1 | 1 | 5:31303866-31305068 | 17.2 | 1 |
| Line6 | 5 | 31525371 | 31526243 | 12.69 | 1 | 1 | 1 | 1 | 5:31525368-31526271 | 14.22 | 1 |
| Line6 | 5 | 31898695 | 31899843 | 5.69 | 0.6 | 0.64 | 1 | 0.5 | 5:31898626-31899883 | 16 | 1 |
| Line6 | 6 | 9811211 | 9812250 | 4.53 | 0.97 | 0.93 | 1 | 1 | 6:9811191-9816444 | 21.53 | 0.99 |
| Line6 | 6 | 9815941 | 9816384 | 7.68 | 0.92 | 0.86 | 1 | 0.98 | 6:9811191-9816444 | 21.53 | 0.99 |
| Line6 | 6 | 13724878 | 13725890 | 1.34 | 0.41 | 0.82 | 0 | 0 | 6:13724843-13726022 | 20.08 | 1 |
| Line6 | 6 | 26190297 | 26191167 | 6.85 | 1 | 1 | 1 | 1 | 6:26190297-26191167 | 17.77 | 1 |
| Line6 | 7 | 17708120 | 17708728 | 6.79 | 0.43 | 0 | 0.76 | 0.88 | 7:17708068-17708753 | 24.51 | 1 |
| Line6 | 8 | 9208415 | 9209246 | 2.32 | 0.85 | 0.99 | 0 | 0.81 | 8:9208394-9209273 | 5.37 | 0.95 |
| Line6 | 12 | 3240730 | 3240844 | 4.59 | 0.79 | 0.99 | 1 | 0.52 | 12:3240697-3240898 | 90.16 | 1 |
| Line6 | 12 | 3277815 | 3278289 | 56.2 | 1 | 1 | 1 | 0.99 | 12:3277416-3278316 | 99.52 | 1 |
| Line6 | 17 | 180661 | 181479 | 12.18 | 0.97 | 0.99 | 1 | 0.94 | 17:180636-181570 | 23.09 | 1 |
| Line6 | 20 | 2428778 | 2429650 | 13.42 | 0.95 | 1 | 1 | 0.88 | 20:2428746-2429684 | 16.76 | 1 |
| Line6 | 20 | 7662092 | 7662742 | 5.37 | 1 | 1 | 1 | 1 | 20:7661897-7662786 | 15.07 | 1 |
| Line6 | AADN03009901.1 | 864 | 1161 | 4.33 | 0.49 | 0.05 | 1 | 0.93 | AADN03009901.1:851-1161 | 18.87 | 1 |
| Line6 | AADN03018735.1 | 536 | 1203 | 11.81 | 0.99 | 0.99 | 1 | 0.98 | AADN03018735.1:281-1293 | 18.99 | 1 |
| Line6 | JH375212.1 | 37372 | 37495 | 3.01 | 0.45 | 0.05 | 1 | 0.84 | JH375212.1:37241-37702 | 6.01 | 1 |
| Line6 | JH375231.1 | 5493 | 5760 | 3.31 | 0.72 | 0.55 | 1 | 0.87 | JH375231.1:4846-8915 | 9.94 | 1 |
| Line6 | JH375231.1 | 8068 | 8925 | 2.73 | 0.62 | 0.25 | 1 | 1 | JH375231.1:4846-8915 | 9.94 | 1 |
| Line6 | JH376310.1 | 6595 | 7035 | 3.98 | 0.97 | 0.99 | 0.71 | 0.98 | JH376310.1:6513-7054 | 5.67 | 1 |
| Line6 | Z | 30997118 | 30997223 | 4.27 | 0.45 | 0 | 0.24 | 1 | Z:30996146-31000811 | 15.75 | 1 |
| Line6 | Z | 32081261 | 32081593 | 4.38 | 0.93 | 1 | 1 | 0.85 | Z:32081154-32086293 | 29.23 | 1 |
| Line6 | Z | 32085834 | 32086235 | 7.45 | 1 | 1 | 1 | 0.99 | Z:32081154-32086293 | 29.23 | 1 |
| Line6 | Z | 61304884 | 61305974 | 8.19 | 0.51 | 0.05 | 1 | 0.98 | Z:61304819-61306021 | 9.21 | 1 |
| Line6 | Z | 79945559 | 79946410 | 5.41 | 0.99 | 0.99 | 1 | 1 | Z:79945501-79946432 | 9.12 | 1 |
| Line7 | 1 | 31728984 | 31729527 | 6.6 | 0.51 | 0.05 | 1 | 0.98 | 1:31728916-31729579 | 18 | 1 |
| Line7 | 1 | 32312255 | 32313054 | 11.78 | 0.99 | 1 | 1 | 0.98 | 1:32312218-32313185 | 13.15 | 1 |
| Line7 | 1 | 42541987 | 42542536 | 13.32 | 0.51 | 0.05 | 1 | 0.98 | 1:42541895-42542614 | 17.72 | 1 |
| Line7 | 1 | 63571458 | 63572040 | 5.8 | 0.52 | 0.05 | 1 | 1 | 1:63571454-63572079 | 16.35 | 1 |
| Line7 | 1 | 67550534 | 67551055 | 16.06 | 0.98 | 1 | 1 | 0.94 | 1:67550436-67551166 | 36.42 | 1 |
| Line7 | 1 | 96182429 | 96182609 | 3.47 | 0.52 | 0.05 | 1 | 1 | 1:96182372-96182610 | 16.06 | 1 |
| Line7 | 1 | 99050590 | 99050975 | 6.95 | 0.5 | 0.05 | 1 | 0.96 | 1:99050515-99051202 | 16.66 | 1 |
| Line7 | 1 | 101165422 | 101166193 | 11.72 | 1 | 1 | 1 | 1 | 1:101165329-101166193 | 17.77 | 1 |
| Line7 | 1 | 126794637 | 126795419 | 11.74 | 0.98 | 1 | 1 | 0.96 | 1:126794615-126795484 | 15.29 | 1 |
| Line7 | 1 | 140710414 | 140710798 | 8.23 | 1 | 1 | 1 | 0.99 | 1:140710345-140715423 | 39.61 | 1 |
| Line7 | 1 | 140714957 | 140715281 | 14.11 | 1 | 1 | 1 | 1 | 1:140710345-140715423 | 39.61 | 1 |
| Line7 | 1 | 157450105 | 157450904 | 13.12 | 0.51 | 0.05 | 1 | 0.98 | 1:157450062-157450954 | 18.92 | 1 |
| Line7 | 1 | 160455452 | 160455608 | 5.53 | 0.51 | 0.05 | 1 | 0.98 | 1:160455218-160455708 | 13.52 | 1 |
| Line7 | 1 | 160463945 | 160464198 | 24.62 | 0.52 | 0.05 | 1 | 1 | 1:160463921-160464245 | 24.3 | 1 |
| Line7 | 1 | 163694811 | 163695236 | 7.36 | 0.52 | 0.05 | 1 | 1 | 1:163694748-163695372 | 10.83 | 0.97 |
| Line7 | 1 | 164415029 | 164415873 | 6.34 | 0.5 | 0.05 | 1 | 0.96 | 1:164414998-164416037 | 24.21 | 1 |
| Line7 | 1 | 182832913 | 182834008 | 12.63 | 0.99 | 0.99 | 1 | 0.98 | 1:182832847-182834082 | 21.16 | 0.99 |
| Line7 | 2 | 51801063 | 51801875 | 16.53 | 0.99 | 0.99 | 1 | 1 | 2:51801055-51801954 | 20.95 | 1 |
| Line7 | 2 | 53111815 | 53112016 | 3.72 | 0.85 | 0.81 | 1 | 0.88 | 2:53111756-53112017 | 18.8 | 1 |
| Line7 | 2 | 80333951 | 80334203 | 5.1 | 0.51 | 0.05 | 1 | 0.98 | 2:80333585-80334273 | 14.56 | 0.99 |
| Line7 | 2 | 82574493 | 82575474 | 9.26 | 0.9 | 0.99 | 1 | 0.77 | 2:82574297-82575518 | 18.54 | 1 |
| Line7 | 2 | 92857031 | 92858110 | 11.05 | 0.76 | 0.99 | 1 | 0.45 | 2:92856996-92858219 | 17.21 | 1 |
| Line7 | 2 | 124765514 | 124765703 | 6.41 | 0.52 | 0.05 | 1 | 1 | 2:124765081-124765718 | 14.57 | 1 |
| Line7 | 2 | 131563802 | 131564124 | 11.99 | 0.51 | 0.05 | 1 | 0.98 | 2:131563757-131567794 | 32.73 | 1 |
| Line7 | 2 | 131567489 | 131567741 | 12.64 | 0.52 | 0.05 | 1 | 1 | 2:131563757-131567794 | 32.73 | 1 |
| Line7 | 2 | 143468622 | 143468826 | 9.49 | 0.51 | 0.05 | 1 | 0.98 | 2:143463257-143468861 | 20.74 | 0.95 |
| Line7 | 3 | 19822193 | 19822981 | 9.45 | 0.44 | 0.87 | 0 | 0 | 3:19821802-19823007 | 12.96 | 1 |
| Line7 | 3 | 34783007 | 34783328 | 9.29 | 0.51 | 0.05 | 1 | 0.98 | 3:34782953-34783379 | 31.89 | 1 |
| Line7 | 3 | 54253895 | 54254129 | 5.19 | 0.52 | 0.05 | 1 | 1 | 3:54250109-54254158 | 26.59 | 1 |
| Line7 | 3 | 68891616 | 68892096 | 4.86 | 0.51 | 0.05 | 1 | 0.98 | 3:68891616-68892157 | 12 | 1 |
| Line7 | 3 | 70604586 | 70605369 | 9.85 | 1 | 1 | 1 | 0.99 | 3:70604555-70605387 | 12.88 | 0.93 |
| Line7 | 3 | 73562004 | 73563035 | NA | NA | NA | NA | NA | 3:73561879-73563101 | 18.48 | 1 |
| Line7 | 3 | 83407688 | 83408503 | 5.07 | 1 | 1 | 1 | 1 | 3:83407619-83408514 | 11.62 | 0.83 |
| Line7 | 3 | 96957582 | 96957900 | 9.29 | 0.51 | 0.05 | 1 | 0.98 | 3:96957515-96961612 | 28.03 | 1 |
| Line7 | 3 | 96961376 | 96961553 | 4.59 | 0.52 | 0.05 | 1 | 1 | 3:96957515-96961612 | 28.03 | 1 |
| Line7 | 4 | 20401593 | 20402414 | 16.24 | 1 | 1 | 1 | 1 | 4:20401588-20402427 | 16.5 | 0.99 |
| Line7 | 4 | 30595770 | 30596557 | 16.7 | 1 | 1 | 1 | 1 | 4:30595679-30596557 | 20.16 | 1 |
| Line7 | 4 | 40212589 | 40213006 | 8.45 | 0.52 | 0.05 | 1 | 1 | 4:40212346-40213006 | 16.23 | 1 |
| Line7 | 4 | 70463849 | 70464210 | 9.03 | 0.52 | 0.05 | 1 | 1 | 4:70463742-70464439 | 22.12 | 1 |
| Line7 | 4 | 72515182 | 72515626 | 9.53 | 0.51 | 0.05 | 1 | 0.98 | 4:72515182-72515626 | 13.08 | 1 |
| Line7 | 4 | 78859381 | 78859756 | 7.18 | 0.51 | 0.05 | 1 | 0.98 | 4:78859384-78859987 | 13.13 | 1 |
| Line7 | 4 | 86220927 | 86221746 | 13.52 | 0.99 | 0.99 | 1 | 1 | 4:86220862-86221807 | 14.67 | 0.91 |
| Line7 | 5 | 2705323 | 2706094 | 6.92 | 0.95 | 0.92 | 1 | 0.98 | 5:2705052-2706284 | 28 | 1 |
| Line7 | 5 | 6164324 | 6165450 | 10.65 | 0.89 | 0.97 | 1 | 0.77 | 5:6164269-6165523 | 24.92 | 1 |
| Line7 | 5 | 19302240 | 19303048 | 15.78 | 1 | 1 | 1 | 1 | 5:19302240-19303103 | 21.54 | 1 |
| Line7 | 5 | 31303979 | 31304921 | 1.62 | 0.55 | 0.72 | 0 | 0.42 | 5:31303866-31305068 | 17.28 | 1 |
| Line7 | 5 | 31898771 | 31899787 | 4.69 | 0.96 | 0.93 | 1 | 0.98 | 5:31898626-31899883 | 19 | 1 |
| Line7 | 6 | 9811269 | 9812243 | 8.02 | 1 | 0.99 | 1 | 1 | 6:9811191-9816444 | 23.53 | 0.99 |
| Line7 | 6 | 9815957 | 9816406 | 11.42 | 0.99 | 0.99 | 1 | 0.98 | 6:9811191-9816444 | 23.53 | 0.99 |
| Line7 | 6 | 13725108 | 13725897 | 4.81 | 0.97 | 0.96 | 1 | 0.98 | 6:13724843-13726022 | 19.41 | 1 |
| Line7 | 6 | 26190349 | 26191157 | 10.93 | 1 | 1 | 1 | 1 | 6:26190297-26191167 | 20.73 | 1 |
| Line7 | 7 | 17708145 | 17708700 | 17.58 | 0.5 | 0.04 | 1 | 0.98 | 7:17708068-17708753 | 22.81 | 1 |
| Line7 | 8 | 9208424 | 9208981 | 2.6 | 0.87 | 1 | 1 | 0.7 | 8:9208394-9209273 | 10.58 | 1 |
| Line7 | 11 | 18978682 | 18979268 | 12.87 | 0.52 | 0.05 | 1 | 1 | 11:18978682-18979268 | 19.4 | 1 |
| Line7 | 12 | 3240720 | 3240844 | 2.34 | 0.48 | 0.94 | 0 | 0 | 12:3240697-3240898 | 84.96 | 1 |
| Line7 | 12 | 3278186 | 3278290 | 32.28 | 1 | 1 | 1 | 0.99 | 12:3277416-3278316 | 112.23 | 1 |
| Line7 | 17 | 180708 | 181468 | 13.86 | 0.99 | 0.99 | 1 | 0.98 | 17:180636-181570 | 23.04 | 1 |
| Line7 | AADN03024906.1 | 3434 | 4113 | 2.55 | 0.38 | 0.05 | 1 | 0.69 | AADN03024906.1:3470-4351 | 12.74 | 1 |
| Line7 | JH375212.1 | 37368 | 37640 | 6.07 | 0.51 | 0.05 | 1 | 0.98 | JH375212.1:37241-37702 | 14.55 | 1 |
| Line7 | JH375231.1 | 5479 | 5751 | 8 | 0.72 | 0.55 | 1 | 0.87 | JH375231.1:4846-8915 | 10.63 | 1 |
| Line7 | JH375231.1 | 8540 | 8903 | 4.37 | 0.77 | 0.55 | 1 | 1 | JH375231.1:4846-8915 | 10.63 | 1 |
| Line7 | JH376310.1 | 6612 | 7034 | 12.66 | 0.99 | 0.99 | 1 | 0.98 | JH376310.1:6513-7054 | 14.09 | 1 |
| Line7 | Z | 30996779 | 30996881 | 5.98 | 1 | 1 | 1 | 1 | Z:30996146-31000811 | 26.85 | 1 |
| Line7 | Z | 32081202 | 32081601 | 5.16 | 1 | 1 | 1 | 1 | Z:32081154-32086293 | 28.46 | 1 |
| Line7 | Z | 32085825 | 32086245 | 20.07 | 0.99 | 1 | 1 | 0.98 | Z:32081154-32086293 | 28.46 | 1 |
| Line7 | Z | 61304945 | 61306000 | 25.44 | 0.99 | 1 | 1 | 0.98 | Z:61304819-61306021 | 13.6 | 1 |
| Line7 | Z | 79945577 | 79946317 | 7.43 | 1 | 1 | 1 | 1 | Z:79945501-79946432 | 12.21 | 1 |
| LineC | 1 | 101333520 | 101334353 | 11.47 | 0.97 | 1 | 1 | 0.93 | 1:101333491-101334404 | 16.83 | 1 |
| LineC | 1 | 113945700 | 113946141 | 6.47 | 0.36 | 0 | 0 | 0.83 | 1:113945460-113946362 | 19.14 | 1 |
| LineC | 1 | 127188644 | 127189303 | 4.13 | 0.99 | 1 | 1 | 0.98 | 1:127188644-127189303 | 18.26 | 1 |
| LineC | 1 | 140710477 | 140710835 | 8.79 | 1 | 1 | 1 | 0.99 | 1:140710345-140715423 | 23.95 | 1 |
| LineC | 1 | 140714989 | 140715397 | 8.22 | 0.82 | 1 | 1 | 0.58 | 1:140710345-140715423 | 23.95 | 1 |
| LineC | 1 | 146731910 | 146732801 | 8.4 | 0.99 | 1 | 1 | 0.98 | 1:146731910-146732833 | 17.7 | 1 |
| LineC | 1 | 160455266 | 160455554 | 7.38 | 0.51 | 0.05 | 1 | 0.98 | 1:160455218-160455708 | 13.85 | 1 |
| LineC | 1 | 160464092 | 160464228 | 5.46 | 0.49 | 0.05 | 1 | 0.94 | 1:160463921-160464245 | 12.76 | 1 |
| LineC | 1 | 164415013 | 164415157 | 3.89 | 0.49 | 0.01 | 1 | 0.98 | 1:164414998-164416037 | 21.51 | 1 |
| LineC | 1 | 164415675 | 164416019 | 9.25 | 0.51 | 0.05 | 1 | 0.98 | 1:164414998-164416037 | 21.51 | 1 |
| LineC | 1 | 180746946 | 180747913 | 2.1 | 0.47 | 0.05 | 1 | 0.9 | 1:180746896-180747970 | 15.68 | 1 |
| LineC | 1 | 182832847 | 182833971 | 13.79 | 0.97 | 0.99 | 1 | 0.93 | 1:182832847-182834082 | 21.29 | 1 |
| LineC | 1 | 184347935 | 184348794 | 7.34 | 0.99 | 1 | 1 | 0.98 | 1:184347893-184348804 | 23.36 | 1 |
| LineC | 1 | 187342002 | 187342148 | 5.45 | 0.51 | 0.05 | 1 | 0.98 | 1:187340668-187342356 | 13.58 | 1 |
| LineC | 12 | 3240723 | 3240882 | 8.83 | 0.94 | 1 | 1 | 0.85 | 12:3240697-3240898 | 76.41 | 1 |
| LineC | 12 | 3277800 | 3278290 | 52.13 | 1 | 1 | 1 | 0.99 | 12:3277416-3278316 | 102.07 | 1 |
| LineC | 1 | 32312244 | 32313016 | 8.46 | 0.99 | 1 | 1 | 0.98 | 1:32312218-32313185 | 13.03 | 1 |
| LineC | 13 | 6876434 | 6877337 | 12.6 | 0.96 | 0.99 | 1 | 0.93 | 13:6876434-6877337 | 17.89 | 1 |
| LineC | 1 | 41318928 | 41319767 | 12.6 | 1 | 1 | 1 | 0.99 | 1:41318900-41319777 | 14.86 | 1 |
| LineC | 1 | 43882636 | 43883815 | 5.65 | 0.5 | 0.58 | 1 | 0.33 | 1:43882636-43883815 | 16.67 | 1 |
| LineC | 1 | 67550436 | 67551052 | 14.28 | 1 | 1 | 1 | 1 | 1:67550436-67551166 | 29.64 | 1 |
| LineC | 1 | 96182413 | 96182610 | 2.91 | 0.52 | 0.05 | 1 | 1 | 1:96182372-96182610 | 14.62 | 1 |
| LineC | 1 | 99050515 | 99051185 | 10.18 | 0.52 | 0.05 | 1 | 1 | 1:99050515-99051202 | 13.84 | 1 |
| LineC | 20 | 2428754 | 2429684 | 14 | 0.97 | 1 | 1 | 0.93 | 20:2428746-2429684 | 19.07 | 1 |
| LineC | 2 | 10512508 | 10512887 | 10.22 | 0.51 | 0.05 | 1 | 0.99 | 2:10512425-10512887 | 22.6 | 1 |
| LineC | 2 | 131563757 | 131563908 | 6.16 | 0.51 | 0.05 | 1 | 0.98 | 2:131563757-131567794 | 21.18 | 1 |
| LineC | 2 | 131567614 | 131567782 | 4.55 | 0.52 | 0.05 | 1 | 1 | 2:131563757-131567794 | 21.18 | 1 |
| LineC | 2 | 20971534 | 20972219 | 10.61 | 0.62 | 0.27 | 1 | 0.98 | 2:20971534-20972437 | 16.76 | 1 |
| LineC | 2 | 34296518 | 34297385 | 14.95 | 1 | 1 | 1 | 1 | 2:34296518-34297385 | 23.83 | 1 |
| LineC | 2 | 81965228 | 81965359 | 4.22 | 0.51 | 0.05 | 1 | 0.99 | 2:81965001-81966072 | 15.24 | 1 |
| LineC | 2 | 82574319 | 82575495 | 11.04 | 0.79 | 0.93 | 1 | 0.6 | 2:82574297-82575518 | 16.36 | 1 |
| LineC | 2 | 92857023 | 92858219 | 2.68 | 0.52 | 0.73 | 1 | 0.21 | 2:92856996-92858219 | 14.74 | 1 |
| LineC | 2 | 95481141 | 95481827 | 10.28 | 1 | 1 | 1 | 0.99 | 2:95480957-95481836 | 15.92 | 1 |
| LineC | 3 | 19821814 | 19822951 | 3.03 | 0.46 | 0.9 | 0 | 0 | 3:19821802-19823007 | 11.36 | 1 |
| LineC | 3 | 34783012 | 34783371 | 5.89 | 0.51 | 0.05 | 1 | 0.98 | 3:34782953-34783379 | 30.24 | 1 |
| LineC | 3 | 73561920 | 73562995 | 7.95 | 0.44 | 0.66 | 1 | 0.1 | 3:73561879-73563101 | 14.98 | 1 |
| LineC | 3 | 83407619 | 83408475 | 7.22 | 1 | 1 | 1 | 1 | 3:83407619-83408514 | 11.03 | 0.83 |
| LineC | 3 | 86212524 | 86213394 | 7.44 | 0.98 | 1 | 1 | 0.96 | 3:86212524-86213394 | 13.7 | 1 |
| LineC | 3 | 93697686 | 93698431 | 7.56 | 0.99 | 1 | 1 | 0.98 | 3:93697657-93698572 | 15.45 | 1 |
| LineC | 3 | 96957541 | 96957902 | 7.98 | 0.51 | 0.05 | 1 | 0.98 | 3:96957515-96961612 | 18.83 | 1 |
| LineC | 3 | 96961380 | 96961612 | 5.35 | 0.52 | 0.05 | 1 | 1 | 3:96957515-96961612 | 18.83 | 1 |
| LineC | 3 | 99426024 | 99426597 | 8.91 | 0.52 | 0.05 | 1 | 1 | 3:99426024-99426621 | 20.65 | 1 |
| LineC | 4 | 11123994 | 11124467 | 9.08 | 0.99 | 1 | 1 | 0.98 | 4:11123901-11124467 | 19.8 | 1 |
| LineC | 4 | 26530896 | 26531216 | 9.85 | 0.51 | 0.05 | 1 | 0.98 | 4:26530893-26531990 | 20.48 | 1 |
| LineC | 4 | 29234085 | 29234423 | 8.44 | 0.52 | 0.05 | 1 | 1 | 4:29234085-29234741 | 20.86 | 1 |
| LineC | 4 | 70552101 | 70552653 | 8.76 | 0.97 | 1 | 1 | 0.93 | 4:70551829-70552669 | 12.08 | 0.84 |
| LineC | 4 | 86220870 | 86221738 | 11.29 | 1 | 1 | 1 | 1 | 4:86220862-86221807 | 15.65 | 0.92 |
| LineC | 5 | 2705091 | 2706225 | 5.24 | 0.79 | 0.73 | 1 | 0.85 | 5:2705052-2706284 | 18.7 | 1 |
| LineC | 5 | 31303989 | 31304978 | 2.68 | 0.35 | 0 | 0 | 0.81 | 5:31303866-31305068 | 12.98 | 1 |
| LineC | 5 | 31898720 | 31899716 | 8.46 | 0.79 | 0.99 | 1 | 0.52 | 5:31898626-31899883 | 18.8 | 1 |
| LineC | 6 | 13724857 | 13725893 | 1.45 | 0.61 | 0.58 | 0 | 0.73 | 6:13724843-13726022 | 14.64 | 1 |
| LineC | 6 | 25237263 | 25237909 | 7.56 | 0.52 | 0.05 | 1 | 1 | 6:25237263-25237909 | 17.1 | 1 |
| LineC | 6 | 9811227 | 9811670 | 11.96 | 0.99 | 0.99 | 1 | 1 | 6:9811191-9816444 | 18.58 | 0.99 |
| LineC | 6 | 9815942 | 9816430 | 11.53 | 0.99 | 0.99 | 1 | 0.98 | 6:9811191-9816444 | 18.58 | 0.99 |
| LineC | 7 | 29830071 | 29830657 | 6.23 | 0.51 | 0.05 | 1 | 0.98 | 7:29830070-29830722 | 19.67 | 1 |
| LineC | AADN03016003.1 | 257 | 448 | 5.1 | 0.75 | 1 | 1 | 0.42 | AADN03016003.1:257-448 | 713.43 | 1 |
| LineC | AADN03024630.1 | 317 | 887 | 10.33 | 1 | 1 | 1 | 1 | AADN03024630.1:317-887 | NA |  |
| LineC | AADN03024906.1 | 4213 | 4362 | 2.43 | 0.51 | 0.04 | 1 | 0.98 | AADN03024906.1:3470-4351 | 14.02 | 1 |
| LineC | JH375212.1 | 37650 | 37755 | 3.67 | 0.37 | 0 | 0 | 0.85 | JH375212.1:37241-37702 | 11.44 | 1 |
| LineC | JH375231.1 | 5466 | 5587 | 5.02 | 0.72 | 0.55 | 1 | 0.87 | JH375231.1:4846-8915 | 9.03 | 1 |
| LineC | JH376310.1 | 6633 | 7025 | 8.05 | 0.99 | 0.99 | 1 | 0.98 | JH376310.1:6513-7054 | 15.87 | 1 |
| LineC | Z | 15471143 | 15471365 | 2.57 | 0.71 | 1 | 1 | 0.32 | Z:15470956-15471752 | 6.32 | 1 |
| LineC | Z | 30996786 | 30997220 | 7.59 | 1 | 1 | 1 | 1 | Z:30996146-31000811 | 19.7 | 0.99 |
| LineC | Z | 32081221 | 32081601 | 6.56 | 1 | 1 | 1 | 1 | Z:32081154-32086293 | 23.39 | 1 |
| LineC | Z | 32085859 | 32086178 | 5.24 | 0.99 | 1 | 1 | 0.98 | Z:32081154-32086293 | 23.39 | 1 |
| LineC | Z | 61304959 | 61305694 | 14.93 | 0.98 | 0.98 | 1 | 0.98 | Z:61304819-61306021 | 12.31 | 1 |
| LineC | Z | 79945501 | 79946410 | 9.22 | 1 | 1 | 1 | 1 | Z:79945501-79946432 | 11.89 | 1 |
| LineN | 1 | 126794782 | 126795399 | 2.68 | 0.94 | 0.89 | 1 | 0.98 | 1:126794615-126795484 | 11.02 | 1 |
| LineN | 1 | 140710594 | 140710834 | 5.93 | 1 | 1 | 1 | 0.99 | 1:140710345-140715423 | 15.54 | 0.99 |
| LineN | 1 | 140714967 | 140715332 | 6.32 | 1 | 1 | 1 | 1 | 1:140710345-140715423 | 15.54 | 0.99 |
| LineN | 1 | 146732050 | 146732698 | 4.68 | 0.99 | 1 | 1 | 0.98 | 1:146731910-146732833 | 9.11 | 0.98 |
| LineN | 1 | 149935206 | 149935623 | 3.84 | 0.51 | 0.05 | 1 | 0.99 | 1:149935105-149935762 | 11.83 | 1 |
| LineN | 1 | 157450231 | 157450765 | 1.86 | 0.47 | 0 | 0.82 | 0.98 | 1:157450062-157450954 | 9.06 | 1 |
| LineN | 1 | 184348006 | 184348711 | 2.91 | 0.96 | 0.93 | 1 | 0.98 | 1:184347893-184348804 | 10.99 | 1 |
| LineN | 13 | 10909882 | 10910211 | 2.14 | 0.89 | 1 | 1 | 0.74 | 13:10909806-10910958 | 10.03 | 1 |
| LineN | 1 | 32312291 | 32312959 | 8.27 | 0.99 | 1 | 1 | 0.98 | 1:32312218-32313185 | 11.1 | 1 |
| LineN | 13 | 6484845 | 6485499 | 4.62 | 0.99 | 1 | 1 | 0.98 | 13:6484735-6485598 | 9.42 | 1 |
| LineN | 13 | 6876605 | 6877194 | 4.73 | 0.99 | 0.99 | 1 | 0.98 | 13:6876434-6877337 | 9.75 | 1 |
| LineN | 1 | 41318982 | 41319659 | 7.58 | 0.99 | 0.99 | 1 | 0.98 | 1:41318900-41319777 | 9.84 | 1 |
| LineN | 1 | 43882737 | 43883751 | 3.01 | 0.47 | 0.57 | 1 | 0.27 | 1:43882636-43883815 | 11.51 | 1 |
| LineN | 17 | 180732 | 181452 | 5.62 | 0.93 | 0.97 | 1 | 0.88 | 17:180636-181570 | 11.18 | 1 |
| LineN | 20 | 7661992 | 7662674 | 2.86 | 1 | 1 | 1 | 1 | 20:7661897-7662786 | 10.86 | 1 |
| LineN | 2 | 10512573 | 10512821 | 5.04 | 0.51 | 0.05 | 1 | 0.99 | 2:10512425-10512887 | 12.16 | 1 |
| LineN | 2 | 124765221 | 124765563 | 7.41 | 0.51 | 0.05 | 1 | 0.98 | 2:124765081-124765718 | 10.04 | 1 |
| LineN | 2 | 131563830 | 131564229 | 8.35 | 0.51 | 0.05 | 1 | 0.99 | 2:131563757-131567794 | 14.03 | 1 |
| LineN | 2 | 131567496 | 131567638 | 5.33 | 0.42 | 0 | 0 | 0.97 | 2:131563757-131567794 | 14.03 | 1 |
| LineN | 2 | 20971708 | 20972295 | 4.6 | 0.62 | 0.27 | 1 | 0.98 | 2:20971534-20972437 | 10.28 | 1 |
| LineN | 2 | 47663562 | 47663887 | 4.97 | 0.52 | 0.05 | 1 | 1 | 2:47663562-47663887 | 6.94 | 1 |
| LineN | 2 | 51801166 | 51801833 | 7.49 | 0.98 | 0.95 | 1 | 1 | 2:51801055-51801954 | 12.94 | 1 |
| LineN | 2 | 5464380 | 5464705 | 3.47 | 0.87 | 1 | 1 | 0.69 | 2:5464380-5464705 | 8.82 | 1 |
| LineN | 2 | 82574393 | 82575363 | 7.79 | 0.89 | 1 | 1 | 0.74 | 2:82574297-82575518 | 14.13 | 1 |
| LineN | 2 | 92071297 | 92071568 | 1.27 | 0.77 | 0.82 | 0 | 0.81 | 2:92070857-92071755 | 12.84 | 1 |
| LineN | 2 | 92857262 | 92858148 | 4.94 | 0.97 | 0.95 | 1 | 0.98 | 2:92856996-92858219 | 8.28 | 1 |
| LineN | 2 | 95481247 | 95481522 | 1.22 | 0.58 | 0.88 | 1 | 0.16 | 2:95480957-95481836 | 8.05 | 1 |
| LineN | 3 | 19821886 | 19822879 | 1.75 | 0.77 | 0.92 | 0 | 0.69 | 3:19821802-19823007 | 9.69 | 1 |
| LineN | 3 | 34783134 | 34783271 | 4.95 | 0.51 | 0.05 | 1 | 0.98 | 3:34782953-34783379 | 19.32 | 1 |
| LineN | 3 | 36156427 | 36156718 | 3.72 | 0.51 | 0.05 | 1 | 0.98 | 3:36156197-36156846 | 14.95 | 1 |
| LineN | 3 | 73561998 | 73562843 | 2.25 | 0.99 | 0.99 | 1 | 0.99 | 3:73561879-73563101 | 13.11 | 1 |
| LineN | 3 | 83407799 | 83408320 | 1 | 0.35 | 0.69 | 0 | 0 | 3:83407619-83408514 | 9.91 | 1 |
| LineN | 3 | 96961296 | 96961423 | 3.68 | 0.52 | 0.05 | 1 | 1 | 3:96957515-96961612 | 11.58 | 0.98 |
| LineN | 4 | 11124012 | 11124315 | 5.69 | 0.93 | 1 | 1 | 0.84 | 4:11123901-11124467 | 14.86 | 1 |
| LineN | 4 | 26531091 | 26531782 | NA | NA | NA | NA | NA | 4:26530893-26531990 | 14.03 | 1 |
| LineN | 4 | 29234199 | 29234639 | 6.59 | 0.52 | 0.05 | 1 | 1 | 4:29234085-29234741 | 14.2 | 1 |
| LineN | 4 | 30595806 | 30596369 | 6.01 | 1 | 1 | 1 | 1 | 4:30595679-30596557 | 15.54 | 1 |
| LineN | 4 | 40212511 | 40212674 | 5.03 | 0.51 | 0.05 | 1 | 0.98 | 4:40212346-40213006 | 12.52 | 1 |
| LineN | 4 | 86221056 | 86221721 | 3.71 | 0.99 | 0.99 | 1 | 0.98 | 4:86220862-86221807 | 10.39 | 1 |
| LineN | 5 | 2705241 | 2705987 | 3.9 | 0.9 | 0.99 | 1 | 0.77 | 5:2705052-2706284 | 9.45 | 1 |
| LineN | 5 | 31304022 | 31304910 | 1 | 0.17 | 0.34 | 0 | 0 | 5:31303866-31305068 | 10.67 | 1 |
| LineN | 5 | 31898830 | 31899731 | 2.54 | 0.69 | 0.67 | 1 | 0.68 | 5:31898626-31899883 | 11.32 | 1 |
| LineN | 5 | 55530558 | 55530845 | 1.54 | 0.59 | 0.77 | 0 | 0.45 | 5:55530364-55531097 | 10.84 | 0.98 |
| LineN | 6 | 13724976 | 13725309 | 4.33 | 0.97 | 0.95 | 1 | 0.99 | 6:13724843-13726022 | 9.27 | 1 |
| LineN | 6 | 9811403 | 9811669 | 8.2 | 1 | 1 | 1 | 1 | 6:9811191-9816444 | 12.85 | 0.99 |
| LineN | 6 | 9815970 | 9816315 | 6.72 | 0.99 | 0.99 | 1 | 0.99 | 6:9811191-9816444 | 12.85 | 0.99 |
| LineN | 8 | 9208688 | 9209076 | 2.09 | 0.67 | 0.46 | 1 | 0.88 | 8:9208394-9209273 | 6.39 | 0.99 |
| LineN | AADN03016632.1 | 789 | 910 | 3.28 | 0.52 | 0.05 | 1 | 1 | AADN03016632.1:765-923 | 10.76 | 1 |
| LineN | AADN03018735.1 | 511 | 648 | 2.87 | 0.98 | 0.99 | 1 | 0.98 | AADN03018735.1:281-1293 | 11.11 | 1 |
| LineN | Z | 15471053 | 15471352 | 3.42 | 0.97 | 0.93 | 1 | 1 | Z:15470956-15471752 | 7.68 | 0.85 |
| LineN | Z | 30996783 | 30996890 | 3.36 | 0.5 | 0.59 | 1 | 0.33 | Z:30996146-31000811 | 11.07 | 0.96 |
| LineN | Z | 32081368 | 32081622 | 4.83 | 1 | 1 | 1 | 1 | Z:32081154-32086293 | 12.07 | 0.99 |
| LineN | Z | 32085864 | 32086163 | 4.05 | 0.99 | 1 | 1 | 0.98 | Z:32081154-32086293 | 12.07 | 0.99 |
| LineN | Z | 61305057 | 61305850 | 2.03 | 0.78 | 0.79 | 0.76 | 0.77 | Z:61304819-61306021 | 15.32 | 1 |
| LineN | Z | 79945635 | 79946197 | 4 | 1 | 1 | 1 | 1 | Z:79945501-79946432 | 8.8 | 1 |
| LineP | 1 | 101333655 | 101334309 | 10.22 | 1 | 1 | 1 | 1 | 1:101333491-101334404 | 20.13 | 1 |
| LineP | 1 | 113945736 | 113946295 | 5.53 | 0.36 | 0 | 0 | 0.84 | 1:113945460-113946362 | 20.69 | 1 |
| LineP | 1 | 126794696 | 126795444 | 4.54 | 0.97 | 1 | 1 | 0.93 | 1:126794615-126795484 | 18.84 | 1 |
| LineP | 1 | 140710473 | 140710830 | 8.29 | 1 | 1 | 1 | 0.99 | 1:140710345-140715423 | 52.61 | 1 |
| LineP | 1 | 140714972 | 140715271 | 12.93 | 1 | 1 | 1 | 1 | 1:140710345-140715423 | 52.61 | 1 |
| LineP | 1 | 146731989 | 146732681 | 15.94 | 0.99 | 1 | 1 | 0.98 | 1:146731910-146732833 | 22.69 | 1 |
| LineP | 1 | 146761593 | 146762130 | 11.07 | 0.47 | 0.05 | 1 | 0.9 | 1:146761527-146762190 | 18.38 | 1 |
| LineP | 1 | 149162809 | 149163257 | 4.77 | 1 | 1 | 1 | 0.99 | 1:149162760-149166227 | 52.34 | 1 |
| LineP | 1 | 149165214 | 149166098 | 7.77 | 0.8 | 1 | 1 | 0.55 | 1:149162760-149166227 | 52.34 | 1 |
| LineP | 1 | 149935224 | 149935516 | 5.95 | 0.52 | 0.05 | 1 | 1 | 1:149935105-149935762 | 18.59 | 1 |
| LineP | 1 | 157450191 | 157450848 | 15.75 | 0.51 | 0.05 | 1 | 0.98 | 1:157450062-157450954 | 18.51 | 1 |
| LineP | 1 | 180746980 | 180747848 | 6.54 | 0.51 | 0.05 | 1 | 0.99 | 1:180746896-180747970 | 24.55 | 1 |
| LineP | 1 | 182832958 | 182834011 | 9.88 | 0.97 | 0.95 | 1 | 0.98 | 1:182832847-182834082 | 31.06 | 1 |
| LineP | 1 | 184348028 | 184348440 | 1.72 | 0.91 | 1 | 1 | 0.8 | 1:184347893-184348804 | 26.22 | 1 |
| LineP | 1 | 187340725 | 187340849 | 4.02 | 0.49 | 0.05 | 1 | 0.94 | 1:187340668-187342356 | 10.1 | 1 |
| LineP | 1 | 187341996 | 187342293 | 8.32 | 0.51 | 0.05 | 1 | 0.98 | 1:187340668-187342356 | 10.1 | 1 |
| LineP | 1 | 188149293 | 188149457 | 1 | 0.35 | 0.69 | 0 | 0 | 1:188148835-188149736 | 20.45 | 1 |
| LineP | 13 | 10909971 | 10910758 | 7.85 | 0.93 | 0.88 | 1 | 0.98 | 13:10909806-10910958 | 21.65 | 1 |
| LineP | 1 | 32312392 | 32313016 | 9.97 | 0.95 | 1 | 1 | 0.88 | 1:32312218-32313185 | 16.35 | 1 |
| LineP | 13 | 6484801 | 6485484 | 5.45 | 0.97 | 1 | 1 | 0.92 | 13:6484735-6485598 | 20.42 | 1 |
| LineP | 1 | 37362094 | 37362376 | 6.35 | 0.37 | 0 | 0 | 0.85 | 1:37362031-37362653 | 17.68 | 1 |
| LineP | 1 | 41194711 | 41195029 | 2.03 | 0.51 | 0.03 | 1 | 0.99 | 1:41194458-41195110 | 27.11 | 1 |
| LineP | 1 | 41319010 | 41319714 | 10.04 | 0.99 | 0.99 | 1 | 0.98 | 1:41318900-41319777 | 21.46 | 1 |
| LineP | 1 | 43882821 | 43883794 | 4.38 | 0.87 | 0.76 | 1 | 0.98 | 1:43882636-43883815 | 18.6 | 1 |
| LineP | 1 | 52743902 | 52744440 | 8.65 | 0.51 | 0.05 | 1 | 0.98 | 1:52743902-52744500 | 21.15 | 1 |
| LineP | 1 | 63571711 | 63571996 | 3.63 | 0.52 | 0.05 | 1 | 1 | 1:63571454-63572079 | 16.5 | 1 |
| LineP | 1 | 67550515 | 67551024 | 6.12 | 0.99 | 1 | 1 | 0.98 | 1:67550436-67551166 | 31.52 | 1 |
| LineP | 17 | 180728 | 181319 | 13.75 | 0.99 | 0.99 | 1 | 0.99 | 17:180636-181570 | 23.17 | 1 |
| LineP | 1 | 96182489 | 96182609 | 1.67 | 0.42 | 0.03 | 1 | 0.8 | 1:96182372-96182610 | 19.67 | 1 |
| LineP | 20 | 7661961 | 7662702 | 10.83 | 1 | 1 | 1 | 1 | 20:7661897-7662786 | 21 | 1 |
| LineP | 2 | 104297304 | 104297785 | 5.34 | 0.51 | 0.05 | 1 | 0.98 | 2:104297204-104297861 | 14.43 | 1 |
| LineP | 2 | 142114149 | 142114454 | 2.64 | 0.45 | 0 | 0.47 | 0.98 | 2:142113866-142114543 | 28.15 | 1 |
| LineP | 2 | 20971618 | 20972185 | 13.42 | 0.61 | 0.25 | 1 | 0.98 | 2:20971534-20972437 | 20.13 | 1 |
| LineP | 2 | 21240779 | 21241100 | 7.03 | 0.51 | 0.05 | 1 | 0.98 | 2:21240687-21244651 | 30.58 | 1 |
| LineP | 2 | 21244566 | 21244662 | 1.5 | 0.4 | 0.05 | 1 | 0.73 | 2:21240687-21244651 | 30.58 | 1 |
| LineP | 2 | 51801136 | 51801839 | 10.4 | 0.98 | 0.98 | 1 | 0.98 | 2:51801055-51801954 | 23.3 | 1 |
| LineP | 2 | 53111889 | 53112008 | 3.73 | 0.56 | 0.49 | 1 | 0.58 | 2:53111756-53112017 | 27.12 | 1 |
| LineP | 2 | 7818090 | 7818416 | 11.31 | 0.51 | 0.05 | 1 | 0.99 | 2:7818049-7818465 | 21.78 | 1 |
| LineP | 2 | 80333674 | 80334001 | 4.99 | 0.51 | 0.05 | 1 | 0.98 | 2:80333585-80334273 | 14.5 | 1 |
| LineP | 2 | 81965058 | 81965187 | 7.92 | 0.4 | 0 | 0 | 0.93 | 2:81965001-81966072 | 19.9 | 1 |
| LineP | 2 | 82574445 | 82575456 | 9.73 | 0.86 | 0.99 | 1 | 0.69 | 2:82574297-82575518 | 26.69 | 1 |
| LineP | 2 | 92857086 | 92857942 | 14.37 | 0.71 | 0.99 | 1 | 0.35 | 2:92856996-92858219 | 22.16 | 1 |
| LineP | 3 | 19821847 | 19822941 | 6.98 | 0.45 | 0.88 | 0 | 0 | 3:19821802-19823007 | 15.39 | 1 |
| LineP | 3 | 34782979 | 34783326 | 8.12 | 0.51 | 0.05 | 1 | 0.98 | 3:34782953-34783379 | 37.41 | 1 |
| LineP | 3 | 36156261 | 36156786 | 11.56 | 0.51 | 0.05 | 1 | 0.99 | 3:36156197-36156846 | 24.11 | 1 |
| LineP | 3 | 73561966 | 73562992 | 3.4 | 0.91 | 0.83 | 1 | 0.99 | 3:73561879-73563101 | 24.2 | 1 |
| LineP | 3 | 83407680 | 83408450 | 5.65 | 1 | 1 | 1 | 1 | 3:83407619-83408514 | 14.86 | 0.83 |
| LineP | 3 | 83777165 | 83777869 | 3.36 | 0.42 | 0 | 0.76 | 0.87 | 3:83776894-83777945 | 25.33 | 1 |
| LineP | 3 | 86869673 | 86870214 | 6.84 | 0.51 | 0.05 | 1 | 0.98 | 3:86869588-86870258 | 25.07 | 1 |
| LineP | 3 | 96961350 | 96961487 | 1.47 | 0.52 | 0.05 | 1 | 1 | 3:96957515-96961612 | 19.97 | 0.98 |
| LineP | 3 | 99426079 | 99426621 | 18.88 | 0.52 | 0.05 | 1 | 1 | 3:99426024-99426621 | 24.33 | 1 |
| LineP | 4 | 11124035 | 11124408 | 6.6 | 0.99 | 1 | 1 | 0.98 | 4:11123901-11124467 | 31.57 | 1 |
| LineP | 4 | 15864150 | 15864742 | 8.32 | 0.99 | 1 | 1 | 0.98 | 4:15864150-15864742 | 21.06 | 1 |
| LineP | 4 | 27180476 | 27180790 | 2.24 | 0.47 | 0 | 0.82 | 0.98 | 4:27180311-27180810 | 16.64 | 1 |
| LineP | 4 | 29234121 | 29234603 | 11.92 | 0.52 | 0.05 | 1 | 1 | 4:29234085-29234741 | 24.37 | 1 |
| LineP | 4 | 78859483 | 78859550 | 2.69 | 0.31 | 0.05 | 1 | 0.52 | 4:78859384-78859987 | 19.19 | 1 |
| LineP | 4 | 86220927 | 86221678 | 11.13 | 1 | 1 | 1 | 1 | 4:86220862-86221807 | 17.97 | 0.99 |
| LineP | 5 | 2705174 | 2706073 | 42.09 | 1 | 1 | 1 | 0.99 | 5:2705052-2706284 | 25.06 | 1 |
| LineP | 5 | 31304125 | 31304964 | 15.32 | 1 | 1 | 1 | 1 | 5:31303866-31305068 | 19.67 | 1 |
| LineP | 5 | 31898750 | 31899720 | 24.33 | 0.99 | 0.99 | 1 | 0.98 | 5:31898626-31899883 | 21.17 | 1 |
| LineP | 5 | 41299474 | 41300133 | 9.04 | 0.99 | 1 | 1 | 0.98 | 5:41299361-41300140 | 19.89 | 1 |
| LineP | 5 | 55530364 | 55531097 | 17.91 | 1 | 1 | 1 | 1 | 5:55530364-55531097 | 20.42 | 1 |
| LineP | 5 | 6164335 | 6165433 | 42.02 | 0.99 | 1 | 1 | 0.98 | 5:6164269-6165523 | 24.89 | 1 |
| LineP | 6 | 13725087 | 13725838 | 26.8 | 0.98 | 0.96 | 1 | 1 | 6:13724843-13726022 | 25.78 | 1 |
| LineP | 6 | 9811291 | 9811666 | 19.85 | 0.99 | 0.99 | 1 | 1 | 6:9811191-9816444 | 26.42 | 1 |
| LineP | 6 | 9812170 | 9812270 | 2.69 | 0.94 | 0.88 | 1 | 1 | 6:9811191-9816444 | 26.42 | 1 |
| LineP | 6 | 9815943 | 9816327 | 21.85 | 0.99 | 0.99 | 1 | 1 | 6:9811191-9816444 | 26.42 | 1 |
| LineP | 7 | 17708128 | 17708618 | 9.35 | 0.43 | 0 | 0.94 | 0.87 | 7:17708068-17708753 | 23.8 | 1 |
| LineP | 8 | 9208487 | 9208962 | 3.07 | 1 | 1 | 1 | 0.99 | 8:9208394-9209273 | 13.26 | 1 |
| LineP | 9 | 18008690 | 18009179 | 12.52 | 0.51 | 0.05 | 1 | 0.98 | 9:18008576-18009249 | 20.37 | 1 |
| LineP | AADN03024906.1 | 4128 | 4308 | 3.43 | 0.51 | 0.05 | 1 | 0.98 | AADN03024906.1:3470-4351 | 10.21 | 1 |
| LineP | JH375231.1 | 4922 | 5774 | 16.39 | 0.74 | 0.55 | 1 | 0.92 | JH375231.1:4846-8915 | 10.77 | 0.99 |
| LineP | JH376310.1 | 6612 | 7049 | 5.49 | 0.99 | 0.99 | 1 | 0.98 | JH376310.1:6513-7054 | 5.99 | 1 |
| LineP | Z | 15471175 | 15471341 | 1.94 | 0.86 | 0.73 | 1 | 1 | Z:15470956-15471752 | 8.16 | 0.91 |
| LineP | Z | 30996770 | 30996898 | 6.46 | 0.95 | 0.99 | 1 | 0.9 | Z:30996146-31000811 | 23.72 | 0.98 |
| LineP | Z | 32081328 | 32081603 | 7.61 | 1 | 1 | 1 | 1 | Z:32081154-32086293 | 27.27 | 1 |
| LineP | Z | 32085825 | 32086133 | 12.92 | 1 | 1 | 1 | 0.99 | Z:32081154-32086293 | 27.27 | 1 |
| LineP | Z | 61304965 | 61305918 | 20.61 | 0.99 | 1 | 1 | 0.98 | Z:61304819-61306021 | 14.89 | 1 |
| LineP | Z | 79945605 | 79946362 | 12.77 | 1 | 1 | 1 | 1 | Z:79945501-79946432 | 10.81 | 1 |
| LineZero | 1 | 101165329 | 101166103 | 4.51 | 1 | 1 | 1 | 1 | 1:101165329-101166193 | 13.78 | 1 |
| LineZero | 1 | 140710401 | 140710835 | 8.07 | 1 | 1 | 1 | 0.99 | 1:140710345-140715423 | 24.97 | 1 |
| LineZero | 1 | 140714978 | 140715351 | 11 | 1 | 1 | 1 | 1 | 1:140710345-140715423 | 24.97 | 1 |
| LineZero | 1 | 146731925 | 146732543 | 2.15 | 0.98 | 0.97 | 1 | 1 | 1:146731910-146732833 | 17.26 | 1 |
| LineZero | 1 | 146761597 | 146761709 | 1.84 | 0.52 | 0.05 | 1 | 1 | 1:146761527-146762190 | 13.37 | 1 |
| LineZero | 1 | 149162896 | 149163144 | 2.17 | 0.77 | 0.95 | 0 | 0.65 | 1:149162760-149166227 | 19.02 | 1 |
| LineZero | 1 | 149165139 | 149165349 | 2.32 | 0.82 | 0.64 | 1 | 1 | 1:149162760-149166227 | 19.02 | 1 |
| LineZero | 1 | 149165922 | 149166094 | 1.4 | 0.5 | 0.33 | 1 | 0.64 | 1:149162760-149166227 | 19.02 | 1 |
| LineZero | 1 | 157450062 | 157450454 | 9.64 | 0.51 | 0.05 | 1 | 0.98 | 1:157450062-157450954 | 13.85 | 1 |
| LineZero | 1 | 15904038 | 15904160 | 4.63 | 0.51 | 0.04 | 1 | 1 | 1:15904038-15904160 | 20.34 | 1 |
| LineZero | 1 | 163501535 | 163501830 | 3.54 | 0.52 | 0.05 | 1 | 1 | 1:163501535-163501830 | 18.6 | 1 |
| LineZero | 1 | 163906737 | 163907011 | 5.95 | 0.52 | 0.05 | 1 | 1 | 1:163906737-163910850 | 24.78 | 1 |
| LineZero | 1 | 163910477 | 163910850 | 14.78 | 0.51 | 0.05 | 1 | 0.99 | 1:163906737-163910850 | 24.78 | 1 |
| LineZero | 1 | 164414979 | 164415166 | 2.5 | 0.51 | 0.05 | 1 | 0.98 | 1:164414998-164416037 | 17.75 | 1 |
| LineZero | 1 | 164415700 | 164415812 | 3.02 | 0.51 | 0.05 | 1 | 0.98 | 1:164414998-164416037 | 17.75 | 1 |
| LineZero | 1 | 180746931 | 180747255 | 7.28 | 0.51 | 0.05 | 1 | 0.99 | 1:180746896-180747970 | 15.77 | 1 |
| LineZero | 1 | 180747859 | 180747995 | 3.14 | 0.51 | 0.05 | 1 | 0.99 | 1:180746896-180747970 | 15.77 | 1 |
| LineZero | 1 | 182833163 | 182833264 | 1 | 0.57 | 0.68 | 1 | 0.37 | 1:182832847-182834082 | 16.83 | 1 |
| LineZero | 1 | 182833931 | 182834038 | 1.33 | 0.53 | 0.93 | 0.88 | 0 | 1:182832847-182834082 | 16.83 | 1 |
| LineZero | 1 | 184347893 | 184348804 | 14.17 | 0.99 | 1 | 1 | 0.98 | 1:184347893-184348804 | 21.97 | 1 |
| LineZero | 1 | 185856854 | 185857511 | 8.5 | 0.51 | 0.05 | 1 | 0.98 | 1:185856831-185857511 | 15.19 | 1 |
| LineZero | 1 | 187340631 | 187340781 | 2.57 | 0.48 | 0.05 | 1 | 0.92 | 1:187340668-187342356 | 4.27 | 0.4 |
| LineZero | 1 | 187342011 | 187342335 | 8.77 | 0.51 | 0.05 | 1 | 0.98 | 1:187340668-187342356 | 4.27 | 0.4 |
| LineZero | 12 | 3240735 | 3240835 | 3.15 | 0.95 | 1 | 1 | 0.9 | 12:3240697-3240898 | 66.97 | 1 |
| LineZero | 12 | 3277416 | 3278287 | 19.33 | 0.99 | 1 | 1 | 0.98 | 12:3277416-3278316 | 80.22 | 1 |
| LineZero | 1 | 32312224 | 32313185 | 11.39 | 0.99 | 1 | 1 | 0.98 | 1:32312218-32313185 | 12.28 | 1 |
| LineZero | 1 | 32508365 | 32508761 | 7.3 | 0.51 | 0.05 | 1 | 0.99 | 1:32508365-32508761 | 11.48 | 1 |
| LineZero | 13 | 6876434 | 6877334 | 10.59 | 0.99 | 0.99 | 1 | 0.98 | 13:6876434-6877337 | 14.99 | 1 |
| LineZero | 1 | 37362273 | 37362653 | 7.94 | 0.52 | 0.05 | 1 | 1 | 1:37362031-37362653 | 17.88 | 1 |
| LineZero | 1 | 41318987 | 41319736 | 6.59 | 0.95 | 0.92 | 1 | 0.98 | 1:41318900-41319777 | 10.52 | 1 |
| LineZero | 1 | 42541928 | 42542339 | 3.13 | 0.51 | 0.05 | 1 | 0.98 | 1:42541895-42542614 | 19.03 | 1 |
| LineZero | 1 | 63571627 | 63572079 | 10.13 | 0.52 | 0.05 | 1 | 1 | 1:63571454-63572079 | 12.5 | 1 |
| LineZero | 1 | 67550475 | 67551166 | 18.54 | 1 | 1 | 1 | 1 | 1:67550436-67551166 | 30.15 | 1 |
| LineZero | 17 | 180659 | 181527 | 9.22 | 0.91 | 0.83 | 1 | 0.98 | 17:180636-181570 | 19.64 | 1 |
| LineZero | 1 | 87425101 | 87425826 | 8.21 | 0.47 | 0.05 | 1 | 0.9 | 1:87425101-87425826 | 12.43 | 1 |
| LineZero | 1 | 90099919 | 90100600 | 15.97 | 0.51 | 0.05 | 1 | 0.99 | 1:90099919-90100600 | 14.97 | 1 |
| LineZero | 1 | 96182436 | 96182569 | 4.25 | 0.36 | 0 | 0 | 0.84 | 1:96182372-96182610 | 10.19 | 1 |
| LineZero | 1 | 99050555 | 99051202 | 5.02 | 0.52 | 0.05 | 1 | 1 | 1:99050515-99051202 | 12.48 | 1 |
| LineZero | 20 | 7661921 | 7662786 | 9 | 1 | 1 | 1 | 1 | 20:7661897-7662786 | 13.52 | 1 |
| LineZero | 2 | 109248726 | 109249064 | 7.92 | 0.51 | 0.05 | 1 | 0.98 | 2:109248726-109249064 | 14.44 | 1 |
| LineZero | 2 | 131563774 | 131564210 | 5.19 | 0.51 | 0.05 | 1 | 0.98 | 2:131563757-131567794 | 28.31 | 1 |
| LineZero | 2 | 131567661 | 131567783 | 3.27 | 0.52 | 0.05 | 1 | 1 | 2:131563757-131567794 | 28.31 | 1 |
| LineZero | 2 | 20971554 | 20972407 | 12.31 | 0.62 | 0.27 | 1 | 0.99 | 2:20971534-20972437 | 15.36 | 1 |
| LineZero | 2 | 3491394 | 3492195 | 7.25 | 1 | 1 | 1 | 1 | 2:3491394-3492279 | 15.3 | 1 |
| LineZero | 2 | 51801081 | 51801932 | 13.09 | 1 | 1 | 1 | 0.99 | 2:51801055-51801954 | 16.66 | 1 |
| LineZero | 2 | 53111791 | 53112009 | 1.1 | 0.64 | 0.76 | 1 | 0.44 | 2:53111756-53112017 | 14.87 | 1 |
| LineZero | 2 | 73742831 | 73743267 | 8.34 | 0.52 | 0.05 | 1 | 1 | 2:73742831-73743267 | 13.06 | 1 |
| LineZero | 2 | 7818082 | 7818361 | 6.48 | 0.51 | 0.05 | 1 | 0.99 | 2:7818049-7818465 | 14.65 | 1 |
| LineZero | 2 | 80333588 | 80334234 | 7.96 | 0.51 | 0.05 | 1 | 0.98 | 2:80333585-80334273 | 13.44 | 0.99 |
| LineZero | 2 | 81965030 | 81966072 | 5.13 | 0.5 | 0.05 | 1 | 0.97 | 2:81965001-81966072 | 14.12 | 1 |
| LineZero | 2 | 82574297 | 82575518 | 1 | 0.8 | 0.82 | 0.94 | 0.76 | 2:82574297-82575518 | 14.08 | 1 |
| LineZero | 2 | 92856996 | 92858144 | 8.13 | 0.99 | 0.99 | 1 | 0.98 | 2:92856996-92858219 | 13.32 | 1 |
| LineZero | 3 | 19822126 | 19822959 | 7 | 0.61 | 0.86 | 0 | 0.4 | 3:19821802-19823007 | 9.15 | 1 |
| LineZero | 3 | 36156197 | 36156846 | 9.7 | 0.51 | 0.05 | 1 | 0.99 | 3:36156197-36156846 | 20.51 | 1 |
| LineZero | 3 | 73561936 | 73563101 | 2.17 | 0.61 | 0.25 | 1 | 0.98 | 3:73561879-73563101 | 15.15 | 1 |
| LineZero | 3 | 83407641 | 83408514 | 6.19 | 1 | 1 | 1 | 1 | 3:83407619-83408514 | 9.56 | 0.83 |
| LineZero | 3 | 83776865 | 83777270 | 11.24 | 0.47 | 0.05 | 1 | 0.89 | 3:83776894-83777945 | 19.07 | 1 |
| LineZero | 3 | 96957515 | 96957902 | 5.76 | 0.51 | 0.05 | 1 | 0.98 | 3:96957515-96961612 | 21.16 | 1 |
| LineZero | 3 | 96961400 | 96961560 | 5.22 | 0.52 | 0.05 | 1 | 1 | 3:96957515-96961612 | 21.16 | 1 |
| LineZero | 4 | 26530936 | 26531248 | 10.44 | 0.51 | 0.05 | 1 | 0.98 | 4:26530893-26531990 | 21.12 | 1 |
| LineZero | 4 | 26531784 | 26531990 | 7.46 | 0.49 | 0.02 | 1 | 0.98 | 4:26530893-26531990 | 21.12 | 1 |
| LineZero | 4 | 40212346 | 40212723 | 6.77 | 0.51 | 0.05 | 1 | 0.98 | 4:40212346-40213006 | 10.51 | 1 |
| LineZero | 4 | 70463742 | 70464015 | 2.46 | 0.52 | 0.05 | 1 | 1 | 4:70463742-70464439 | 15.11 | 1 |
| LineZero | 4 | 72515153 | 72515431 | 2.73 | 0.51 | 0.05 | 1 | 0.98 | 4:72515182-72515626 | 9.9 | 1 |
| LineZero | 5 | 19302242 | 19303103 | 4.67 | 1 | 1 | 1 | 1 | 5:19302240-19303103 | 13.63 | 1 |
| LineZero | 5 | 2705075 | 2706178 | 12.39 | 1 | 1 | 1 | 1 | 5:2705052-2706284 | 25.18 | 1 |
| LineZero | 5 | 31303958 | 31305006 | 18.59 | 1 | 0.99 | 1 | 1 | 5:31303866-31305068 | 16.13 | 1 |
| LineZero | 5 | 31525567 | 31526167 | 3.71 | 1 | 1 | 1 | 1 | 5:31525368-31526271 | 14.38 | 1 |
| LineZero | 5 | 31898626 | 31899741 | 20.99 | 0.99 | 0.99 | 1 | 0.98 | 5:31898626-31899883 | 13.28 | 1 |
| LineZero | 5 | 40463978 | 40464844 | 10 | 0.99 | 0.99 | 1 | 0.99 | 5:40463978-40464915 | 15.73 | 1 |
| LineZero | 5 | 6164269 | 6165523 | 35.94 | 0.99 | 1 | 1 | 0.98 | 5:6164269-6165523 | 24.07 | 1 |
| LineZero | 6 | 13724912 | 13726003 | 18.23 | 0.98 | 0.96 | 1 | 0.99 | 6:13724843-13726022 | 19.06 | 1 |
| LineZero | 6 | 9811279 | 9811632 | 9.01 | 0.99 | 0.99 | 1 | 1 | 6:9811191-9816444 | 20.61 | 0.99 |
| LineZero | 6 | 9815237 | 9815374 | 4.72 | 0.99 | 0.99 | 1 | 0.98 | 6:9811191-9816444 | 20.61 | 0.99 |
| LineZero | 6 | 9816003 | 9816420 | 9.97 | 0.99 | 0.99 | 1 | 0.98 | 6:9811191-9816444 | 20.61 | 0.99 |
| LineZero | 7 | 17708614 | 17708762 | 5.12 | 0.51 | 0.04 | 1 | 0.98 | 7:17708068-17708753 | 15.93 | 1 |
| LineZero | AADN03018589.1 | 648 | 744 | 2.92 | 0.49 | 0.01 | 1 | 0.98 | AADN03018589.1:484-802 | 10.92 | 1 |
| LineZero | AADN03025776.1 | 820 | 1020 | 7.04 | 0.52 | 0.05 | 1 | 1 | AADN03025776.1:820-1020 | 23.51 | 1 |
| LineZero | JH375212.1 | 37389 | 37728 | 4.68 | 0.51 | 0.05 | 1 | 0.98 | JH375212.1:37241-37702 | 6.9 | 1 |
| LineZero | JH375231.1 | 5451 | 5784 | 5 | 0.87 | 0.85 | 1 | 0.87 | JH375231.1:4846-8915 | 9.6 | 1 |
| LineZero | JH376310.1 | 6513 | 7049 | 14.77 | 0.97 | 0.95 | 1 | 0.99 | JH376310.1:6513-7054 | 9.07 | 1 |
| LineZero | Z | 32081305 | 32081539 | 2.11 | 1 | 1 | 1 | 1 | Z:32081154-32086293 | 18 | 1 |
| LineZero | Z | 32085825 | 32086268 | 12.56 | 0.91 | 0.99 | 0 | 0.94 | Z:32081154-32086293 | 18 | 1 |
| LineZero | Z | 61305161 | 61305772 | 19.52 | 0.98 | 0.98 | 1 | 0.98 | Z:61304819-61306021 | 9.89 | 1 |
| Wellcome | 1 | 113946045 | 113946202 | 6.41 | 0.36 | 0 | 0 | 0.83 | 1:113945460-113946362 | 13.78 | 1 |
| Wellcome | 1 | 140710497 | 140710856 | 8 | 1 | 1 | 1 | 0.99 | 1:140710345-140715423 | 38.06 | 1 |
| Wellcome | 1 | 140714969 | 140715265 | 13.01 | 1 | 1 | 1 | 1 | 1:140710345-140715423 | 38.06 | 1 |
| Wellcome | 1 | 146731976 | 146732735 | 9.38 | 1 | 1 | 1 | 1 | 1:146731910-146732833 | 16.42 | 1 |
| Wellcome | 1 | 146761779 | 146762120 | 4.41 | 0.47 | 0.05 | 1 | 0.9 | 1:146761527-146762190 | 15.77 | 0.98 |
| Wellcome | 1 | 149162814 | 149163230 | 11.06 | 1 | 1 | 1 | 0.99 | 1:149162760-149166227 | 55.77 | 1 |
| Wellcome | 1 | 149165133 | 149166091 | 5.65 | 0.9 | 1 | 1 | 0.77 | 1:149162760-149166227 | 55.77 | 1 |
| Wellcome | 1 | 160455277 | 160455591 | 5.43 | 0.51 | 0.05 | 1 | 0.98 | 1:160455218-160455708 | 8.05 | 1 |
| Wellcome | 1 | 160463921 | 160464074 | 8.57 | 0.52 | 0.05 | 1 | 1 | 1:160463921-160464245 | 14.78 | 1 |
| Wellcome | 1 | 187341926 | 187342280 | 5.57 | 0.51 | 0.05 | 1 | 0.98 | 1:187340668-187342356 | 6.83 | 0.41 |
| Wellcome | 1 | 21322138 | 21322493 | 8.29 | 0.51 | 0.05 | 1 | 0.99 | 1:21322138-21322493 | 14.33 | 1 |
| Wellcome | 12 | 3240739 | 3240840 | 3.42 | 0.51 | 1 | 0 | 0 | 12:3240697-3240898 | 59.98 | 1 |
| Wellcome | 12 | 3277807 | 3278287 | 36.18 | 0.99 | 1 | 1 | 0.98 | 12:3277416-3278316 | 91.06 | 1 |
| Wellcome | 1 | 23890739 | 23891336 | 4.51 | 0.52 | 0.05 | 1 | 1 | 1:23890739-23891336 | 20.8 | 1 |
| Wellcome | 13 | 1868408 | 1868957 | 7.13 | 0.51 | 0.05 | 1 | 0.98 | 13:1868296-1868957 | 19.94 | 1 |
| Wellcome | 13 | 6876470 | 6877320 | 7.87 | 0.98 | 0.99 | 1 | 0.98 | 13:6876434-6877337 | 13.28 | 1 |
| Wellcome | 1 | 41319115 | 41319593 | 6.39 | 0.89 | 0.99 | 1 | 0.76 | 1:41318900-41319777 | 16.5 | 1 |
| Wellcome | 14 | 236512 | 236813 | 4.61 | 0.51 | 0.04 | 1 | 0.99 | 14:236512-236813 | 22.62 | 1 |
| Wellcome | 1 | 42542203 | 42542469 | 5.84 | 0.51 | 0.05 | 1 | 0.98 | 1:42541895-42542614 | 15.53 | 1 |
| Wellcome | 1 | 63571574 | 63572021 | 7.42 | 0.52 | 0.05 | 1 | 1 | 1:63571454-63572079 | 12.64 | 1 |
| Wellcome | 1 | 67550514 | 67551050 | 13.37 | 1 | 1 | 1 | 1 | 1:67550436-67551166 | 25.99 | 1 |
| Wellcome | 17 | 180713 | 181436 | 9.43 | 0.99 | 0.99 | 1 | 0.98 | 17:180636-181570 | 20.27 | 1 |
| Wellcome | 1 | 99050540 | 99051019 | 8.66 | 0.51 | 0.05 | 1 | 0.98 | 1:99050515-99051202 | 16.52 | 1 |
| Wellcome | 1 | 99136741 | 99136842 | 2.12 | 0.39 | 0 | 0.24 | 0.87 | 1:99136310-99136953 | 17.2 | 1 |
| Wellcome | 20 | 7661993 | 7662714 | 10.24 | 1 | 1 | 1 | 1 | 20:7661897-7662786 | 15.4 | 1 |
| Wellcome | 2 | 104297315 | 104297578 | 4.4 | 0.51 | 0.05 | 1 | 0.98 | 2:104297204-104297861 | 15.95 | 1 |
| Wellcome | 2 | 124765112 | 124765479 | 2.92 | 0.47 | 0 | 0.82 | 0.98 | 2:124765081-124765718 | 12.7 | 1 |
| Wellcome | 2 | 131567524 | 131567648 | 4.51 | 0.52 | 0.05 | 1 | 1 | 2:131563757-131567794 | 26.76 | 1 |
| Wellcome | 2 | 20971567 | 20972368 | 11.42 | 0.61 | 0.25 | 1 | 0.98 | 2:20971534-20972437 | 12.88 | 1 |
| Wellcome | 2 | 21240963 | 21241100 | 4.09 | 0.51 | 0.05 | 1 | 0.98 | 2:21240687-21244651 | 27.81 | 1 |
| Wellcome | 2 | 21244451 | 21244564 | 4.53 | 0.51 | 0.05 | 1 | 0.99 | 2:21240687-21244651 | 27.81 | 1 |
| Wellcome | 2 | 3491491 | 3492132 | 5.26 | 0.99 | 1 | 1 | 0.98 | 2:3491394-3492279 | 15.37 | 1 |
| Wellcome | 2 | 51801351 | 51801685 | 4.5 | 0.83 | 0.68 | 1 | 0.99 | 2:51801055-51801954 | 18.18 | 1 |
| Wellcome | 2 | 7818255 | 7818465 | 7 | 0.51 | 0.05 | 1 | 0.99 | 2:7818049-7818465 | 16.51 | 1 |
| Wellcome | 2 | 80333690 | 80334022 | 2.55 | 0.4 | 0 | 0 | 0.92 | 2:80333585-80334273 | 10.13 | 0.99 |
| Wellcome | 2 | 81965109 | 81965998 | 4.01 | 0.5 | 0.05 | 1 | 0.96 | 2:81965001-81966072 | 14.47 | 1 |
| Wellcome | 2 | 82574318 | 82575474 | 10.14 | 0.99 | 1 | 1 | 0.98 | 2:82574297-82575518 | 20.68 | 1 |
| Wellcome | 2 | 92857158 | 92858129 | 3.83 | 0.83 | 0.83 | 1 | 0.81 | 2:92856996-92858219 | 14.1 | 1 |
| Wellcome | 2 | 95481020 | 95481760 | 7.44 | 0.99 | 1 | 1 | 0.98 | 2:95480957-95481836 | 11.31 | 1 |
| Wellcome | 3 | 19822017 | 19822864 | 4.21 | 0.64 | 0.92 | 0 | 0.4 | 3:19821802-19823007 | 11.46 | 1 |
| Wellcome | 3 | 34783007 | 34783255 | 2.88 | 0.51 | 0.05 | 1 | 0.98 | 3:34782953-34783379 | 28.82 | 1 |
| Wellcome | 3 | 46003530 | 46004338 | 11.18 | 1 | 1 | 1 | 1 | 3:46003530-46004338 | 19.18 | 1 |
| Wellcome | 3 | 49572091 | 49572843 | 5.61 | 1 | 1 | 1 | 1 | 3:49572068-49572931 | 20.78 | 1 |
| Wellcome | 3 | 59937604 | 59938107 | 3.28 | 0.5 | 0.05 | 1 | 0.96 | 3:59937540-59938220 | 14.11 | 1 |
| Wellcome | 3 | 70604657 | 70605239 | 1.95 | 0.53 | 1 | 0.35 | 0 | 3:70604555-70605387 | 14.09 | 0.95 |
| Wellcome | 3 | 73562100 | 73563060 | 3.32 | 0.33 | 0 | 0 | 0.77 | 3:73561879-73563101 | 14.8 | 1 |
| Wellcome | 3 | 83408055 | 83408421 | 5.2 | 1 | 1 | 1 | 1 | 3:83407619-83408514 | 12.12 | 0.99 |
| Wellcome | 3 | 83777189 | 83777880 | 1.45 | 0.46 | 0.05 | 1 | 0.86 | 3:83776894-83777945 | 19.6 | 1 |
| Wellcome | 3 | 93697680 | 93698465 | 8.48 | 0.91 | 0.84 | 1 | 0.98 | 3:93697657-93698572 | 10.71 | 1 |
| Wellcome | 3 | 96957563 | 96957913 | 6.09 | 0.51 | 0.05 | 1 | 0.98 | 3:96957515-96961612 | 26.15 | 1 |
| Wellcome | 3 | 96961383 | 96961498 | 2.86 | 0.52 | 0.05 | 1 | 1 | 3:96957515-96961612 | 26.15 | 1 |
| Wellcome | 3 | 99426305 | 99426586 | 5.03 | 0.52 | 0.05 | 1 | 1 | 3:99426024-99426621 | 15.6 | 1 |
| Wellcome | 4 | 11124003 | 11124392 | 7.28 | 0.99 | 1 | 1 | 0.98 | 4:11123901-11124467 | 22.21 | 1 |
| Wellcome | 4 | 26530976 | 26531845 | 3.13 | 0.45 | 0 | 0.24 | 1 | 4:26530893-26531990 | 19.86 | 1 |
| Wellcome | 4 | 27180311 | 27180810 | 9.05 | 0.51 | 0.05 | 1 | 0.99 | 4:27180311-27180810 | 18.21 | 1 |
| Wellcome | 4 | 29234170 | 29234620 | 7.28 | 0.52 | 0.05 | 1 | 1 | 4:29234085-29234741 | 15.65 | 1 |
| Wellcome | 4 | 30595750 | 30596512 | 11.64 | 1 | 1 | 1 | 1 | 4:30595679-30596557 | 16.28 | 1 |
| Wellcome | 4 | 40212469 | 40212904 | 6.5 | 0.45 | 0.05 | 1 | 0.85 | 4:40212346-40213006 | 12.23 | 1 |
| Wellcome | 4 | 58818773 | 58819243 | 1.62 | 0.65 | 0.34 | 1 | 0.97 | 4:58818773-58819243 | 19.53 | 1 |
| Wellcome | 5 | 2705170 | 2706132 | 9.06 | 0.7 | 0.51 | 1 | 0.88 | 5:2705052-2706284 | 26.42 | 1 |
| Wellcome | 5 | 31303866 | 31304972 | NA | NA | NA | NA | NA | 5:31303866-31305068 | 15.5 | 1 |
| Wellcome | 5 | 31898829 | 31899676 | 9.27 | 0.91 | 0.97 | 1 | 0.82 | 5:31898626-31899883 | 16.69 | 1 |
| Wellcome | 5 | 41299447 | 41300124 | 9.9 | 1 | 1 | 1 | 1 | 5:41299361-41300140 | 13.28 | 1 |
| Wellcome | 6 | 13724918 | 13725742 | 1.44 | 0.49 | 0.84 | 1 | 0.01 | 6:13724843-13726022 | 17.99 | 1 |
| Wellcome | 6 | 9811255 | 9812236 | 3.48 | 1 | 0.99 | 1 | 1 | 6:9811191-9816444 | 23.61 | 0.99 |
| Wellcome | 6 | 9815951 | 9816333 | 9.31 | 0.99 | 0.99 | 1 | 0.98 | 6:9811191-9816444 | 23.61 | 0.99 |
| Wellcome | 7 | 29830142 | 29830590 | 10.55 | 0.51 | 0.05 | 1 | 0.98 | 7:29830070-29830722 | 15.58 | 1 |
| Wellcome | AADN03016632.1 | 783 | 898 | 4.63 | 0.52 | 0.05 | 1 | 1 | AADN03016632.1:765-923 | 17.35 | 1 |
| Wellcome | AADN03024906.1 | 4003 | 4210 | 4.26 | 0.5 | 0.03 | 1 | 0.98 | AADN03024906.1:3470-4351 | 11.21 | 1 |
| Wellcome | JH375231.1 | 4846 | 5617 | 9.36 | 0.76 | 0.55 | 1 | 0.98 | JH375231.1:4846-8915 | 7.27 | 0.99 |
| Wellcome | JH376310.1 | 6659 | 7038 | 4.25 | 0.85 | 0.74 | 0.94 | 0.96 | JH376310.1:6513-7054 | 5.99 | 1 |
| Wellcome | Z | 32081228 | 32081541 | 3.86 | 0.79 | 0.96 | 1 | 0.57 | Z:32081154-32086293 | 26.27 | 1 |
| Wellcome | Z | 32085867 | 32086081 | 7.28 | 0.99 | 1 | 1 | 0.98 | Z:32081154-32086293 | 26.27 | 1 |
| Wellcome | Z | 50872968 | 50873178 | 3.42 | 0.51 | 0.04 | 1 | 0.98 | Z:50872653-50873337 | 5.33 | 0.99 |
| Wellcome | Z | 61304956 | 61305708 | 21.89 | 0.91 | 0.83 | 1 | 0.99 | Z:61304819-61306021 | 11.28 | 1 |
| RJF | 1 | 140710511 | 140710872 | 21.48 | 1 | 1 | 1 | 0.99 | 1:140710345-140715423 | 59.83 | 1 |
| RJF | 1 | 140714361 | 140715230 | 2.03 | 0.4 | 0.79 | 0 | 0 | 1:140710345-140715423 | 59.83 | 1 |
| RJF | 1 | 148568134 | 148568461 | 21.74 | 0.51 | 0.05 | 1 | 0.98 | 1:148564562-148568615 | 86.26 | 1 |
| RJF | 1 | 149162974 | 149163255 | 12.06 | 1 | 1 | 1 | 0.99 | 1:149162760-149166227 | 60.07 | 1 |
| RJF | 1 | 149165178 | 149166025 | 1.25 | 0.44 | 0.41 | 1 | 0.4 | 1:149162760-149166227 | 60.07 | 1 |
| RJF | 1 | 151410012 | 151410307 | 45.91 | 0.52 | 0.05 | 1 | 0.99 | 1:151407118-151410307 | 61.5 | 0.99 |
| RJF | 1 | 159294092 | 159294382 | 30.4 | 0.51 | 0.05 | 1 | 0.99 | 1:159294092-159294382 | 64.12 | 1 |
| RJF | 1 | 160455392 | 160455708 | 25.07 | 0.51 | 0.05 | 1 | 0.99 | 1:160455218-160455708 | 51.59 | 1 |
| RJF | 1 | 163910330 | 163910681 | 34.34 | 0.51 | 0.05 | 1 | 0.98 | 1:163906737-163910850 | 64.69 | 1 |
| RJF | 1 | 164415166 | 164415884 | 1.5 | 0.39 | 0.05 | 1 | 0.72 | 1:164414998-164416037 | 61.04 | 1 |
| RJF | 1 | 180747124 | 180747762 | NA | NA | NA | NA | NA | 1:180746896-180747970 | 55.44 | 1 |
| RJF | 1 | 182832996 | 182833874 | 2.01 | 0.89 | 0.82 | 1 | 0.94 | 1:182832847-182834082 | 57.21 | 1 |
| RJF | 12 | 15106212 | 15106979 | 2.49 | 0.83 | 0.92 | 0.41 | 0.78 | 12:15106212-15106979 | 28.35 | 1 |
| RJF | 12 | 3277795 | 3278292 | 11.08 | 1 | 1 | 1 | 0.99 | 12:3277416-3278316 | 157.41 | 1 |
| RJF | 13 | 10909930 | 10910774 | 1.96 | 0.47 | 0.5 | 0 | 0.49 | 13:10909806-10910958 | 61.19 | 1 |
| RJF | 1 | 43882858 | 43883632 | 2.83 | 0.45 | 0.84 | 0.41 | 0 | 1:43882636-43883815 | 65.85 | 1 |
| RJF | 1 | 96156897 | 96157152 | 22.27 | 0.99 | 1 | 1 | 0.98 | 1:96156897-96157369 | 67.81 | 1 |
| RJF | 2 | 115446270 | 115446856 | 1.99 | 0.28 | 0.05 | 1 | 0.45 | 2:115446270-115446856 | 34.26 | 1 |
| RJF | 2 | 131563946 | 131564242 | 29.31 | 0.51 | 0.05 | 1 | 0.99 | 2:131563757-131567794 | 72.62 | 1 |
| RJF | 2 | 142513037 | 142513743 | 4 | 0.44 | 0 | 0.18 | 0.98 | 2:142512897-142513907 | 60.85 | 1 |
| RJF | 2 | 143463458 | 143463723 | 21.25 | 0.51 | 0.05 | 1 | 0.98 | 2:143463257-143468861 | 54.93 | 0.98 |
| RJF | 2 | 20044191 | 20044405 | 24.35 | 0.51 | 0.05 | 1 | 0.98 | 2:20044191-20044405 | 35.05 | 1 |
| RJF | 2 | 21240875 | 21241179 | 24.18 | 0.51 | 0.05 | 1 | 0.98 | 2:21240687-21244651 | 75.06 | 1 |
| RJF | 2 | 58047649 | 58047977 | 35.25 | 0.52 | 0.05 | 1 | 0.99 | 2:58047649-58047977 | 67.69 | 1 |
| RJF | 2 | 81458689 | 81458997 | 15.97 | 1 | 1 | 1 | 1 | 2:81458689-81462600 | 47.8 | 1 |
| RJF | 2 | 81462137 | 81462600 | 2 | 0.18 | 0 | 0 | 0.41 | 2:81458689-81462600 | 47.8 | 1 |
| RJF | 2 | 81965218 | 81965871 | 5.03 | 0.25 | 0 | 0 | 0.58 | 2:81965001-81966072 | 66.6 | 1 |
| RJF | 2 | 82574530 | 82575341 | 1.79 | 0.51 | 0.68 | 1 | 0.24 | 2:82574297-82575518 | 66.11 | 1 |
| RJF | 2 | 92857191 | 92857951 | 3.53 | 0.64 | 0.99 | 1 | 0.19 | 2:92856996-92858219 | 51.32 | 1 |
| RJF | 3 | 19822051 | 19822794 | 2.3 | 0.51 | 0.77 | 0 | 0.27 | 3:19821802-19823007 | 54.46 | 1 |
| RJF | 3 | 54250303 | 54250586 | 34.92 | 0.51 | 0.05 | 1 | 0.99 | 3:54250109-54254158 | 73.62 | 1 |
| RJF | 3 | 55831442 | 55831755 | 17.93 | 1 | 1 | 1 | 0.99 | 3:55831442-55836192 | 45.79 | 1 |
| RJF | 3 | 55835868 | 55836192 | 16.13 | 1 | 1 | 1 | 1 | 3:55831442-55836192 | 45.79 | 1 |
| RJF | 3 | 73562061 | 73562884 | 1.42 | 0.37 | 0.53 | 1 | 0.08 | 3:73561879-73563101 | 58.13 | 1 |
| RJF | 3 | 83777060 | 83777752 | 1.66 | 0.37 | 0 | 0 | 0.86 | 3:83776894-83777945 | 78.76 | 1 |
| RJF | 3 | 84892673 | 84892945 | 21.77 | 0.51 | 0.05 | 1 | 0.98 | 3:84889113-84893120 | 68.57 | 1 |
| RJF | 3 | 96957718 | 96958022 | 24.68 | 0.51 | 0.05 | 1 | 0.98 | 3:96957515-96961612 | 68.71 | 1 |
| RJF | 4 | 11123987 | 11124226 | 14.33 | 0.99 | 1 | 1 | 0.98 | 4:11123901-11124467 | 69.48 | 1 |
| RJF | 4 | 26531126 | 26531394 | 25.77 | 0.51 | 0.05 | 1 | 0.98 | 4:26530893-26531990 | 68.39 | 1 |
| RJF | 4 | 55419659 | 55420314 | NA | NA | NA | NA | NA | 4:55419659-55420314 | 56.52 | 1 |
| RJF | 5 | 19126313 | 19126773 | 17.86 | 0.22 | 0 | 0 | 0.51 | 5:19126313-19126773 | 54.89 | 1 |
| RJF | 5 | 2705267 | 2706061 | 3.49 | 0.23 | 0 | 0.76 | 0.42 | 5:2705052-2706284 | 53 | 1 |
| RJF | 5 | 31304065 | 31304847 | 1 | 0.35 | 0 | 0.06 | 0.8 | 5:31303866-31305068 | 58.28 | 1 |
| RJF | 5 | 31898868 | 31899688 | 1.8 | 0.65 | 0.55 | 0 | 0.85 | 5:31898626-31899883 | 58.13 | 1 |
| RJF | 5 | 39163672 | 39164482 | 2.13 | 0.55 | 0.4 | 0 | 0.8 | 5:39163484-39164645 | 58.13 | 1 |
| RJF | 5 | 4737109 | 4737429 | 26.68 | 0.51 | 0.05 | 1 | 0.98 | 5:4737109-4737429 | 55.73 | 1 |
| RJF | 5 | 6164414 | 6165265 | 1.62 | 0.86 | 0.95 | 1 | 0.73 | 5:6164269-6165523 | 55.93 | 1 |
| RJF | 6 | 13725047 | 13725814 | 1.87 | 0.63 | 0.86 | 0.41 | 0.38 | 6:13724843-13726022 | 58.58 | 1 |
| RJF | 6 | 9811364 | 9812260 | 1 | 0.27 | 0.11 | 1 | 0.35 | 6:9811191-9816444 | 53.9 | 1 |
| RJF | 6 | 9815557 | 9816246 | 1.57 | 0.82 | 0.73 | 1 | 0.9 | 6:9811191-9816444 | 53.9 | 1 |
| RJF | 8 | 8899619 | 8900337 | 3.35 | 0.42 | 0 | 0 | 0.98 | 8:8899619-8900337 | 56.2 | 1 |
| RJF | AADN03018735.1 | 321 | 1166 | 3.64 | 0.41 | 0.75 | 0.41 | 0 | AADN03018735.1:281-1293 | 32.62 | 1 |
| RJF | JH375212.1 | 37241 | 37575 | 7.57 | 0.51 | 0.05 | 1 | 0.98 | JH375212.1:37241-37702 | 29.23 | 1 |
| RJF | JH375231.1 | 4893 | 5626 | 1 | 0.62 | 0.68 | 0 | 0.63 | JH375231.1:4846-8915 | 25 | 1 |
| RJF | JH375231.1 | 8174 | 8709 | 1.42 | 0.43 | 0 | 0 | 0.99 | JH375231.1:4846-8915 | 25 | 1 |
| RJF | JH375237.1 | 49867 | 50521 | 1 | 0.19 | 0 | 0 | 0.44 | JH375237.1:49865-50701 | 26.06 | 1 |
| RJF | JH376310.1 | 6805 | 7054 | 9.42 | 0.99 | 0.99 | 1 | 0.98 | JH376310.1:6513-7054 | 30.63 | 1 |
| RJF | Z | 18808285 | 18808949 | 2.5 | 0.22 | 0.21 | 1 | 0.14 | Z:18807991-18809115 | 28.92 | 1 |
| RJF | Z | 30996146 | 30996935 | 2.13 | 0.31 | 0.25 | 0 | 0.44 | Z:30996146-31000811 | 30.45 | 1 |
| RJF | Z | 31000091 | 31000811 | 2.65 | 0.85 | 0.75 | 1 | 0.94 | Z:30996146-31000811 | 30.45 | 1 |
| RJF | Z | 32085773 | 32086047 | 12.21 | 0.99 | 1 | 1 | 0.98 | Z:32081154-32086293 | 35.83 | 1 |
| RJF | Z | 61305043 | 61305820 | 1.96 | 0.2 | 0 | 0.24 | 0.42 | Z:61304819-61306021 | 30.4 | 1 |
| RJF | Z | 64336432 | 64337248 | 1.38 | 0.76 | 0.82 | 1 | 0.65 | Z:64336432-64337248 | 34.21 | 1 |
| RJF | Z | 78845015 | 78845880 | 1.98 | 0.62 | 0.75 | 1 | 0.42 | Z:78844863-78846057 | 27.06 | 1 |
| Silkie | 10 | 9703254 | 9703841 | 7.38 | 0.51 | 0.05 | 1 | 0.99 | 10:9703183-9703845 | 19.35 | 1 |
| Silkie | 1 | 101333491 | 101334404 | 21.36 | 1 | 1 | 1 | 1 | 1:101333491-101334404 | 27.12 | 1 |
| Silkie | 11 | 11174286 | 11174756 | 7.38 | 0.99 | 1 | 1 | 0.98 | 11:11174286-11174756 | 20.3 | 1 |
| Silkie | 1 | 114080840 | 114081279 | 18.52 | 1 | 1 | 1 | 1 | 1:114080840-114081279 | 26.9 | 1 |
| Silkie | 1 | 140710388 | 140710862 | 27.1 | 1 | 1 | 1 | 0.99 | 1:140710345-140715423 | 65.17 | 1 |
| Silkie | 1 | 140714912 | 140715382 | 53.1 | 1 | 1 | 1 | 1 | 1:140710345-140715423 | 65.17 | 1 |
| Silkie | 1 | 141097673 | 141098554 | 37.6 | 0.51 | 0.05 | 1 | 0.99 | 1:141097673-141098554 | 23.09 | 1 |
| Silkie | 1 | 149162760 | 149163252 | 14.39 | 1 | 1 | 1 | 1 | 1:149162760-149166227 | 62.19 | 1 |
| Silkie | 1 | 149165096 | 149166155 | 26.7 | 1 | 1 | 1 | 1 | 1:149162760-149166227 | 62.19 | 1 |
| Silkie | 1 | 153879253 | 153880132 | 22.09 | 1 | 1 | 1 | 1 | 1:153879253-153880132 | 31.2 | 1 |
| Silkie | 1 | 180746953 | 180747948 | 6.36 | 0.52 | 0.05 | 1 | 1 | 1:180746896-180747970 | 16.54 | 1 |
| Silkie | 1 | 182832905 | 182834082 | 17.39 | 0.99 | 1 | 1 | 0.98 | 1:182832847-182834082 | 51.45 | 1 |
| Silkie | 12 | 3240697 | 3240890 | 7.95 | 0.83 | 1 | 1 | 0.61 | 12:3240697-3240898 | 182.78 | 1 |
| Silkie | 12 | 3277778 | 3278313 | 55.37 | 1 | 1 | 1 | 1 | 12:3277416-3278316 | 67.2 | 1 |
| Silkie | 12 | 335283 | 335965 | 42.2 | 0.52 | 0.05 | 1 | 1 | 12:335263-335965 | 33.59 | 1 |
| Silkie | 12 | 6823591 | 6824459 | 20 | 1 | 1 | 1 | 1 | 12:6823591-6824459 | 33.33 | 1 |
| Silkie | 1 | 32130327 | 32131074 | 9.99 | 0.99 | 1 | 1 | 0.98 | 1:32130327-32131074 | 11.9 | 1 |
| Silkie | 1 | 32312372 | 32313048 | 6.09 | 0.99 | 1 | 1 | 0.98 | 1:32312218-32313185 | 10.72 | 1 |
| Silkie | 1 | 42541895 | 42542614 | 9.81 | 0.51 | 0.05 | 1 | 0.99 | 1:42541895-42542614 | 22.82 | 1 |
| Silkie | 14 | 7965945 | 7966669 | 15.53 | 0.99 | 1 | 1 | 0.98 | 14:7965871-7966753 | 26.09 | 1 |
| Silkie | 1 | 52743958 | 52744500 | 10.44 | 0.52 | 0.05 | 1 | 1 | 1:52743902-52744500 | 16.83 | 1 |
| Silkie | 17 | 180636 | 181570 | 36.38 | 0.99 | 0.99 | 1 | 0.99 | 17:180636-181570 | 39.49 | 1 |
| Silkie | 1 | 77444595 | 77445260 | 19.63 | 0.52 | 0.05 | 1 | 1 | 1:77444595-77445260 | 31.23 | 1 |
| Silkie | 1 | 84555463 | 84555939 | 10.94 | 0.51 | 0.05 | 1 | 0.98 | 1:84555430-84556039 | 14.65 | 1 |
| Silkie | 1 | 89899733 | 89900529 | 18.73 | 1 | 1 | 1 | 0.99 | 1:89899733-89900560 | 18.45 | 1 |
| Silkie | 1 | 93473773 | 93474297 | 6.18 | 0.52 | 0.05 | 1 | 1 | 1:93473773-93474297 | 15.4 | 1 |
| Silkie | 2 | 142113866 | 142114543 | 25.54 | 0.51 | 0.05 | 1 | 0.98 | 2:142113866-142114543 | 32.73 | 1 |
| Silkie | 2 | 21240687 | 21241169 | 33.18 | 0.51 | 0.05 | 1 | 0.99 | 2:21240687-21244651 | 64.93 | 1 |
| Silkie | 2 | 21244402 | 21244651 | 17.16 | 0.52 | 0.05 | 1 | 1 | 2:21240687-21244651 | 64.93 | 1 |
| Silkie | 2 | 53111797 | 53112017 | 5.71 | 0.64 | 0.32 | 1 | 0.98 | 2:53111756-53112017 | 31.76 | 1 |
| Silkie | 2 | 65341352 | 65341989 | 5.36 | 0.51 | 0.05 | 1 | 0.99 | 2:65341352-65341989 | 15.37 | 1 |
| Silkie | 2 | 92857046 | 92857955 | 11.86 | 0.99 | 0.99 | 1 | 0.98 | 2:92856996-92858219 | 20.05 | 1 |
| Silkie | 3 | 12508635 | 12509292 | 19.51 | 0.51 | 0.05 | 1 | 0.99 | 3:12508635-12509292 | 29.01 | 1 |
| Silkie | 3 | 19822179 | 19822862 | 7.37 | 0.89 | 0.99 | 0.71 | 0.79 | 3:19821802-19823007 | 9.1 | 0.97 |
| Silkie | 3 | 54250240 | 54250584 | 13.34 | 0.91 | 0.82 | 1 | 1 | 3:54250109-54254158 | 46.89 | 1 |
| Silkie | 3 | 54253844 | 54254157 | 14.16 | 0.52 | 0.05 | 1 | 1 | 3:54250109-54254158 | 46.89 | 1 |
| Silkie | 3 | 63745337 | 63745917 | 24.08 | 0.52 | 0.05 | 1 | 1 | 3:63745322-63745967 | 32.04 | 1 |
| Silkie | 3 | 68891684 | 68892157 | 8.95 | 0.52 | 0.05 | 1 | 1 | 3:68891616-68892157 | 17.89 | 1 |
| Silkie | 3 | 77502831 | 77503430 | 9.12 | 0.51 | 0.05 | 1 | 0.98 | 3:77502831-77503430 | 26.5 | 1 |
| Silkie | 3 | 83407649 | 83408428 | 9.88 | 1 | 1 | 1 | 1 | 3:83407619-83408514 | 16.52 | 1 |
| Silkie | 3 | 83776924 | 83777945 | 20.08 | 0.52 | 0.05 | 1 | 1 | 3:83776894-83777945 | 28.72 | 1 |
| Silkie | 3 | 93697760 | 93698240 | 10.12 | 1 | 1 | 1 | 1 | 3:93697657-93698572 | 14.77 | 0.98 |
| Silkie | 3 | 96957532 | 96958002 | 16.07 | 1 | 1 | 1 | 0.99 | 3:96957515-96961612 | 39.7 | 1 |
| Silkie | 3 | 96961326 | 96961596 | 15.24 | 0.52 | 0.05 | 1 | 1 | 3:96957515-96961612 | 39.7 | 1 |
| Silkie | 4 | 11123901 | 11124448 | 35.15 | 0.99 | 1 | 1 | 0.98 | 4:11123901-11124467 | 54.12 | 1 |
| Silkie | 4 | 19211542 | 19212344 | 24.04 | 1 | 1 | 1 | 1 | 4:19211542-19212344 | 42.18 | 1 |
| Silkie | 4 | 22222499 | 22223210 | 33.7 | 0.52 | 0.06 | 1 | 1 | 4:22222499-22223210 | 40.85 | 1 |
| Silkie | 4 | 26531010 | 26531944 | 6.27 | 0.47 | 0 | 0.76 | 0.99 | 4:26530893-26531990 | 22.54 | 1 |
| Silkie | 4 | 70552107 | 70552669 | 20.07 | 0.99 | 1 | 1 | 0.98 | 4:70551829-70552669 | 17.86 | 0.95 |
| Silkie | 4 | 71535841 | 71536331 | 11.85 | 0.99 | 1 | 1 | 0.98 | 4:71535538-71536391 | 9.17 | 0.88 |
| Silkie | 5 | 10249946 | 10250591 | 30.26 | 0.52 | 0.05 | 1 | 1 | 5:10249946-10250591 | 21.54 | 1 |
| Silkie | 5 | 2705052 | 2706284 | 10.83 | 0.93 | 0.93 | 1 | 0.93 | 5:2705052-2706284 | 43.32 | 1 |
| Silkie | 5 | 31303892 | 31304909 | 1.89 | 0.66 | 0.69 | 0 | 0.7 | 5:31303866-31305068 | 19.72 | 1 |
| Silkie | 5 | 31898753 | 31899749 | 11.92 | 0.99 | 1 | 1 | 0.98 | 5:31898626-31899883 | 21.63 | 1 |
| Silkie | 5 | 39163504 | 39164623 | 14.24 | 0.99 | 0.99 | 1 | 1 | 5:39163484-39164645 | 23.45 | 1 |
| Silkie | 5 | 40464077 | 40464755 | 11.39 | 0.99 | 0.99 | 1 | 1 | 5:40463978-40464915 | 22.51 | 1 |
| Silkie | 5 | 6164279 | 6165519 | 18.26 | 1 | 1 | 1 | 0.99 | 5:6164269-6165523 | 50.07 | 1 |
| Silkie | 6 | 13724938 | 13725997 | 6.38 | 0.99 | 0.99 | 1 | 1 | 6:13724843-13726022 | 29.32 | 1 |
| Silkie | 6 | 9811218 | 9812261 | 9.18 | 0.99 | 0.99 | 1 | 1 | 6:9811191-9816444 | 38.6 | 0.97 |
| Silkie | 6 | 9815948 | 9816436 | 23.48 | 0.99 | 0.99 | 1 | 0.98 | 6:9811191-9816444 | 38.6 | 0.97 |
| Silkie | 7 | 17708068 | 17708753 | 26.75 | 0.51 | 0.04 | 1 | 1 | 7:17708068-17708753 | 35.37 | 1 |
| Silkie | 7 | 9022731 | 9023457 | 18.99 | 0.52 | 0.05 | 1 | 1 | 7:9022731-9023457 | 29.59 | 1 |
| Silkie | 8 | 17696823 | 17697651 | 12.97 | 0.99 | 1 | 1 | 0.98 | 8:17696796-17697651 | 18.83 | 1 |
| Silkie | AADN03018589.1 | 484 | 802 | 14.72 | 0.98 | 0.97 | 1 | 1 | AADN03018589.1:484-802 | 26.92 | 1 |
| Silkie | AADN03018735.1 | 337 | 1293 | 21.31 | 1 | 0.99 | 1 | 1 | AADN03018735.1:281-1293 | 35.34 | 1 |
| Silkie | JH375157.1 | 356 | 536 | 18.74 | 0.52 | 0.05 | 1 | 1 | JH375157.1:356-536 | 26.24 | 1 |
| Silkie | Z | 18807991 | 18809115 | 2.99 | 0.28 | 0.49 | 0.41 | 0 | Z:18807991-18809115 | 35.74 | 1 |
| Silkie | Z | 30996700 | 30996963 | 16.11 | 1 | 1 | 1 | 1 | Z:30996146-31000811 | 22.92 | 0.97 |
| Silkie | Z | 32081161 | 32081653 | 31.82 | 1 | 1 | 1 | 1 | Z:32081154-32086293 | 59.5 | 1 |
| Silkie | Z | 32085814 | 32086227 | 37.52 | 1 | 1 | 1 | 0.99 | Z:32081154-32086293 | 59.5 | 1 |
| Silkie | Z | 3540391 | 3541231 | 10.11 | 0.25 | 0 | 0 | 0.59 | Z:3540391-3541231 | 20.28 | 1 |
| Silkie | Z | 57973076 | 57973674 | 14.68 | 0.52 | 0.05 | 1 | 1 | Z:57973076-57973674 | 23.23 | 1 |
| Silkie | Z | 61304879 | 61305992 | 10.56 | 0.99 | 1 | 1 | 0.98 | Z:61304819-61306021 | 32.04 | 1 |
| Silkie | Z | 69879377 | 69879901 | 13.01 | 0.52 | 0.05 | 1 | 1 | Z:69879377-69879901 | 16.37 | 1 |
| Silkie | Z | 79945533 | 79946432 | 48.65 | 1 | 1 | 1 | 1 | Z:79945501-79946432 | 46.67 | 1 |
| Taiwanese | 10 | 9703377 | 9703745 | 12.26 | 0.51 | 0.05 | 1 | 0.98 | 10:9703183-9703845 | 28.29 | 1 |
| Taiwanese | 11 | 18978787 | 18979081 | 10.82 | 0.52 | 0.05 | 1 | 1 | 11:18978682-18979268 | 40.74 | 1 |
| Taiwanese | 1 | 125858928 | 125859550 | 17.31 | 1 | 1 | 1 | 1 | 1:125858928-125859550 | 46.34 | 1 |
| Taiwanese | 1 | 140710619 | 140710862 | 15.64 | 1 | 1 | 1 | 0.99 | 1:140710345-140715423 | 92.9 | 1 |
| Taiwanese | 1 | 140714934 | 140715323 | 58.93 | 1 | 1 | 1 | 1 | 1:140710345-140715423 | 92.9 | 1 |
| Taiwanese | 1 | 140995850 | 140996513 | 25.53 | 1 | 1 | 1 | 1 | 1:140995796-140996700 | 27.9 | 1 |
| Taiwanese | 1 | 146731980 | 146732732 | 27.55 | 1 | 1 | 1 | 0.99 | 1:146731910-146732833 | 40.62 | 1 |
| Taiwanese | 1 | 146761661 | 146762103 | 9.61 | 0.52 | 0.05 | 1 | 1 | 1:146761527-146762190 | 45.2 | 1 |
| Taiwanese | 1 | 149162894 | 149163255 | 18.63 | 1 | 1 | 1 | 1 | 1:149162760-149166227 | 93.72 | 1 |
| Taiwanese | 1 | 149165149 | 149166035 | 13.67 | 1 | 1 | 1 | 1 | 1:149162760-149166227 | 93.72 | 1 |
| Taiwanese | 1 | 153879411 | 153880045 | 11.98 | 0.99 | 1 | 1 | 0.98 | 1:153879253-153880132 | 35.55 | 1 |
| Taiwanese | 1 | 179728795 | 179729315 | 14.22 | 0.52 | 0.05 | 1 | 1 | 1:179728775-179729463 | 45.94 | 1 |
| Taiwanese | 1 | 180747167 | 180747801 | 25.11 | 0.52 | 0.05 | 1 | 1 | 1:180746896-180747970 | 32.46 | 1 |
| Taiwanese | 1 | 182832952 | 182833960 | 10.18 | 0.99 | 1 | 1 | 0.98 | 1:182832847-182834082 | 48.3 | 1 |
| Taiwanese | 12 | 3240741 | 3240898 | 14.69 | 0.48 | 0.95 | 0 | 0 | 12:3240697-3240898 | 559.21 | 1 |
| Taiwanese | 12 | 3277776 | 3278316 | 45.26 | 1 | 1 | 1 | 0.99 | 12:3277416-3278316 | 283.77 | 1 |
| Taiwanese | 13 | 6261859 | 6262487 | 13.89 | 0.22 | 0 | 0 | 0.51 | 13:6261859-6262487 | 31.41 | 1 |
| Taiwanese | 1 | 37362120 | 37362477 | 11.97 | 0.51 | 0.05 | 1 | 0.99 | 1:37362031-37362653 | 30.13 | 1 |
| Taiwanese | 1 | 41318967 | 41319608 | 23.46 | 0.99 | 1 | 1 | 0.98 | 1:41318900-41319777 | 21.1 | 1 |
| Taiwanese | 1 | 46027902 | 46028388 | 7.49 | 0.51 | 0.05 | 1 | 0.98 | 1:46027902-46028388 | 26.59 | 1 |
| Taiwanese | 17 | 180844 | 181440 | 24.14 | 0.99 | 0.99 | 1 | 0.98 | 17:180636-181570 | 40.51 | 1 |
| Taiwanese | 1 | 73743848 | 73744438 | 11.09 | 1 | 1 | 1 | 1 | 1:73743848-73744438 | 32.49 | 1 |
| Taiwanese | 1 | 7919664 | 7919997 | 19.04 | 0.51 | 0.05 | 1 | 0.98 | 1:7919664-7919997 | 24.66 | 1 |
| Taiwanese | 20 | 7662075 | 7662516 | 15.38 | 1 | 1 | 1 | 1 | 20:7661897-7662786 | 24.76 | 1 |
| Taiwanese | 2 | 10512425 | 10512808 | 20.99 | 0.51 | 0.05 | 1 | 0.99 | 2:10512425-10512887 | 37.66 | 1 |
| Taiwanese | 2 | 142114005 | 142114475 | 16.29 | 0.51 | 0.05 | 1 | 0.98 | 2:142113866-142114543 | 43.02 | 1 |
| Taiwanese | 2 | 142512897 | 142513780 | 6.74 | 0.76 | 0.53 | 1 | 0.98 | 2:142512897-142513907 | 52.13 | 1 |
| Taiwanese | 2 | 51801229 | 51801824 | 15.33 | 0.99 | 0.99 | 1 | 0.99 | 2:51801055-51801954 | 32.98 | 1 |
| Taiwanese | 2 | 81965254 | 81965870 | 13.05 | 0.89 | 0.77 | 1 | 1 | 2:81965001-81966072 | 37.55 | 1 |
| Taiwanese | 2 | 82574536 | 82575418 | 11.48 | 1 | 1 | 1 | 1 | 2:82574297-82575518 | 38.36 | 1 |
| Taiwanese | 2 | 92857165 | 92858066 | 5.73 | 0.99 | 0.99 | 1 | 0.98 | 2:92856996-92858219 | 30.18 | 1 |
| Taiwanese | 2 | 95481137 | 95481700 | 13.12 | 1 | 1 | 1 | 0.99 | 2:95480957-95481836 | 20.79 | 1 |
| Taiwanese | 3 | 19822021 | 19822797 | 3.61 | 0.9 | 0.99 | 1 | 0.78 | 3:19821802-19823007 | 26.62 | 1 |
| Taiwanese | 3 | 34782953 | 34783236 | 7.87 | 0.51 | 0.05 | 1 | 0.98 | 3:34782953-34783379 | 41.84 | 1 |
| Taiwanese | 3 | 40908352 | 40908732 | 12.12 | 0.46 | 0 | 0.76 | 0.96 | 3:40908270-40908901 | 25.92 | 1 |
| Taiwanese | 3 | 49572247 | 49572841 | 18.69 | 1 | 1 | 1 | 1 | 3:49572068-49572931 | 31.47 | 1 |
| Taiwanese | 3 | 63745502 | 63745839 | 13.56 | 0.51 | 0.05 | 1 | 0.99 | 3:63745322-63745967 | 39.01 | 1 |
| Taiwanese | 3 | 73561990 | 73562865 | 1.39 | 0.98 | 0.97 | 1 | 0.99 | 3:73561879-73563101 | 31.27 | 1 |
| Taiwanese | 3 | 83407746 | 83408287 | 8.06 | 1 | 1 | 1 | 1 | 3:83407619-83408514 | 20.68 | 0.82 |
| Taiwanese | 3 | 83777155 | 83777799 | 4.71 | 0.43 | 0 | 0.29 | 0.96 | 3:83776894-83777945 | 35.21 | 1 |
| Taiwanese | 4 | 15864295 | 15864632 | 6.42 | 0.99 | 1 | 1 | 0.98 | 4:15864150-15864742 | 18.76 | 1 |
| Taiwanese | 4 | 26531003 | 26531815 | 4.72 | 0.47 | 0 | 0.76 | 0.98 | 4:26530893-26531990 | 31.14 | 1 |
| Taiwanese | 4 | 30595818 | 30596323 | 8.65 | 1 | 1 | 1 | 1 | 4:30595679-30596557 | 32.27 | 1 |
| Taiwanese | 4 | 70552105 | 70552594 | 22.73 | 0.99 | 1 | 1 | 0.98 | 4:70551829-70552669 | 24.4 | 1 |
| Taiwanese | 4 | 75154630 | 75154881 | 5.15 | 1 | 1 | 1 | 1 | 4:75154549-75155039 | 28.54 | 1 |
| Taiwanese | 4 | 78859444 | 78859746 | 11.56 | 0.51 | 0.05 | 1 | 0.99 | 4:78859384-78859987 | 25.67 | 1 |
| Taiwanese | 5 | 2705198 | 2706196 | 7.04 | 0.99 | 0.99 | 1 | 1 | 5:2705052-2706284 | 48.16 | 1 |
| Taiwanese | 5 | 31304004 | 31304796 | 2.68 | 0.84 | 0.84 | 0 | 0.97 | 5:31303866-31305068 | 23.76 | 1 |
| Taiwanese | 5 | 31898782 | 31899567 | 6.05 | 1 | 1 | 1 | 0.99 | 5:31898626-31899883 | 26.41 | 1 |
| Taiwanese | 5 | 31998288 | 31998903 | 9.24 | 0.99 | 1 | 1 | 0.98 | 5:31998141-31999034 | 22.8 | 1 |
| Taiwanese | 5 | 39163763 | 39164532 | 10.4 | 1 | 1 | 1 | 1 | 5:39163484-39164645 | 29.3 | 1 |
| Taiwanese | 5 | 40954947 | 40955227 | 14.31 | 0.51 | 0.05 | 1 | 0.99 | 5:40954947-40955227 | 33.61 | 1 |
| Taiwanese | 6 | 13725065 | 13725795 | 2.89 | 0.86 | 0.72 | 1 | 1 | 6:13724843-13726022 | 28.54 | 1 |
| Taiwanese | 6 | 25237488 | 25237870 | 18.33 | 0.51 | 0.05 | 1 | 0.99 | 6:25237263-25237909 | 39.97 | 1 |
| Taiwanese | 6 | 9811321 | 9811705 | 24.39 | 1 | 0.99 | 1 | 1 | 6:9811191-9816444 | 56.38 | 0.97 |
| Taiwanese | 6 | 9815556 | 9816224 | 2.07 | 0.99 | 0.99 | 1 | 0.98 | 6:9811191-9816444 | 56.38 | 0.97 |
| Taiwanese | 7 | 9022836 | 9023269 | 33.8 | 0.51 | 0.05 | 1 | 0.99 | 7:9022731-9023457 | 39.03 | 1 |
| Taiwanese | AADN03018735.1 | 341 | 1207 | 17.33 | 0.99 | 0.99 | 1 | 0.98 | AADN03018735.1:281-1293 | 45.88 | 1 |
| Taiwanese | AADN03024906.1 | 3482 | 4128 | 1 | 0.21 | 0 | 0 | 0.48 | AADN03024906.1:3470-4351 | 23.22 | 1 |
| Taiwanese | JH375231.1 | 4876 | 5708 | 3.18 | 0.4 | 0 | 0.47 | 0.87 | JH375231.1:4846-8915 | 22.53 | 1 |
| Taiwanese | JH375231.1 | 8096 | 8863 | NA | NA | NA | NA | NA | JH375231.1:4846-8915 | 22.53 | 1 |
| Taiwanese | JH375237.1 | 49865 | 50591 | NA | NA | NA | NA | NA | JH375237.1:49865-50701 | 22.89 | 1 |
| Taiwanese | Z | 15471067 | 15471485 | 7 | 0.99 | 1 | 1 | 0.98 | Z:15470956-15471752 | 12.19 | 1 |
| Taiwanese | Z | 18808172 | 18808900 | 1 | 0.22 | 0 | 0 | 0.52 | Z:18807991-18809115 | 21.93 | 1 |
| Taiwanese | Z | 30996666 | 30996912 | 43.94 | 1 | 1 | 1 | 1 | Z:30996146-31000811 | 57.7 | 1 |
| Taiwanese | Z | 32081339 | 32081656 | 39.08 | 1 | 1 | 1 | 1 | Z:32081154-32086293 | 78.78 | 1 |
| Taiwanese | Z | 32085820 | 32086072 | 13.03 | 1 | 1 | 1 | 0.99 | Z:32081154-32086293 | 78.78 | 1 |
| Taiwanese | Z | 47227949 | 47228506 | 15.31 | 1 | 1 | 1 | 1 | Z:47227949-47228506 | 17.36 | 1 |
| Taiwanese | Z | 61305039 | 61305809 | 12.22 | 0.95 | 0.91 | 1 | 1 | Z:61304819-61306021 | 17.43 | 1 |
| Taiwanese | Z | 79945656 | 79946254 | 13.34 | 1 | 1 | 1 | 1 | Z:79945501-79946432 | 19.78 | 1 |

  


---

  


# Supplementary Table 3: *S3*

| S3. Summary of integration site interval per bird/line, indicating presence (Y) / absence (N) / not determined (NA) followed by mean depth of coverage at interval | | | | | | | | | | | | | | | | | | | | | | | | | | | | | | |  | S3a. Frequency of intervals | | |  |
|  |  |  |  |  |  |  |  |  |  |  |  |  |  |  |  |  |  |  |  |  |  |  |  |  |  |  |  |  |  |  |  |  |  |  |  |
| **Chr** | **Start** | **End** | **HA1A22A** | **HA1B25B** | **HA2A10B** | **HA2A25B** | **HB1A16A** | **HB1B21B** | **JA1A17A** | **JA2A10B** | **JB2A04B.1** | **JB2A04B.2** | **JB1A25B** | **JB1B16A** | **Line15** | **Line6** | **Line7** | **LineC** | **LineN** | **LineP** | **LineZero** | **Wellcome** | **RJF** | **Silkie** | **Taiwanese** | **Total** | **Global frequency** | **Horro frequency** | **Jarso frequency** | **Pirbright frequency** |  | **Frequency (f)** | **Count (n ≥ f)** | **Fraction** |  |
| 1 | 7414982 | 7415621 | N:29.85 | N:26.2 | N:30.37 | N:28.39 | N:30.52 | N:31.07 | N:29.32 | Y:28.68 | N:23.51 | N:22.41 | N:31.42 | N:30.49 | N:11.07 | N:16.22 | N:13.05 | N:15.48 | N:11.81 | N:19.56 | N:15.1 | N:13.43 | N:68.87 | N:24.18 | N:30 | 1 | 0.04 | 0 | 0.17 | 0 |  | 0 | 297 | 1 |  |
| 1 | 7919664 | 7919997 | N:33.36 | N:30.6 | N:29.63 | N:31.28 | N:28.99 | N:35.42 | N:30.21 | N:35.84 | N:31.59 | N:28.11 | N:28.25 | N:32.17 | N:8.43 | N:15.47 | N:12.63 | N:13.4 | N:11.11 | N:21.17 | N:12.58 | N:19.22 | N:63.75 | N:16.79 | Y:24.66 | 1 | 0.04 | 0 | 0 | 0 |  | 0.1 | 150 | 0.51 |  |
| 1 | 8127806 | 8128614 | N:27.43 | Y:23.47 | Y:31.33 | Y:31.07 | N:28.56 | N:28.35 | N:25.2 | N:26.97 | N:22.47 | N:28.72 | N:26.98 | N:27.73 | N:15.76 | N:21.69 | N:22.69 | N:16.49 | N:12.28 | N:20.72 | N:19.03 | N:22.6 | N:50.78 | N:39.63 | N:52.97 | 3 | 0.13 | 0.5 | 0 | 0 |  | 0.2 | 105 | 0.35 |  |
| 1 | 15904038 | 15904160 | N:42.61 | N:49.25 | N:36.35 | N:27.44 | N:33.67 | N:28.67 | N:15.67 | N:27.72 | N:12.49 | N:16.2 | N:31.71 | N:32.35 | N:18.6 | Y:22.44 | N:8.54 | N:3.42 | N:2.13 | N:16.15 | Y:20.34 | N:20.52 | N:18.55 | N:30.23 | N:70.8 | 2 | 0.09 | 0 | 0 | 0.25 |  | 0.3 | 81 | 0.27 |  |
| 1 | 18836195 | 18836791 | N:29.47 | N:25.83 | N:25.66 | N:31.45 | N:28.89 | N:27.43 | N:24.82 | N:28.15 | N:28.71 | N:27.86 | N:28.21 | N:32.82 | Y:10.47 | N:25.23 | N:31.48 | N:19.55 | N:12.69 | N:29.47 | N:19.2 | N:20.26 | N:62.35 | N:39.76 | N:42.17 | 1 | 0.04 | 0 | 0 | 0.13 |  | 0.4 | 56 | 0.19 |  |
| 1 | 21322138 | 21322493 | N:17.32 | N:33.18 | N:20.63 | N:40.21 | N:29.74 | N:36.14 | N:30.32 | N:38.1 | N:31.53 | N:27.72 | N:30.83 | N:28.49 | N:6.97 | N:10.66 | N:20.3 | N:16.6 | N:12.72 | N:20.29 | N:11.85 | Y:14.33 | N:62.97 | N:18.08 | N:25.85 | 1 | 0.04 | 0 | 0 | 0.13 |  | 0.5 | 41 | 0.14 |  |
| 1 | 23890739 | 23891336 | N:34.2 | N:31.43 | N:34.35 | N:30.7 | N:36.38 | N:35.32 | N:32.07 | N:33.72 | N:32.58 | N:28.94 | N:33.63 | N:32.62 | N:10.4 | N:17.07 | N:19.72 | N:18.84 | N:13.45 | N:21.01 | N:17.11 | Y:20.8 | N:60.74 | N:29 | N:31.67 | 1 | 0.04 | 0 | 0 | 0.13 |  | 0.6 | 36 | 0.12 |  |
| 1 | 28836094 | 28836942 | N:30 | N:28.61 | N:31.07 | N:34.9 | N:33.82 | Y:46.62 | N:28.73 | N:28.13 | N:28.53 | N:26.13 | N:31.66 | N:31.71 | N:9.35 | N:15.48 | N:14.58 | N:15.91 | N:14.09 | N:21.37 | N:16.1 | N:17.3 | N:65.49 | N:24 | N:28.31 | 1 | 0.04 | 0.17 | 0 | 0 |  | 0.7 | 26 | 0.09 |  |
| 1 | 31728916 | 31729579 | Y:29.21 | N:29.61 | Y:29.53 | N:33.14 | N:32.1 | N:33.31 | N:35.35 | N:34.53 | N:34.04 | N:23.37 | Y:29.66 | N:32.79 | N:8.6 | N:16.76 | Y:18 | N:17.86 | N:14.16 | N:28.91 | N:16.35 | N:22.78 | N:55.43 | N:27.48 | N:34.23 | 4 | 0.17 | 0.33 | 0.17 | 0.13 |  | 0.8 | 20 | 0.07 |  |
| 1 | 32130327 | 32131074 | N:36.3 | N:32.18 | N:36.03 | N:31.8 | N:34.28 | N:36.68 | N:36.64 | N:38.01 | N:33.45 | N:32.84 | N:33.08 | N:33.92 | N:7.9 | N:12.08 | N:10.53 | N:15.04 | N:13.23 | N:14.52 | N:11.36 | N:12.46 | N:64.97 | Y:11.9 | N:18.69 | 1 | 0.04 | 0 | 0 | 0 |  | 0.9 | 14 | 0.05 |  |
| 1 | 32312218 | 32313185 | Y:28.36 | Y:26.21 | Y:28.71 | Y:30 | N:27.34 | Y:25.59 | Y:28.95 | Y:28.7 | Y:27.61 | Y:22.3 | Y:26.08 | Y:25.21 | Y:8.16 | Y:10.35 | Y:13.15 | Y:13.03 | Y:11.1 | Y:16.35 | Y:12.28 | N:12.65 | N:61.68 | Y:10.72 | N:23.09 | 19 | 0.83 | 0.83 | 1 | 0.88 |  | 1 | 8 | 0.03 |  |
| 1 | 32508365 | 32508761 | N:34.11 | N:35.13 | N:37.14 | N:32.59 | N:33.59 | N:37.88 | N:31.61 | Y:35.42 | Y:33.53 | Y:25.77 | N:34.9 | Y:31.22 | N:6.2 | N:17.85 | N:14.4 | N:12.07 | N:8.34 | N:26.83 | Y:11.48 | N:13.33 | N:56.85 | N:18.88 | N:24.01 | 5 | 0.22 | 0 | 0.67 | 0.13 |  |  |  |  |  |
| 1 | 34786290 | 34786522 | Y:29.05 | N:29.43 | N:34.44 | N:32.51 | N:28.45 | N:31.79 | N:29.28 | N:28.3 | N:33.92 | N:25.7 | N:30 | N:32.3 | N:10.26 | N:21.75 | N:24.79 | N:21.23 | N:12.77 | N:24.03 | N:15.7 | N:16.62 | N:74.24 | N:36.03 | N:33.58 | 1 | 0.04 | 0.17 | 0 | 0 |  |  |  |  |  |
| 1 | 37362031 | 37362653 | N:29.39 | N:28.85 | N:37.63 | N:32.35 | Y:31 | N:35.7 | N:38.64 | N:33.89 | N:31.48 | N:23.81 | N:31.75 | N:30.94 | N:10.04 | N:13.15 | N:14.29 | N:13.15 | N:13.52 | Y:17.68 | Y:17.88 | N:14.88 | N:72.32 | N:14.88 | Y:30.13 | 4 | 0.17 | 0.17 | 0 | 0.25 |  | S3b. Distribution of intervals | | |  |
| 1 | 41194458 | 41195110 | Y:29.71 | N:35.34 | N:37.66 | N:33.54 | N:33.59 | N:36.12 | N:34.18 | N:35.87 | N:34.22 | N:33.5 | N:32.35 | N:34.1 | N:9.24 | N:17.33 | N:17.8 | N:16.81 | N:14.76 | Y:27.11 | N:15.83 | N:16.25 | N:62.59 | N:22.43 | N:25.22 | 2 | 0.09 | 0.17 | 0 | 0.13 |  |  |  |  |  |
| 1 | 41318900 | 41319777 | N:31.97 | N:31.03 | Y:34.4 | Y:34.54 | N:31.36 | N:37.1 | Y:32.99 | Y:30.61 | Y:30.1 | Y:24.81 | N:30.31 | N:29.92 | Y:9.1 | Y:12.97 | N:16.08 | Y:14.86 | Y:9.84 | Y:21.46 | Y:10.52 | Y:16.5 | N:64.77 | N:20.71 | Y:21.1 | 14 | 0.61 | 0.33 | 0.67 | 0.88 |  | **Chr** | **Count** |  |  |
| 1 | 42541895 | 42542614 | N:31.61 | Y:32.01 | Y:32.17 | N:29.35 | Y:29.85 | Y:34.25 | N:35.23 | N:30.2 | N:29.83 | N:25.2 | N:33.03 | Y:34.89 | N:11.69 | Y:15.93 | Y:17.72 | N:18.93 | N:11.91 | N:28.81 | Y:19.03 | Y:15.53 | N:60.57 | Y:22.82 | N:32.45 | 10 | 0.43 | 0.67 | 0.17 | 0.5 |  | **1** | 83 |  |  |
| 1 | 43882636 | 43883815 | N:34.55 | N:32.74 | Y:35.97 | N:33.46 | N:28.23 | N:34.79 | N:32.3 | N:38.44 | N:34.94 | N:23.69 | N:33.39 | N:29.72 | N:3.94 | Y:12.88 | N:9.89 | Y:16.67 | Y:11.51 | Y:18.6 | N:8.77 | N:8.47 | Y:65.85 | N:11.45 | N:15.4 | 6 | 0.26 | 0.17 | 0 | 0.5 |  | **2** | 47 |  |  |
| 1 | 44606699 | 44607552 | N:37.84 | N:33.21 | N:29.66 | N:27.61 | N:36.07 | N:29.75 | N:29.92 | N:29.89 | N:31.5 | N:24.86 | Y:29.25 | N:26.72 | N:11.67 | N:18.97 | N:18.45 | N:19.65 | N:9.68 | N:24.13 | N:15.82 | N:15.27 | N:60.49 | N:20.8 | N:34.32 | 1 | 0.04 | 0 | 0.17 | 0 |  | **3** | 34 |  |  |
| 1 | 46027902 | 46028388 | N:34.3 | N:29.1 | N:28.24 | N:32.94 | N:35.7 | N:29.37 | N:30.55 | N:26.11 | N:30.1 | N:27.87 | N:30.85 | N:27.51 | N:8.15 | N:17.74 | N:19.15 | N:24.52 | N:12.3 | N:20.63 | N:17.81 | N:16.41 | N:75.76 | N:27.88 | Y:26.59 | 1 | 0.04 | 0 | 0 | 0 |  | **4** | 32 |  |  |
| 1 | 47364771 | 47365611 | N:29.78 | N:32.02 | Y:30.25 | N:31.8 | N:30.63 | N:32.85 | N:28.91 | N:29.98 | N:30.91 | N:24.68 | N:30.85 | N:27.15 | N:8.87 | N:10.75 | N:17.1 | N:8.48 | N:9.14 | N:16.34 | N:10.04 | N:13.91 | N:57.45 | N:16.85 | N:25.41 | 1 | 0.04 | 0.17 | 0 | 0 |  | **5** | 16 |  |  |
| 1 | 52743902 | 52744500 | N:31.93 | N:33.92 | N:33.44 | N:35.26 | N:38.02 | N:36.02 | N:32.54 | N:36.82 | N:32.84 | N:28.95 | N:35.76 | N:30.63 | N:9.34 | N:12.19 | N:16 | N:16.62 | N:9.63 | Y:21.15 | N:12.9 | N:13.34 | N:62.11 | Y:16.83 | N:19.95 | 2 | 0.09 | 0 | 0 | 0.13 |  | **6** | 7 |  |  |
| 1 | 63571454 | 63572079 | Y:28.51 | N:25.3 | N:34.32 | N:28.86 | N:31.72 | Y:34.2 | N:26.82 | N:26.34 | N:31.4 | N:19.74 | N:28.51 | N:28.61 | Y:5.16 | N:11.04 | Y:16.35 | N:17.65 | N:10.15 | Y:16.5 | Y:12.5 | Y:12.64 | N:57.27 | N:12.01 | N:25.56 | 7 | 0.3 | 0.33 | 0 | 0.63 |  | **7** | 8 |  |  |
| 1 | 67550436 | 67551166 | N:35.03 | N:26.88 | N:36.59 | N:30.53 | N:27.78 | Y:32.94 | N:31.81 | Y:38.79 | N:26.22 | N:25.77 | N:27.57 | Y:35.56 | Y:19.83 | Y:25.26 | Y:36.42 | Y:29.64 | N:12.33 | Y:31.52 | Y:30.15 | Y:25.99 | N:63.94 | N:49.81 | N:46.66 | 10 | 0.43 | 0.17 | 0.33 | 0.88 |  | **8** | 5 |  |  |
| 1 | 70454672 | 70455338 | N:36.93 | N:31.08 | N:35.89 | N:38.86 | N:25.69 | N:32.3 | N:30.66 | N:28.4 | Y:26.38 | Y:23.75 | N:29.43 | Y:31.31 | N:13.73 | N:19.54 | N:21.87 | N:19.99 | N:11.94 | N:27.45 | N:17.56 | N:20.13 | N:63.38 | N:31.99 | N:35.52 | 3 | 0.13 | 0 | 0.5 | 0 |  | **9** | 4 |  |  |
| 1 | 73743848 | 73744438 | N:30.15 | N:27.28 | N:25.71 | N:32.06 | N:29.58 | N:23.4 | N:30.29 | N:31.7 | N:31.53 | N:25.34 | N:35.38 | N:28.08 | N:8.14 | N:21.77 | N:18.16 | N:20.14 | N:13.06 | N:23.83 | N:19.41 | N:20.12 | N:58.33 | N:30.7 | Y:32.49 | 1 | 0.04 | 0 | 0 | 0 |  | **10** | 1 |  |  |
| 1 | 77444595 | 77445260 | N:18.81 | N:28.52 | N:35.83 | N:22.6 | N:15.47 | N:17.1 | NA | N:30.36 | NA | NA | N:18.76 | N:16.72 | N:1.55 | N:15.39 | N:19.97 | N:10.94 | NA | N:25.27 | N:14.11 | N:17.02 | N:72.94 | Y:31.23 | N:11.33 | 1 | 0.05 | 0 | 0 | 0 |  | **11** | 3 |  |  |
| 1 | 84555430 | 84556039 | N:34.87 | N:33.37 | N:36.34 | N:33.78 | N:28.19 | N:33.67 | N:35.01 | Y:32.23 | N:37.02 | N:27.52 | N:35.21 | N:30.17 | N:6.26 | N:10.29 | N:12.89 | N:14.02 | N:7.17 | N:16.99 | N:7.89 | N:10.51 | N:59.31 | Y:14.65 | N:21.24 | 2 | 0.09 | 0 | 0.17 | 0 |  | **12** | 6 |  |  |
| 1 | 87425101 | 87425826 | N:35.39 | N:31.25 | N:28.18 | N:29.83 | N:29.6 | N:35.73 | N:29.54 | Y:32.9 | N:29.27 | N:24.56 | N:29.97 | N:23.16 | N:8.69 | N:11.36 | N:11.38 | N:15.66 | N:10.79 | N:20.35 | Y:12.43 | N:11.2 | N:55.55 | N:10.5 | N:22.13 | 2 | 0.09 | 0 | 0.17 | 0.13 |  | **13** | 6 |  |  |
| 1 | 89899733 | 89900560 | N:34.88 | N:34.27 | N:43.45 | Y:32.27 | N:29.57 | Y:29.4 | N:36.04 | N:38.94 | N:36.18 | N:24.62 | N:34.68 | N:34.05 | N:9.69 | N:10.67 | N:13.86 | N:14.51 | N:12.19 | N:19.7 | N:11.07 | N:14.21 | N:71.51 | Y:18.45 | N:18.7 | 3 | 0.13 | 0.33 | 0 | 0 |  | **14** | 2 |  |  |
| 1 | 90099919 | 90100600 | N:29.28 | N:30.64 | N:27.3 | N:27.71 | N:28.72 | N:26.57 | N:28.97 | N:25.65 | N:24.63 | N:20.16 | N:26.98 | Y:23.96 | N:9.81 | N:17.38 | N:19.6 | N:14.8 | N:12.37 | N:21.22 | Y:14.97 | N:20.59 | N:50.95 | N:38.42 | N:33.79 | 2 | 0.09 | 0 | 0.17 | 0.13 |  | **15** | 1 |  |  |
| 1 | 93473773 | 93474297 | N:20.87 | N:25.56 | N:24 | N:26 | N:21.65 | N:20.51 | N:19.54 | N:20.27 | N:25.36 | N:13.98 | N:16.15 | N:22.58 | N:9.2 | N:4.71 | N:9.18 | N:8.15 | N:7.8 | N:12.39 | N:6.91 | N:11.19 | N:56.34 | Y:15.4 | N:22.42 | 1 | 0.04 | 0 | 0 | 0 |  | **16** | 0 |  |  |
| 1 | 95854597 | 95855224 | N:32.85 | N:32.6 | N:31.7 | N:37.15 | N:36.54 | N:35.48 | N:36.29 | N:36.75 | Y:31.13 | Y:29.75 | N:29.6 | N:31.11 | N:6.02 | N:17.1 | N:21.34 | N:12.41 | N:12.66 | N:20.84 | N:12.87 | N:14.25 | N:57.36 | N:18.76 | N:23.01 | 2 | 0.09 | 0 | 0.33 | 0 |  | **17** | 1 |  |  |
| 1 | 96156897 | 96157369 | Y:30.74 | N:26.25 | N:36.76 | N:33.47 | N:29.81 | N:35.24 | N:35.5 | N:35.2 | Y:36.91 | Y:29.9 | N:27.93 | N:32.21 | N:8.25 | N:15.27 | N:15.83 | N:13.11 | N:11.29 | N:21.39 | N:8.03 | N:12.91 | Y:67.81 | N:17.97 | N:22.05 | 4 | 0.17 | 0.17 | 0.33 | 0 |  | **18** | 0 |  |  |
| 1 | 96182372 | 96182610 | N:27.6 | N:35.87 | N:35.69 | N:34.63 | N:36.5 | Y:33.65 | N:36.98 | N:46.34 | N:31.45 | N:25.1 | N:29.57 | Y:32.13 | N:8.2 | Y:15.37 | Y:16.06 | Y:14.62 | N:7.19 | Y:19.67 | Y:10.19 | N:11.47 | N:51.14 | N:14.27 | N:25.91 | 7 | 0.3 | 0.17 | 0.17 | 0.63 |  | **19** | 1 |  |  |
| 1 | 99050515 | 99051202 | N:35.82 | Y:30.13 | N:37.08 | N:39.28 | N:32.7 | N:37.86 | N:37.88 | N:32.59 | Y:27.66 | Y:25.48 | N:32.18 | N:37.79 | N:7.76 | Y:14.29 | Y:16.66 | Y:13.84 | N:12.46 | N:12.58 | Y:12.48 | Y:16.52 | N:60.02 | N:26.6 | N:27.97 | 8 | 0.35 | 0.17 | 0.33 | 0.63 |  | **20** | 3 |  |  |
| 1 | 99136310 | 99136953 | N:33.16 | Y:25.25 | Y:37.5 | Y:34.03 | Y:35.3 | N:31.89 | N:36.75 | N:27.5 | Y:27.12 | Y:21.61 | Y:31.1 | Y:29.89 | Y:13.37 | N:20.87 | N:18.28 | N:21.25 | N:12.4 | N:20.88 | N:23.64 | Y:17.2 | N:66.25 | N:25.58 | N:25.94 | 10 | 0.43 | 0.67 | 0.67 | 0.25 |  | **21** | 0 |  |  |
| 1 | 101165329 | 101166193 | N:33.55 | N:33.92 | N:33 | N:34.61 | N:34.58 | N:32.86 | N:34.43 | N:31.54 | N:32.31 | N:25.97 | N:31.95 | N:34.58 | N:8.28 | N:12.09 | Y:17.77 | N:15.75 | N:8.81 | N:22.22 | Y:13.78 | N:17.45 | N:65.72 | N:18.11 | N:24.55 | 2 | 0.09 | 0 | 0 | 0.25 |  | **22** | 0 |  |  |
| 1 | 101333491 | 101334404 | N:33.04 | Y:29 | N:31.91 | N:29.94 | N:28.91 | N:32.1 | N:26.54 | Y:30.28 | Y:27.73 | Y:22.7 | N:23.31 | N:30.24 | N:9.4 | N:17.2 | N:13.89 | Y:16.83 | N:9.92 | Y:20.13 | N:13.5 | N:16.28 | N:56.11 | Y:27.12 | N:34.67 | 7 | 0.3 | 0.17 | 0.5 | 0.25 |  | **23** | 0 |  |  |
| 1 | 113945460 | 113946362 | N:30.54 | Y:31.04 | Y:37.16 | Y:34.32 | Y:35.81 | N:34.44 | N:35.9 | N:31.86 | N:31.44 | N:30.1 | Y:30.9 | Y:31.68 | N:10.33 | N:10.13 | N:16.02 | Y:19.14 | N:11.15 | Y:20.69 | N:11.52 | Y:13.78 | N:53.58 | N:15.85 | N:20.98 | 9 | 0.39 | 0.67 | 0.33 | 0.38 |  | **24** | 1 |  |  |
| 1 | 114080840 | 114081279 | N:34.35 | N:32.49 | N:36.85 | N:32.77 | N:32.73 | N:31.34 | N:34.08 | N:36.35 | N:30.55 | N:26.63 | N:31 | N:29.62 | N:9.89 | N:12.8 | N:19.02 | N:13.53 | N:15.37 | N:21.44 | N:14.52 | N:20.33 | N:62.62 | Y:26.9 | N:15.32 | 1 | 0.04 | 0 | 0 | 0 |  | **25** | 0 |  |  |
| 1 | 117166631 | 117167523 | N:34.94 | Y:31.17 | N:36.66 | N:33.46 | N:34.05 | N:38.15 | N:34.18 | Y:31.8 | N:32.04 | N:27.76 | N:30.64 | N:32.26 | N:11.5 | N:17.6 | N:18.02 | N:15.08 | N:11.97 | N:19.6 | N:13.92 | N:24.84 | N:66.32 | N:21.36 | N:31.08 | 2 | 0.09 | 0.17 | 0.17 | 0 |  | **26** | 0 |  |  |
| 1 | 123156456 | 123157321 | Y:25.44 | N:28.78 | Y:28.48 | Y:31.01 | N:31.15 | N:31.37 | N:30.24 | N:28.83 | N:32.55 | N:25.52 | N:32.31 | N:32.28 | N:7.21 | N:13.21 | N:46.7 | N:14.05 | N:11.97 | N:18.92 | N:12.07 | N:16.17 | N:61.36 | N:18.23 | N:26.83 | 3 | 0.13 | 0.5 | 0 | 0 |  | **27** | 0 |  |  |
| 1 | 125858928 | 125859550 | N:33.49 | N:31.26 | N:35.15 | N:31.4 | N:30.03 | N:33.77 | N:30.78 | N:33.53 | N:29.34 | N:27.97 | N:29.52 | N:28.07 | N:8.1 | N:21.6 | N:27.82 | N:22.49 | N:18.19 | N:35.97 | N:19.55 | N:22.09 | N:71.31 | N:48.75 | Y:46.34 | 1 | 0.04 | 0 | 0 | 0 |  | **28** | 0 |  |  |
| 1 | 126794615 | 126795484 | N:31.96 | N:30.34 | N:33.96 | N:33.82 | N:36.02 | N:35.61 | N:33.09 | N:33.8 | Y:31.68 | Y:27.89 | N:32.88 | Y:30.14 | N:9.77 | N:12.96 | Y:15.29 | N:15.43 | Y:11.02 | Y:18.84 | N:10.89 | N:13.71 | N:53.93 | N:12.01 | N:27.73 | 6 | 0.26 | 0 | 0.5 | 0.38 |  | **Z** | 18 |  |  |
| 1 | 127188644 | 127189303 | N:32.43 | N:26.7 | N:25.7 | N:28.6 | N:31.09 | N:33.9 | N:29.49 | N:26.14 | N:26.77 | N:20.31 | N:31.51 | N:26.7 | N:12.74 | N:17.22 | N:20.1 | Y:18.26 | N:11.38 | N:25.37 | N:20.4 | N:17.55 | N:47.23 | N:36.89 | N:31.35 | 1 | 0.04 | 0 | 0 | 0.13 |  | **Unplaced ['A...']** | 10 |  |  |
| 1 | 131477317 | 131477931 | N:35.31 | N:34.2 | N:36.75 | N:31.27 | N:33.44 | N:32.4 | N:33.04 | Y:30.93 | N:34.9 | N:30.39 | N:33.89 | N:35.18 | N:8.42 | N:16.04 | N:14.52 | N:17.69 | N:9.29 | N:26.15 | N:9.62 | N:20.28 | N:72.15 | N:21.91 | N:31.24 | 1 | 0.04 | 0 | 0.17 | 0 |  | **Unplaced ['J...']** | 8 |  |  |
| 1 | 140037593 | 140038154 | N:33.63 | N:35.85 | N:34.98 | Y:32.08 | N:34.79 | N:40.57 | N:33.98 | N:36.94 | N:42.96 | N:30.9 | N:33.79 | N:29.35 | N:6.66 | N:12.85 | N:12.06 | N:12.26 | N:11.43 | N:18.59 | N:14.85 | N:8.79 | N:65.15 | N:10.19 | N:20.21 | 1 | 0.04 | 0.17 | 0 | 0 |  | **Macro** | 0.714 |  |  |
| 1 | 140710345 | 140715423 | Y:43.38 | Y:42.42 | Y:45.32 | Y:48.92 | Y:52.86 | Y:49.22 | Y:41.69 | Y:45.46 | Y:41.19 | Y:30.29 | Y:45.08 | Y:30.71 | Y:23.37 | Y:38.64 | Y:39.61 | Y:23.95 | Y:15.54 | Y:52.61 | Y:24.97 | Y:38.06 | Y:59.83 | Y:65.17 | Y:92.9 | 23 | 1 | 1 | 1 | 1 |  | **Inter** | 0.084 |  |  |
| 1 | 140995796 | 140996700 | N:20.7 | N:21.48 | N:23.91 | N:27.95 | N:27.31 | N:25.03 | Y:22.3 | N:23.28 | N:21.68 | N:22.26 | N:25.58 | N:26.8 | N:9.57 | N:19.79 | N:22.97 | N:17.74 | N:10.89 | N:18.76 | N:16.13 | N:12.47 | N:56.05 | N:21.49 | Y:27.9 | 2 | 0.09 | 0 | 0.17 | 0 |  | **Micro** | 0.081 |  |  |
| 1 | 141097673 | 141098554 | N:33.13 | N:32.93 | N:37.72 | Y:32.57 | N:34.36 | N:35.15 | N:35.82 | N:37.97 | N:35.82 | N:27.5 | Y:35.82 | N:31.69 | N:12.13 | N:16.84 | N:20.19 | N:16.3 | N:13.61 | N:20.05 | N:14.41 | N:14.94 | N:69.77 | Y:23.09 | N:26.78 | 3 | 0.13 | 0.17 | 0.17 | 0 |  | **Z** | 0.061 |  |  |
| 1 | 142068256 | 142068910 | N:35.35 | N:37.08 | N:33.84 | N:35.87 | Y:30.67 | N:35.02 | N:34 | N:34.54 | N:34.06 | N:30.13 | N:34.78 | N:34.76 | N:9.95 | N:18.98 | N:15.96 | N:19.44 | N:9.41 | N:24.15 | N:15.29 | N:18.22 | N:61.47 | N:19.15 | N:30.66 | 1 | 0.04 | 0.17 | 0 | 0 |  | **Unplaced** | 0.061 |  |  |
| 1 | 146731910 | 146732833 | N:28.18 | Y:28.61 | Y:30.98 | Y:31.09 | Y:31.29 | Y:33.79 | Y:28.9 | Y:26.45 | Y:27.6 | Y:25.16 | Y:29.79 | Y:29.03 | Y:9.95 | N:20.62 | N:18.73 | Y:17.7 | Y:9.11 | Y:22.69 | Y:17.26 | Y:16.42 | N:49.4 | N:32.33 | Y:40.62 | 18 | 0.78 | 0.83 | 1 | 0.75 |  |  |  |  |  |
| 1 | 146761527 | 146762190 | N:31.56 | Y:29.75 | N:30.08 | N:30.75 | N:29.27 | N:28.66 | N:31.37 | N:30.34 | N:30.11 | N:22.67 | Y:32.66 | Y:29.91 | N:10.26 | N:15.42 | N:14.57 | N:13.25 | N:9.93 | Y:18.38 | Y:13.37 | Y:15.77 | N:55.47 | N:21.49 | Y:45.2 | 7 | 0.3 | 0.17 | 0.33 | 0.38 |  |  |  |  |  |
| 1 | 148564562 | 148568615 | N:38.39 | N:43.69 | N:40.83 | N:40.76 | Y:51.66 | N:37.16 | Y:31.35 | Y:44.68 | N:33.23 | N:23.07 | N:37.02 | Y:40.72 | N:11.3 | N:14.56 | N:29.19 | N:18.91 | N:7.14 | N:19.47 | N:19.54 | N:17.65 | Y:86.26 | N:40.78 | N:43.3 | 5 | 0.22 | 0.17 | 0.5 | 0 |  |  |  |  |  |
| 1 | 149162760 | 149166227 | N:33.17 | Y:47.75 | Y:41.72 | N:32.83 | N:36.27 | N:39.58 | N:29.24 | Y:38.9 | Y:32.94 | Y:29.29 | N:32.71 | N:28.17 | N:21.03 | N:23.6 | N:20.13 | N:22.57 | N:15.89 | Y:52.34 | Y:19.02 | Y:55.77 | Y:60.07 | Y:62.19 | Y:93.72 | 11 | 0.48 | 0.33 | 0.5 | 0.38 |  | S3c. Mean interval sites per chromosome per chicken 'type' | | | |
| 1 | 149501163 | 149502040 | N:31.65 | Y:31.85 | N:37.13 | N:33.51 | N:33.59 | N:38 | N:37.23 | N:39.94 | N:36.12 | N:30.27 | N:33.27 | N:30.14 | N:10 | N:14.06 | N:23.96 | N:13.83 | N:13.27 | N:23.54 | N:13.05 | N:15.49 | N:65.08 | N:18.39 | N:22.12 | 1 | 0.04 | 0.17 | 0 | 0 |  |  |  |  |  |
| 1 | 149935105 | 149935762 | N:33.27 | Y:28.68 | N:36.99 | N:36.89 | N:33.13 | N:40.34 | N:35.45 | N:37.86 | N:32.16 | N:26.41 | N:29.82 | N:31.09 | N:7.8 | N:12.36 | N:17.56 | N:21.01 | Y:11.83 | Y:18.59 | N:12.71 | N:16.45 | N:63.75 | N:14.27 | N:24.95 | 3 | 0.13 | 0.17 | 0 | 0.25 |  | **Chr** | **Ethiopian** | **Pirbright** | **SRA** |
| 1 | 150131206 | 150132006 | N:35.26 | N:30.69 | N:37.43 | N:30.01 | N:33.51 | N:35.56 | N:36.1 | N:32.34 | N:32.66 | N:28.2 | N:35.97 | Y:33.6 | N:10.03 | N:18.79 | N:26.51 | N:18.66 | N:17.27 | N:21.67 | N:17.13 | N:19.94 | N:63.84 | N:31.58 | N:38.43 | 1 | 0.04 | 0 | 0.17 | 0 |  | **1** | 18 | 17.13 | 15.5 |
| 1 | 151384845 | 151385716 | N:33 | Y:31.1 | N:32.59 | N:31.28 | N:37.47 | N:31.88 | N:32.1 | N:36.1 | N:30.9 | N:22.56 | N:32.34 | N:32.28 | N:9.97 | N:16.44 | N:25.82 | N:23.33 | N:16.13 | N:23.98 | N:15.25 | N:18.51 | N:58.5 | N:24.22 | N:26.32 | 1 | 0.04 | 0.17 | 0 | 0 |  | **2** | 9.92 | 10.75 | 6.5 |
| 1 | 151407118 | 151410307 | N:15.81 | N:22.66 | N:20.1 | N:23.11 | N:19.42 | N:16.46 | N:15.57 | N:14.47 | N:15.58 | N:12.09 | Y:16.92 | N:15.35 | N:7.46 | N:12.14 | N:22.69 | N:12.88 | N:7.87 | N:13.86 | N:15.42 | N:16.11 | Y:61.5 | N:24.92 | N:19.93 | 2 | 0.09 | 0 | 0.17 | 0 |  | **3** | 10.33 | 7.88 | 9 |
| 1 | 153879253 | 153880132 | N:31.78 | N:31.86 | N:29.17 | N:29.85 | N:29.05 | N:31 | N:32.38 | N:30.55 | N:26.84 | N:28.76 | N:27.93 | N:33.54 | N:7.33 | N:16.07 | N:18.76 | N:15.88 | N:10.98 | N:17.69 | N:18.08 | N:17.46 | N:54.53 | Y:31.2 | Y:35.55 | 2 | 0.09 | 0 | 0 | 0 |  | **4** | 6.5 | 5.75 | 6 |
| 1 | 153995978 | 153996637 | N:34.38 | N:32.08 | N:30.67 | N:30.3 | Y:31.3 | N:24.74 | N:26.35 | N:31.42 | N:30.71 | N:23.14 | N:27.1 | N:30.55 | N:11.62 | N:19.69 | N:23.6 | N:21.34 | N:14.04 | N:28.54 | N:21.53 | N:17.33 | N:57.4 | N:40.63 | N:34.63 | 1 | 0.04 | 0.17 | 0 | 0 |  | **5** | 5.67 | 4.88 | 6.5 |
| 1 | 157450062 | 157450954 | N:30.52 | N:30.33 | N:32.64 | N:31.86 | N:29.55 | N:30.39 | N:27.98 | N:34.07 | N:27.46 | N:26.21 | N:32.4 | N:30.29 | N:13.74 | Y:17.22 | Y:18.92 | N:17.9 | Y:9.06 | Y:18.51 | Y:13.85 | N:12.23 | N:59.65 | N:14.41 | N:28.67 | 5 | 0.22 | 0 | 0 | 0.63 |  | **6** | 2.42 | 2.38 | 2.5 |
| 1 | 157698899 | 157699774 | N:31.04 | N:29.81 | N:37.96 | N:35.33 | N:30.53 | N:33.85 | N:29.67 | N:29.12 | N:33.06 | N:27.92 | Y:30.1 | N:30.24 | N:10.56 | Y:17.03 | N:19.72 | N:18.18 | N:10.49 | N:25.16 | N:15 | N:19.17 | N:56.97 | N:23.7 | N:35.57 | 2 | 0.09 | 0 | 0.17 | 0.13 |  | **7** | 2.67 | 0.88 | 1.5 |
| 1 | 159294092 | 159294382 | N:30.54 | N:35.12 | N:36.16 | N:33.7 | N:29.42 | N:33.87 | N:31.51 | N:34.16 | N:41.67 | N:26.52 | N:29.2 | N:28.29 | N:8.79 | N:9.42 | N:14.71 | N:14.21 | N:11.14 | N:21.42 | N:10.56 | N:16.72 | Y:64.12 | N:17.91 | N:27.93 | 1 | 0.04 | 0 | 0 | 0 |  | **8** | 1.5 | 0.63 | 0.5 |
| 1 | 159625843 | 159626482 | N:31.55 | Y:29.05 | N:38.15 | N:31.95 | N:34.38 | N:31.45 | N:37.86 | N:36.26 | N:34.78 | N:15.96 | N:34.93 | N:32.18 | N:5.34 | N:6.89 | N:8.44 | N:8.56 | N:6.07 | N:10.57 | N:6.61 | N:6.03 | N:58.41 | N:4.95 | N:11.38 | 1 | 0.04 | 0.17 | 0 | 0 |  | **9** | 1.08 | 0.13 | 0 |
| 1 | 160455218 | 160455708 | N:29.23 | Y:27.08 | Y:27.13 | Y:25.7 | Y:31.53 | Y:25.45 | N:23.71 | Y:25.27 | Y:29.13 | Y:17.78 | Y:20.88 | Y:24.06 | N:5.51 | N:8.04 | Y:13.52 | Y:13.85 | N:4.97 | N:13.26 | N:6.16 | Y:8.05 | Y:51.59 | N:12.13 | N:18.26 | 14 | 0.61 | 0.83 | 0.83 | 0.38 |  | **10** | 0.33 | 0 | 1 |
| 1 | 160463921 | 160464245 | Y:28.33 | Y:26.36 | Y:36.38 | Y:33.48 | Y:30.56 | Y:35.83 | N:26.67 | Y:37 | Y:33.28 | Y:26.98 | Y:27.46 | Y:27.11 | N:7.35 | N:10.78 | Y:24.3 | Y:12.76 | N:8.64 | N:19.88 | N:9.19 | Y:14.78 | N:53.34 | N:18.05 | N:26.32 | 14 | 0.61 | 1 | 0.83 | 0.38 |  | **11** | 0.25 | 0.13 | 1 |
| 1 | 162222519 | 162223183 | N:33 | N:34.11 | N:36.3 | N:35.12 | N:31.99 | N:35.28 | N:34.18 | N:35.09 | N:32.51 | N:22 | N:28.32 | N:31.54 | N:7.19 | Y:14.55 | N:11.96 | N:12.76 | N:11.96 | N:20.41 | N:7.86 | N:12.2 | N:58.99 | N:18.16 | N:24.8 | 1 | 0.04 | 0 | 0 | 0.13 |  | **12** | 1.17 | 1.5 | 3 |
| 1 | 163501535 | 163501830 | Y:24.72 | Y:20.79 | N:25.16 | N:22.45 | Y:29.57 | N:28 | N:29.19 | Y:29.37 | Y:25.47 | Y:22.33 | Y:25.92 | N:26.2 | N:7.54 | N:14.52 | N:20.13 | N:12.16 | N:12.79 | N:31.31 | Y:18.6 | N:15.15 | N:47.71 | N:25.27 | N:28.64 | 8 | 0.35 | 0.5 | 0.67 | 0.13 |  | **13** | 0.67 | 1.25 | 0.5 |
| 1 | 163694748 | 163695372 | N:20.28 | N:19.15 | N:22.22 | N:26.68 | N:23.24 | N:23.45 | Y:17.25 | N:23.17 | Y:23.37 | Y:18.33 | Y:19.71 | Y:20.45 | Y:8.13 | N:11.72 | Y:10.83 | N:11 | N:11.07 | N:18.59 | N:12.49 | N:15.99 | N:51.97 | N:17.81 | N:32.76 | 7 | 0.3 | 0 | 0.83 | 0.25 |  | **14** | 0.25 | 0.13 | 0.5 |
| 1 | 163906737 | 163910850 | N:16.87 | Y:27.66 | N:17.73 | N:18.85 | N:18.47 | Y:27.03 | Y:23.49 | N:16.75 | N:19.39 | N:14.35 | N:19.7 | N:20.39 | N:7.96 | N:14.89 | N:17.41 | N:10.85 | N:7.12 | N:14.43 | Y:24.78 | N:15.69 | Y:64.69 | N:28.74 | N:31.19 | 5 | 0.22 | 0.33 | 0.17 | 0.13 |  | **15** | 0.25 | 0 | 0 |
| 1 | 164414998 | 164416037 | N:22.53 | N:21.8 | N:23.52 | N:21.4 | N:23.78 | Y:29.98 | N:20.03 | N:22.99 | N:24.74 | N:17.03 | N:23.2 | Y:28.66 | Y:12.13 | Y:18.09 | Y:24.21 | Y:21.51 | N:9.62 | N:19.37 | Y:17.75 | N:11.85 | Y:61.04 | N:14.65 | N:24.32 | 8 | 0.35 | 0.17 | 0.17 | 0.63 |  | **16** | 0 | 0 | 0 |
| 1 | 171152000 | 171152790 | N:33.77 | N:31.86 | N:34.43 | N:28.44 | N:33.14 | Y:33.91 | N:34.25 | N:32.64 | N:28.79 | N:26.27 | N:28.56 | N:33.94 | N:9.76 | N:14.41 | N:19.6 | N:20.95 | N:11.32 | N:26.58 | N:13.62 | N:20.12 | N:62.11 | N:26.96 | N:30.97 | 1 | 0.04 | 0.17 | 0 | 0 |  | **17** | 0.5 | 0.88 | 1 |
| 1 | 179728775 | 179729463 | N:28.08 | N:23.79 | Y:27.5 | N:30.09 | N:25.98 | N:28.05 | N:24.6 | N:28.95 | N:28.41 | N:21.1 | N:24.49 | N:26.37 | N:15.06 | N:22.84 | N:24.17 | N:19.58 | N:15.72 | N:25.97 | N:17.47 | N:22.55 | N:54.86 | N:42.92 | Y:45.94 | 2 | 0.09 | 0.17 | 0 | 0 |  | **18** | 0 | 0 | 0 |
| 1 | 180746896 | 180747970 | Y:27.38 | Y:38.09 | Y:33.62 | Y:34.5 | Y:24.74 | Y:33.59 | Y:24.21 | N:21.75 | Y:31.97 | Y:23.93 | Y:26.88 | Y:33.71 | N:5.45 | Y:16.07 | N:9.21 | Y:15.68 | N:8.55 | Y:24.55 | Y:15.77 | N:9.83 | Y:55.44 | Y:16.54 | Y:32.46 | 18 | 0.78 | 1 | 0.83 | 0.5 |  | **19** | 0.08 | 0 | 0 |
| 1 | 182832847 | 182834082 | Y:23.45 | Y:23.81 | Y:26.51 | Y:31.13 | Y:29.01 | Y:29.36 | Y:30.14 | Y:29.49 | Y:26.81 | Y:20.89 | Y:24.81 | Y:25.72 | Y:17.95 | Y:25.04 | Y:21.16 | Y:21.29 | N:10.85 | Y:31.06 | Y:16.83 | N:16.5 | Y:57.21 | Y:51.45 | Y:48.3 | 21 | 0.91 | 1 | 1 | 0.75 |  | **20** | 0.67 | 1 | 0.5 |
| 1 | 184347893 | 184348804 | N:28.21 | N:27.1 | N:30.62 | N:32.58 | Y:27.44 | Y:28.36 | Y:25.49 | N:28.76 | N:24.77 | N:28.51 | N:27.82 | N:26.4 | N:13.74 | Y:26.35 | N:27.19 | Y:23.36 | Y:10.99 | Y:26.22 | Y:21.97 | N:23.44 | N:59.54 | N:48.21 | N:43.38 | 8 | 0.35 | 0.33 | 0.17 | 0.63 |  | **21** | 0 | 0 | 0 |
| 1 | 185856831 | 185857511 | N:31.58 | N:32.03 | N:34.71 | Y:30.93 | Y:34.14 | N:33.62 | Y:31.19 | Y:32.88 | N:36.08 | N:27.85 | N:33.37 | N:31.06 | Y:10.89 | Y:16.89 | N:19.14 | N:18.85 | N:9.46 | N:18.73 | Y:15.19 | N:18.73 | N:63.71 | N:20.04 | N:22.76 | 7 | 0.3 | 0.33 | 0.33 | 0.38 |  | **22** | 0 | 0 | 0 |
| 1 | 186846427 | 186847073 | N:27.85 | Y:30.91 | Y:33.27 | N:33.26 | N:32.05 | Y:27.53 | N:31.68 | N:31.33 | N:34.67 | N:27.86 | N:31.07 | N:32.88 | N:11.97 | N:17.91 | N:21.44 | N:15.72 | N:8.91 | N:23.1 | N:14.04 | N:18.75 | N:71.54 | N:24.57 | N:32.43 | 3 | 0.13 | 0.5 | 0 | 0 |  | **23** | 0 | 0 | 0 |
| 1 | 187340668 | 187342356 | N:30.55 | N:29.9 | N:34.74 | N:33.7 | N:31.52 | N:35.57 | N:35.31 | N:31.36 | Y:21.53 | Y:18.23 | N:33.31 | N:30.79 | N:6.83 | N:14.11 | N:17.54 | Y:13.58 | N:7.45 | Y:10.1 | Y:4.27 | Y:6.83 | N:58.91 | N:18.2 | N:25.8 | 6 | 0.26 | 0 | 0.33 | 0.5 |  | **24** | 0.08 | 0 | 0 |
| 1 | 188148835 | 188149736 | Y:34.88 | Y:28.31 | N:35.15 | Y:28.18 | N:24.41 | N:27.14 | N:29.89 | N:35.52 | N:31.43 | N:24.15 | N:31.91 | N:29.36 | N:8.59 | N:17.43 | N:22.25 | N:16.67 | N:13.52 | Y:20.45 | N:17.22 | N:13.78 | N:62.05 | N:22.09 | N:28.7 | 4 | 0.17 | 0.5 | 0 | 0.13 |  | **25** | 0 | 0 | 0 |
| 10 | 9703183 | 9703845 | Y:31.12 | Y:31.69 | Y:33.96 | Y:31.75 | N:32.58 | N:32.91 | N:31.34 | N:30.72 | N:38.52 | N:28.11 | N:35.51 | N:28.79 | N:6.01 | N:18.22 | N:20.03 | N:19.79 | N:11.33 | N:23.15 | N:16.32 | N:14.43 | N:71.92 | Y:19.35 | Y:28.29 | 6 | 0.26 | 0.67 | 0 | 0 |  | **26** | 0 | 0 | 0 |
| 12 | 335263 | 335965 | N:18.92 | N:23.53 | N:23.51 | N:25.9 | N:23.44 | N:23.7 | Y:23.76 | N:25.47 | N:25.86 | N:21.54 | N:24.79 | N:24.52 | N:9.95 | N:14.1 | N:20.25 | N:19.04 | N:8.38 | N:19.66 | N:14.09 | N:12.24 | N:54.94 | Y:33.59 | N:39.07 | 2 | 0.09 | 0 | 0.17 | 0 |  | **27** | 0 | 0 | 0 |
| 12 | 3240697 | 3240898 | Y:203.09 | Y:163.91 | Y:251.69 | Y:151.86 | Y:210.81 | Y:150.94 | Y:183.72 | Y:191.2 | Y:141.61 | Y:80.62 | N:174.01 | Y:143.42 | Y:66.56 | Y:90.16 | Y:84.96 | Y:76.41 | N:13.22 | N:62.44 | Y:66.97 | Y:59.98 | N:259.74 | Y:182.78 | Y:559.21 | 19 | 0.83 | 1 | 0.83 | 0.75 |  | **28** | 0 | 0 | 0 |
| 12 | 3277416 | 3278316 | N:95.15 | N:52.48 | N:99.43 | N:124.42 | N:70.58 | N:53.99 | N:96.32 | N:56.02 | N:80.65 | N:66.55 | N:30.79 | N:74.87 | Y:50.9 | Y:99.52 | Y:112.23 | Y:102.07 | N:17.89 | N:37.9 | Y:80.22 | Y:91.06 | Y:157.41 | Y:67.2 | Y:283.77 | 9 | 0.39 | 0 | 0 | 0.75 |  | **Z** | 5.83 | 4 | 7.5 |
| 12 | 6823591 | 6824459 | N:26.54 | N:29.28 | N:29.74 | N:29.48 | N:33.75 | N:28.65 | N:28.94 | N:29.25 | N:32.15 | N:24.71 | N:30.93 | N:29.95 | N:12.53 | N:19.32 | N:19.12 | N:18.73 | N:13.91 | N:22.84 | N:17.26 | N:22.74 | N:65.2 | Y:33.33 | N:33.56 | 1 | 0.04 | 0 | 0 | 0 |  | **Unplaced ['A...']** | 0.17 | 1.75 | 1 |
| 12 | 15106212 | 15106979 | N:8.59 | N:4.1 | N:7.55 | N:5.08 | N:6.45 | N:6.86 | N:5.48 | N:6.67 | N:4.28 | N:3.31 | N:5.73 | N:4.26 | N:2.89 | N:5.14 | N:5.65 | N:6.99 | N:2.26 | N:5.26 | N:2.76 | N:7.28 | Y:28.35 | N:8.95 | N:22.88 | 1 | 0.04 | 0 | 0 | 0 |  | **Unplaced ['J...']** | 1.17 | 2.13 | 1.5 |
| 12 | 17400816 | 17401479 | N:37.53 | N:30.8 | N:39.07 | N:34.49 | N:31.07 | N:39.49 | N:34.35 | Y:31.34 | N:30.56 | N:31.42 | N:34.38 | Y:30.66 | N:7.1 | N:14.6 | N:21.72 | N:17.36 | N:15.5 | N:21.51 | N:10.42 | N:14.77 | N:68.97 | N:21.44 | N:31.65 | 2 | 0.09 | 0 | 0.33 | 0 |  | **Total** | 69.5 | 63.13 | 62.33 |
| 2 | 1183407 | 1184047 | Y:26.6 | N:33.85 | N:33.79 | N:29.38 | Y:29.23 | N:29.5 | N:26.65 | N:27.35 | N:27.84 | N:23.32 | N:31.38 | N:25.69 | N:8.57 | N:15.9 | N:16.82 | N:16.3 | N:10.12 | N:18.32 | N:15.3 | N:17.77 | N:60.96 | N:9.82 | N:43.01 | 2 | 0.09 | 0.33 | 0 | 0 |  | **(Total) St.Dev.** | 7.18 | 7.4 | 5.69 |
| 2 | 3491394 | 3492279 | N:38.01 | Y:32.09 | N:38.37 | Y:32.03 | N:29.41 | N:33.95 | N:33.97 | N:33.85 | N:38.01 | N:28.41 | N:35.09 | N:28.48 | Y:10.25 | Y:19.37 | N:17.93 | N:21.26 | N:11.72 | N:25.88 | Y:15.3 | Y:15.37 | N:66.2 | N:23.25 | N:24.63 | 6 | 0.26 | 0.33 | 0 | 0.5 |  |  |  |  |  |
| 2 | 5464380 | 5464705 | N:33.14 | N:37.4 | N:31.6 | N:32.13 | N:35.87 | N:31.54 | N:37.65 | N:31.82 | N:35.05 | N:22.32 | N:27.37 | N:34.04 | N:8.78 | N:8.68 | N:15.64 | N:12.98 | Y:8.82 | N:13.88 | N:11.72 | N:10.3 | N:70.01 | N:8.63 | N:17.71 | 1 | 0.04 | 0 | 0 | 0.13 |  |  |  |  |  |
| 2 | 7818049 | 7818465 | N:29.3 | N:26.83 | N:32.29 | N:34.01 | N:29.99 | Y:29.02 | N:27.06 | N:31.59 | N:29.89 | N:25.11 | N:26.12 | N:31.68 | N:15.14 | N:14.23 | N:17.25 | N:21.02 | N:10.65 | Y:21.78 | Y:14.65 | Y:16.51 | N:52.24 | N:37.93 | N:37.97 | 4 | 0.17 | 0.17 | 0 | 0.38 |  | S3d. Mean integrations per chromosome per chicken 'type' | | | |
| 2 | 10512425 | 10512887 | N:30.16 | Y:27.64 | Y:30.1 | N:35.35 | Y:28.01 | N:27.58 | N:28.49 | Y:26.7 | N:29.63 | N:27.28 | Y:33.9 | N:33.68 | Y:12.42 | Y:16.73 | N:25.4 | Y:22.6 | Y:12.16 | N:29.46 | N:18.09 | N:22.06 | N:48.31 | N:38.63 | Y:37.66 | 10 | 0.43 | 0.5 | 0.33 | 0.5 |  |  |  |  |  |
| 2 | 11018857 | 11019506 | N:34.1 | N:32.93 | Y:36.63 | N:35.61 | N:33.13 | N:32.42 | N:35.25 | N:39.07 | N:31.77 | N:30.48 | N:36.28 | N:34.87 | N:10.2 | N:16.02 | N:20.38 | N:16.95 | N:11.05 | N:22.39 | N:15.16 | N:14.89 | N:71.75 | N:18.94 | N:28.51 | 1 | 0.04 | 0.17 | 0 | 0 |  | **Chr** | **Ethiopian** | **Pirbright** | **SRA** |
| 2 | 16258680 | 16259495 | N:28.66 | N:36.09 | N:37.28 | N:36.46 | N:36.51 | Y:34.27 | N:35.12 | N:32.32 | N:30.54 | N:19.73 | N:30.48 | N:30.48 | N:10.23 | N:17.77 | N:19.01 | N:18.16 | N:11.3 | N:22.14 | N:13.77 | N:16.93 | N:58.08 | N:33.03 | N:29.82 | 1 | 0.04 | 0.17 | 0 | 0 |  | **1** | 21.08 | 19.75 | 17.5 |
| 2 | 20044191 | 20044405 | N:15.5 | N:13.89 | N:13.83 | N:15.19 | N:13.25 | N:16.38 | N:8.2 | N:14.2 | N:13.89 | N:9.43 | N:11.4 | N:15.33 | N:9.03 | N:16.81 | N:10.26 | N:7.22 | N:3.05 | N:7.82 | N:10.84 | N:8.44 | Y:35.05 | N:11.51 | N:23.92 | 1 | 0.04 | 0 | 0 | 0 |  | **2** | 11.17 | 11.75 | 7 |
| 2 | 20971534 | 20972437 | N:33.47 | Y:30.84 | N:33.37 | N:39.56 | N:33.29 | N:38.12 | N:32.21 | Y:37.69 | N:34.39 | N:23.75 | N:32.76 | N:32.96 | N:4.61 | Y:13 | N:13.22 | Y:16.76 | Y:10.28 | Y:20.13 | Y:15.36 | Y:12.88 | N:57.7 | N:17.26 | N:30.51 | 8 | 0.35 | 0.17 | 0.17 | 0.75 |  | **3** | 12 | 8.63 | 10 |
| 2 | 21240687 | 21244651 | N:33.14 | N:36.39 | N:31.41 | N:31.29 | N:35.05 | N:29.25 | N:28.49 | N:30.68 | N:30.62 | N:22.71 | N:32.77 | Y:41.74 | Y:12.38 | N:21.99 | N:29.67 | N:20.21 | N:11.35 | Y:30.58 | N:28.03 | Y:27.81 | Y:75.06 | Y:64.93 | N:42.91 | 6 | 0.26 | 0 | 0.17 | 0.38 |  | **4** | 7.42 | 5.88 | 6 |
| 2 | 23631823 | 23632481 | N:31.32 | N:34.1 | N:31.45 | Y:29.8 | N:34.33 | N:37.85 | N:29.21 | N:31.8 | N:38.08 | N:29.62 | N:33.91 | N:31.02 | N:9.36 | N:15.46 | N:15.78 | N:17.48 | N:13.62 | N:27.81 | N:15.16 | N:25.43 | N:66.97 | N:19.78 | N:21.49 | 1 | 0.04 | 0.17 | 0 | 0 |  | **5** | 5.67 | 4.88 | 6.5 |
| 2 | 34296518 | 34297385 | N:30.27 | N:31.41 | N:29.95 | N:34.16 | N:31.68 | N:31.17 | N:33.44 | N:30.3 | N:32.06 | N:26.95 | N:27.3 | N:32.37 | Y:10.76 | N:21.03 | N:25.38 | Y:23.83 | N:13.4 | N:26.69 | N:17.52 | N:20.96 | N:66.88 | N:34.24 | N:43.54 | 2 | 0.09 | 0 | 0 | 0.25 |  | **6** | 3.42 | 3.63 | 3.5 |
| 2 | 47663562 | 47663887 | N:31.79 | N:24.56 | N:26.43 | N:32.56 | N:35.25 | N:29.45 | N:32.34 | N:34.18 | N:32.49 | N:25.41 | N:28.96 | N:23.92 | N:16.67 | N:18.03 | N:24.69 | N:18.82 | Y:6.94 | N:27.06 | N:20.56 | N:27.26 | N:54.26 | N:44.25 | N:49.19 | 1 | 0.04 | 0 | 0 | 0.13 |  | **7** | 2.67 | 0.88 | 1.5 |
| 2 | 50798296 | 50798951 | Y:29.87 | N:33.07 | N:37.42 | Y:35.82 | N:40.12 | Y:37.35 | N:35.67 | N:30.5 | N:32.12 | N:27.45 | N:31.54 | Y:28.47 | N:13.48 | N:19.39 | N:19.16 | N:22.33 | N:17.09 | N:27.79 | N:16.25 | N:18.21 | N:66.4 | N:26.29 | N:35.8 | 4 | 0.17 | 0.5 | 0.17 | 0 |  | **8** | 1.5 | 0.63 | 0.5 |
| 2 | 51801055 | 51801954 | N:37.9 | N:33.94 | N:35.26 | N:37.05 | N:35.43 | N:34.78 | Y:33.08 | Y:33.49 | Y:34.47 | Y:24.6 | Y:33.88 | N:34.95 | Y:9.52 | Y:18.45 | Y:20.95 | N:18.45 | Y:12.94 | Y:23.3 | Y:16.66 | Y:18.18 | N:70.93 | N:16.51 | Y:32.98 | 13 | 0.57 | 0 | 0.83 | 0.88 |  | **9** | 1.08 | 0.13 | 0 |
| 2 | 53111756 | 53112017 | Y:27.96 | Y:27.24 | N:28.94 | Y:32.03 | Y:28.03 | Y:29.3 | Y:27.06 | Y:24.88 | Y:27.26 | Y:23.18 | Y:26.17 | N:23.59 | N:7.63 | Y:14.37 | Y:18.8 | N:22.92 | N:10.14 | Y:27.12 | Y:14.87 | N:18.86 | N:52.24 | Y:31.76 | N:36.23 | 15 | 0.65 | 0.83 | 0.83 | 0.5 |  | **10** | 0.33 | 0 | 1 |
| 2 | 58047649 | 58047977 | N:31.83 | N:28.98 | N:30.88 | N:31.42 | N:28.25 | N:29.54 | N:34.32 | N:31.68 | N:27.7 | N:24.16 | N:28.65 | N:29.97 | N:9.42 | N:18.2 | N:17.91 | N:16.51 | N:14.17 | N:24.84 | N:14.78 | N:18.74 | Y:67.69 | N:29.26 | N:33.22 | 1 | 0.04 | 0 | 0 | 0 |  | **11** | 0.25 | 0.13 | 1 |
| 2 | 65341352 | 65341989 | N:31.67 | N:34.43 | N:35.81 | N:32.74 | Y:32.96 | Y:35.19 | N:33.89 | N:34.28 | N:39.23 | N:31.47 | N:34.8 | Y:28.75 | N:13.3 | N:11.25 | N:18.16 | N:16.9 | N:9.39 | N:20.73 | N:10.77 | N:14.52 | N:66.32 | Y:15.37 | N:20.33 | 4 | 0.17 | 0.33 | 0.17 | 0 |  | **12** | 1.17 | 1.5 | 3 |
| 2 | 73742831 | 73743267 | N:31.1 | N:30.48 | N:30.01 | N:31.84 | N:31.28 | N:37.8 | N:35.16 | N:32.17 | N:36.73 | N:26 | N:34.3 | N:31.46 | N:10.04 | N:12.2 | N:14.26 | N:14.93 | N:9.66 | N:19.75 | Y:13.06 | N:10.55 | N:60.41 | N:15.58 | N:24.31 | 1 | 0.04 | 0 | 0 | 0.13 |  | **13** | 0.67 | 1.25 | 0.5 |
| 2 | 75447503 | 75448357 | N:30.15 | N:33.05 | N:33.6 | N:37.01 | Y:30.54 | N:32.79 | N:27.78 | N:30.31 | N:29.04 | N:25.14 | N:31.35 | N:25.92 | N:8.86 | N:18.35 | N:22.38 | N:18.16 | N:10.44 | N:18.51 | N:13.8 | N:16.16 | N:59.14 | N:32.26 | N:44.2 | 1 | 0.04 | 0.17 | 0 | 0 |  | **14** | 0.25 | 0.13 | 0.5 |
| 2 | 77178330 | 77179069 | Y:26.26 | N:29.13 | N:37.57 | N:30.83 | N:34.85 | N:33.6 | N:31.13 | N:33.85 | N:31.78 | N:25.11 | N:30.38 | N:32.7 | N:7.13 | N:14.47 | N:12.41 | N:14.38 | N:11.45 | N:21.52 | N:11.19 | N:12.42 | N:52.98 | N:14.59 | N:24.79 | 1 | 0.04 | 0.17 | 0 | 0 |  | **15** | 0.25 | 0 | 0 |
| 2 | 80333585 | 80334273 | N:34.07 | Y:35.1 | N:30.52 | Y:33.44 | Y:26.67 | Y:29.71 | N:31.43 | N:37.66 | N:26.61 | N:20.73 | N:28.45 | N:33.19 | N:7.72 | Y:10.84 | Y:14.56 | N:11.31 | N:8.3 | Y:14.5 | Y:13.44 | Y:10.13 | N:57.8 | N:12.07 | N:24.3 | 9 | 0.39 | 0.67 | 0 | 0.63 |  | **16** | 0 | 0 | 0 |
| 2 | 81458689 | 81462600 | N:24.11 | N:30.39 | N:25.47 | N:24.83 | N:25.98 | N:28.27 | N:18.02 | N:20.4 | N:17.93 | N:14.94 | N:19.12 | N:20.05 | N:8.65 | N:11.45 | N:15.88 | N:10.41 | N:5.65 | N:16.88 | N:8.52 | N:14.09 | Y:47.8 | N:27.19 | N:43.5 | 1 | 0.04 | 0 | 0 | 0 |  | **17** | 0.5 | 0.88 | 1 |
| 2 | 81965001 | 81966072 | Y:31.86 | Y:28.73 | N:18.81 | N:22.46 | Y:29.26 | Y:26.96 | N:18.38 | Y:27.15 | N:19.22 | N:11.65 | N:14.18 | Y:23.14 | Y:8.85 | N:13.92 | N:11.2 | Y:15.24 | N:7.27 | Y:19.9 | Y:14.12 | Y:14.47 | Y:66.6 | N:14.29 | Y:37.55 | 13 | 0.57 | 0.67 | 0.33 | 0.63 |  | **18** | 0 | 0 | 0 |
| 2 | 82574297 | 82575518 | Y:31.31 | Y:27.11 | Y:32.68 | Y:31.44 | Y:32.4 | Y:33.42 | Y:26.55 | Y:29.88 | Y:30.92 | Y:24.15 | Y:31.03 | N:25.06 | Y:11.07 | Y:23.2 | Y:18.54 | Y:16.36 | Y:14.13 | Y:26.69 | Y:14.08 | Y:20.68 | Y:66.11 | N:14.5 | Y:38.36 | 21 | 0.91 | 1 | 0.83 | 1 |  | **19** | 0.08 | 0 | 0 |
| 2 | 87444008 | 87444641 | N:30.91 | N:30.96 | N:31.27 | N:33.43 | N:34.29 | N:30.08 | Y:35.5 | N:27.58 | N:33.54 | N:32.56 | N:31.13 | N:26.71 | N:9.18 | N:12.95 | N:17.28 | N:11.49 | N:12.07 | N:24.59 | N:15.81 | N:8.9 | N:64.52 | N:20.6 | N:32.64 | 1 | 0.04 | 0 | 0.17 | 0 |  | **20** | 0.67 | 1 | 0.5 |
| 2 | 92058477 | 92059335 | N:30.97 | N:36.04 | N:37.93 | N:35.45 | N:37.93 | N:34.5 | N:36.11 | Y:33.95 | N:38.79 | N:30.71 | N:35.12 | N:38.58 | N:13.69 | N:17.21 | N:18.72 | N:20.18 | N:15.36 | N:30.56 | N:14.35 | N:18.57 | N:63.93 | N:18.54 | N:22.85 | 1 | 0.04 | 0 | 0.17 | 0 |  | **21** | 0 | 0 | 0 |
| 2 | 92070857 | 92071755 | N:34.36 | N:31.35 | Y:31.48 | N:32.87 | Y:29.98 | N:38.35 | N:27.37 | N:31.45 | N:31.62 | N:26.86 | Y:26.75 | N:27.8 | N:9.49 | N:14.84 | N:19.68 | N:15.35 | Y:12.84 | N:22.63 | N:15.17 | N:15.64 | N:57.51 | N:23.39 | N:22.04 | 4 | 0.17 | 0.33 | 0.17 | 0.13 |  | **22** | 0 | 0 | 0 |
| 2 | 92856996 | 92858219 | Y:28.34 | Y:29.91 | Y:29.58 | Y:27.19 | N:24.26 | Y:35.34 | Y:30.67 | Y:34.32 | Y:34.58 | Y:24.2 | Y:27.48 | Y:24.05 | Y:10.08 | Y:12.18 | Y:17.21 | Y:14.74 | Y:8.28 | Y:22.16 | Y:13.32 | Y:14.1 | Y:51.32 | Y:20.05 | Y:30.18 | 22 | 0.96 | 0.83 | 1 | 1 |  | **23** | 0 | 0 | 0 |
| 2 | 93341773 | 93342126 | N:34.83 | N:32.44 | N:31.51 | Y:31.2 | N:32.57 | N:33.85 | N:34.65 | N:32.66 | N:33.1 | N:28.35 | N:35.12 | N:31.41 | N:9.95 | N:11.28 | N:14.22 | N:14.34 | N:13.14 | N:26.53 | N:11.44 | N:15.63 | N:61.5 | N:19.04 | N:25.31 | 1 | 0.04 | 0.17 | 0 | 0 |  | **24** | 0.08 | 0 | 0 |
| 2 | 94577735 | 94578007 | N:28.37 | N:28.77 | N:28.71 | N:27.48 | N:29.26 | N:28.83 | N:32.95 | N:25.06 | Y:34.55 | Y:24.71 | N:29.37 | Y:31.22 | N:10.64 | N:18.55 | N:22.33 | N:12.69 | N:13.82 | N:23.52 | N:17.32 | N:17.89 | N:55.27 | N:30.6 | N:26.08 | 3 | 0.13 | 0 | 0.5 | 0 |  | **25** | 0 | 0 | 0 |
| 2 | 94594272 | 94594911 | Y:25.18 | N:34.85 | Y:29.45 | N:31.55 | N:28.57 | N:32.66 | N:31.13 | N:28.84 | N:35.16 | N:23.33 | N:34.27 | N:30.5 | N:13.83 | N:17.62 | N:22.81 | N:20.37 | N:12.47 | N:28.37 | N:18.45 | N:19.12 | N:60.96 | N:41.68 | N:49.55 | 2 | 0.09 | 0.33 | 0 | 0 |  | **26** | 0 | 0 | 0 |
| 2 | 95480957 | 95481836 | Y:35.81 | N:31.89 | Y:32.96 | Y:36.06 | N:33.08 | Y:36.03 | Y:36.7 | N:35.01 | Y:32.11 | Y:29.31 | N:34.58 | Y:27.43 | N:7.64 | Y:11.57 | N:15.36 | Y:15.92 | Y:8.05 | N:17.44 | N:9.16 | Y:11.31 | N:60.53 | N:11.6 | Y:20.79 | 13 | 0.57 | 0.67 | 0.67 | 0.5 |  | **27** | 0 | 0 | 0 |
| 2 | 104297204 | 104297861 | N:35.05 | N:32.33 | N:31.9 | N:34.31 | Y:32.52 | N:33.99 | N:29.16 | N:35.67 | N:35.01 | N:25.73 | N:26.93 | Y:30.81 | N:7.71 | N:12.83 | N:15.79 | N:17.53 | N:7.92 | Y:14.43 | N:11.5 | Y:15.95 | N:66.47 | N:14.38 | N:26 | 4 | 0.17 | 0.17 | 0.17 | 0.25 |  | **28** | 0 | 0 | 0 |
| 2 | 106764059 | 106764678 | N:32.22 | N:28.2 | N:35.81 | N:36.91 | N:33.07 | N:33.79 | N:34.83 | N:33.91 | N:29.1 | N:29.53 | N:33.25 | N:34.16 | N:9.48 | Y:18.25 | N:18.87 | N:15.92 | N:12.87 | N:28.54 | N:13.48 | N:19.42 | N:68.12 | N:31.31 | N:27.39 | 1 | 0.04 | 0 | 0 | 0.13 |  | **Z** | 7.67 | 4.88 | 8.5 |
| 2 | 109248726 | 109249064 | N:24.81 | N:22.34 | N:26.88 | N:26.7 | N:21.02 | N:25.68 | N:28.82 | N:31.81 | N:18.31 | N:16.94 | N:21.72 | N:25.17 | N:9.31 | N:13.71 | N:15.02 | N:9.77 | N:6.72 | N:15.21 | Y:14.44 | N:13.55 | N:49.19 | N:30.79 | N:43.99 | 1 | 0.04 | 0 | 0 | 0.13 |  | **Unplaced ['A...']** | 0.83 | 2.13 | 2 |
| 2 | 115446270 | 115446856 | N:18.2 | N:9.78 | N:6.02 | N:9.43 | N:7.91 | N:5.78 | N:7.86 | N:22.26 | N:21.17 | N:13.77 | N:7.92 | N:16.71 | N:2.73 | N:8.19 | N:11.33 | N:8.05 | N:6.93 | N:10.68 | N:22.22 | N:8.43 | Y:34.26 | N:13.83 | N:14.61 | 1 | 0.04 | 0 | 0 | 0 |  | **Unplaced ['J...']** | 1.08 | 2.38 | 2 |
| 2 | 123095199 | 123095828 | N:32.58 | N:31.81 | N:35.14 | N:31.97 | N:34.8 | N:34.91 | Y:26.9 | N:37.26 | N:39.31 | N:29.3 | N:34.33 | N:30.36 | N:9.25 | N:10.31 | N:12.66 | N:12.92 | N:8.39 | N:16.31 | N:13.07 | N:10.12 | N:58.85 | N:8.1 | N:19.48 | 1 | 0.04 | 0 | 0.17 | 0 |  | **Total** | 79.83 | 70.38 | 69.67 |
| 2 | 123945004 | 123945201 | N:41.78 | N:37.87 | N:40.25 | N:36.01 | N:37.67 | N:37.57 | Y:43.32 | N:30.05 | N:29.02 | N:24.64 | N:27.31 | N:34.75 | N:5.12 | N:13.44 | N:18.76 | N:10.55 | N:13.36 | N:17.54 | N:13.41 | N:16.98 | N:71.67 | N:24.81 | N:27.26 | 1 | 0.04 | 0 | 0.17 | 0 |  | **(Total) St.Dev.** | 7.9 | 10.1 | 5.51 |
| 2 | 124765081 | 124765718 | N:32.63 | N:31.5 | N:29.79 | N:29.19 | Y:29.55 | Y:35.05 | N:32.96 | N:31.42 | N:32.79 | N:20.6 | N:30.14 | N:30.14 | Y:6.41 | Y:15.99 | Y:14.57 | N:14.87 | Y:10.04 | N:20.53 | N:12.24 | Y:12.7 | N:66.45 | N:16.34 | N:29.81 | 7 | 0.3 | 0.33 | 0 | 0.63 |  |  |  |  |  |
| 2 | 129140825 | 129141707 | N:32.84 | Y:29.06 | Y:26.32 | Y:29.17 | Y:28.9 | Y:31.96 | N:30.03 | N:30.68 | N:32.32 | N:26.22 | N:29.64 | N:27.45 | N:11.46 | N:36.51 | N:24.51 | N:20.26 | N:22.72 | N:42.7 | N:21.72 | N:72.5 | N:69.24 | N:34.12 | N:47.87 | 5 | 0.22 | 0.83 | 0 | 0 |  |  |  |  |  |
| 2 | 131563757 | 131567794 | N:25.25 | Y:38.94 | Y:39.61 | Y:33.69 | N:31.99 | Y:32.15 | Y:28.66 | Y:33.6 | Y:32 | Y:25.4 | Y:38.7 | Y:33.57 | N:12.43 | Y:23.32 | Y:32.73 | Y:21.18 | Y:14.03 | N:23.05 | Y:28.31 | Y:26.76 | Y:72.62 | N:35.82 | N:41.78 | 17 | 0.74 | 0.67 | 1 | 0.75 |  |  |  |  |  |
| 2 | 133607552 | 133608408 | N:26.47 | N:25.63 | N:30.75 | N:31.35 | N:30.63 | N:28.79 | N:23.38 | N:26.58 | N:33.09 | N:23.17 | N:27.46 | Y:29.44 | N:7.53 | N:17.57 | N:20.03 | N:19.52 | N:13.82 | N:21.62 | N:16.03 | N:13.16 | N:59.25 | N:24.85 | N:33.56 | 1 | 0.04 | 0 | 0.17 | 0 |  |  |  |  |  |
| 2 | 142113866 | 142114543 | Y:26.53 | N:33.15 | Y:29.29 | Y:32.07 | N:31.43 | N:37.63 | Y:29.05 | N:27.81 | N:26.88 | N:29.09 | N:28.89 | N:27.5 | Y:12.29 | N:16.28 | N:20.5 | N:16.26 | N:15.15 | Y:28.15 | N:15.35 | N:21.51 | N:60.44 | Y:32.73 | Y:43.02 | 8 | 0.35 | 0.5 | 0.17 | 0.25 |  |  |  |  |  |
| 2 | 142512897 | 142513907 | N:15.25 | N:15.98 | N:17.99 | Y:26.7 | N:17.76 | N:18.99 | N:17.11 | N:20.52 | N:16.59 | N:15.92 | N:19.18 | N:19.7 | N:9.19 | N:10.98 | N:15.97 | N:11.51 | N:5.96 | N:14 | N:14.78 | N:14.72 | Y:60.85 | N:23.88 | Y:52.13 | 3 | 0.13 | 0.17 | 0 | 0 |  |  |  |  |  |
| 2 | 143463257 | 143468861 | N:12.74 | N:12.76 | N:11.97 | N:11.71 | N:13.08 | N:15.4 | N:13.55 | N:12.65 | N:12.12 | N:11.27 | N:12.28 | N:12.1 | N:4.6 | Y:17.5 | Y:20.74 | N:12.13 | N:4.77 | N:12.75 | N:11.7 | N:11.96 | Y:54.93 | N:16.66 | N:17.05 | 3 | 0.13 | 0 | 0 | 0.25 |  |  |  |  |  |
| 2 | 147548249 | 147548775 | N:17.83 | N:17.87 | N:21.41 | N:19.41 | Y:21.72 | N:16.36 | N:19.06 | N:20.8 | N:20.46 | N:17.92 | N:18.61 | N:16 | N:15.66 | N:24.01 | N:13.04 | N:19.59 | N:9.89 | N:15.29 | N:22.02 | N:10.5 | N:48.1 | N:18.33 | N:25.97 | 1 | 0.04 | 0.17 | 0 | 0 |  |  |  |  |  |
| 20 | 2428746 | 2429684 | N:31.01 | N:37.15 | N:32.71 | N:27.78 | N:32.61 | N:34.76 | N:31.46 | N:30.67 | N:27.36 | N:26.39 | Y:31.62 | Y:29.61 | N:11.77 | Y:16.76 | N:22.38 | Y:19.07 | N:11.7 | N:27.04 | N:15.88 | N:19.82 | N:70.11 | N:22.84 | N:24.42 | 4 | 0.17 | 0 | 0.33 | 0.25 |  |  |  |  |  |
| 20 | 7661897 | 7662786 | Y:30.32 | Y:30.13 | Y:33.44 | N:36.48 | N:36.36 | N:34.52 | Y:32.38 | N:31.7 | N:32.93 | N:24.95 | N:32.37 | Y:32.79 | Y:8.58 | Y:15.07 | N:12.07 | N:16.91 | Y:10.86 | Y:21 | Y:13.52 | Y:15.4 | N:65.9 | N:15.24 | Y:24.76 | 12 | 0.52 | 0.5 | 0.33 | 0.75 |  |  |  |  |  |
| 20 | 9408955 | 9409567 | Y:24.8 | N:29.14 | N:31.92 | N:31.89 | N:31.58 | N:30.96 | N:32.29 | N:31.51 | N:29.53 | N:28.03 | N:34.54 | N:30.2 | N:14.5 | N:16.34 | N:25.57 | N:18.79 | N:10.36 | N:25.35 | N:13.33 | N:16.89 | N:58.69 | N:35.35 | N:26.56 | 1 | 0.04 | 0.17 | 0 | 0 |  |  |  |  |  |
| 3 | 12508635 | 12509292 | N:28.15 | N:30.33 | N:33.17 | N:32.83 | N:34.56 | N:29.7 | N:33.45 | N:32.04 | N:30.69 | N:27.88 | N:32.43 | N:36.9 | N:7.47 | N:14.3 | N:12.58 | N:13.49 | N:10.12 | N:17.03 | N:10.7 | N:15.61 | N:66.01 | Y:29.01 | N:30 | 1 | 0.04 | 0 | 0 | 0 |  |  |  |  |  |
| 3 | 19821802 | 19823007 | Y:24.05 | Y:26.6 | Y:27.49 | Y:24.79 | Y:31.01 | Y:24.72 | Y:22.93 | Y:27.11 | Y:27.85 | Y:20.24 | Y:23.11 | Y:23.13 | Y:7.54 | Y:7.56 | Y:12.96 | Y:11.36 | Y:9.69 | Y:15.39 | Y:9.15 | Y:11.46 | Y:54.46 | Y:9.1 | Y:26.62 | 23 | 1 | 1 | 1 | 1 |  |  |  |  |  |
| 3 | 22595889 | 22596501 | N:33.72 | N:29.91 | N:34.81 | N:38.77 | N:30.19 | N:37.51 | N:37.66 | N:33.23 | N:35.55 | N:34.02 | N:29.96 | Y:32.23 | N:10.59 | N:18.11 | N:21.35 | N:20.72 | N:10 | N:27.63 | N:13.68 | N:17.88 | N:69.92 | N:27.02 | N:33.53 | 1 | 0.04 | 0 | 0.17 | 0 |  |  |  |  |  |
| 3 | 34782953 | 34783379 | Y:36.16 | N:29.25 | N:33.34 | Y:36.23 | Y:41.04 | N:29.79 | N:33.44 | N:32.31 | Y:41.52 | Y:35.66 | N:35.9 | Y:34.11 | N:12.46 | N:17.27 | Y:31.89 | Y:30.24 | Y:19.32 | Y:37.41 | N:13.51 | Y:28.82 | N:58.68 | N:33.64 | Y:41.84 | 12 | 0.52 | 0.5 | 0.5 | 0.63 |  |  |  |  |  |
| 3 | 36156197 | 36156846 | N:33.57 | N:28.09 | N:27.88 | N:25.92 | N:29.09 | N:31.32 | N:32 | N:29.94 | N:30.14 | N:28.56 | N:32.79 | N:29.03 | Y:13.45 | N:23.48 | N:22.63 | N:16.98 | Y:14.95 | Y:24.11 | Y:20.51 | N:22.79 | N:63.57 | N:31.13 | N:32.92 | 4 | 0.17 | 0 | 0 | 0.5 |  |  |  |  |  |
| 3 | 40908270 | 40908901 | N:32.37 | N:33.13 | N:32.22 | N:31.53 | N:35.8 | N:30.97 | Y:30.96 | Y:29.59 | N:29.53 | N:25.87 | Y:30.54 | Y:28.96 | N:10.37 | N:17.61 | N:15.78 | N:19.12 | N:13.4 | N:30 | N:13.72 | N:14.46 | N:62.44 | N:17.61 | Y:25.92 | 5 | 0.22 | 0 | 0.67 | 0 |  |  |  |  |  |
| 3 | 44140622 | 44141485 | N:29.54 | Y:29.16 | Y:31.06 | N:31.35 | Y:29.84 | Y:29.8 | Y:30.79 | N:31.84 | N:33.45 | N:22.93 | N:35.82 | N:31.69 | N:8.18 | N:16.91 | N:15.63 | N:16.5 | N:12.17 | N:21.28 | N:13.85 | N:15.21 | N:56.66 | N:14.02 | N:35.52 | 5 | 0.22 | 0.67 | 0.17 | 0 |  |  |  |  |  |
| 3 | 46003530 | 46004338 | N:31.03 | N:30.76 | N:33.63 | N:35.38 | N:33.43 | N:33.73 | N:32.17 | N:36.11 | N:26.79 | N:29.24 | N:30.95 | N:29.57 | N:12.44 | N:18.91 | N:15.84 | N:19.67 | N:12.38 | N:21.92 | N:19.97 | Y:19.18 | N:64.99 | N:19.56 | N:40.02 | 1 | 0.04 | 0 | 0 | 0.13 |  |  |  |  |  |
| 3 | 49572068 | 49572931 | Y:36.88 | Y:34.7 | Y:36.43 | N:32.39 | Y:33 | Y:35.8 | N:40.34 | Y:35.36 | N:35.41 | N:29.9 | N:31.02 | Y:31.57 | N:10.17 | Y:17.51 | N:20.53 | N:19.3 | N:16.46 | N:27.09 | N:14.58 | Y:20.78 | N:60.58 | N:23.08 | Y:31.47 | 10 | 0.43 | 0.83 | 0.33 | 0.25 |  |  |  |  |  |
| 3 | 50010952 | 50011588 | Y:28.08 | N:32.95 | N:29.09 | N:32.62 | N:31.96 | N:32.5 | N:29.15 | N:32.78 | N:29.43 | N:19.78 | N:31.77 | N:27.64 | N:8.84 | N:7.07 | N:14.44 | N:10.99 | N:7.49 | N:11.12 | N:6.44 | N:8.43 | N:59.39 | N:8.66 | N:23.48 | 1 | 0.04 | 0.17 | 0 | 0 |  |  |  |  |  |
| 3 | 54250109 | 54254158 | Y:40.33 | Y:42.3 | N:27.88 | N:29.68 | N:27.73 | Y:35.09 | N:21.31 | N:23.79 | N:22.88 | N:17.76 | N:25.69 | Y:34.96 | N:10.61 | Y:21.43 | Y:26.59 | N:13.17 | N:9.18 | N:18.64 | N:17.4 | N:18.69 | Y:73.62 | Y:46.89 | N:33.33 | 8 | 0.35 | 0.5 | 0.17 | 0.25 |  |  |  |  |  |
| 3 | 55831442 | 55836192 | N:31.04 | N:34.81 | N:33.1 | N:30.19 | N:38.6 | N:34.96 | N:22.41 | N:26.36 | N:18.6 | N:15.91 | N:22.93 | N:22.19 | N:11.92 | N:17.98 | N:18.32 | N:17.04 | N:8.48 | N:21.68 | N:11.49 | N:17.33 | Y:45.79 | N:36.14 | N:66.9 | 1 | 0.04 | 0 | 0 | 0 |  |  |  |  |  |
| 3 | 59937540 | 59938220 | N:30.24 | Y:27.7 | Y:32.88 | Y:30.23 | Y:32.34 | Y:31.38 | Y:28.59 | Y:30.48 | Y:25.6 | Y:24.32 | N:32.14 | Y:28.28 | N:7.14 | N:16.95 | N:14.9 | N:19.76 | N:10.12 | N:19.61 | N:14.13 | Y:14.11 | N:59.46 | N:17.03 | N:36.32 | 11 | 0.48 | 0.83 | 0.83 | 0.13 |  |  |  |  |  |
| 3 | 60621854 | 60622499 | Y:28.46 | N:31.51 | N:35.83 | N:35.89 | N:34.38 | N:37.51 | N:38.11 | Y:30.33 | N:38.03 | N:30.64 | N:36.61 | N:34.64 | N:7.34 | N:10 | N:14.4 | N:14.81 | N:10.81 | N:18.5 | N:11.77 | N:14.51 | N:69.1 | N:11.96 | N:11.48 | 2 | 0.09 | 0.17 | 0.17 | 0 |  |  |  |  |  |
| 3 | 63745322 | 63745967 | N:34.22 | N:28.61 | N:35.74 | Y:34.05 | N:35.8 | N:32.3 | N:35.69 | N:31.72 | N:33.05 | N:27.09 | N:35.27 | N:34.45 | N:9.54 | Y:20.16 | N:18.69 | N:15.72 | N:13.4 | N:25.55 | N:13.91 | N:18.72 | N:66.08 | Y:32.04 | Y:39.01 | 4 | 0.17 | 0.17 | 0 | 0.13 |  |  |  |  |  |
| 3 | 68891616 | 68892157 | N:33.79 | N:35.74 | N:28.72 | N:35.72 | N:31.19 | N:29.01 | N:34.73 | N:38.06 | N:31.26 | N:27.59 | N:29.31 | N:29.94 | N:10.99 | N:12.85 | Y:12 | N:14.19 | N:7.72 | N:20.27 | N:11.29 | N:12.73 | N:58.13 | Y:17.89 | N:24.3 | 2 | 0.09 | 0 | 0 | 0.13 |  |  |  |  |  |
| 3 | 70604555 | 70605387 | Y:22.24 | Y:22.51 | Y:24.06 | Y:22.01 | N:24.57 | Y:22.73 | N:15.59 | Y:20.99 | Y:25.26 | Y:19.59 | N:21.58 | Y:22.75 | N:5.95 | N:10.83 | Y:12.88 | N:13.7 | N:10.94 | N:12.24 | N:13.09 | Y:14.09 | N:50.16 | N:15.14 | N:20.68 | 11 | 0.48 | 0.83 | 0.67 | 0.25 |  |  |  |  |  |
| 3 | 72558723 | 72559573 | N:27.41 | N:32.97 | N:33.41 | N:29.59 | Y:30 | N:31.18 | N:33.04 | N:37.44 | N:30.67 | N:26.75 | N:29.37 | N:36.62 | N:7.75 | N:19.26 | N:22.52 | N:23.72 | N:17.31 | N:28.62 | N:16.66 | N:19.7 | N:63.96 | N:29.55 | N:47.33 | 1 | 0.04 | 0.17 | 0 | 0 |  |  |  |  |  |
| 3 | 73561879 | 73563101 | Y:29.52 | Y:29.85 | Y:33.08 | Y:35.76 | Y:31.02 | Y:32.76 | Y:27.13 | Y:33.32 | Y:32 | Y:23.15 | Y:26.12 | Y:32.4 | Y:8.82 | Y:13.85 | Y:18.48 | Y:14.98 | Y:13.11 | Y:24.2 | Y:15.15 | Y:14.8 | Y:58.13 | N:10.31 | Y:31.27 | 22 | 0.96 | 1 | 1 | 1 |  |  |  |  |  |
| 3 | 77502831 | 77503430 | N:33.94 | N:29.95 | N:29.73 | N:32.82 | N:35.78 | N:32.59 | N:33.08 | N:32.31 | N:33.05 | N:28.53 | N:33.3 | N:34.32 | N:11.15 | N:16.34 | N:19.89 | N:18.61 | N:13.37 | N:24.15 | N:13.12 | N:18.85 | N:62.88 | Y:26.5 | N:30.35 | 1 | 0.04 | 0 | 0 | 0 |  |  |  |  |  |
| 3 | 79282754 | 79283588 | N:34.18 | N:34.28 | Y:32.18 | N:31.51 | N:28.97 | N:34.29 | Y:29.62 | N:30.36 | N:32.68 | N:28.44 | N:30.99 | N:29.96 | N:10.8 | N:20.89 | N:19.75 | N:22.01 | N:13.21 | N:27.23 | N:19.6 | N:20.63 | N:65.54 | N:35.3 | N:43.48 | 2 | 0.09 | 0.17 | 0.17 | 0 |  |  |  |  |  |
| 3 | 83407619 | 83408514 | N:30.8 | N:31.55 | N:34.86 | Y:24.18 | Y:25.36 | Y:27.84 | Y:27.56 | N:31.34 | N:31.21 | N:22.39 | N:27.78 | N:31.53 | Y:5.79 | Y:12.31 | Y:11.62 | Y:11.03 | Y:9.91 | Y:14.86 | Y:9.56 | Y:12.12 | N:60.54 | Y:16.52 | Y:20.68 | 14 | 0.61 | 0.5 | 0.17 | 1 |  |  |  |  |  |
| 3 | 83776894 | 83777945 | Y:35.14 | Y:34.2 | Y:33.81 | Y:34.48 | Y:31.08 | Y:32.31 | Y:32.03 | Y:35.08 | Y:31.61 | Y:28.36 | Y:36.48 | Y:31.7 | Y:11.08 | N:17.36 | N:21.62 | N:22.36 | N:14.99 | Y:25.33 | Y:19.07 | Y:19.6 | Y:78.76 | Y:28.72 | Y:35.21 | 19 | 0.83 | 1 | 1 | 0.5 |  |  |  |  |  |
| 3 | 84550518 | 84551452 | N:33.7 | N:30.79 | N:33.12 | N:35.95 | N:36.86 | N:32.55 | Y:33.42 | Y:32.32 | Y:27.52 | Y:29.89 | Y:28.01 | Y:32.44 | N:10.11 | N:12.31 | N:22.7 | N:17.07 | N:14.75 | N:24.44 | N:18.71 | N:17.89 | N:58.07 | N:19.96 | N:26.68 | 6 | 0.26 | 0 | 1 | 0 |  |  |  |  |  |
| 3 | 84889113 | 84893120 | N:21.74 | N:25.35 | N:25.32 | N:23.7 | Y:33.07 | Y:31.12 | N:20.2 | N:23.4 | N:22.72 | N:18.76 | N:25.48 | N:24.41 | N:12.96 | N:15.4 | N:22.65 | N:11.64 | N:11.27 | N:18.93 | N:17.88 | N:15.42 | Y:68.57 | N:31.2 | N:36 | 3 | 0.13 | 0.33 | 0 | 0 |  |  |  |  |  |
| 3 | 86212524 | 86213394 | N:32.42 | N:31.22 | N:32.34 | N:30.89 | N:30.93 | N:30.96 | N:31.38 | N:33.15 | N:35.29 | N:22.28 | N:32.14 | N:29.78 | N:12.48 | N:18.44 | N:19.09 | Y:13.7 | N:9.56 | N:22.13 | N:16.17 | N:21.38 | N:59.86 | N:31.31 | N:31.29 | 1 | 0.04 | 0 | 0 | 0.13 |  |  |  |  |  |
| 3 | 86869588 | 86870258 | Y:31.34 | N:31.52 | N:28.81 | Y:30.93 | N:32.69 | N:30.42 | N:30.84 | N:28.02 | N:31.23 | N:24.88 | N:29.29 | N:26.93 | N:9.59 | N:11.69 | N:18.05 | N:17.49 | N:11.22 | Y:25.07 | N:18.75 | N:17.16 | N:62.07 | N:20.19 | N:21.61 | 3 | 0.13 | 0.33 | 0 | 0.13 |  |  |  |  |  |
| 3 | 88244141 | 88245055 | N:29.98 | Y:30.2 | N:31.08 | N:35.17 | N:33.01 | N:33.55 | N:31.6 | N:36.11 | N:34.47 | N:26.17 | N:30.51 | N:31.62 | N:11.75 | N:15.71 | N:20.75 | N:17.86 | N:14.65 | N:30.51 | N:16.71 | N:16.96 | N:58.45 | N:28.9 | N:32.02 | 1 | 0.04 | 0.17 | 0 | 0 |  |  |  |  |  |
| 3 | 91661280 | 91662061 | N:24.19 | N:30.98 | N:25.82 | N:35.51 | N:30.51 | N:29.31 | N:27.55 | N:32.19 | N:31.25 | N:28.28 | N:26.32 | N:25.19 | N:11.17 | Y:19.71 | N:18.64 | N:23.29 | N:12.21 | N:24.92 | N:15.38 | N:21.69 | N:56.52 | N:27.3 | N:23.02 | 1 | 0.04 | 0 | 0 | 0.13 |  |  |  |  |  |
| 3 | 93697657 | 93698572 | N:34.98 | N:33.32 | Y:31.14 | N:28.02 | N:33.23 | Y:31.57 | Y:27.19 | Y:29.45 | Y:28.86 | Y:19.2 | Y:31.94 | Y:26.06 | N:6.78 | N:14.47 | N:10.51 | Y:15.45 | N:6.77 | N:20.54 | N:15.33 | Y:10.71 | N:64.41 | Y:14.77 | N:19.14 | 11 | 0.48 | 0.33 | 1 | 0.25 |  |  |  |  |  |
| 3 | 95722787 | 95723662 | N:30.01 | N:31.02 | N:31.58 | N:35.89 | N:31.81 | N:32.68 | N:28.65 | N:30.87 | N:27.88 | N:25.8 | Y:33.05 | N:32.23 | N:9.77 | N:16.98 | N:14.77 | N:15.89 | N:8.76 | N:22.49 | N:15.34 | N:17.25 | N:56.57 | N:24.08 | N:31.55 | 1 | 0.04 | 0 | 0.17 | 0 |  |  |  |  |  |
| 3 | 96957515 | 96961612 | Y:35.27 | N:26.91 | Y:28.66 | N:18.96 | Y:31.02 | Y:31.69 | Y:22.62 | N:23.67 | N:18.37 | N:16.15 | N:23.8 | N:22.48 | N:12.4 | Y:23.41 | Y:28.03 | Y:18.83 | Y:11.58 | Y:19.97 | Y:21.16 | Y:26.15 | Y:68.71 | Y:39.7 | N:35.19 | 14 | 0.61 | 0.67 | 0.17 | 0.88 |  |  |  |  |  |
| 3 | 99426024 | 99426621 | N:37.42 | N:34.68 | N:36.52 | N:41.81 | N:33.9 | N:33.45 | N:35.37 | N:34.69 | N:33.59 | N:27.27 | N:27.93 | N:33.08 | Y:6.76 | N:13.67 | N:17.37 | Y:20.65 | N:10.41 | Y:24.33 | N:10.62 | Y:15.6 | N:60.58 | N:23.11 | N:22.92 | 4 | 0.17 | 0 | 0 | 0.5 |  |  |  |  |  |
| 3 | 102777226 | 102778143 | Y:30.65 | N:31.56 | Y:31.84 | Y:29.87 | Y:28.95 | Y:30.31 | N:32.51 | N:35.01 | N:32.83 | N:25.34 | Y:28.08 | N:28.95 | N:7.86 | N:13.05 | N:15.51 | N:13.02 | N:10.56 | N:16.75 | N:11.54 | N:11.9 | N:58.94 | N:10.11 | N:25.2 | 6 | 0.26 | 0.83 | 0.17 | 0 |  |  |  |  |  |
| 4 | 11123901 | 11124467 | Y:31.7 | Y:25.59 | Y:29.71 | N:18.19 | Y:24.05 | Y:28.05 | Y:23.99 | Y:26.73 | Y:27.3 | Y:22.84 | N:20.5 | Y:25.73 | Y:11.38 | Y:27.19 | N:21.32 | Y:19.8 | Y:14.86 | Y:31.57 | N:19.21 | Y:22.21 | Y:69.48 | Y:54.12 | N:36.16 | 18 | 0.78 | 0.83 | 0.83 | 0.75 |  |  |  |  |  |
| 4 | 15864150 | 15864742 | N:34.24 | N:37.05 | N:37.39 | N:37.5 | N:36.48 | N:38.28 | N:30.85 | N:35.72 | N:34.2 | N:27.76 | N:37.61 | N:31.88 | N:9.54 | N:10.92 | N:17.87 | N:14.02 | N:12.56 | Y:21.06 | N:12.91 | N:13.12 | N:60.03 | N:13.97 | Y:18.76 | 2 | 0.09 | 0 | 0 | 0.13 |  |  |  |  |  |
| 4 | 16267586 | 16268257 | N:31.26 | N:25.32 | N:27.11 | N:31.71 | N:32.85 | N:29.03 | N:26.38 | Y:28.29 | N:28.55 | N:29.26 | Y:27.6 | N:28.77 | N:8.9 | N:18.22 | N:20.03 | N:17.8 | N:8.88 | N:23.7 | N:11.81 | N:21.11 | N:57.43 | N:23.76 | N:28.17 | 2 | 0.09 | 0 | 0.33 | 0 |  |  |  |  |  |
| 4 | 19211542 | 19212344 | N:28.81 | N:29.47 | N:31.26 | N:27.9 | N:28.76 | N:30.77 | N:28.3 | N:34.87 | N:32.72 | N:26.22 | N:33.71 | N:31.32 | N:25.79 | N:30.39 | N:26.47 | N:40.1 | N:19.62 | N:29.36 | N:34.13 | N:18.98 | N:67.05 | Y:42.18 | N:51.47 | 1 | 0.04 | 0 | 0 | 0 |  |  |  |  |  |
| 4 | 20401588 | 20402427 | N:32.5 | N:34.28 | N:28.9 | N:33.1 | N:28.48 | Y:33.43 | N:26.07 | N:31.22 | N:28.76 | N:21.17 | N:29.87 | N:31.57 | N:6.66 | N:9.98 | Y:16.5 | N:13.45 | N:9.78 | N:16.06 | N:10.97 | N:10.09 | N:49.61 | N:13.09 | N:27.94 | 2 | 0.09 | 0.17 | 0 | 0.13 |  |  |  |  |  |
| 4 | 22222499 | 22223210 | N:27.07 | Y:23.95 | N:32.99 | Y:28.32 | Y:30.01 | Y:29.58 | N:27.18 | N:27.21 | N:23.39 | N:19.47 | N:28.32 | N:26.74 | N:10.4 | N:21.87 | N:21.2 | N:17.44 | N:9.34 | N:32.24 | N:18.24 | N:19.34 | N:54.36 | Y:40.85 | N:43.94 | 5 | 0.22 | 0.67 | 0 | 0 |  |  |  |  |  |
| 4 | 25887286 | 25887901 | N:29.8 | N:26.92 | N:31.18 | N:36.64 | N:30.73 | N:30.73 | N:30.85 | N:32.26 | N:30.67 | N:24.6 | N:31.07 | Y:31.29 | N:10.38 | N:22.41 | N:24.36 | N:24.73 | N:15.61 | N:28.37 | N:18.22 | N:24.92 | N:65.11 | N:38.52 | N:38.3 | 1 | 0.04 | 0 | 0.17 | 0 |  |  |  |  |  |
| 4 | 26530893 | 26531990 | Y:29.93 | Y:29.53 | Y:28.36 | Y:30.29 | Y:24.95 | Y:30.09 | Y:30.38 | Y:28.05 | Y:29.14 | Y:23.32 | Y:26.71 | Y:29.71 | N:9.43 | N:10.81 | N:13.11 | Y:20.48 | Y:14.03 | N:18.84 | Y:21.12 | Y:19.86 | Y:68.39 | Y:22.54 | Y:31.14 | 19 | 0.83 | 1 | 1 | 0.5 |  |  |  |  |  |
| 4 | 27180311 | 27180810 | N:29.52 | N:32.9 | N:35.02 | N:33.5 | N:37.95 | N:37.11 | N:35.19 | N:34.98 | N:33.37 | N:27.66 | N:33.97 | N:30 | N:8.37 | N:10.69 | N:12.9 | N:14.52 | N:11.64 | Y:16.64 | N:11.62 | Y:18.21 | N:61.01 | N:17.22 | N:27.6 | 2 | 0.09 | 0 | 0 | 0.25 |  |  |  |  |  |
| 4 | 27394572 | 27395212 | N:31.05 | N:31.3 | N:31.98 | Y:33.11 | N:34.56 | Y:33.72 | N:35.14 | N:37.92 | N:32.99 | N:25.88 | N:33.73 | Y:36.02 | N:10 | N:21.86 | N:26.73 | N:23.92 | N:17.06 | N:26.21 | N:16.76 | N:21.49 | N:59.55 | N:39.8 | N:26.62 | 3 | 0.13 | 0.33 | 0.17 | 0 |  |  |  |  |  |
| 4 | 29234085 | 29234741 | N:34.56 | Y:28.49 | N:34.71 | N:28.54 | N:29.65 | Y:26.25 | N:27.26 | N:31.28 | N:36.22 | N:27.15 | Y:26.24 | N:29.98 | Y:11.39 | N:22.41 | N:21.78 | Y:20.86 | Y:14.2 | Y:24.37 | N:19.28 | Y:15.65 | N:54.14 | N:41.06 | N:38.61 | 8 | 0.35 | 0.33 | 0.17 | 0.63 |  |  |  |  |  |
| 4 | 30595679 | 30596557 | N:26.64 | N:28.14 | N:28.44 | N:31.13 | N:30.25 | N:32.4 | Y:31.75 | N:27.44 | N:28.1 | N:24.27 | N:26.75 | N:33.71 | Y:11.57 | Y:16.94 | Y:20.16 | N:19.8 | Y:15.54 | N:21.26 | N:13.73 | Y:16.28 | N:53.97 | N:28.55 | Y:32.27 | 7 | 0.3 | 0 | 0.17 | 0.63 |  |  |  |  |  |
| 4 | 30632716 | 30633375 | Y:26.87 | Y:28.76 | N:37.94 | Y:32.97 | Y:33.06 | Y:30.91 | Y:26.31 | Y:30.72 | Y:33.34 | Y:24.43 | Y:29.36 | Y:27.69 | N:6.05 | N:14.57 | N:17.32 | N:15.1 | N:10.42 | N:20.93 | N:11.71 | N:17.35 | N:57.01 | N:20.99 | N:27.95 | 11 | 0.48 | 0.83 | 1 | 0 |  |  |  |  |  |
| 4 | 35886007 | 35886673 | N:32.39 | N:31.03 | N:35.06 | N:31.19 | N:37.74 | N:35.65 | Y:35.14 | N:41.02 | N:38.34 | N:29.21 | Y:31.34 | N:34.48 | N:4.96 | N:11.39 | N:14.49 | N:12.13 | N:10.17 | N:19.64 | N:13.55 | N:11.16 | N:64.33 | N:8.46 | N:23.54 | 2 | 0.09 | 0 | 0.33 | 0 |  |  |  |  |  |
| 4 | 40212346 | 40213006 | N:35.56 | N:33.79 | N:39.87 | N:38.72 | N:35.76 | N:38.11 | N:34.05 | N:38.29 | N:39.04 | N:28.49 | N:31.85 | N:33.84 | N:7.28 | Y:11.45 | Y:16.23 | N:8.87 | Y:12.52 | N:16.01 | Y:10.51 | Y:12.23 | N:70.34 | N:10.25 | N:17.13 | 5 | 0.22 | 0 | 0 | 0.63 |  |  |  |  |  |
| 4 | 48096947 | 48097615 | Y:28.23 | N:31.44 | N:31.45 | Y:30.15 | N:29.74 | N:34.63 | N:34.77 | N:32.92 | N:34.72 | N:24.84 | Y:30.71 | N:30.36 | N:12.1 | N:14.34 | N:19.58 | N:16.38 | N:15.47 | N:19 | N:14.33 | N:21.64 | N:57.54 | N:15.9 | N:26.11 | 3 | 0.13 | 0.33 | 0.17 | 0 |  |  |  |  |  |
| 4 | 55419659 | 55420314 | N:27.58 | N:31.26 | N:34.95 | N:31.44 | N:30.62 | N:31.36 | N:30.06 | N:30.75 | N:31.49 | N:26.03 | N:30.59 | N:25.87 | N:7.33 | N:13.53 | N:15.92 | N:16.26 | N:11.99 | N:18.1 | N:11.58 | N:13.89 | Y:56.52 | N:18.33 | N:36.92 | 1 | 0.04 | 0 | 0 | 0 |  |  |  |  |  |
| 4 | 58818773 | 58819243 | N:31.9 | N:30.46 | N:34.08 | N:28.21 | N:33.82 | N:31.21 | N:32.39 | N:30.1 | N:32.58 | N:26.99 | N:26.09 | N:34.53 | N:10.74 | N:16.58 | N:21.35 | N:23.36 | N:12.89 | N:26.48 | N:15.52 | Y:19.53 | N:66.57 | N:27.33 | N:29.68 | 1 | 0.04 | 0 | 0 | 0.13 |  |  |  |  |  |
| 4 | 62324172 | 62324557 | N:31.94 | N:26.81 | N:35.52 | N:30.27 | Y:33.06 | N:37.99 | N:30.67 | N:28.23 | N:35.12 | N:16.04 | N:29.7 | N:29.56 | N:3.63 | N:6.47 | N:10.75 | N:9.93 | N:6.73 | N:14.06 | N:8.59 | N:7.25 | N:55.12 | N:7.23 | N:16.1 | 1 | 0.04 | 0.17 | 0 | 0 |  |  |  |  |  |
| 4 | 62460729 | 62461331 | N:37.58 | N:34.52 | N:37.67 | N:35.49 | N:31.51 | N:30.33 | N:34.14 | Y:29.57 | N:33.02 | N:26.08 | N:33.85 | N:30.45 | N:6.06 | N:12.98 | N:15.77 | N:8.83 | N:10.69 | N:15.68 | N:12.85 | N:14.1 | N:70.78 | N:11.48 | N:23.82 | 1 | 0.04 | 0 | 0.17 | 0 |  |  |  |  |  |
| 4 | 66767916 | 66768002 | N:33.09 | N:45.37 | N:35.85 | N:44.6 | N:37.95 | N:39.01 | N:35.64 | N:37.52 | N:36.4 | N:23.34 | N:22.99 | N:34.02 | Y:13.32 | N:16.53 | N:22.6 | N:20.45 | N:16.26 | N:28.43 | N:8.64 | N:18.15 | N:65.09 | N:17.76 | N:33.87 | 1 | 0.04 | 0 | 0 | 0.13 |  |  |  |  |  |
| 4 | 68033286 | 68033906 | N:32.82 | Y:30.42 | N:34 | N:32.82 | N:30.97 | N:32.04 | N:37.69 | N:36.14 | N:34.22 | N:25.47 | N:32.63 | N:34.24 | N:11.27 | N:13.37 | N:15.91 | N:12.92 | N:7.81 | N:19.14 | N:15.08 | N:18.37 | N:57.8 | N:34.5 | N:37.47 | 1 | 0.04 | 0.17 | 0 | 0 |  |  |  |  |  |
| 4 | 69679128 | 69679960 | N:28.45 | N:34.93 | N:33.62 | N:36.05 | N:34.18 | Y:29.76 | N:39.47 | N:30.52 | N:33.34 | N:33.59 | N:29.99 | N:31.87 | N:10.06 | N:16.81 | N:14.27 | N:18.39 | N:9.28 | N:22.28 | N:15.44 | N:18.27 | N:61 | N:19.54 | N:32.68 | 1 | 0.04 | 0.17 | 0 | 0 |  |  |  |  |  |
| 4 | 70463742 | 70464439 | N:38.09 | N:35.56 | N:38.35 | Y:40.03 | N:35.11 | N:35.82 | N:36.33 | N:34.24 | N:37.2 | N:30.82 | N:33.64 | N:33.85 | N:8.69 | N:14.49 | Y:22.12 | N:17.45 | N:11.44 | N:26.4 | Y:15.11 | N:17.18 | N:67.23 | N:22.4 | N:28.06 | 3 | 0.13 | 0.17 | 0 | 0.25 |  |  |  |  |  |
| 4 | 70551829 | 70552669 | Y:22.64 | N:17.04 | N:20.25 | Y:25.96 | Y:18.52 | Y:23.92 | N:17.34 | N:18.24 | N:19.12 | N:14.3 | N:17.18 | Y:19.4 | Y:5.86 | Y:10.1 | N:9.02 | Y:12.08 | N:7.1 | N:12.87 | N:6.52 | N:9.24 | N:46.13 | Y:17.86 | Y:24.4 | 10 | 0.43 | 0.67 | 0.17 | 0.38 |  |  |  |  |  |
| 4 | 71535538 | 71536391 | N:27.23 | N:27.54 | Y:29.14 | N:29.13 | N:27.93 | N:30.31 | N:28.27 | N:28.84 | N:28.33 | N:20.43 | N:24.71 | Y:26.82 | N:5.59 | N:8.7 | N:12.42 | N:9.01 | N:8.59 | N:14.64 | N:8.67 | N:10.09 | N:56.09 | Y:9.17 | N:17.05 | 3 | 0.13 | 0.17 | 0.17 | 0 |  |  |  |  |  |
| 4 | 72515182 | 72515626 | N:28.81 | N:33.57 | N:38.12 | N:32.71 | N:37.54 | N:34.75 | N:34.32 | N:31.77 | N:35.01 | N:28.89 | N:33.21 | N:30.6 | N:6.8 | N:9.91 | Y:13.08 | N:18.91 | N:8.91 | N:19.11 | Y:9.9 | N:12.68 | N:63.66 | N:10.37 | N:16.16 | 2 | 0.09 | 0 | 0 | 0.25 |  |  |  |  |  |
| 4 | 72705264 | 72705454 | N:28.73 | N:30.26 | Y:34.7 | N:33.72 | N:32.63 | N:30.39 | N:33.1 | N:34.13 | N:33.81 | N:24.17 | N:35.14 | Y:27.72 | N:8.12 | N:14.82 | N:15.8 | N:19.93 | N:9.59 | N:22.59 | N:11.86 | N:21.43 | N:67.98 | N:9.89 | N:26.24 | 2 | 0.09 | 0.17 | 0.17 | 0 |  |  |  |  |  |
| 4 | 75154549 | 75155039 | N:36.33 | Y:31.33 | N:35.66 | N:43.35 | N:35.06 | N:36.12 | N:33.44 | N:31.98 | N:35.1 | N:26.46 | N:28.35 | N:36.65 | N:11.63 | N:18.5 | N:12.03 | N:12.29 | N:9.99 | N:25.42 | N:15.33 | N:20.45 | N:52.86 | N:28.29 | Y:28.54 | 2 | 0.09 | 0.17 | 0 | 0 |  |  |  |  |  |
| 4 | 78859384 | 78859987 | N:36.14 | N:37.54 | N:40.21 | N:29.58 | N:40.52 | N:35.58 | N:32.33 | N:39.24 | N:31.36 | N:23.86 | N:34.44 | N:34.67 | Y:11.69 | Y:15.16 | Y:13.13 | N:14.3 | N:13.34 | Y:19.19 | N:10.02 | N:11.23 | N:63.12 | N:17.4 | Y:25.67 | 5 | 0.22 | 0 | 0 | 0.5 |  |  |  |  |  |
| 4 | 78901609 | 78902511 | N:30.9 | N:32.01 | N:31.32 | N:31.22 | N:35.8 | N:32.12 | N:34.61 | Y:30.76 | Y:31.14 | Y:25.04 | N:31.27 | N:30.72 | N:10.87 | N:20.27 | N:18.57 | N:14.53 | N:12.79 | N:21.38 | N:13.07 | N:22.96 | N:63.52 | N:24.33 | N:22.95 | 3 | 0.13 | 0 | 0.5 | 0 |  |  |  |  |  |
| 4 | 86220862 | 86221807 | Y:28.75 | Y:32.66 | Y:32.13 | N:32.72 | N:35.98 | Y:34.49 | Y:28.14 | Y:27.52 | N:38.95 | N:27.81 | Y:31.46 | N:33.79 | N:8.59 | N:16.32 | Y:14.67 | Y:15.65 | Y:10.39 | Y:17.97 | N:10.7 | N:15.58 | N:66.6 | N:15.97 | N:19.38 | 11 | 0.48 | 0.67 | 0.5 | 0.5 |  |  |  |  |  |
| 5 | 2705052 | 2706284 | Y:30.6 | Y:30.97 | Y:30.8 | Y:30.93 | Y:29.81 | Y:30.96 | Y:32.16 | Y:30.37 | Y:27 | Y:23.05 | Y:26.01 | Y:29.86 | Y:14.86 | Y:23.19 | Y:28 | Y:18.7 | Y:9.45 | Y:25.06 | Y:25.18 | Y:26.42 | Y:53 | Y:43.32 | Y:48.16 | 23 | 1 | 1 | 1 | 1 |  |  |  |  |  |
| 5 | 4202332 | 4203178 | N:33.89 | Y:33.41 | Y:34.26 | N:34.53 | N:32.74 | N:39.22 | N:32.02 | N:35.4 | N:32.94 | N:27.65 | N:39.57 | N:33.37 | N:9.19 | N:11.33 | N:15 | N:14.26 | N:11.56 | N:19.08 | N:11.3 | N:15.59 | N:55.62 | N:13.48 | N:27.84 | 2 | 0.09 | 0.33 | 0 | 0 |  |  |  |  |  |
| 5 | 4737109 | 4737429 | N:23.98 | N:30.08 | N:23.64 | N:32.83 | N:30.58 | N:33.83 | N:27.74 | N:31.06 | N:29.47 | N:29.78 | N:29.7 | N:30.89 | N:4.2 | N:11.08 | N:18.63 | N:13.52 | N:14.81 | N:17.61 | N:14.68 | N:10.02 | Y:55.73 | N:13.49 | N:16.11 | 1 | 0.04 | 0 | 0 | 0 |  |  |  |  |  |
| 5 | 6164269 | 6165523 | Y:19.21 | Y:19.66 | Y:20.22 | Y:23.87 | N:19.68 | Y:20.77 | N:15.68 | N:16.64 | Y:27.35 | Y:21.82 | N:16.87 | Y:20.11 | Y:12.93 | Y:26.77 | Y:24.92 | N:13.21 | N:7.59 | Y:24.89 | Y:24.07 | N:16.72 | Y:55.93 | Y:50.07 | N:39.62 | 15 | 0.65 | 0.83 | 0.5 | 0.63 |  |  |  |  |  |
| 5 | 10249946 | 10250591 | N:30.74 | N:30.28 | N:36.39 | N:34.58 | N:34.37 | N:38.4 | N:35.87 | N:29.94 | N:32.58 | N:27.37 | N:35.24 | N:32.03 | N:9.69 | N:12.4 | N:13.6 | N:12.56 | N:12.46 | N:21.19 | N:11.74 | N:13 | N:55.51 | Y:21.54 | N:25.06 | 1 | 0.04 | 0 | 0 | 0 |  |  |  |  |  |
| 5 | 19126313 | 19126773 | N:30.92 | N:33.37 | N:34.99 | N:34.28 | N:38.73 | N:35.26 | N:34.42 | N:34.76 | N:36.05 | N:26.55 | N:34.58 | N:28.74 | N:9.65 | N:11.78 | N:11.79 | N:14.17 | N:6.7 | N:16.34 | N:12.14 | N:8.17 | Y:54.89 | N:7.14 | N:13.84 | 1 | 0.04 | 0 | 0 | 0 |  |  |  |  |  |
| 5 | 19302240 | 19303103 | N:30.52 | N:34.14 | N:33.56 | N:30.67 | N:34.46 | N:31.59 | N:40.66 | N:36.98 | N:34.34 | N:24.97 | N:32.23 | N:31.45 | N:10.31 | N:15.7 | Y:21.54 | N:13.88 | N:13.19 | N:22.07 | Y:13.63 | N:15.85 | N:65.14 | N:23.26 | N:22.06 | 2 | 0.09 | 0 | 0 | 0.25 |  |  |  |  |  |
| 5 | 31303866 | 31305068 | Y:29.46 | Y:31.33 | Y:33.51 | Y:34.43 | Y:33.42 | Y:35.9 | Y:34.09 | Y:34.59 | Y:31.41 | Y:25.99 | Y:34.32 | Y:33.72 | Y:10.65 | Y:17.2 | Y:17.28 | Y:12.98 | Y:10.67 | Y:19.67 | Y:16.13 | Y:15.5 | Y:58.28 | Y:19.72 | Y:23.76 | 23 | 1 | 1 | 1 | 1 |  |  |  |  |  |
| 5 | 31525368 | 31526271 | N:31.01 | Y:30.82 | Y:31.49 | N:33.43 | N:33.19 | Y:32.62 | N:39.52 | N:32.16 | N:35.34 | N:26.22 | N:32.05 | N:33.17 | N:8.36 | Y:14.22 | N:16.89 | N:15.06 | N:13.62 | N:25.61 | Y:14.38 | N:17.72 | N:60.89 | N:19.85 | N:34.12 | 5 | 0.22 | 0.5 | 0 | 0.25 |  |  |  |  |  |
| 5 | 31898626 | 31899883 | Y:29 | Y:27.68 | Y:31.88 | Y:31.25 | Y:33.52 | Y:39.06 | Y:29.47 | Y:36.5 | Y:30.03 | Y:29.92 | Y:32.34 | Y:32.36 | Y:8.03 | Y:16 | Y:19 | Y:18.8 | Y:11.32 | Y:21.17 | Y:13.28 | Y:16.69 | Y:58.13 | Y:21.63 | Y:26.41 | 23 | 1 | 1 | 1 | 1 |  |  |  |  |  |
| 5 | 31998141 | 31999034 | N:34.92 | Y:35.91 | Y:29.55 | Y:29.33 | Y:32.87 | Y:29.23 | N:36.12 | Y:30.77 | Y:35.16 | Y:28.51 | Y:31.6 | Y:31.19 | N:9.69 | N:14.14 | N:17.64 | N:19.59 | N:15.61 | N:26.19 | N:13.92 | N:18.52 | N:56.35 | N:18.15 | Y:22.8 | 11 | 0.48 | 0.83 | 0.83 | 0 |  |  |  |  |  |
| 5 | 39163484 | 39164645 | Y:26.6 | N:24.51 | N:23.89 | Y:28.99 | N:25.17 | N:24.48 | N:24.28 | N:21.96 | N:24.14 | N:19.16 | Y:26.68 | N:20.93 | N:6.12 | N:10.16 | N:13.13 | N:9.56 | N:9.02 | N:14.62 | N:11.38 | N:10.65 | Y:58.13 | Y:23.45 | Y:29.3 | 6 | 0.26 | 0.33 | 0.17 | 0 |  |  |  |  |  |
| 5 | 40463978 | 40464915 | Y:33.82 | N:36.99 | Y:35.65 | Y:30.58 | N:33.37 | Y:31.83 | N:40.52 | N:36.25 | N:33.12 | N:27.86 | N:33.05 | Y:35.51 | N:9.26 | N:19.57 | N:14.52 | N:13.38 | N:10.09 | N:23.02 | Y:15.73 | N:16.57 | N:63.5 | Y:22.51 | N:28.28 | 7 | 0.3 | 0.67 | 0.17 | 0.13 |  |  |  |  |  |
| 5 | 40954947 | 40955227 | N:31.2 | N:36.23 | N:32.34 | N:39.29 | N:29.54 | N:36.72 | N:34.04 | N:39.31 | N:36.69 | N:28.53 | N:35.5 | N:30.83 | N:10.44 | N:18.43 | N:18.56 | N:10.21 | N:11.54 | N:29.26 | N:15.02 | N:14.19 | N:59.2 | N:18.77 | Y:33.61 | 1 | 0.04 | 0 | 0 | 0 |  |  |  |  |  |
| 5 | 41299361 | 41300140 | Y:31.27 | N:33.23 | N:37.42 | N:36.81 | N:37.92 | N:37.36 | N:35.58 | N:36.02 | N:37.18 | N:31.57 | N:30.38 | N:34.67 | Y:10.96 | N:11.38 | N:10.08 | N:12.43 | N:10.23 | Y:19.89 | N:10.65 | Y:13.28 | N:60.97 | N:16.37 | N:21.44 | 4 | 0.17 | 0.17 | 0 | 0.38 |  |  |  |  |  |
| 5 | 55530364 | 55531097 | N:30.48 | N:23.56 | N:28.74 | N:28.64 | N:23.12 | N:30.32 | N:22.72 | N:23.08 | N:26.62 | N:27.54 | N:29.21 | N:28.27 | N:12.58 | N:17.59 | N:15.44 | N:16.04 | Y:10.84 | Y:20.42 | N:16.23 | N:14.68 | N:64.31 | N:29.49 | N:39.99 | 2 | 0.09 | 0 | 0 | 0.25 |  |  |  |  |  |
| 6 | 5291837 | 5292712 | N:31.49 | N:27.53 | N:36.43 | N:31.88 | N:32.57 | N:35.46 | N:33.04 | Y:32.76 | N:35.17 | N:26.78 | Y:31.42 | N:29.71 | N:7.88 | N:10.9 | N:18.18 | N:18.12 | N:12.03 | N:18.14 | N:16.18 | N:14.59 | N:62.85 | N:18.55 | N:27.2 | 2 | 0.09 | 0 | 0.33 | 0 |  |  |  |  |  |
| 6 | 9811191 | 9816444 | Y:29.13 | Y:28.6 | Y:28.61 | Y:29.76 | Y:28.26 | Y:29.95 | Y:24.86 | Y:32.03 | Y:27.91 | Y:23.45 | Y:24.67 | Y:26.7 | Y:13.57 | Y:21.53 | Y:23.53 | Y:18.58 | Y:12.85 | Y:26.42 | Y:20.61 | Y:23.61 | Y:53.9 | Y:38.6 | Y:56.38 | 23 | 1 | 1 | 1 | 1 |  |  |  |  |  |
| 6 | 13724843 | 13726022 | Y:31.64 | Y:30.74 | Y:33.6 | Y:33.33 | Y:35.02 | Y:33.66 | Y:31.97 | Y:31.22 | Y:30.99 | Y:28.61 | Y:31.45 | Y:32.9 | Y:8.76 | Y:20.08 | Y:19.41 | Y:14.64 | Y:9.27 | Y:25.78 | Y:19.06 | Y:17.99 | Y:58.58 | Y:29.32 | Y:28.54 | 23 | 1 | 1 | 1 | 1 |  |  |  |  |  |
| 6 | 25237263 | 25237909 | N:31.81 | N:28.21 | N:33.64 | N:27.41 | N:31.85 | N:30.93 | N:27.15 | N:32.73 | N:29.24 | N:26.21 | N:29.87 | N:31.35 | N:8.86 | N:16.94 | N:25.15 | Y:17.1 | N:12.4 | N:22.34 | N:18.75 | N:22.56 | N:64.14 | N:33.25 | Y:39.97 | 2 | 0.09 | 0 | 0 | 0.13 |  |  |  |  |  |
| 6 | 26190297 | 26191167 | N:32.67 | N:31.21 | N:33.33 | N:29.76 | N:32.39 | N:34.58 | N:29.32 | N:31.33 | N:32.66 | N:27.15 | N:29.92 | N:27.66 | N:11.5 | Y:17.77 | Y:20.73 | N:16.12 | N:11.07 | N:23.55 | N:15.81 | N:18.42 | N:58.85 | N:34.45 | N:40.44 | 2 | 0.09 | 0 | 0 | 0.25 |  |  |  |  |  |
| 6 | 33482245 | 33483060 | N:28.03 | N:27.86 | N:35.04 | N:33.53 | N:27.53 | N:32.95 | N:31.36 | N:34.46 | N:34.63 | N:24.54 | Y:28.79 | N:27.42 | N:8.26 | N:17.65 | N:18.45 | N:15.58 | N:14.19 | N:22.81 | N:13.38 | N:18.79 | N:56.65 | N:29.93 | N:28.05 | 1 | 0.04 | 0 | 0.17 | 0 |  |  |  |  |  |
| 6 | 33847042 | 33847694 | N:34.04 | Y:33.68 | Y:33.27 | N:34.27 | N:34.74 | N:37.15 | N:36.67 | N:34.81 | N:34.45 | N:25.31 | N:30.57 | N:33.17 | N:8.61 | N:20.73 | N:15.47 | N:18.16 | N:9.6 | N:32.76 | N:15.77 | N:12.58 | N:59.14 | N:26.18 | N:32.09 | 2 | 0.09 | 0.33 | 0 | 0 |  |  |  |  |  |
| 7 | 2507955 | 2508579 | N:33.2 | Y:31.35 | N:38.88 | N:35.98 | N:33.01 | N:38.33 | N:36.75 | N:34.28 | N:37.21 | N:31.15 | N:39.33 | N:33.32 | N:8.19 | N:13.99 | N:22.48 | N:20.9 | N:16.08 | N:24.8 | N:11.93 | N:17.71 | N:78.97 | N:21.63 | N:26.74 | 1 | 0.04 | 0.17 | 0 | 0 |  |  |  |  |  |
| 7 | 8070564 | 8071157 | N:35.87 | N:33.72 | N:31.61 | N:37.72 | Y:31.1 | N:34.47 | N:37.61 | N:38.36 | N:38.61 | N:26.87 | N:33.25 | N:34.35 | N:12.32 | N:13.75 | N:19.03 | N:22.63 | N:10.2 | N:23.78 | N:12.34 | N:13.47 | N:76.46 | N:19.55 | N:28.6 | 1 | 0.04 | 0.17 | 0 | 0 |  |  |  |  |  |
| 7 | 9022731 | 9023457 | Y:30.13 | N:32.29 | Y:30.83 | N:33.44 | N:34.04 | N:35.08 | Y:30.55 | Y:33.04 | Y:31.84 | Y:23.86 | Y:33.43 | Y:31.07 | N:7.88 | N:14.58 | N:21.36 | N:16.93 | N:13.18 | N:28.9 | N:16.11 | N:18.94 | N:58.41 | Y:29.59 | Y:39.03 | 10 | 0.43 | 0.33 | 1 | 0 |  |  |  |  |  |
| 7 | 9139826 | 9140674 | N:33.58 | N:29.72 | N:32.65 | N:34.24 | N:34.94 | N:33.61 | N:34.55 | N:31.91 | N:38.49 | N:28.16 | Y:29.49 | N:32.25 | N:10.87 | N:17.46 | N:21.08 | N:16.6 | N:10.77 | N:23.88 | N:18.27 | N:21.03 | N:70.77 | N:21.78 | N:30.29 | 1 | 0.04 | 0 | 0.17 | 0 |  |  |  |  |  |
| 7 | 15504223 | 15504887 | N:36.81 | Y:35.05 | N:33.36 | Y:35.92 | N:34.7 | N:33.51 | Y:34.22 | Y:32.32 | Y:35.62 | Y:21.51 | N:30.95 | Y:29.22 | N:5.58 | N:15.25 | N:17.13 | N:17.44 | N:9.65 | N:17.79 | N:12.04 | N:11.95 | N:64.71 | N:10.05 | N:18.67 | 7 | 0.3 | 0.33 | 0.83 | 0 |  |  |  |  |  |
| 7 | 17708068 | 17708753 | Y:29.75 | Y:27.55 | Y:31.15 | N:31.75 | Y:31.78 | Y:32.44 | Y:29.12 | Y:32.21 | Y:30.32 | Y:27.63 | Y:31.14 | Y:31.44 | Y:12.57 | Y:24.51 | Y:22.81 | N:18.69 | N:14.6 | Y:23.8 | Y:15.93 | N:20.69 | N:56.21 | Y:35.37 | N:24.51 | 17 | 0.74 | 0.83 | 1 | 0.63 |  |  |  |  |  |
| 7 | 26115687 | 26116356 | N:31.28 | N:30 | N:27.81 | N:30.41 | N:26.49 | Y:31.23 | N:27.13 | Y:29.07 | N:29 | N:27.81 | N:29.46 | N:31.8 | N:9.2 | N:15 | N:19.42 | N:15.88 | N:8.83 | N:23.57 | N:11.52 | N:19 | N:63.56 | N:26.44 | N:30.05 | 2 | 0.09 | 0.17 | 0.17 | 0 |  |  |  |  |  |
| 7 | 29830070 | 29830722 | N:24.86 | N:32.73 | N:30.83 | N:30.04 | Y:31.63 | N:31.82 | N:34.21 | N:28.85 | N:28.84 | N:23.67 | N:31.21 | N:29.75 | N:10.95 | N:25.28 | N:19.38 | Y:19.67 | N:17.74 | N:24.32 | N:19.16 | Y:15.58 | N:62.28 | N:41.46 | N:52.34 | 3 | 0.13 | 0.17 | 0 | 0.25 |  |  |  |  |  |
| 8 | 8899619 | 8900337 | N:10.97 | N:14.44 | N:18.51 | N:15.24 | N:15.37 | N:14.78 | N:15.08 | N:15.76 | N:17.59 | N:10.72 | N:15.64 | N:12.92 | N:7.33 | N:6.22 | N:12.43 | N:9.65 | N:5.96 | N:10.85 | N:10.48 | N:11.42 | Y:56.2 | N:17.23 | N:24.96 | 1 | 0.04 | 0 | 0 | 0 |  |  |  |  |  |
| 8 | 9208394 | 9209273 | Y:17.61 | N:14.61 | Y:22.67 | Y:20.04 | Y:23.88 | Y:16 | Y:19.77 | Y:21.84 | Y:21.63 | Y:11.53 | Y:16.25 | Y:17.77 | Y:5.5 | Y:5.37 | Y:10.58 | N:6.62 | Y:6.39 | Y:13.26 | N:4.2 | N:8.34 | N:47.55 | N:6.22 | N:14.99 | 16 | 0.7 | 0.83 | 1 | 0.63 |  |  |  |  |  |
| 8 | 11644653 | 11645314 | N:30.77 | N:32.21 | N:32.24 | N:34.77 | N:34.73 | N:33.1 | N:32.48 | Y:29.31 | Y:35.31 | Y:18.44 | Y:24.16 | Y:29.43 | N:4.92 | N:10.32 | N:16.42 | N:8.04 | N:8.28 | N:18.14 | N:8.5 | N:14.63 | N:57.57 | N:8.69 | N:19.74 | 5 | 0.22 | 0 | 0.83 | 0 |  |  |  |  |  |
| 8 | 17696796 | 17697651 | N:31.01 | N:29.01 | N:33.76 | N:34.43 | N:32.95 | N:33.5 | Y:34.75 | N:35.18 | N:31.22 | N:28.87 | N:33.18 | N:30.55 | N:5.78 | N:18.19 | N:16.06 | N:16.07 | N:11.5 | N:18.73 | N:15.05 | N:13.1 | N:62.2 | Y:18.83 | N:25.5 | 2 | 0.09 | 0 | 0.17 | 0 |  |  |  |  |  |
| 8 | 23631239 | 23632111 | N:31.8 | N:31.17 | N:30.15 | N:33.01 | N:30.34 | N:37.21 | N:32.1 | Y:29.12 | N:32.73 | N:29.47 | N:30.5 | N:28.98 | N:12.47 | N:19.62 | N:23.42 | N:15.85 | N:12.84 | N:25.29 | N:16.46 | N:22.66 | N:60.5 | N:41.89 | N:35.03 | 1 | 0.04 | 0 | 0.17 | 0 |  |  |  |  |  |
| 9 | 1710352 | 1710900 | N:21.11 | Y:26.77 | N:27.15 | N:22.11 | N:18.45 | Y:20.82 | N:20.3 | N:23.06 | N:26.42 | N:21.4 | N:20.15 | N:26.71 | N:9.09 | N:13.87 | N:14.29 | N:10.11 | N:10.63 | N:20.8 | N:16.93 | N:13.66 | N:53.26 | N:36.93 | N:31.57 | 2 | 0.09 | 0.33 | 0 | 0 |  |  |  |  |  |
| 9 | 5602574 | 5603257 | Y:30.91 | N:34.2 | N:38.46 | N:35.58 | N:38.1 | Y:30.45 | N:35.93 | N:31.78 | Y:34 | Y:26.76 | Y:33.27 | Y:31.79 | N:10.75 | N:16.54 | N:19.14 | N:16.16 | N:12.53 | N:21.89 | N:14.25 | N:14.76 | N:65.32 | N:19.66 | N:28.29 | 6 | 0.26 | 0.33 | 0.67 | 0 |  |  |  |  |  |
| 9 | 18008576 | 18009249 | N:20.37 | N:20.2 | Y:24.27 | N:22.13 | Y:23.63 | N:25.79 | N:20.77 | N:20.86 | N:20.77 | N:13.57 | N:16.66 | N:25 | N:5.71 | N:14.76 | N:12.54 | N:11.78 | N:11.52 | Y:20.37 | N:15.1 | N:14.16 | N:58.28 | N:21.22 | N:34.93 | 3 | 0.13 | 0.33 | 0 | 0.13 |  |  |  |  |  |
| 9 | 21346066 | 21346914 | N:29.9 | Y:26.96 | Y:31.57 | N:30.65 | Y:29.49 | N:30.75 | N:28.16 | N:33.48 | N:32.08 | N:27.55 | N:26.64 | N:24.38 | N:14.37 | N:19.26 | N:15.85 | N:17.85 | N:11.01 | N:21.67 | N:16.17 | N:17.65 | N:64.01 | N:27.08 | N:34.94 | 3 | 0.13 | 0.5 | 0 | 0 |  |  |  |  |  |
| AADN03009901.1 | 851 | 1161 | N:32.98 | N:30.45 | N:32.57 | N:30.39 | N:26.83 | N:31.38 | N:24.7 | N:27.1 | N:26.97 | N:23.05 | N:28.68 | N:30.75 | N:7.48 | N:18.87 | N:30.44 | N:18.85 | N:16.37 | N:23.74 | N:15.89 | N:21.61 | N:48.12 | N:77.06 | N:59.82 | 0 | 0 | 0 | 0 | 0 |  |  |  |  |  |
| JH375157.1 | 356 | 536 | Y:20.28 | N:11.65 | NA | N:11.48 | Y:26.52 | N:2.11 | N:1.03 | N:22.6 | N:12.52 | N:7.4 | N:2.18 | N:12.1 | N:5.35 | N:15.72 | N:14.65 | N:21.28 | N:12.15 | N:21.02 | N:18.76 | N:1.55 | N:31.86 | Y:26.24 | N:45.1 | 3 | 0.14 | 0.4 | 0 | 0 |  |  |  |  |  |
| JH375968.1 | 8535 | 8818 | Y:157.5 | N:217.26 | N:163.21 | N:204.26 | N:192.05 | N:169.29 | N:213.25 | N:225.29 | N:223.11 | N:190.88 | N:177.43 | N:226.7 | N:62.7 | N:108.43 | N:191.2 | N:167.84 | N:60.59 | N:117.69 | N:133.32 | N:91.78 | N:783.61 | N:298.16 | N:475.23 | 1 | 0.04 | 0.17 | 0 | 0 |  |  |  |  |  |
| JH376323.1 | 928 | 1072 | Y:114.68 | Y:87.56 | N:93.6 | N:82.34 | N:97.73 | N:75.46 | N:70.62 | Y:108.57 | N:56.74 | N:49.37 | N:95.73 | Y:55.88 | N:25.58 | N:163.21 | N:29.34 | N:33.98 | N:8.59 | N:51.68 | N:80.68 | N:28.11 | N:44.57 | N:347.01 | N:325.32 | 4 | 0.17 | 0.33 | 0.33 | 0 |  |  |  |  |  |
| JH376323.1 | 17955 | 18124 | N:80.84 | Y:87.56 | N:52.32 | N:97.51 | N:66.28 | N:35.25 | N:41.58 | Y:108.57 | N:52.43 | N:46.72 | N:73.05 | Y:55.88 | N:23.25 | N:39.74 | N:26.04 | N:47.17 | N:22.42 | N:63.68 | N:21.68 | N:31.36 | N:98.21 | N:154.11 | N:164.29 | 3 | 0.13 | 0.17 | 0.33 | 0 |  |  |  |  |  |
| Z | 3540391 | 3541231 | N:35.82 | N:30.56 | N:35.72 | N:33.98 | N:30.35 | N:35.86 | N:35.49 | N:31.04 | N:30.39 | N:28.76 | N:32.64 | N:18.25 | N:4.45 | N:7.79 | N:11.27 | N:9.31 | N:14.27 | N:12.47 | N:8.29 | N:11.74 | N:29.86 | Y:20.28 | N:16.83 | 1 | 0.04 | 0 | 0 | 0 |  |  |  |  |  |
| Z | 15470956 | 15471752 | N:17.49 | N:18.62 | N:18.91 | N:20.8 | Y:24.01 | N:21.62 | N:18.27 | N:23.42 | N:19.87 | N:12.04 | N:20.6 | N:11.56 | N:3.4 | N:3.65 | N:5.12 | Y:6.32 | Y:7.68 | Y:8.16 | N:6.26 | N:4.49 | N:22.82 | N:12.29 | Y:12.19 | 5 | 0.22 | 0.17 | 0 | 0.38 |  |  |  |  |  |
| Z | 18807991 | 18809115 | Y:32.44 | Y:33.35 | Y:30.03 | Y:34.98 | Y:30.11 | Y:27.83 | Y:30.59 | Y:30.38 | Y:30.92 | Y:24.97 | Y:31.38 | Y:14.56 | N:3.44 | N:11.19 | N:11.82 | N:10.05 | N:14.61 | N:15.01 | N:8.02 | N:14.02 | Y:28.92 | Y:35.74 | Y:21.93 | 15 | 0.65 | 1 | 1 | 0 |  |  |  |  |  |
| Z | 30996146 | 31000811 | N:21.58 | Y:23.72 | Y:30.7 | N:17.3 | N:24.41 | Y:30.77 | N:32.32 | N:28.49 | N:18.25 | N:16.24 | N:37.18 | N:19.04 | N:12.06 | Y:15.75 | Y:26.85 | Y:19.7 | Y:11.07 | Y:23.72 | N:8.06 | N:16.04 | Y:30.45 | Y:22.92 | Y:57.7 | 11 | 0.48 | 0.5 | 0 | 0.63 |  |  |  |  |  |
| Z | 32081154 | 32086293 | Y:55.12 | Y:53.28 | Y:53.58 | Y:51.34 | Y:68.62 | Y:70.1 | Y:39.69 | Y:48.38 | Y:36.28 | Y:30.05 | Y:42.22 | Y:32.64 | N:16.13 | Y:29.23 | Y:28.46 | Y:23.39 | Y:12.07 | Y:27.27 | Y:18 | Y:26.27 | Y:35.83 | Y:59.5 | Y:78.78 | 22 | 0.96 | 1 | 1 | 0.88 |  |  |  |  |  |
| Z | 47227949 | 47228506 | N:31.19 | N:33.48 | N:35.99 | N:29.35 | N:33.34 | N:36.51 | N:29.35 | N:29.34 | N:30.13 | N:30.57 | N:32.85 | N:16.66 | N:6.02 | N:8.13 | N:13.08 | N:10.32 | N:14.46 | N:16.13 | N:12.38 | N:11.16 | N:29.19 | N:34.87 | Y:17.36 | 1 | 0.04 | 0 | 0 | 0 |  |  |  |  |  |
| Z | 47830813 | 47831469 | N:32.41 | N:30.63 | N:32.47 | Y:31.12 | N:35.67 | N:28.3 | N:30.18 | N:31.73 | N:35.47 | N:26.33 | N:29.96 | N:16.59 | N:3.94 | N:7.78 | N:7.39 | N:10.26 | N:7.86 | N:9.3 | N:6 | N:7.21 | N:34.07 | N:19.69 | N:16.26 | 1 | 0.04 | 0.17 | 0 | 0 |  |  |  |  |  |
| Z | 49125790 | 49126200 | N:33.85 | N:37.49 | N:36.15 | N:29.31 | N:33.11 | N:36.72 | N:41.15 | N:37.64 | N:32.97 | N:24.38 | N:36.98 | N:15.79 | Y:3.22 | N:5 | N:6.35 | N:5.94 | N:8.32 | N:8.23 | N:3.22 | N:5.98 | N:35.54 | N:4.51 | N:13.18 | 1 | 0.04 | 0 | 0 | 0.13 |  |  |  |  |  |
| Z | 50801143 | 50801818 | N:30.39 | N:32.44 | N:35.29 | N:31.45 | N:32.17 | N:35.96 | N:38.42 | N:34.35 | Y:28.05 | Y:19.16 | Y:30.23 | N:15.38 | N:6.07 | N:7.92 | N:7.42 | N:5.21 | N:13.79 | N:10.01 | N:6.51 | N:6.27 | N:27.08 | N:14.92 | N:9.57 | 3 | 0.13 | 0 | 0.5 | 0 |  |  |  |  |  |
| Z | 50872653 | 50873337 | Y:33.99 | Y:33.65 | Y:40.33 | Y:35.28 | Y:35.08 | Y:32.67 | Y:37.71 | N:38.36 | N:35.86 | N:26.54 | N:33.55 | N:18.97 | N:6.13 | N:3.5 | N:6.98 | N:7.98 | N:10.3 | N:10.32 | N:5.53 | Y:5.33 | N:31.28 | N:13.39 | N:8.15 | 8 | 0.35 | 1 | 0.17 | 0.13 |  |  |  |  |  |
| Z | 57973076 | 57973674 | N:36.19 | N:32.73 | N:34.62 | N:29.84 | N:33.36 | N:36.62 | N:29.99 | N:36.3 | N:34.68 | N:23.07 | N:30.3 | N:14.76 | N:4.25 | N:7.53 | N:7.71 | N:6.13 | N:11.03 | N:14.51 | N:7.88 | N:7.46 | N:27.71 | Y:23.23 | N:9.52 | 1 | 0.04 | 0 | 0 | 0 |  |  |  |  |  |
| Z | 61304819 | 61306021 | Y:34.03 | Y:34.44 | Y:36.57 | Y:33.81 | Y:34.38 | Y:35.4 | Y:32.41 | Y:36.39 | Y:35.48 | Y:24.04 | Y:37.29 | Y:18.12 | Y:5.15 | Y:9.21 | Y:13.6 | Y:12.31 | Y:15.32 | Y:14.89 | Y:9.89 | Y:11.28 | Y:30.4 | Y:32.04 | Y:17.43 | 23 | 1 | 1 | 1 | 1 |  |  |  |  |  |
| Z | 64336432 | 64337248 | N:19.46 | N:18.03 | N:19.17 | N:18.76 | N:18.39 | N:14.23 | N:16.47 | N:17.82 | N:16.62 | N:10.79 | N:17.01 | N:8.47 | N:4.8 | N:9.66 | N:10.44 | N:7.38 | N:7.02 | N:9.08 | N:5.89 | N:6.98 | Y:34.21 | N:19.89 | N:23.71 | 1 | 0.04 | 0 | 0 | 0 |  |  |  |  |  |
| Z | 64574244 | 64574638 | N:25.69 | N:31.94 | N:29.18 | N:31.92 | N:28.15 | N:30.3 | N:24.97 | N:29.55 | N:28.89 | N:21.16 | N:29.35 | N:12.1 | Y:6.55 | N:6.08 | N:11.18 | N:7.27 | N:2.93 | N:9.79 | NA | N:2.31 | N:22.04 | N:29.21 | N:20.07 | 1 | 0.05 | 0 | 0 | 0.14 |  |  |  |  |  |
| Z | 65689607 | 65690259 | N:33.67 | N:32.74 | Y:35.12 | N:29.76 | N:30.42 | N:32.61 | N:35.06 | N:34.67 | N:39.23 | N:26.38 | N:28.2 | N:16.03 | N:4.42 | N:10.3 | N:10.34 | N:11.19 | N:9.27 | N:11.27 | N:9.33 | N:12.82 | N:31.67 | N:24.35 | N:14.61 | 1 | 0.04 | 0.17 | 0 | 0 |  |  |  |  |  |
| Z | 69879377 | 69879901 | N:33.87 | N:31.55 | N:33.86 | N:35.76 | N:32.1 | N:35.09 | N:31.2 | N:34.87 | N:32.05 | N:26.63 | N:28.86 | N:20.26 | N:4.65 | N:7.16 | N:7.38 | N:7.16 | N:11.93 | N:7.34 | N:4.93 | N:6.99 | N:31.01 | Y:16.37 | N:8.96 | 1 | 0.04 | 0 | 0 | 0 |  |  |  |  |  |
| Z | 78844863 | 78846057 | N:20.64 | Y:25.79 | Y:31.6 | N:17.69 | Y:21.38 | N:21.59 | Y:30.59 | Y:22.15 | Y:26.22 | Y:21.07 | N:19.45 | N:8.23 | N:8.48 | N:10.8 | N:11.02 | N:12.79 | N:10.75 | N:13.95 | N:6.48 | N:8.96 | Y:27.06 | N:24.1 | N:33.35 | 8 | 0.35 | 0.5 | 0.67 | 0 |  |  |  |  |  |
| Z | 79945501 | 79946432 | Y:27.28 | Y:28.95 | Y:27.05 | Y:28.92 | N:30.13 | Y:30.2 | Y:25.96 | Y:32.93 | Y:26.45 | Y:20.16 | Y:28.2 | Y:13.81 | Y:7.15 | Y:9.12 | Y:12.21 | Y:11.89 | Y:8.8 | Y:10.81 | N:10.73 | N:9.43 | N:30.21 | Y:46.67 | Y:19.78 | 19 | 0.83 | 0.83 | 1 | 0.75 |  |  |  |  |  |
| 11 | 7345413 | 7345603 | N:35.57 | Y:30.7 | N:29.17 | N:28.63 | N:30.02 | N:29.59 | N:35.48 | N:32.2 | N:33.02 | N:25.36 | N:29.71 | N:33.57 | N:7.47 | N:10.25 | N:9.12 | N:8.64 | N:10.2 | N:8.92 | N:6.32 | N:14.85 | N:66.75 | N:15.05 | N:15.92 | 1 | 0.04 | 0.17 | 0 | 0 |  |  |  |  |  |
| 11 | 11174286 | 11174756 | N:35.56 | N:33.42 | N:37.45 | Y:33.07 | N:38.17 | N:33.07 | Y:31.87 | N:37.24 | N:31.36 | N:27.57 | N:31.4 | N:33.21 | N:8.45 | N:18.83 | N:18.8 | N:18.51 | N:10.75 | N:28.19 | N:10.14 | N:13.94 | N:69.96 | Y:20.3 | N:22.89 | 3 | 0.13 | 0.17 | 0.17 | 0 |  |  |  |  |  |
| 11 | 18978682 | 18979268 | N:30.81 | N:29.81 | N:27.94 | N:29.6 | N:29.69 | N:29.46 | N:30.53 | N:34.19 | N:28.61 | N:23.11 | N:29.97 | N:32.77 | N:7.97 | N:19.02 | Y:19.4 | N:18.22 | N:13.39 | N:24.48 | N:19.6 | N:20.9 | N:70.04 | N:35 | Y:40.74 | 2 | 0.09 | 0 | 0 | 0.13 |  |  |  |  |  |
| 15 | 1590630 | 1591272 | N:28.13 | Y:27.07 | N:32.07 | Y:28.48 | N:25.12 | N:30.83 | Y:27.48 | N:31.53 | N:26.55 | N:22.56 | N:25.63 | N:27.63 | N:13.56 | N:21.62 | N:19.43 | N:18.63 | N:12.84 | N:24.57 | N:17.19 | N:23.96 | N:76.46 | N:40.71 | N:36.24 | 3 | 0.13 | 0.33 | 0.17 | 0 |  |  |  |  |  |
| 24 | 5789018 | 5789217 | N:35.16 | N:35.38 | Y:36.63 | N:34.16 | N:32.91 | N:35.96 | N:34.05 | N:35.21 | N:39.95 | N:25.32 | N:36.88 | N:37.58 | N:8.64 | N:13.14 | N:18.9 | N:15.32 | N:13.3 | N:24.83 | N:17.48 | N:21.48 | N:74.53 | N:27.9 | N:32.1 | 1 | 0.04 | 0.17 | 0 | 0 |  |  |  |  |  |
| 13 | 1868296 | 1868957 | N:30.31 | N:25.05 | N:31.03 | Y:31.22 | Y:28.5 | Y:28.04 | N:30.6 | N:29.31 | N:30.81 | N:22.62 | N:27.61 | N:30.97 | N:12.32 | N:21.75 | N:19.44 | N:19.2 | N:10.73 | N:28.3 | N:17.32 | Y:19.94 | N:59.01 | N:37.48 | N:48.33 | 4 | 0.17 | 0.5 | 0 | 0.13 |  |  |  |  |  |
| 13 | 2970495 | 2971368 | N:28.28 | N:27.91 | N:26.43 | N:27.77 | N:28.16 | N:27.74 | N:21.46 | N:28.25 | Y:25.74 | Y:24.77 | N:27.27 | N:27.16 | N:11.17 | N:17.66 | N:25.39 | N:18.64 | N:15.84 | N:24.2 | N:21 | N:16.57 | N:65.99 | N:40.99 | N:35.72 | 2 | 0.09 | 0 | 0.33 | 0 |  |  |  |  |  |
| 13 | 6261859 | 6262487 | N:30.38 | N:34.26 | N:36.55 | N:38.65 | N:33.79 | N:34 | N:32.37 | N:30.61 | N:35.3 | N:30.3 | N:30.27 | N:32.01 | N:10.78 | N:16.64 | N:17.89 | N:20.74 | N:10.73 | N:21.38 | N:11.98 | N:15.61 | N:64.19 | N:19.16 | Y:31.41 | 1 | 0.04 | 0 | 0 | 0 |  |  |  |  |  |
| 13 | 6484735 | 6485598 | N:24.15 | N:25.3 | N:26.23 | N:28.15 | N:24.38 | Y:27.07 | Y:22.95 | N:28.45 | N:24.68 | N:21.26 | N:23 | N:25.82 | N:7.08 | N:13.52 | N:16.47 | N:12.15 | Y:9.42 | Y:20.42 | N:15.2 | N:16 | N:50.6 | N:43.81 | N:48.62 | 4 | 0.17 | 0.17 | 0.17 | 0.25 |  |  |  |  |  |
| 13 | 6876434 | 6877337 | N:30 | N:34.74 | N:37.16 | N:38.19 | N:29.32 | N:34.29 | N:35.79 | N:34.74 | N:33.35 | N:30.69 | N:36.92 | N:35.78 | Y:8.86 | N:13.86 | N:17.1 | Y:17.89 | Y:9.75 | N:18.55 | Y:14.99 | Y:13.28 | N:67.06 | N:16.21 | N:24.06 | 5 | 0.22 | 0 | 0 | 0.63 |  |  |  |  |  |
| 13 | 10909806 | 10910958 | N:22.13 | N:23.47 | N:24.69 | N:25.61 | N:20.71 | N:20.64 | N:22.73 | N:23.74 | N:20.68 | N:18.59 | Y:26.15 | N:24.94 | N:8.26 | N:13.86 | N:15.49 | N:10.29 | Y:10.03 | Y:21.65 | N:10.75 | N:14.71 | Y:61.19 | N:27.17 | N:29.76 | 4 | 0.17 | 0 | 0.17 | 0.25 |  |  |  |  |  |
| AADN03017712.1 | 157 | 1286 | N:10701.64 | N:10739.58 | N:12515.23 | Y:10694.45 | N:10164.47 | N:9811.11 | N:7791.51 | N:7647.74 | N:7814.2 | N:7123.75 | N:6808.31 | N:9549.05 | NA | NA | NA | NA | NA | NA | NA | NA | N:19183.13 | N:2592.41 | N:3563.07 | 1 | 0.07 | 0.17 | 0 | 0 |  |  |  |  |  |
| 17 | 180636 | 181570 | N:27.13 | N:26.94 | N:27.36 | N:24.32 | Y:25.86 | Y:30.01 | Y:27.86 | N:26.66 | Y:29.25 | Y:24.64 | N:24.61 | Y:27.7 | Y:13.16 | Y:23.09 | Y:23.04 | N:29.96 | Y:11.18 | Y:23.17 | Y:19.64 | Y:20.27 | N:62.1 | Y:39.49 | Y:40.51 | 15 | 0.65 | 0.33 | 0.67 | 0.88 |  |  |  |  |  |
| 19 | 2694054 | 2694923 | N:36.05 | N:32.12 | N:30.71 | N:32.29 | Y:32.4 | N:32.49 | N:26.7 | N:33.09 | N:29.76 | N:31.97 | N:31.89 | N:30.91 | N:9.76 | N:15.05 | N:24.38 | N:19.45 | N:11.78 | N:21.22 | N:15.37 | N:14.24 | N:57.48 | N:30.71 | N:27.21 | 1 | 0.04 | 0.17 | 0 | 0 |  |  |  |  |  |
| AADN03018735.1 | 281 | 1293 | N:14.16 | N:5.05 | N:2.97 | N:4.87 | N:24.17 | N:28.21 | N:4.1 | N:14.45 | N:17.42 | N:14.34 | N:17.63 | N:16.62 | Y:30.65 | N:18.99 | N:9.59 | N:18.22 | N:11.11 | N:11.1 | N:6.26 | N:15.61 | N:32.62 | N:35.34 | N:45.88 | 1 | 0.04 | 0 | 0 | 0.13 |  |  |  |  |  |
| 14 | 236512 | 236813 | N:28.97 | N:29.03 | N:30.05 | N:26.41 | N:31.94 | N:25.77 | N:30.73 | N:30.4 | N:29.23 | N:22.46 | N:30.68 | N:34.66 | N:12.62 | N:21.96 | N:16.79 | N:17.27 | N:14.64 | N:29.55 | N:13.85 | Y:22.62 | N:55.26 | N:28.04 | N:26.64 | 1 | 0.04 | 0 | 0 | 0.13 |  |  |  |  |  |
| 14 | 7965871 | 7966753 | N:32.18 | N:27.11 | N:34.04 | N:34.61 | N:31.19 | N:30.1 | N:30.58 | N:32.92 | Y:26.46 | Y:26.43 | Y:26.21 | N:30.21 | N:9.49 | N:15.5 | N:18.55 | N:14.48 | N:8.37 | N:16.72 | N:12.67 | N:16.45 | N:58.3 | Y:26.09 | N:25.09 | 4 | 0.17 | 0 | 0.5 | 0 |  |  |  |  |  |
| AADN03024906.1 | 3470 | 4351 | N:4.12 | N:9.21 | N:5.59 | N:6.6 | N:7.9 | N:6.59 | N:6.56 | N:6.75 | N:4.63 | N:3.08 | N:3.89 | Y:20.54 | Y:7.79 | N:8.72 | Y:12.74 | Y:14.02 | NA | Y:10.21 | N:11.44 | Y:11.21 | N:36.35 | N:2.21 | Y:23.22 | 7 | 0.32 | 0 | 0.17 | 0.71 |  |  |  |  |  |
| JH375212.1 | 37241 | 37702 | NA | NA | NA | NA | NA | NA | NA | NA | NA | NA | NA | Y:12.13 | N:5.25 | Y:6.01 | Y:14.55 | Y:11.44 | NA | N:9.71 | Y:6.9 | N:9 | Y:29.23 | NA | N:19.09 | 6 | 0.6 | 0 | 1 | 0.57 |  |  |  |  |  |
| JH375231.1 | 4846 | 8915 | NA | NA | NA | NA | NA | NA | N:1.96 | NA | NA | NA | NA | Y:12.07 | N:5.54 | Y:9.94 | Y:10.63 | Y:9.03 | NA | Y:10.77 | Y:9.6 | Y:7.27 | Y:25 | NA | Y:22.53 | 9 | 0.82 | 0 | 0.5 | 0.86 |  |  |  |  |  |
| JH375237.1 | 49865 | 50701 | NA | NA | NA | NA | NA | NA | NA | NA | NA | NA | NA | Y:12.76 | N:5.91 | N:11.59 | N:10.91 | N:11.86 | NA | N:10.51 | N:10.31 | N:9.07 | Y:26.06 | NA | Y:22.89 | 3 | 0.3 | 0 | 1 | 0 |  |  |  |  |  |
| JH376310.1 | 6513 | 7054 | NA | NA | NA | NA | NA | NA | NA | NA | NA | NA | NA | Y:12.44 | Y:3.92 | Y:5.67 | Y:14.09 | Y:15.87 | NA | Y:5.99 | Y:9.07 | Y:5.99 | Y:30.63 | NA | N:10.71 | 9 | 0.9 | 0 | 1 | 1 |  |  |  |  |  |
| AADN03016632.1 | 765 | 923 | N:20.7 | N:10.39 | N:8.92 | N:11.19 | N:29.23 | N:20.42 | N:11.55 | N:19.31 | N:8.68 | N:7.44 | N:22.54 | N:21.33 | Y:32.26 | N:15.71 | N:5.57 | N:10.6 | Y:10.76 | N:3.37 | N:1.26 | Y:17.35 | N:33.65 | N:8.79 | N:86.87 | 3 | 0.13 | 0 | 0 | 0.38 |  |  |  |  |  |
| AADN03019391.1 | 477 | 583 | NA | NA | NA | N:2.59 | N:1.43 | NA | NA | NA | NA | NA | NA | N:1.93 | Y:11.09 | N:1.25 | N:1.72 | NA | NA | N:8.08 | NA | N:15.95 | N:29.85 | N:21.14 | N:1.31 | 1 | 0.09 | 0 | 0 | 0.2 |  |  |  |  |  |
| AADN03016003.1 | 257 | 448 | N:804.04 | N:895.22 | N:866.44 | N:753.36 | N:708.25 | N:930.69 | N:652.17 | N:701.08 | N:751.85 | N:699.99 | N:676.23 | N:858.21 | N:278.74 | N:359.99 | N:464.19 | Y:713.43 | N:295.28 | N:498.06 | N:387.71 | N:372.42 | N:2614.92 | N:1103.23 | N:1683.5 | 1 | 0.04 | 0 | 0 | 0.13 |  |  |  |  |  |
| AADN03024630.1 | 317 | 887 | N:893.03 | N:939.16 | N:947.48 | N:753.75 | N:796.99 | N:1021.72 | N:759.82 | N:810.68 | N:881.87 | N:770.38 | N:752.38 | N:865.29 | N:310.47 | N:429.15 | N:647.72 | Y:NA | N:355.08 | N:848.4 | N:480.22 | N:476.23 | N:2401.09 | N:1680.96 | N:2531.17 | 1 | 0.04 | 0 | 0 | 0.13 |  |  |  |  |  |
| AADN03025776.1 | 820 | 1020 | N:18.74 | N:17.16 | N:21.81 | N:24.25 | N:18.31 | N:17.65 | N:22.71 | N:14.84 | N:15.42 | N:11.32 | N:21.32 | N:21.76 | N:16.41 | N:16.73 | N:12.65 | N:11.86 | N:9.07 | N:13.84 | Y:23.51 | N:13.67 | N:49.2 | N:39.73 | N:50.23 | 1 | 0.04 | 0 | 0 | 0.13 |  |  |  |  |  |
| AADN03018589.1 | 484 | 802 | N:39.06 | N:9.38 | N:20.99 | N:7.53 | N:12.46 | N:27.26 | N:19.94 | N:24.34 | N:26.13 | N:20.03 | N:21.44 | N:25.13 | N:1.83 | NA | N:2.71 | N:4.63 | N:3.96 | N:10.56 | Y:10.92 | N:8.42 | N:55.42 | Y:26.92 | N:7.29 | 2 | 0.09 | 0 | 0 | 0.14 |  |  |  |  |  |
|  | **Total** | **Intervals** | 66 | 80 | 73 | 70 | 70 | 78 | 62 | 68 | 62 | 62 | 62 | 81 | 57 | 65 | 61 | 61 | 50 | 72 | 70 | 69 | 56 | 67 | 64 |  |  |  |  |  |  |  |  |  |  |
|  |  |  |  |  |  |  |  |  |  |  |  |  |  |  |  |  |  |  |  |  |  |  |  |  |  |  |  |  |  |  |  |  |  |  |  |
|  |  |  |  |  |  |  |  |  |  |  |  |  |  |  |  |  |  |  |  |  |  |  |  |  |  |  |  |  |  |  |  |  |  |  |  |
| S3e. Summary of intervals per chromosome per bird/line | | | | | | | | | | | | | | | | | | | | | | | | | |  |  |  |  |  |  |  |  |  |  |
|  |  |  |  |  |  |  |  |  |  |  |  |  |  |  |  |  |  |  |  |  |  |  |  |  |  |  |  |  |  |  |  |  |  |  |  |
| **Chr** | | | **HA1A22A** | **HA1B25B** | **HA2A10B** | **HA2A25B** | **HB1A16A** | **HB1B21B** | **JA1A17A** | **JA2A10B** | **JB2A04B.1** | **JB2A04B.2** | **JB1A25B** | **JB1B16A** | **Line15** | **Line6** | **Line7** | **LineC** | **LineN** | **LineP** | **LineZero** | **Wellcome** | **RJF** | **Silkie** | **Taiwanese** |  |  |  |  |  |  |  |  |  |  |
| **1** | | | 13 | 24 | 19 | 17 | 16 | 18 | 12 | 19 | 20 | 17 | 21 | 20 | 12 | 17 | 16 | 18 | 9 | 23 | 26 | 16 | 12 | 16 | 15 |  |  |  |  |  |  |  |  |  |  |
| **2** | | | 10 | 10 | 10 | 13 | 13 | 13 | 10 | 9 | 7 | 7 | 10 | 7 | 10 | 13 | 8 | 8 | 11 | 11 | 12 | 13 | 11 | 5 | 8 |  |  |  |  |  |  |  |  |  |  |
| **3** | | | 12 | 9 | 11 | 10 | 12 | 13 | 11 | 10 | 8 | 8 | 12 | 8 | 6 | 8 | 8 | 8 | 6 | 9 | 6 | 12 | 7 | 10 | 8 |  |  |  |  |  |  |  |  |  |  |
| **4** | | | 6 | 8 | 5 | 7 | 6 | 10 | 6 | 7 | 4 | 7 | 8 | 4 | 6 | 5 | 7 | 5 | 6 | 6 | 4 | 7 | 3 | 6 | 6 |  |  |  |  |  |  |  |  |  |  |
| **5** | | | 7 | 7 | 8 | 7 | 4 | 7 | 3 | 4 | 5 | 5 | 6 | 5 | 5 | 5 | 5 | 3 | 4 | 6 | 7 | 4 | 7 | 7 | 6 |  |  |  |  |  |  |  |  |  |  |
| **6** | | | 2 | 3 | 3 | 2 | 2 | 2 | 2 | 3 | 2 | 4 | 2 | 2 | 2 | 3 | 3 | 3 | 2 | 2 | 2 | 2 | 2 | 2 | 3 |  |  |  |  |  |  |  |  |  |  |
| **7** | | | 2 | 3 | 2 | 1 | 3 | 2 | 3 | 4 | 3 | 3 | 3 | 3 | 1 | 1 | 1 | 1 | 0 | 1 | 1 | 1 | 0 | 2 | 1 |  |  |  |  |  |  |  |  |  |  |
| **8** | | | 1 | 0 | 1 | 1 | 1 | 1 | 2 | 3 | 2 | 2 | 2 | 2 | 1 | 1 | 1 | 0 | 1 | 1 | 0 | 0 | 1 | 1 | 0 |  |  |  |  |  |  |  |  |  |  |
| **9** | | | 1 | 2 | 2 | 0 | 2 | 2 | 0 | 0 | 1 | 1 | 1 | 1 | 0 | 0 | 0 | 0 | 0 | 1 | 0 | 0 | 0 | 0 | 0 |  |  |  |  |  |  |  |  |  |  |
| **10** | | | 1 | 1 | 1 | 1 | 0 | 0 | 0 | 0 | 0 | 0 | 0 | 0 | 0 | 0 | 0 | 0 | 0 | 0 | 0 | 0 | 0 | 1 | 1 |  |  |  |  |  |  |  |  |  |  |
| **11** | | | 0 | 1 | 0 | 1 | 0 | 0 | 1 | 0 | 0 | 0 | 0 | 0 | 0 | 0 | 1 | 0 | 0 | 0 | 0 | 0 | 0 | 1 | 1 |  |  |  |  |  |  |  |  |  |  |
| **12** | | | 1 | 1 | 1 | 1 | 1 | 1 | 2 | 2 | 1 | 0 | 2 | 1 | 2 | 2 | 2 | 2 | 0 | 0 | 2 | 2 | 2 | 4 | 2 |  |  |  |  |  |  |  |  |  |  |
| **13** | | | 0 | 0 | 0 | 1 | 1 | 2 | 1 | 0 | 1 | 1 | 0 | 1 | 1 | 0 | 0 | 1 | 3 | 2 | 1 | 2 | 1 | 0 | 1 |  |  |  |  |  |  |  |  |  |  |
| **14** | | | 0 | 0 | 0 | 0 | 0 | 0 | 0 | 0 | 1 | 1 | 0 | 1 | 0 | 0 | 0 | 0 | 0 | 0 | 0 | 1 | 0 | 1 | 0 |  |  |  |  |  |  |  |  |  |  |
| **15** | | | 0 | 1 | 0 | 1 | 0 | 0 | 1 | 0 | 0 | 0 | 0 | 0 | 0 | 0 | 0 | 0 | 0 | 0 | 0 | 0 | 0 | 0 | 0 |  |  |  |  |  |  |  |  |  |  |
| **16** | | | 0 | 0 | 0 | 0 | 0 | 0 | 0 | 0 | 0 | 0 | 0 | 0 | 0 | 0 | 0 | 0 | 0 | 0 | 0 | 0 | 0 | 0 | 0 |  |  |  |  |  |  |  |  |  |  |
| **17** | | | 0 | 0 | 0 | 0 | 1 | 1 | 1 | 0 | 1 | 0 | 1 | 1 | 1 | 1 | 1 | 0 | 1 | 1 | 1 | 1 | 0 | 1 | 1 |  |  |  |  |  |  |  |  |  |  |
| **18** | | | 0 | 0 | 0 | 0 | 0 | 0 | 0 | 0 | 0 | 0 | 0 | 0 | 0 | 0 | 0 | 0 | 0 | 0 | 0 | 0 | 0 | 0 | 0 |  |  |  |  |  |  |  |  |  |  |
| **19** | | | 0 | 0 | 0 | 0 | 1 | 0 | 0 | 0 | 0 | 0 | 0 | 0 | 0 | 0 | 0 | 0 | 0 | 0 | 0 | 0 | 0 | 0 | 0 |  |  |  |  |  |  |  |  |  |  |
| **20** | | | 2 | 1 | 1 | 0 | 0 | 0 | 1 | 0 | 0 | 1 | 2 | 0 | 1 | 2 | 0 | 1 | 1 | 1 | 1 | 1 | 0 | 0 | 1 |  |  |  |  |  |  |  |  |  |  |
| **21** | | | 0 | 0 | 0 | 0 | 0 | 0 | 0 | 0 | 0 | 0 | 0 | 0 | 0 | 0 | 0 | 0 | 0 | 0 | 0 | 0 | 0 | 0 | 0 |  |  |  |  |  |  |  |  |  |  |
| **22** | | | 0 | 0 | 0 | 0 | 0 | 0 | 0 | 0 | 0 | 0 | 0 | 0 | 0 | 0 | 0 | 0 | 0 | 0 | 0 | 0 | 0 | 0 | 0 |  |  |  |  |  |  |  |  |  |  |
| **23** | | | 0 | 0 | 0 | 0 | 0 | 0 | 0 | 0 | 0 | 0 | 0 | 0 | 0 | 0 | 0 | 0 | 0 | 0 | 0 | 0 | 0 | 0 | 0 |  |  |  |  |  |  |  |  |  |  |
| **24** | | | 0 | 0 | 1 | 0 | 0 | 0 | 0 | 0 | 0 | 0 | 0 | 0 | 0 | 0 | 0 | 0 | 0 | 0 | 0 | 0 | 0 | 0 | 0 |  |  |  |  |  |  |  |  |  |  |
| **25** | | | 0 | 0 | 0 | 0 | 0 | 0 | 0 | 0 | 0 | 0 | 0 | 0 | 0 | 0 | 0 | 0 | 0 | 0 | 0 | 0 | 0 | 0 | 0 |  |  |  |  |  |  |  |  |  |  |
| **26** | | | 0 | 0 | 0 | 0 | 0 | 0 | 0 | 0 | 0 | 0 | 0 | 0 | 0 | 0 | 0 | 0 | 0 | 0 | 0 | 0 | 0 | 0 | 0 |  |  |  |  |  |  |  |  |  |  |
| **27** | | | 0 | 0 | 0 | 0 | 0 | 0 | 0 | 0 | 0 | 0 | 0 | 0 | 0 | 0 | 0 | 0 | 0 | 0 | 0 | 0 | 0 | 0 | 0 |  |  |  |  |  |  |  |  |  |  |
| **28** | | | 0 | 0 | 0 | 0 | 0 | 0 | 0 | 0 | 0 | 0 | 0 | 0 | 0 | 0 | 0 | 0 | 0 | 0 | 0 | 0 | 0 | 0 | 0 |  |  |  |  |  |  |  |  |  |  |
| **Z** | | | 5 | 7 | 8 | 6 | 6 | 6 | 6 | 5 | 6 | 5 | 4 | 6 | 4 | 4 | 4 | 5 | 5 | 5 | 2 | 3 | 6 | 8 | 7 |  |  |  |  |  |  |  |  |  |  |
| **Unplaced ['A...']** | | | 0 | 0 | 0 | 1 | 0 | 0 | 0 | 0 | 0 | 0 | 1 | 0 | 4 | 0 | 1 | 3 | 1 | 1 | 2 | 2 | 0 | 1 | 1 |  |  |  |  |  |  |  |  |  |  |
| **Unplaced ['J...']** | | | 3 | 2 | 0 | 0 | 1 | 0 | 0 | 2 | 0 | 0 | 6 | 0 | 1 | 3 | 3 | 3 | 0 | 2 | 3 | 2 | 4 | 1 | 2 |  |  |  |  |  |  |  |  |  |  |
| **Total** | | | 66 | 80 | 73 | 70 | 70 | 78 | 62 | 68 | 62 | 62 | 81 | 62 | 57 | 65 | 61 | 61 | 50 | 72 | 70 | 69 | 56 | 67 | 64 |  |  |  |  |  |  |  |  |  |  |
| **Ratio (intervals/integrations)** | | | 0.86 | 0.89 | 0.87 | 0.89 | 0.86 | 0.88 | 0.87 | 0.88 | 0.86 | 0.86 | 1.13 | 0.66 | 0.97 | 0.84 | 0.91 | 0.91 | 0.91 | 0.91 | 0.83 | 0.92 | 0.88 | 0.89 | 0.91 |  |  |  |  |  |  |  |  |  |  |
| **Average ratio** | | | 0.88 | | | | | | | | | | | | 0.90 | | | | | | | | 0.89 | | |  |  |  |  |  |  |  |  |  |  |
|  |  |  |  |  |  |  |  |  |  |  |  |  |  |  |  |  |  |  |  |  |  |  |  |  |  |  |  |  |  |  |  |  |  |  |  |
|  |  |  |  |  |  |  |  |  |  |  |  |  |  |  |  |  |  |  |  |  |  |  |  |  |  |  |  |  |  |  |  |  |  |  |  |
| S3f. Summary of integrations per chromosome per bird/line | | | | | | | | | | | | | | | | | | | | | | | | | |  |  |  |  |  |  |  |  |  |  |
|  |  |  |  |  |  |  |  |  |  |  |  |  |  |  |  |  |  |  |  |  |  |  |  |  |  |  |  |  |  |  |  |  |  |  |  |
| **Chr** | | | **HA1A22A** | **HA1B25B** | **HA2A10B** | **HA2A25B** | **HB1A16A** | **HB1B21B** | **JA1A17A** | **JA2A10B** | **JB2A04B.1** | **JB2A04B.2** | **JB1A25B** | **JB1B16A** | **Line15** | **Line6** | **Line7** | **LineC** | **LineN** | **LineP** | **LineZero** | **Wellcome** | **RJF** | **Silkie** | **Taiwanese** |  |  |  |  |  |  |  |  |  |  |
| **1** | | | 15 | 28 | 22 | 19 | 19 | 20 | 15 | 22 | 24 | 24 | 19 | 26 | 13 | 20 | 17 | 20 | 10 | 26 | 34 | 18 | 14 | 18 | 17 |  |  |  |  |  |  |  |  |  |  |
| **2** | | | 11 | 11 | 11 | 15 | 14 | 14 | 11 | 11 | 8 | 8 | 8 | 12 | 10 | 15 | 9 | 9 | 12 | 12 | 13 | 14 | 12 | 6 | 8 |  |  |  |  |  |  |  |  |  |  |
| **3** | | | 15 | 11 | 13 | 11 | 15 | 15 | 12 | 11 | 9 | 9 | 9 | 14 | 6 | 10 | 9 | 9 | 6 | 9 | 7 | 13 | 8 | 12 | 8 |  |  |  |  |  |  |  |  |  |  |
| **4** | | | 7 | 9 | 6 | 8 | 6 | 11 | 7 | 8 | 5 | 5 | 8 | 9 | 6 | 5 | 7 | 5 | 6 | 6 | 5 | 7 | 3 | 6 | 6 |  |  |  |  |  |  |  |  |  |  |
| **5** | | | 7 | 7 | 8 | 7 | 4 | 7 | 3 | 4 | 5 | 5 | 5 | 6 | 5 | 5 | 5 | 3 | 4 | 6 | 7 | 4 | 7 | 7 | 6 |  |  |  |  |  |  |  |  |  |  |
| **6** | | | 3 | 4 | 4 | 3 | 3 | 3 | 3 | 4 | 3 | 3 | 5 | 3 | 3 | 4 | 4 | 4 | 3 | 4 | 4 | 3 | 3 | 3 | 4 |  |  |  |  |  |  |  |  |  |  |
| **7** | | | 2 | 3 | 2 | 1 | 3 | 2 | 3 | 4 | 3 | 3 | 3 | 3 | 1 | 1 | 1 | 1 | 0 | 1 | 1 | 1 | 0 | 2 | 1 |  |  |  |  |  |  |  |  |  |  |
| **8** | | | 1 | 0 | 1 | 1 | 1 | 1 | 2 | 3 | 2 | 2 | 2 | 2 | 1 | 1 | 1 | 0 | 1 | 1 | 0 | 0 | 1 | 1 | 0 |  |  |  |  |  |  |  |  |  |  |
| **9** | | | 1 | 2 | 2 | 0 | 2 | 2 | 0 | 0 | 1 | 1 | 1 | 1 | 0 | 0 | 0 | 0 | 0 | 1 | 0 | 0 | 0 | 0 | 0 |  |  |  |  |  |  |  |  |  |  |
| **10** | | | 1 | 1 | 1 | 1 | 0 | 0 | 0 | 0 | 0 | 0 | 0 | 0 | 0 | 0 | 0 | 0 | 0 | 0 | 0 | 0 | 0 | 1 | 1 |  |  |  |  |  |  |  |  |  |  |
| **11** | | | 0 | 1 | 0 | 1 | 0 | 0 | 1 | 0 | 0 | 0 | 0 | 0 | 0 | 0 | 1 | 0 | 0 | 0 | 0 | 0 | 0 | 1 | 1 |  |  |  |  |  |  |  |  |  |  |
| **12** | | | 1 | 1 | 1 | 1 | 1 | 1 | 2 | 2 | 1 | 1 | 0 | 2 | 2 | 2 | 2 | 2 | 0 | 0 | 2 | 2 | 2 | 4 | 2 |  |  |  |  |  |  |  |  |  |  |
| **13** | | | 0 | 0 | 0 | 1 | 1 | 2 | 1 | 0 | 1 | 1 | 1 | 0 | 1 | 0 | 0 | 1 | 3 | 2 | 1 | 2 | 1 | 0 | 1 |  |  |  |  |  |  |  |  |  |  |
| **14** | | | 0 | 0 | 0 | 0 | 0 | 0 | 0 | 0 | 1 | 1 | 1 | 0 | 0 | 0 | 0 | 0 | 0 | 0 | 0 | 1 | 0 | 1 | 0 |  |  |  |  |  |  |  |  |  |  |
| **15** | | | 0 | 1 | 0 | 1 | 0 | 0 | 1 | 0 | 0 | 0 | 0 | 0 | 0 | 0 | 0 | 0 | 0 | 0 | 0 | 0 | 0 | 0 | 0 |  |  |  |  |  |  |  |  |  |  |
| **16** | | | 0 | 0 | 0 | 0 | 0 | 0 | 0 | 0 | 0 | 0 | 0 | 0 | 0 | 0 | 0 | 0 | 0 | 0 | 0 | 0 | 0 | 0 | 0 |  |  |  |  |  |  |  |  |  |  |
| **17** | | | 0 | 0 | 0 | 0 | 1 | 1 | 1 | 0 | 1 | 1 | 0 | 1 | 1 | 1 | 1 | 0 | 1 | 1 | 1 | 1 | 0 | 1 | 1 |  |  |  |  |  |  |  |  |  |  |
| **18** | | | 0 | 0 | 0 | 0 | 0 | 0 | 0 | 0 | 0 | 0 | 0 | 0 | 0 | 0 | 0 | 0 | 0 | 0 | 0 | 0 | 0 | 0 | 0 |  |  |  |  |  |  |  |  |  |  |
| **19** | | | 0 | 0 | 0 | 0 | 1 | 0 | 0 | 0 | 0 | 0 | 0 | 0 | 0 | 0 | 0 | 0 | 0 | 0 | 0 | 0 | 0 | 0 | 0 |  |  |  |  |  |  |  |  |  |  |
| **20** | | | 2 | 1 | 1 | 0 | 0 | 0 | 1 | 0 | 0 | 0 | 1 | 2 | 1 | 2 | 0 | 1 | 1 | 1 | 1 | 1 | 0 | 0 | 1 |  |  |  |  |  |  |  |  |  |  |
| **21** | | | 0 | 0 | 0 | 0 | 0 | 0 | 0 | 0 | 0 | 0 | 0 | 0 | 0 | 0 | 0 | 0 | 0 | 0 | 0 | 0 | 0 | 0 | 0 |  |  |  |  |  |  |  |  |  |  |
| **22** | | | 0 | 0 | 0 | 0 | 0 | 0 | 0 | 0 | 0 | 0 | 0 | 0 | 0 | 0 | 0 | 0 | 0 | 0 | 0 | 0 | 0 | 0 | 0 |  |  |  |  |  |  |  |  |  |  |
| **23** | | | 0 | 0 | 0 | 0 | 0 | 0 | 0 | 0 | 0 | 0 | 0 | 0 | 0 | 0 | 0 | 0 | 0 | 0 | 0 | 0 | 0 | 0 | 0 |  |  |  |  |  |  |  |  |  |  |
| **24** | | | 0 | 0 | 1 | 0 | 0 | 0 | 0 | 0 | 0 | 0 | 0 | 0 | 0 | 0 | 0 | 0 | 0 | 0 | 0 | 0 | 0 | 0 | 0 |  |  |  |  |  |  |  |  |  |  |
| **25** | | | 0 | 0 | 0 | 0 | 0 | 0 | 0 | 0 | 0 | 0 | 0 | 0 | 0 | 0 | 0 | 0 | 0 | 0 | 0 | 0 | 0 | 0 | 0 |  |  |  |  |  |  |  |  |  |  |
| **26** | | | 0 | 0 | 0 | 0 | 0 | 0 | 0 | 0 | 0 | 0 | 0 | 0 | 0 | 0 | 0 | 0 | 0 | 0 | 0 | 0 | 0 | 0 | 0 |  |  |  |  |  |  |  |  |  |  |
| **27** | | | 0 | 0 | 0 | 0 | 0 | 0 | 0 | 0 | 0 | 0 | 0 | 0 | 0 | 0 | 0 | 0 | 0 | 0 | 0 | 0 | 0 | 0 | 0 |  |  |  |  |  |  |  |  |  |  |
| **28** | | | 0 | 0 | 0 | 0 | 0 | 0 | 0 | 0 | 0 | 0 | 0 | 0 | 0 | 0 | 0 | 0 | 0 | 0 | 0 | 0 | 0 | 0 | 0 |  |  |  |  |  |  |  |  |  |  |
| **Z** | | | 7 | 9 | 10 | 8 | 7 | 8 | 7 | 7 | 8 | 8 | 7 | 6 | 4 | 5 | 5 | 6 | 6 | 6 | 3 | 4 | 7 | 9 | 8 |  |  |  |  |  |  |  |  |  |  |
| **Unplaced ['A...']** | | | 1 | 0 | 1 | 1 | 1 | 2 | 1 | 0 | 0 | 0 | 2 | 1 | 4 | 2 | 1 | 3 | 2 | 1 | 2 | 2 | 1 | 2 | 2 |  |  |  |  |  |  |  |  |  |  |
| **Unplaced ['J...']** | | | 3 | 1 | 0 | 0 | 2 | 0 | 0 | 1 | 0 | 0 | 0 | 6 | 1 | 4 | 4 | 3 | 0 | 2 | 3 | 2 | 5 | 1 | 3 |  |  |  |  |  |  |  |  |  |  |
| **Total** | | | 77 | 90 | 84 | 79 | 81 | 89 | 71 | 77 | 72 | 72 | 72 | 94 | 59 | 77 | 67 | 67 | 55 | 79 | 84 | 75 | 64 | 75 | 70 |  |  |  |  |  |  |  |  |  |  |
|  |  |  |  |  |  |  |  |  |  |  |  |  |  |  |  |  |  |  |  |  |  |  |  |  |  |  |  |  |  |  |  |  |  |  |  |
|  |  |  |  |  |  |  |  |  |  |  |  |  |  |  |  |  |  |  |  |  |  |  |  |  |  |  |  |  |  |  |  |  |  |  |  |
| S3g. Summary of coverage across integration sites | | | | | | | | | | | | | | | | | | | | | | | | | | |  |  |  |  |  |  |  |  |  |
|  |  |  |  |  |  |  |  |  |  |  |  |  |  |  |  |  |  |  |  |  |  |  |  |  |  |  |  |  |  |  |  |  |  |  |  |
|  | | | **HA1A22A** | **HA1B25B** | **HA2A10B** | **HA2A25B** | **HB1A16A** | **HB1B21B** | **JA1A17A** | **JA2A10B** | **JB2A04B.1** | **JB2A04B.2** | **JB1A25B** | **JB1B16A** | **Line15** | **Line6** | **Line7** | **LineC** | **LineN** | **LineP** | **LineZero** | **Wellcome** | **RJF** | **Silkie** | **Taiwanese** | **Average** |  |  |  |  |  |  |  |  |  |
| **Galgal4 mean depth\*** | | | 29 | 31 | 32 | 31 | 29 | 30 | 29 | 31 | 30 | 24 | 30 | 28 | 9 | 16 | 18 | 16 | 11 | 20 | 16 | 16 | 52 | 26 | 29 | 25 |  |  |  |  |  |  |  |  |  |
| **Mean across type** | | | 29.50 | | | | | | | | | | | | 15.25 | | | | | | | | 27.50 | | |  |  |  |  |  |  |  |  |  |  |
| **St.dev. Across type** | | | 2.07 | | | | | | | | | | | | 3.58 | | | | | | | | 2.12 | | |  |  |  |  |  |  |  |  |  |  |
| **EAV-HP LTR mean depth** | | | 13 | 12 | 14 | 14 | 12 | 13 | 12 | 13 | 13 | 10 | 13 | 12 | 4 | 8 | 10 | 8 | 4 | 10 | 8 | 7 | 11 | 18 | 14 | 11.00 |  |  |  |  |  |  |  |  |  |
| **Mean across type** | | | 12.58 | | | | | | | | | | | | 7.38 | | | | | | | | 16 | | |  |  |  |  |  |  |  |  |  |  |
| **St.dev. Across type** | | | 1.08 | | | | | | | | | | | | 2.33 | | | | | | | | 2.83 | | |  |  |  |  |  |  |  |  |  |  |
| **EAV-HP LTR U3 (100% coverage)** | | | 21 | 29 | 33 | 24 | 23 | 31 | 25 | 25 | 24 | 22 | 21 | 25 | 15 | 24 | 19 | 27 | 15 | 26 | 17 | 20 | 9 | 31 | 30 | 23.30 |  |  |  |  |  |  |  |  |  |
| **EAV-HP LTR R (100% coverage)** | | | 70 | 76 | 77 | 73 | 73 | 80 | 65 | 69 | 67 | 63 | 64 | 88 | 48 | 68 | 63 | 62 | 46 | 70 | 78 | 64 | 40 | 70 | 59 | 66.65 |  |  |  |  |  |  |  |  |  |
| **EAV-HP LTR U5 (100% coverage)** | | | 26 | 42 | 34 | 26 | 34 | 34 | 21 | 26 | 23 | 21 | 27 | 30 | 10 | 23 | 25 | 16 | 12 | 19 | 26 | 19 | 2 | 39 | 26 | 24.39 |  |  |  |  |  |  |  |  |  |
| **EAV-HP LTR (100% coverage)** | | | 15 | 25 | 24 | 17 | 18 | 21 | 15 | 17 | 16 | 11 | 17 | 15 | 6 | 15 | 14 | 12 | 7 | 14 | 13 | 12 | 6 | 20 | 24 | 15.39 |  |  |  |  |  |  |  |  |  |
| **Fraction sites with 100% U3** | | | 0.27 | 0.32 | 0.39 | 0.30 | 0.28 | 0.35 | 0.35 | 0.32 | 0.33 | 0.31 | 0.29 | 0.27 | 0.25 | 0.31 | 0.28 | 0.40 | 0.27 | 0.33 | 0.20 | 0.27 | 0.14 | 0.41 | 0.43 | 0.31 |  |  |  |  |  |  |  |  |  |
| **Fraction sites with 100% R** | | | 0.91 | 0.84 | 0.92 | 0.92 | 0.90 | 0.90 | 0.92 | 0.90 | 0.93 | 0.88 | 0.89 | 0.94 | 0.81 | 0.88 | 0.94 | 0.93 | 0.84 | 0.89 | 0.93 | 0.85 | 0.63 | 0.93 | 0.84 | 0.88 |  |  |  |  |  |  |  |  |  |
| **Fraction sites with 100% U5** | | | 0.34 | 0.47 | 0.40 | 0.33 | 0.42 | 0.38 | 0.30 | 0.34 | 0.32 | 0.29 | 0.38 | 0.32 | 0.17 | 0.30 | 0.37 | 0.24 | 0.22 | 0.24 | 0.31 | 0.25 | 0.03 | 0.52 | 0.37 | 0.32 |  |  |  |  |  |  |  |  |  |
| **Fraction sites with 100% LTR** | | | 0.19 | 0.28 | 0.29 | 0.22 | 0.22 | 0.24 | 0.21 | 0.22 | 0.22 | 0.15 | 0.24 | 0.16 | 0.10 | 0.19 | 0.21 | 0.18 | 0.13 | 0.18 | 0.15 | 0.16 | 0.09 | 0.27 | 0.34 | 0.20 |  |  |  |  |  |  |  |  |  |
|  |  |  |  |  |  |  |  |  |  |  |  |  |  |  |  |  |  |  |  |  |  |  |  |  |  |  |  |  |  |  |  |  |  |  |  |
| \* Excludes sites with excess coverage ( > 2\* unadjusted mean) | | | | | | | | | | | | | | | | | | | | | | | | | | |  |  |  |  |  |  |  |  |  |

  


---

  


# Supplementary Table 4: *S4*

| S4. Summary of distribution of intervals | | | | | | | | | | | | | | | | | | | | | | | | |
|  |  |  |  |  |  |  |  |  |  |  |  |  |  |  |  |  |  |  |  |  |  |  |  |  |
| **Fraction of intervals along..** | **HA1A22A** | **HA1B25B** | **HA2A10B** | **HA2A25B** | **HB1A16A** | **HB1B21B** | **JA1A17A** | **JA2A10B** | **JB2A04B.1** | **JB2A04B.2** | **JB1A25B** | **JB1B16A** | **Line15** | **Line6** | **Line7** | **LineC** | **LineN** | **LineP** | **LineZero** | **Wellcome** | **RJF** | **Silkie** | **Taiwanese** | **Average** |
| Macrochromosomes | 0.73 | 0.73 | 0.73 | 0.77 | 0.73 | 0.78 | 0.68 | 0.72 | 0.71 | 0.71 | 0.70 | 0.71 | 0.68 | 0.74 | 0.72 | 0.69 | 0.72 | 0.76 | 0.79 | 0.75 | 0.71 | 0.66 | 0.67 | 0.72 |
| Intermediate-chromosomes | 0.11 | 0.11 | 0.12 | 0.07 | 0.11 | 0.09 | 0.11 | 0.15 | 0.13 | 0.16 | 0.10 | 0.13 | 0.07 | 0.08 | 0.08 | 0.07 | 0.06 | 0.07 | 0.04 | 0.04 | 0.05 | 0.09 | 0.08 | 0.09 |
| Microchromosomes | 0.05 | 0.05 | 0.04 | 0.06 | 0.06 | 0.05 | 0.11 | 0.03 | 0.06 | 0.05 | 0.06 | 0.06 | 0.09 | 0.08 | 0.07 | 0.07 | 0.10 | 0.06 | 0.07 | 0.10 | 0.05 | 0.10 | 0.09 | 0.07 |
| Z chromosome | 0.08 | 0.09 | 0.11 | 0.09 | 0.09 | 0.08 | 0.10 | 0.07 | 0.10 | 0.08 | 0.05 | 0.10 | 0.07 | 0.06 | 0.07 | 0.08 | 0.10 | 0.07 | 0.03 | 0.04 | 0.11 | 0.12 | 0.11 | 0.08 |
| Unplaced | 0.05 | 0.03 | 0.00 | 0.01 | 0.01 | 0.00 | 0.00 | 0.03 | 0.00 | 0.00 | 0.09 | 0.00 | 0.09 | 0.05 | 0.07 | 0.10 | 0.02 | 0.04 | 0.07 | 0.06 | 0.07 | 0.03 | 0.05 | 0.04 |

  


---

  


# Supplementary Table 5: *S5*

| S5. Distribution of intervals relative to chromosome length | | | | | | | | | | | | |
|  |  |  |  |  |  |  |  |  |  |  |  |  |
| **Chr** | **Chr length** | **Genes[1]** | **Gene density** | **Observed intervals** | **Expected intervals** | **Interval density** |  | protein\_coding |  |  |  |  |
| 1 | 195,276,750 | 2,234 | 1.14E-005 | 83 | 54 | 4.25E-007 |  | 2015 |  | Genome size | 1,001,787,339 | bp |
| 2 | 148,809,762 | 1,502 | 1.01E-005 | 47 | 41 | 3.16E-007 |  | 1323 |  | Total interval count | 279 |  |
| 3 | 110,447,801 | 1,289 | 1.17E-005 | 34 | 31 | 3.08E-007 |  | 1168 |  | Expected interval density | 2.79E-007 |  |
| 4 | 90,216,835 | 1,176 | 1.30E-005 | 32 | 25 | 3.55E-007 |  | 1071 |  | Expected interval distribution (1 per) | 3,590,636 | bp |
| 5 | 59,580,361 | 1,009 | 1.69E-005 | 16 | 17 | 2.69E-007 |  | 921 |  |  |  |  |
| 6 | 34,951,654 | 553 | 1.58E-005 | 7 | 10 | 2.00E-007 |  | 521 |  | Genes (total) | 15,863 |  |
| 7 | 36,245,040 | 532 | 1.47E-005 | 8 | 10 | 2.21E-007 |  | 491 |  | Genes (protein\_coding) | 14,459 |  |
| 8 | 28,767,244 | 570 | 1.98E-005 | 5 | 8 | 1.74E-007 |  | 510 |  |  |  |  |
| 9 | 23,441,680 | 482 | 2.06E-005 | 4 | 7 | 1.71E-007 |  | 439 |  |  |  |  |
| 10 | 19,911,089 | 455 | 2.29E-005 | 1 | 6 | 5.02E-008 |  | 405 |  |  |  |  |
| 11 | 19,401,079 | 396 | 2.04E-005 | 3 | 5 | 1.55E-007 |  | 365 |  |  |  |  |
| 12 | 19,897,011 | 366 | 1.84E-005 | 6 | 6 | 3.02E-007 |  | 342 |  |  |  |  |
| 13 | 17,760,035 | 363 | 2.04E-005 | 6 | 5 | 3.38E-007 |  | 341 |  |  |  |  |
| 14 | 15,161,805 | 437 | 2.88E-005 | 2 | 4 | 1.32E-007 |  | 413 |  |  |  |  |
| 15 | 12,656,803 | 384 | 3.03E-005 | 1 | 4 | 7.90E-008 |  | 352 |  |  |  |  |
| 16 | 535,270 | 47 | 8.78E-005 | 0 | 0 | 0.00E+000 |  | 46 |  |  |  |  |
| 17 | 10,454,150 | 319 | 3.05E-005 | 1 | 3 | 9.57E-008 |  | 287 |  |  |  |  |
| 18 | 11,219,875 | 341 | 3.04E-005 | 0 | 3 | 0.00E+000 |  | 323 |  |  |  |  |
| 19 | 9,983,394 | 348 | 3.49E-005 | 1 | 3 | 1.00E-007 |  | 311 |  |  |  |  |
| 20 | 14,302,601 | 379 | 2.65E-005 | 3 | 4 | 2.10E-007 |  | 347 |  |  |  |  |
| 21 | 6,802,778 | 262 | 3.85E-005 | 0 | 2 | 0.00E+000 |  | 250 |  |  |  |  |
| 22 | 4,081,097 | 125 | 3.06E-005 | 0 | 1 | 0.00E+000 |  | 123 |  |  |  |  |
| 23 | 5,723,239 | 247 | 4.32E-005 | 0 | 2 | 0.00E+000 |  | 222 |  |  |  |  |
| 24 | 6,323,281 | 202 | 3.19E-005 | 1 | 2 | 1.58E-007 |  | 182 |  |  |  |  |
| 25 | 2,191,139 | 183 | 8.35E-005 | 0 | 1 | 0.00E+000 |  | 172 |  |  |  |  |
| 26 | 5,329,985 | 261 | 4.90E-005 | 0 | 1 | 0.00E+000 |  | 248 |  |  |  |  |
| 27 | 5,209,285 | 271 | 5.20E-005 | 0 | 1 | 0.00E+000 |  | 254 |  |  |  |  |
| 28 | 4,742,627 | 271 | 5.71E-005 | 0 | 1 | 0.00E+000 |  | 253 |  |  |  |  |
| Z | 82,363,669 | 859 | 1.04E-005 | 18 | 23 | 2.19E-007 |  | 764 |  |  |  |  |
|  |  |  |  |  |  |  |  |  |  |  |  |  |
| [1] Includes protein coding and non-protein coding genes in the Ensembl Genes 78 database for Galgal4 | | | | | | |  |  |  |  |  |  |
|  |  |  |  |  |  |  |  |  |  |  |  |  |
| **Pearson's product-moment correlation** | | | | | |  |  |  |  |  |  |  |
|  |  |  |  |  |  |  |  |  |  |  |  |  |
| **Data.X** | **Data.Y** | ***t*** | ***df*** | ***P*** | ***r*** |  |  |  |  |  |  |  |
| Chr length | Observed intervals | 24.8111 | 27 | 2.20E-016 | 0.9787658 |  |  |  |  |  |  |  |
| Genes | Observed intervals | 21.2296 | 27 | 2.20E-016 | 0.9713283 |  |  |  |  |  |  |  |
| Chr.length | Interval density | 5.9669 | 27 | 2.31E-006 | 0.7541347 |  |  |  |  |  |  |  |
| Gene density | Interval density | -5.6424 | 27 | 5.46E-006 | -0.7355989 |  |  |  |  |  |  |  |

  


---

  


# Supplementary Table 6: *S6*

| S6. Intervals and their nearest transcripts (Ensembl, Galgal4.78 gtf) | | | | | | | | | | | | | | |
|  |  |  |  |  |  |  |  |  |  |  |  |  |  |  |
| **chr** | **start** | **end** | **Nearest transcript distance** | **transcript\_id** | **transcript\_version** | **gene\_id** | **gene\_version** | **gene start** | **gene end** | **strand** | **gene\_name** | **gene\_biotype** | **transcript\_name** | **transcript\_biotype** |
| 1 | 7414982 | 7415621 | 7339 | ENSGALT00000010664 | 4 | ENSGALG00000006606 | 4 | 7422961 | 7518502 | + | KIAA1324L | protein\_coding | KIAA1324L-201 | protein\_coding |
| 1 | 7919664 | 7919997 | 132812 | ENSGALT00000043210 | 1 | ENSGALG00000028689 | 1 | 7786733 | 7786851 | + |  | miRNA |  | miRNA |
| 1 | 8127806 | 8128614 | 48489 | ENSGALT00000010596 | 3 | ENSGALG00000006563 | 3 | 8177104 | 8318907 | + | SEMA3D | protein\_coding | SEMA3D-201 | protein\_coding |
| 1 | 15904038 | 15904160 | 25425 | ENSGALT00000030740 | 3 | ENSGALG00000019397 | 3 | 15929586 | 15971452 | - | CERK | protein\_coding | CERK-201 | protein\_coding |
| 1 | 18836195 | 18836791 | 111307 | ENSGALT00000039200 | 2 | ENSGALG00000023576 | 2 | 18948099 | 18951794 | - | PIM3 | protein\_coding | PIM3-201 | protein\_coding |
| 1 | 21322138 | 21322493 | 85505 | ENSGALT00000042610 | 1 | ENSGALG00000025697 | 1 | 21236511 | 21236632 | - | U1 | snRNA | U1-201 | snRNA |
| 1 | 23890739 | 23891336 | 172699 | ENSGALT00000014827 | 4 | ENSGALG00000009104 | 4 | 24064036 | 24111623 | + |  | protein\_coding |  | protein\_coding |
| 1 | 28836094 | 28836942 | 0 | ENSGALT00000038834 | 2 | ENSGALG00000009523 | 4 | 28777011 | 28942648 | + | CNTN1 | protein\_coding | CNTN1-201 | protein\_coding |
| 1 | 31728916 | 31729579 | 5172 | ENSGALT00000015872 | 4 | ENSGALG00000009755 | 4 | 31675731 | 31723743 | - | LRIG3 | protein\_coding | LRIG3-201 | protein\_coding |
| 1 | 32130327 | 32131074 | 48046 | ENSGALT00000015878 | 4 | ENSGALG00000009759 | 4 | 31999958 | 32082280 | + | SLC16A7 | protein\_coding | SLC16A7-201 | protein\_coding |
| 1 | 32312218 | 32313185 | 229937 | ENSGALT00000015878 | 4 | ENSGALG00000009759 | 4 | 31999958 | 32082280 | + | SLC16A7 | protein\_coding | SLC16A7-201 | protein\_coding |
| 1 | 32508365 | 32508761 | 165958 | ENSGALT00000030682 | 3 | ENSGALG00000019361 | 3 | 32674720 | 32843052 | - | FAM19A2 | protein\_coding | FAM19A2-201 | protein\_coding |
| 1 | 34786290 | 34786522 | 16982 | ENSGALT00000016099 | 4 | ENSGALG00000009899 | 4 | 34744581 | 34769307 | + | CAND1 | protein\_coding | CAND1-201 | protein\_coding |
| 1 | 37362031 | 37362653 | 194492 | ENSGALT00000016589 | 4 | ENSGALG00000010204 | 4 | 37557146 | 37660197 | - | KCNC2 | protein\_coding | KCNC2-201 | protein\_coding |
| 1 | 41194458 | 41195110 | 212099 | ENSGALT00000017879 | 3 | ENSGALG00000010975 | 3 | 40841112 | 40982358 | + | TMTC2 | protein\_coding | TMTC2-201 | protein\_coding |
| 1 | 41318900 | 41319777 | 198034 | ENSGALT00000043826 | 1 | ENSGALG00000027733 | 1 | 41517812 | 41517930 | - |  | miRNA |  | miRNA |
| 1 | 42541895 | 42542614 | 122383 | ENSGALT00000018193 | 4 | ENSGALG00000011159 | 4 | 42664998 | 42677602 | - | C12orf50 | protein\_coding | C12orf50-201 | protein\_coding |
| 1 | 43882636 | 43883815 | 9768 | ENSGALT00000018392 | 4 | ENSGALG00000011271 | 4 | 43860883 | 43872867 | - | LUM | protein\_coding | LUM-201 | protein\_coding |
| 1 | 44606699 | 44607552 | 43902 | ENSGALT00000018404 | 4 | ENSGALG00000011280 | 4 | 44497302 | 44562796 | - | EEA1 | protein\_coding | EEA1-201 | protein\_coding |
| 1 | 46027902 | 46028388 | 59001 | ENSGALT00000042323 | 1 | ENSGALG00000025410 | 1 | 46087390 | 46087480 | + | gga-mir-1691 | miRNA | gga-mir-1691-201 | miRNA |
| 1 | 47364771 | 47365611 | 0 | ENSGALT00000018969 | 4 | ENSGALG00000011614 | 4 | 47292275 | 47464213 | + | ANO4 | protein\_coding | ANO4-201 | protein\_coding |
| 1 | 52743902 | 52744500 | 0 | ENSGALT00000020508 | 3 | ENSGALG00000012559 | 4 | 52524419 | 52799322 | + | LARGE | protein\_coding | LARGE-201 | protein\_coding |
| 1 | 63571454 | 63572079 | 10387 | ENSGALT00000009596 | 4 | ENSGALG00000019280 | 3 | 63504395 | 63561066 | - | LMO3 | protein\_coding | LMO3-201 | protein\_coding |
| 1 | 67550436 | 67551166 | 23646 | ENSGALT00000022731 | 3 | ENSGALG00000014037 | 3 | 67510064 | 67526789 | - |  | protein\_coding |  | protein\_coding |
| 1 | 70454672 | 70455338 | 0 | ENSGALT00000023003 | 4 | ENSGALG00000014226 | 4 | 70394229 | 70462643 | - | KIAA0930 | protein\_coding | KIAA0930-201 | protein\_coding |
| 1 | 73743848 | 73744438 | 16993 | ENSGALT00000027933 | 4 | ENSGALG00000017279 | 4 | 73761432 | 73763327 | + | KCNA5 | protein\_coding | KCNA5-201 | protein\_coding |
| 1 | 77444595 | 77445260 | 14937 | ENSGALT00000023783 | 4 | ENSGALG00000014749 | 4 | 77460198 | 77533109 | - | EPHB6 | protein\_coding | EPHB6-201 | protein\_coding |
| 1 | 84555430 | 84556039 | 33833 | ENSGALT00000024704 | 4 | ENSGALG00000015307 | 4 | 84389704 | 84521596 | - | ABI3BP | protein\_coding | ABI3BP-201 | protein\_coding |
| 1 | 87425101 | 87425826 | 186150 | ENSGALT00000024797 | 4 | ENSGALG00000015367 | 4 | 87611977 | 87655399 | + | PVRL3 | protein\_coding | PVRL3-201 | protein\_coding |
| 1 | 89899733 | 89900560 | 316113 | ENSGALT00000024870 | 3 | ENSGALG00000015418 | 3 | 90216674 | 90572208 | + | EPHA6 | protein\_coding | EPHA6-201 | protein\_coding |
| 1 | 90099919 | 90100600 | 116073 | ENSGALT00000024870 | 3 | ENSGALG00000015418 | 3 | 90216674 | 90572208 | + | EPHA6 | protein\_coding | EPHA6-201 | protein\_coding |
| 1 | 93473773 | 93474297 | 288489 | ENSGALT00000042090 | 1 | ENSGALG00000025177 | 1 | 93185199 | 93185283 | - | gga-mir-1806 | miRNA | gga-mir-1806-201 | miRNA |
| 1 | 95854597 | 95855224 | 0 | ENSGALT00000025009 | 4 | ENSGALG00000015511 | 4 | 95642548 | 95948471 | + | ROBO1 | protein\_coding | ROBO1-201 | protein\_coding |
| 1 | 96156897 | 96157369 | 83297 | ENSGALT00000025020 | 4 | ENSGALG00000015519 | 4 | 96240667 | 96396977 | - |  | protein\_coding |  | protein\_coding |
| 1 | 96182372 | 96182610 | 58056 | ENSGALT00000025020 | 4 | ENSGALG00000015519 | 4 | 96240667 | 96396977 | - |  | protein\_coding |  | protein\_coding |
| 1 | 99050515 | 99051202 | 144139 | ENSGALT00000025369 | 3 | ENSGALG00000015730 | 3 | 98898052 | 98906375 | - | TMPRSS15 | protein\_coding | TMPRSS15-201 | protein\_coding |
| 1 | 99136310 | 99136953 | 229934 | ENSGALT00000025369 | 3 | ENSGALG00000015730 | 3 | 98898052 | 98906375 | - | TMPRSS15 | protein\_coding | TMPRSS15-201 | protein\_coding |
| 1 | 101165329 | 101166193 | 322229 | ENSGALT00000028603 | 1 | ENSGALG00000017896 | 1 | 101488423 | 101488527 | + | U6 | snRNA | U6-201 | snRNA |
| 1 | 101333491 | 101334404 | 154018 | ENSGALT00000028603 | 1 | ENSGALG00000017896 | 1 | 101488423 | 101488527 | + | U6 | snRNA | U6-201 | snRNA |
| 1 | 113945460 | 113946362 | 253176 | ENSGALT00000026271 | 3 | ENSGALG00000022792 | 2 | 113688262 | 113692283 | - |  | protein\_coding |  | protein\_coding |
| 1 | 114080840 | 114081279 | 152387 | ENSGALT00000026250 | 4 | ENSGALG00000016281 | 4 | 114233667 | 114932830 | + | DMD | protein\_coding | DMD-201 | protein\_coding |
| 1 | 117166631 | 117167523 | 118969 | ENSGALT00000026316 | 4 | ENSGALG00000016312 | 4 | 117286493 | 117477666 | - | POLA1 | protein\_coding | POLA1-201 | protein\_coding |
| 1 | 123156456 | 123157321 | 0 | ENSGALT00000026784 | 4 | ENSGALG00000016594 | 4 | 122962850 | 123267815 | - | FRMPD4 | protein\_coding | FRMPD4-201 | protein\_coding |
| 1 | 125858928 | 125859550 | 0 | ENSGALT00000026830 | 4 | ENSGALG00000016622 | 4 | 125766037 | 125867934 | - | STS | protein\_coding | STS-201 | protein\_coding |
| 1 | 126794615 | 126795484 | 385817 | ENSGALT00000045890 | 1 | ENSGALG00000016628 | 4 | 126290134 | 126408797 | + | NLGN4 | protein\_coding | NLGN4-203 | protein\_coding |
| 1 | 127188644 | 127189303 | 319126 | ENSGALT00000030281 | 3 | ENSGALG00000016629 | 4 | 127508430 | 127539291 | + | PRKX | protein\_coding | PRKX-201 | protein\_coding |
| 1 | 131477317 | 131477931 | 0 | ENSGALT00000036592 | 2 | ENSGALG00000016751 | 4 | 131443219 | 131531601 | + | TMEM131 | protein\_coding | TMEM131-201 | protein\_coding |
| 1 | 140037593 | 140038154 | 10899 | ENSGALT00000036542 | 2 | ENSGALG00000022762 | 2 | 140049054 | 140070659 | + |  | protein\_coding |  | protein\_coding |
| 1 | 140710345 | 140715423 | 67959 | ENSGALT00000042402 | 1 | ENSGALG00000025489 | 1 | 140642265 | 140642385 | - |  | miRNA |  | miRNA |
| 1 | 140995796 | 140996700 | 353410 | ENSGALT00000042402 | 1 | ENSGALG00000025489 | 1 | 140642265 | 140642385 | - |  | miRNA |  | miRNA |
| 1 | 141097673 | 141098554 | 455287 | ENSGALT00000042402 | 1 | ENSGALG00000025489 | 1 | 140642265 | 140642385 | - |  | miRNA |  | miRNA |
| 1 | 142068256 | 142068910 | 3824 | ENSGALT00000027242 | 4 | ENSGALG00000016863 | 4 | 142057711 | 142064431 | - | C1H13ORF27 | protein\_coding | C1H13ORF27-201 | protein\_coding |
| 1 | 146731910 | 146732833 | 98219 | ENSGALT00000027322 | 4 | ENSGALG00000016902 | 4 | 146831053 | 147232836 | - | GPC5 | protein\_coding | GPC5-201 | protein\_coding |
| 1 | 146761527 | 146762190 | 68862 | ENSGALT00000027322 | 4 | ENSGALG00000016902 | 4 | 146831053 | 147232836 | - | GPC5 | protein\_coding | GPC5-201 | protein\_coding |
| 1 | 148564562 | 148568615 | 71660 | ENSGALT00000044859 | 1 | ENSGALG00000026361 | 1 | 148640276 | 148643140 | - | SLITRK5 | protein\_coding | SLITRK5-201 | protein\_coding |
| 1 | 149162760 | 149166227 | 311936 | ENSGALT00000027325 | 4 | ENSGALG00000016904 | 4 | 149478164 | 149480686 | + | SLITRK6 | protein\_coding | SLITRK6-201 | protein\_coding |
| 1 | 149501163 | 149502040 | 20476 | ENSGALT00000027325 | 4 | ENSGALG00000016904 | 4 | 149478164 | 149480686 | + | SLITRK6 | protein\_coding | SLITRK6-201 | protein\_coding |
| 1 | 149935105 | 149935762 | 454418 | ENSGALT00000027325 | 4 | ENSGALG00000016904 | 4 | 149478164 | 149480686 | + | SLITRK6 | protein\_coding | SLITRK6-201 | protein\_coding |
| 1 | 150131206 | 150132006 | 289558 | ENSGALT00000027326 | 2 | ENSGALG00000016905 | 2 | 150421565 | 150423643 | + | SLITRK1 | protein\_coding | SLITRK1-201 | protein\_coding |
| 1 | 151384845 | 151385716 | 510425 | ENSGALT00000027327 | 4 | ENSGALG00000016906 | 4 | 151896142 | 151897919 | + | SPRY2 | protein\_coding | SPRY2-201 | protein\_coding |
| 1 | 151407118 | 151410307 | 485834 | ENSGALT00000027327 | 4 | ENSGALG00000016906 | 4 | 151896142 | 151897919 | + | SPRY2 | protein\_coding | SPRY2-201 | protein\_coding |
| 1 | 153879253 | 153880132 | 73947 | ENSGALT00000043460 | 1 | ENSGALG00000027869 | 1 | 153954080 | 153954189 | + |  | miRNA |  | miRNA |
| 1 | 153995978 | 153996637 | 41788 | ENSGALT00000043460 | 1 | ENSGALG00000027869 | 1 | 153954080 | 153954189 | + |  | miRNA |  | miRNA |
| 1 | 157450062 | 157450954 | 335756 | ENSGALT00000027328 | 3 | ENSGALG00000016907 | 3 | 156898969 | 157114305 | + |  | protein\_coding |  | protein\_coding |
| 1 | 157698899 | 157699774 | 309730 | ENSGALT00000044044 | 1 | ENSGALG00000028006 | 1 | 158009505 | 158009600 | - |  | miRNA |  | miRNA |
| 1 | 159294092 | 159294382 | 561512 | ENSGALT00000042622 | 1 | ENSGALG00000025709 | 1 | 158732522 | 158732579 | - | SNORD63 | snoRNA | SNORD63-201 | snoRNA |
| 1 | 159625843 | 159626482 | 893263 | ENSGALT00000042622 | 1 | ENSGALG00000025709 | 1 | 158732522 | 158732579 | - | SNORD63 | snoRNA | SNORD63-201 | snoRNA |
| 1 | 160455218 | 160455708 | 569190 | ENSGALT00000023466 | 4 | ENSGALG00000014545 | 4 | 161024899 | 161059435 | - |  | protein\_coding |  | protein\_coding |
| 1 | 160463921 | 160464245 | 560653 | ENSGALT00000023466 | 4 | ENSGALG00000014545 | 4 | 161024899 | 161059435 | - |  | protein\_coding |  | protein\_coding |
| 1 | 162222519 | 162223183 | 289813 | ENSGALT00000027377 | 6 | ENSGALG00000016937 | 6 | 161696600 | 161932705 | + | DIAPH3 | protein\_coding | DIAPH3-201 | protein\_coding |
| 1 | 163501535 | 163501830 | 418086 | ENSGALT00000042701 | 1 | ENSGALG00000029031 | 1 | 163083292 | 163083448 | - |  | miRNA |  | miRNA |
| 1 | 163694748 | 163695372 | 467669 | ENSGALT00000044640 | 1 | ENSGALG00000027756 | 1 | 164163042 | 164163205 | - | uc\_338 | misc\_RNA | uc\_338-201 | misc\_RNA |
| 1 | 163906737 | 163910850 | 252191 | ENSGALT00000044640 | 1 | ENSGALG00000027756 | 1 | 164163042 | 164163205 | - | uc\_338 | misc\_RNA | uc\_338-201 | misc\_RNA |
| 1 | 164414998 | 164416037 | 44308 | ENSGALT00000042312 | 1 | ENSGALG00000025399 | 1 | 164370610 | 164370689 | - | snoU2\_19 | snoRNA | snoU2\_19-201 | snoRNA |
| 1 | 171152000 | 171152790 | 0 | ENSGALT00000030118 | 3 | ENSGALG00000017044 | 4 | 171102508 | 171244344 | + | TRPC4 | protein\_coding | TRPC4-201 | protein\_coding |
| 1 | 179728775 | 179729463 | 9708 | ENSGALT00000027729 | 4 | ENSGALG00000017168 | 4 | 179739172 | 179741023 | - |  | protein\_coding |  | protein\_coding |
| 1 | 180746896 | 180747970 | 109685 | ENSGALT00000036434 | 2 | ENSGALG00000017178 | 4 | 180413530 | 180637210 | - | GRIA4 | protein\_coding | GRIA4-202 | protein\_coding |
| 1 | 182832847 | 182834082 | 0 | ENSGALT00000027797 | 4 | ENSGALG00000017197 | 4 | 182715565 | 183138508 | - | FAR-2 | protein\_coding | FAR-2-201 | protein\_coding |
| 1 | 184347893 | 184348804 | 0 | ENSGALT00000027799 | 4 | ENSGALG00000017199 | 4 | 184236229 | 184401679 | + | MAML2 | protein\_coding | MAML2-201 | protein\_coding |
| 1 | 185856831 | 185857511 | 0 | ENSGALT00000027850 | 4 | ENSGALG00000017229 | 4 | 185616759 | 185962787 | - | FAT3 | protein\_coding | FAT3-201 | protein\_coding |
| 1 | 186846427 | 186847073 | 0 | ENSGALT00000027858 | 3 | ENSGALG00000017234 | 4 | 186845641 | 186876181 | - | FOLH1 | protein\_coding | FOLH1-201 | protein\_coding |
| 1 | 187340668 | 187342356 | 0 | ENSGALT00000027866 | 2 | ENSGALG00000017238 | 3 | 187158336 | 187407442 | + | GRM5 | protein\_coding | GRM5-201 | protein\_coding |
| 1 | 188148835 | 188149736 | 0 | ENSGALT00000027880 | 4 | ENSGALG00000017246 | 4 | 188071573 | 188189672 | + | ME3 | protein\_coding | ME3-201 | protein\_coding |
| 10 | 9703183 | 9703845 | 0 | ENSGALT00000007955 | 4 | ENSGALG00000004960 | 4 | 9669985 | 9805190 | - | FBN1 | protein\_coding | FBN1-201 | protein\_coding |
| 12 | 335263 | 335965 | 122728 | ENSGALT00000044759 | 1 | ENSGALG00000026085 | 1 | 203478 | 212534 | + | DUSP7 | protein\_coding | DUSP7-201 | protein\_coding |
| 12 | 3240697 | 3240898 | 1120 | ENSGALT00000010583 | 3 | ENSGALG00000027650 | 1 | 3237733 | 3239576 | + |  | protein\_coding |  | protein\_coding |
| 12 | 3277416 | 3278316 | 1539 | ENSGALT00000007442 | 4 | ENSGALG00000004670 | 4 | 3279856 | 3290698 | - | ATRIP | protein\_coding | ATRIP-201 | protein\_coding |
| 12 | 6823591 | 6824459 | 0 | ENSGALT00000008433 | 4 | ENSGALG00000005249 | 4 | 6815655 | 6886985 | - | BICD2 | protein\_coding | BICD2-201 | protein\_coding |
| 12 | 15106212 | 15106979 | 0 | ENSGALT00000045436 | 1 | ENSGALG00000027934 | 1 | 14921472 | 15122905 | + | FAM19A1 | protein\_coding | FAM19A1-201 | protein\_coding |
| 12 | 17400816 | 17401479 | 103481 | ENSGALT00000012841 | 4 | ENSGALG00000007911 | 4 | 17219615 | 17297334 | + | CHL1 | protein\_coding | CHL1-201 | protein\_coding |
| 2 | 1183407 | 1184047 | 19175 | ENSGALT00000039264 | 2 | ENSGALG00000005212 | 4 | 1146175 | 1164231 | + | GHRHR | protein\_coding | GHRHR-202 | protein\_coding |
| 2 | 3491394 | 3492279 | 36168 | ENSGALT00000043912 | 1 | ENSGALG00000005448 | 3 | 3420143 | 3455225 | - | MYL3 | protein\_coding | MYL3-202 | protein\_coding |
| 2 | 5464380 | 5464705 | 0 | ENSGALT00000031281 | 3 | ENSGALG00000006112 | 4 | 5408261 | 5613834 | + | SCN5A | protein\_coding | SCN5A-201 | protein\_coding |
| 2 | 7818049 | 7818465 | 8995 | ENSGALT00000028082 | 3 | ENSGALG00000017394 | 3 | 7827461 | 7832977 | + | INSIG1 | protein\_coding | INSIG1-201 | protein\_coding |
| 2 | 10512425 | 10512887 | 0 | ENSGALT00000010993 | 4 | ENSGALG00000006797 | 4 | 10438685 | 10736438 | - | ADARB2 | protein\_coding | ADARB2-201 | protein\_coding |
| 2 | 11018857 | 11019506 | 282418 | ENSGALT00000010993 | 4 | ENSGALG00000006797 | 4 | 10438685 | 10736438 | - | ADARB2 | protein\_coding | ADARB2-201 | protein\_coding |
| 2 | 16258680 | 16259495 | 0 | ENSGALT00000012375 | 4 | ENSGALG00000007647 | 4 | 16182205 | 16276588 | - | MYO3A | protein\_coding | MYO3A-201 | protein\_coding |
| 2 | 20044191 | 20044405 | 24085 | ENSGALT00000014205 | 3 | ENSGALG00000008728 | 4 | 20004930 | 20020105 | - | PTER | protein\_coding | PTER-201 | protein\_coding |
| 2 | 20971534 | 20972437 | 76555 | ENSGALT00000014634 | 4 | ENSGALG00000008997 | 4 | 20889645 | 20894978 | - | STEAP4 | protein\_coding | STEAP4-201 | protein\_coding |
| 2 | 21240687 | 21244651 | 0 | ENSGALT00000014653 | 4 | ENSGALG00000009004 | 5 | 21054837 | 21268859 | + | ZNF804B | protein\_coding | ZNF804B-201 | protein\_coding |
| 2 | 23631823 | 23632481 | 69542 | ENSGALT00000015703 | 4 | ENSGALG00000009641 | 4 | 23702024 | 23740814 | + | COL1A2 | protein\_coding | COL1A2-201 | protein\_coding |
| 2 | 34296518 | 34297385 | 3655 | ENSGALT00000018199 | 4 | ENSGALG00000011164 | 4 | 34130670 | 34292862 | + | CHN2 | protein\_coding | CHN2-201 | protein\_coding |
| 2 | 47663562 | 47663887 | 48112 | ENSGALT00000019843 | 3 | ENSGALG00000012149 | 4 | 47596636 | 47615449 | + | HERPUD2 | protein\_coding | HERPUD2-201 | protein\_coding |
| 2 | 50798296 | 50798951 | 0 | ENSGALT00000037440 | 2 | ENSGALG00000026371 | 1 | 50690130 | 50901617 | + |  | protein\_coding |  | protein\_coding |
| 2 | 51801055 | 51801954 | 46381 | ENSGALT00000020160 | 3 | ENSGALG00000012337 | 3 | 51752758 | 51754673 | + | MRPL32 | protein\_coding | MRPL32-201 | protein\_coding |
| 2 | 53111756 | 53112017 | 101242 | ENSGALT00000020200 | 4 | ENSGALG00000012369 | 4 | 52757783 | 53010513 | - | TPK1 | protein\_coding | TPK1-201 | protein\_coding |
| 2 | 58047649 | 58047977 | 16342 | ENSGALT00000031085 | 3 | ENSGALG00000019587 | 3 | 58064320 | 58070320 | + |  | protein\_coding |  | protein\_coding |
| 2 | 65341352 | 65341989 | 0 | ENSGALT00000020890 | 4 | ENSGALG00000012805 | 4 | 65290448 | 65521910 | - | FARS2 | protein\_coding | FARS2-201 | protein\_coding |
| 2 | 73742831 | 73743267 | 165877 | ENSGALT00000037142 | 2 | ENSGALG00000022947 | 2 | 73909145 | 73909823 | - |  | protein\_coding |  | protein\_coding |
| 2 | 75447503 | 75448357 | 34707 | ENSGALT00000021128 | 4 | ENSGALG00000012954 | 4 | 75483065 | 75633537 | + | MYO10 | protein\_coding | MYO10-201 | protein\_coding |
| 2 | 77178330 | 77179069 | 125982 | ENSGALT00000042381 | 1 | ENSGALG00000025468 | 1 | 77052244 | 77052347 | - | U6 | snRNA | U6-201 | snRNA |
| 2 | 80333585 | 80334273 | 113392 | ENSGALT00000021359 | 4 | ENSGALG00000013081 | 4 | 80447666 | 80505627 | + | VWC2 | protein\_coding | VWC2-201 | protein\_coding |
| 2 | 81458689 | 81462600 | 279901 | ENSGALT00000045764 | 1 | ENSGALG00000026637 | 1 | 81742502 | 81745670 | - |  | protein\_coding |  | protein\_coding |
| 2 | 81965001 | 81966072 | 219330 | ENSGALT00000045764 | 1 | ENSGALG00000026637 | 1 | 81742502 | 81745670 | - |  | protein\_coding |  | protein\_coding |
| 2 | 82574297 | 82575518 | 249546 | ENSGALT00000046240 | 1 | ENSGALG00000028075 | 1 | 82285136 | 82324750 | + | VSTM2A | protein\_coding | VSTM2A-201 | protein\_coding |
| 2 | 87444008 | 87444641 | 308946 | ENSGALT00000021555 | 3 | ENSGALG00000013194 | 4 | 87131471 | 87135061 | + | IRX1 | protein\_coding | IRX1-201 | protein\_coding |
| 2 | 92058477 | 92059335 | 40944 | ENSGALT00000022302 | 3 | ENSGALG00000013720 | 3 | 91997447 | 92017532 | + | FBXO15 | protein\_coding | FBXO15-201 | protein\_coding |
| 2 | 92070857 | 92071755 | 53324 | ENSGALT00000022302 | 3 | ENSGALG00000013720 | 3 | 91997447 | 92017532 | + | FBXO15 | protein\_coding | FBXO15-201 | protein\_coding |
| 2 | 92856996 | 92858219 | 49759 | ENSGALT00000044305 | 1 | ENSGALG00000028456 | 1 | 92807129 | 92807236 | - |  | miRNA |  | miRNA |
| 2 | 93341773 | 93342126 | 39209 | ENSGALT00000043448 | 1 | ENSGALG00000027455 | 1 | 93302421 | 93302563 | - | uc\_338 | misc\_RNA | uc\_338-201 | misc\_RNA |
| 2 | 94577735 | 94578007 | 84436 | ENSGALT00000022360 | 4 | ENSGALG00000013766 | 4 | 94662444 | 94666094 | + | DSEL | protein\_coding | DSEL-201 | protein\_coding |
| 2 | 94594272 | 94594911 | 67532 | ENSGALT00000022360 | 4 | ENSGALG00000013766 | 4 | 94662444 | 94666094 | + | DSEL | protein\_coding | DSEL-201 | protein\_coding |
| 2 | 95480957 | 95481836 | 0 | ENSGALT00000022381 | 3 | ENSGALG00000013782 | 3 | 95477061 | 95554752 | - | CDH7 | protein\_coding | CDH7-201 | protein\_coding |
| 2 | 104297204 | 104297861 | 151322 | ENSGALT00000024417 | 2 | ENSGALG00000015132 | 2 | 104449184 | 104562821 | - | CDH2 | protein\_coding | CDH2-201 | protein\_coding |
| 2 | 106764059 | 106764678 | 0 | ENSGALT00000024522 | 4 | ENSGALG00000015198 | 4 | 106756439 | 106849498 | - |  | protein\_coding |  | protein\_coding |
| 2 | 109248726 | 109249064 | 25907 | ENSGALT00000024613 | 4 | ENSGALG00000015254 | 4 | 109274972 | 109324045 | - | ST18 | protein\_coding | ST18-201 | protein\_coding |
| 2 | 115446270 | 115446856 | 0 | ENSGALT00000025094 | 4 | ENSGALG00000015564 | 4 | 115324553 | 115492355 | + | PREX2 | protein\_coding | PREX2-201 | protein\_coding |
| 2 | 123095199 | 123095828 | 112271 | ENSGALT00000036637 | 2 | ENSGALG00000015886 | 4 | 122953408 | 122982927 | - | CNGB3 | protein\_coding | CNGB3-201 | protein\_coding |
| 2 | 123945004 | 123945201 | 102541 | ENSGALT00000025621 | 3 | ENSGALG00000015899 | 4 | 124047743 | 124066705 | + | RIPK2 | protein\_coding | RIPK2-201 | protein\_coding |
| 2 | 124765081 | 124765718 | 57465 | ENSGALT00000036630 | 2 | ENSGALG00000015926 | 4 | 124823184 | 124932746 | - | RUNX1T1 | protein\_coding | RUNX1T1-201 | protein\_coding |
| 2 | 129140825 | 129141707 | 10978 | ENSGALT00000025890 | 4 | ENSGALG00000016069 | 4 | 129152686 | 129181081 | + | FZD6 | protein\_coding | FZD6-201 | protein\_coding |
| 2 | 131563757 | 131567794 | 31309 | ENSGALT00000025937 | 3 | ENSGALG00000016097 | 3 | 131497565 | 131532447 | + | EMC2 | protein\_coding | EMC2-201 | protein\_coding |
| 2 | 133607552 | 133608408 | 226156 | ENSGALT00000025958 | 4 | ENSGALG00000016111 | 4 | 132812344 | 133381395 | - | CSMD3 | protein\_coding | CSMD3-201 | protein\_coding |
| 2 | 142113866 | 142114543 | 8242 | ENSGALT00000026126 | 4 | ENSGALG00000016207 | 4 | 142122786 | 142207755 | - | ZFAT | protein\_coding | ZFAT-201 | protein\_coding |
| 2 | 142512897 | 142513907 | 186578 | ENSGALT00000026120 | 4 | ENSGALG00000016203 | 4 | 142700486 | 142776708 | + | KHDRBS3 | protein\_coding | KHDRBS3-201 | protein\_coding |
| 2 | 143463257 | 143468861 | 386887 | ENSGALT00000026112 | 6 | ENSGALG00000016197 | 6 | 143855749 | 144038521 | - | FAM135B | protein\_coding | FAM135B-201 | protein\_coding |
| 2 | 147548249 | 147548775 | 40241 | ENSGALT00000026027 | 1 | ENSGALG00000016154 | 1 | 147589017 | 147590231 | - | ARC | protein\_coding | ARC-201 | protein\_coding |
| 20 | 2428746 | 2429684 | 0 | ENSGALT00000003268 | 2 | ENSGALG00000002095 | 2 | 2427087 | 2448634 | - | CHMP4B | protein\_coding | CHMP4B-201 | protein\_coding |
| 20 | 7661897 | 7662786 | 39481 | ENSGALT00000008180 | 4 | ENSGALG00000005102 | 4 | 7702268 | 8001028 | + | CDH4 | protein\_coding | CDH4-201 | protein\_coding |
| 20 | 9408955 | 9409567 | 0 | ENSGALT00000009463 | 4 | ENSGALG00000005887 | 4 | 9382942 | 9424831 | - | PXDNL | protein\_coding | PXDNL-201 | protein\_coding |
| 3 | 12508635 | 12509292 | 63619 | ENSGALT00000014689 | 4 | ENSGALG00000009028 | 4 | 12436961 | 12445015 | - | BTBD3 | protein\_coding | BTBD3-201 | protein\_coding |
| 3 | 19821802 | 19823007 | 12230 | ENSGALT00000043631 | 1 | ENSGALG00000027525 | 1 | 19835238 | 19933929 | + |  | protein\_coding |  | protein\_coding |
| 3 | 22595889 | 22596501 | 62611 | ENSGALT00000016120 | 3 | ENSGALG00000009912 | 3 | 22659113 | 22664193 | + |  | protein\_coding |  | protein\_coding |
| 3 | 34782953 | 34783379 | 30160 | ENSGALT00000017474 | 3 | ENSGALG00000010737 | 3 | 34813540 | 34977620 | + | PLD5 | protein\_coding | PLD5-201 | protein\_coding |
| 3 | 36156197 | 36156846 | 278702 | ENSGALT00000017530 | 3 | ENSGALG00000010778 | 4 | 35727538 | 35877494 | - | CHRM3 | protein\_coding | CHRM3-201 | protein\_coding |
| 3 | 40908270 | 40908901 | 30501 | ENSGALT00000018269 | 4 | ENSGALG00000011205 | 4 | 40939403 | 41033678 | - | SMOC2 | protein\_coding | SMOC2-201 | protein\_coding |
| 3 | 44140622 | 44141485 | 0 | ENSGALT00000018857 | 3 | ENSGALG00000011562 | 4 | 43765827 | 44445298 | + | PARK2 | protein\_coding | PARK2-201 | protein\_coding |
| 3 | 46003530 | 46004338 | 0 | ENSGALT00000020095 | 4 | ENSGALG00000012297 | 4 | 45937157 | 46118743 | + | GRM1 | protein\_coding | GRM1-201 | protein\_coding |
| 3 | 49572068 | 49572931 | 15568 | ENSGALT00000037555 | 2 | ENSGALG00000023058 | 2 | 49554948 | 49556499 | - |  | protein\_coding |  | protein\_coding |
| 3 | 50010952 | 50011588 | 58514 | ENSGALT00000022217 | 3 | ENSGALG00000013659 | 3 | 49915188 | 49952437 | - | NOX3 | protein\_coding | NOX3-201 | protein\_coding |
| 3 | 54250109 | 54254158 | 12239 | ENSGALT00000022499 | 4 | ENSGALG00000013874 | 4 | 54204622 | 54237869 | - | PEX7 | protein\_coding | PEX7-201 | protein\_coding |
| 3 | 55831442 | 55836192 | 7547 | ENSGALT00000022662 | 4 | ENSGALG00000013987 | 4 | 55666849 | 55823894 | - | EYA4 | protein\_coding | EYA4-201 | protein\_coding |
| 3 | 59937540 | 59938220 | 0 | ENSGALT00000031739 | 3 | ENSGALG00000014844 | 4 | 59710415 | 59999592 | - | NKAIN2 | protein\_coding | NKAIN2-201 | protein\_coding |
| 3 | 60621854 | 60622499 | 23714 | ENSGALT00000023971 | 2 | ENSGALG00000014853 | 2 | 60548727 | 60598139 | - | CLVS2 | protein\_coding | CLVS2-201 | protein\_coding |
| 3 | 63745322 | 63745967 | 226022 | ENSGALT00000024165 | 4 | ENSGALG00000014979 | 4 | 63472387 | 63519299 | + | FRK | protein\_coding | FRK-201 | protein\_coding |
| 3 | 68891616 | 68892157 | 341395 | ENSGALT00000042972 | 1 | ENSGALG00000026120 | 1 | 69233553 | 69233659 | + |  | miRNA |  | miRNA |
| 3 | 70604555 | 70605387 | 195266 | ENSGALT00000024939 | 4 | ENSGALG00000015466 | 4 | 70369050 | 70409288 | + | SIM1 | protein\_coding | SIM1-201 | protein\_coding |
| 3 | 72558723 | 72559573 | 3594 | ENSGALT00000043426 | 1 | ENSGALG00000027427 | 1 | 72554067 | 72555128 | + |  | protein\_coding |  | protein\_coding |
| 3 | 73561879 | 73563101 | 99390 | ENSGALT00000025144 | 3 | ENSGALG00000015593 | 3 | 73318964 | 73462488 | + | EPHA7 | protein\_coding | EPHA7-201 | protein\_coding |
| 3 | 77502831 | 77503430 | 52841 | ENSGALT00000031669 | 3 | ENSGALG00000015860 | 4 | 77404161 | 77449989 | + | UBE3D | protein\_coding | UBE3D-201 | protein\_coding |
| 3 | 79282754 | 79283588 | 110363 | ENSGALT00000046171 | 1 | ENSGALG00000028314 | 1 | 79172283 | 79172390 | + |  | miRNA |  | miRNA |
| 3 | 83407619 | 83408514 | 252138 | ENSGALT00000043794 | 1 | ENSGALG00000025975 | 1 | 83660653 | 83660762 | - | gga-mir-6605 | miRNA | gga-mir-6605-201 | miRNA |
| 3 | 83776894 | 83777945 | 116131 | ENSGALT00000043794 | 1 | ENSGALG00000025975 | 1 | 83660653 | 83660762 | - | gga-mir-6605 | miRNA | gga-mir-6605-201 | miRNA |
| 3 | 84550518 | 84551452 | 0 | ENSGALT00000026073 | 3 | ENSGALG00000016183 | 3 | 84449717 | 84579680 | + |  | protein\_coding |  | protein\_coding |
| 3 | 84889113 | 84893120 | 0 | ENSGALT00000026224 | 3 | ENSGALG00000016263 | 3 | 84795509 | 84972849 | + | EYS | protein\_coding | EYS-201 | protein\_coding |
| 3 | 86212524 | 86213394 | 0 | ENSGALT00000026251 | 4 | ENSGALG00000016282 | 4 | 86180711 | 86221899 | - | ZNF451 | protein\_coding | ZNF451-201 | protein\_coding |
| 3 | 86869588 | 86870258 | 18255 | ENSGALT00000026278 | 4 | ENSGALG00000016294 | 4 | 86888514 | 86960499 | + | HMGCLL1 | protein\_coding | HMGCLL1-201 | protein\_coding |
| 3 | 88244141 | 88245055 | 155026 | ENSGALT00000031627 | 3 | ENSGALG00000016332 | 4 | 87966853 | 88089114 | - | MCPH1 | protein\_coding | MCPH1-201 | protein\_coding |
| 3 | 91661280 | 91662061 | 58227 | ENSGALT00000045991 | 1 | ENSGALG00000028744 | 1 | 91720289 | 91726599 | - | TMEM18 | protein\_coding | TMEM18-201 | protein\_coding |
| 3 | 93697657 | 93698572 | 338278 | ENSGALT00000044792 | 1 | ENSGALG00000027532 | 1 | 93337088 | 93359378 | + | COLEC11 | protein\_coding | COLEC11-201 | protein\_coding |
| 3 | 95722787 | 95723662 | 0 | ENSGALT00000026486 | 4 | ENSGALG00000016419 | 4 | 95690442 | 95751830 | + | ASAP2 | protein\_coding | ASAP2-201 | protein\_coding |
| 3 | 96957515 | 96961612 | 0 | ENSGALT00000026546 | 4 | ENSGALG00000016455 | 4 | 96906122 | 96965158 | + | GREB1 | protein\_coding | GREB1-201 | protein\_coding |
| 3 | 99426024 | 99426621 | 252062 | ENSGALT00000026562 | 2 | ENSGALG00000016463 | 4 | 99136709 | 99173961 | - | FAM49A | protein\_coding | FAM49A-201 | protein\_coding |
| 3 | 102777226 | 102778143 | 141984 | ENSGALT00000026605 | 3 | ENSGALG00000016494 | 3 | 102920128 | 102956873 | + |  | protein\_coding |  | protein\_coding |
| 4 | 11123901 | 11124467 | 0 | ENSGALT00000012073 | 4 | ENSGALG00000007466 | 4 | 11117414 | 11126899 | + | EDNRB2 | protein\_coding | EDNRB2-201 | protein\_coding |
| 4 | 15864150 | 15864742 | 18194 | ENSGALT00000013865 | 4 | ENSGALG00000008512 | 4 | 15700804 | 15845955 | - | GRIA3 | protein\_coding | GRIA3-201 | protein\_coding |
| 4 | 16267586 | 16268257 | 74751 | ENSGALT00000013866 | 3 | ENSGALG00000008513 | 3 | 16343009 | 16344697 | + | C1GALT1C1 | protein\_coding | C1GALT1C1-201 | protein\_coding |
| 4 | 19211542 | 19212344 | 654 | ENSGALT00000038924 | 2 | ENSGALG00000009239 | 4 | 19209334 | 19210887 | + | TLR2-2 | protein\_coding | TLR2-2-201 | protein\_coding |
| 4 | 20401588 | 20402427 | 37464 | ENSGALT00000015270 | 4 | ENSGALG00000009373 | 4 | 20356529 | 20364123 | - | CTSO | protein\_coding | CTSO-201 | protein\_coding |
| 4 | 22222499 | 22223210 | 27616 | ENSGALT00000015450 | 4 | ENSGALG00000009489 | 4 | 22250827 | 22516765 | - | FSTL5 | protein\_coding | FSTL5-201 | protein\_coding |
| 4 | 25887286 | 25887901 | 467391 | ENSGALT00000015818 | 4 | ENSGALG00000009719 | 4 | 26355293 | 26387417 | + | PCDH10 | protein\_coding | PCDH10-201 | protein\_coding |
| 4 | 26530893 | 26531990 | 143475 | ENSGALT00000015818 | 4 | ENSGALG00000009719 | 4 | 26355293 | 26387417 | + | PCDH10 | protein\_coding | PCDH10-201 | protein\_coding |
| 4 | 27180311 | 27180810 | 792893 | ENSGALT00000015818 | 4 | ENSGALG00000009719 | 4 | 26355293 | 26387417 | + | PCDH10 | protein\_coding | PCDH10-201 | protein\_coding |
| 4 | 27394572 | 27395212 | 723337 | ENSGALT00000015841 | 4 | ENSGALG00000009732 | 4 | 28118550 | 28128788 | - | PCDH18 | protein\_coding | PCDH18-201 | protein\_coding |
| 4 | 29234085 | 29234741 | 11577 | ENSGALT00000032250 | 3 | ENSGALG00000009865 | 5 | 29246319 | 29359420 | - | RNF150 | protein\_coding | RNF150-201 | protein\_coding |
| 4 | 30595679 | 30596557 | 0 | ENSGALT00000016217 | 4 | ENSGALG00000009977 | 4 | 30583397 | 30608810 | + | SMAD1 | protein\_coding | SMAD1-201 | protein\_coding |
| 4 | 30632716 | 30633375 | 0 | ENSGALT00000016214 | 4 | ENSGALG00000009974 | 4 | 30627025 | 30633032 | + | MMAA | protein\_coding | MMAA-201 | protein\_coding |
| 4 | 35886007 | 35886673 | 20437 | ENSGALT00000042885 | 1 | ENSGALG00000026602 | 1 | 35865270 | 35865569 | - |  | protein\_coding |  | protein\_coding |
| 4 | 40212346 | 40213006 | 397611 | ENSGALT00000017416 | 4 | ENSGALG00000010706 | 4 | 39503734 | 39814734 | - | ODZ3 | protein\_coding | ODZ3-203 | protein\_coding |
| 4 | 48096947 | 48097615 | 306157 | ENSGALT00000018468 | 4 | ENSGALG00000011319 | 4 | 47308221 | 47790789 | + | LPHN3 | protein\_coding | LPHN3-201 | protein\_coding |
| 4 | 55419659 | 55420314 | 298068 | ENSGALT00000019606 | 4 | ENSGALG00000012015 | 4 | 55718383 | 55803843 | + | NDST4 | protein\_coding | NDST4-201 | protein\_coding |
| 4 | 58818773 | 58819243 | 371394 | ENSGALT00000037649 | 2 | ENSGALG00000012219 | 3 | 58208189 | 58447378 | - | UNC5C | protein\_coding | UNC5C-202 | protein\_coding |
| 4 | 62324172 | 62324557 | 46568 | ENSGALT00000022114 | 4 | ENSGALG00000013590 | 4 | 62371126 | 62379523 | + |  | protein\_coding |  | protein\_coding |
| 4 | 62460729 | 62461331 | 6225 | ENSGALT00000022129 | 4 | ENSGALG00000013602 | 4 | 62415272 | 62454503 | - | PCM1 | protein\_coding | PCM1-202 | protein\_coding |
| 4 | 66767916 | 66768002 | 0 | ENSGALT00000022976 | 4 | ENSGALG00000020143 | 3 | 66759878 | 66809013 | + | GABRG1 | protein\_coding | GABRG1-201 | protein\_coding |
| 4 | 68033286 | 68033906 | 14225 | ENSGALT00000037450 | 2 | ENSGALG00000014250 | 4 | 68048132 | 68050245 | + | PHOX2B | protein\_coding | PHOX2B-201 | protein\_coding |
| 4 | 69679128 | 69679960 | 101431 | ENSGALT00000032078 | 3 | ENSGALG00000020139 | 3 | 69781392 | 69796531 | - | DTHD1 | protein\_coding | DTHD1-201 | protein\_coding |
| 4 | 70463742 | 70464439 | 296769 | ENSGALT00000043039 | 1 | ENSGALG00000026486 | 1 | 70157581 | 70166972 | + | ZNF22 | protein\_coding | ZNF22-201 | protein\_coding |
| 4 | 70551829 | 70552669 | 384856 | ENSGALT00000043039 | 1 | ENSGALG00000026486 | 1 | 70157581 | 70166972 | + | ZNF22 | protein\_coding | ZNF22-201 | protein\_coding |
| 4 | 71535538 | 71536391 | 95744 | ENSGALT00000023178 | 4 | ENSGALG00000014349 | 4 | 71188250 | 71439793 | - | PCDH7 | protein\_coding | PCDH7-201 | protein\_coding |
| 4 | 72515182 | 72515626 | 141504 | ENSGALT00000045410 | 1 | ENSGALG00000026918 | 1 | 72373573 | 72373677 | - |  | miRNA |  | miRNA |
| 4 | 72705264 | 72705454 | 0 | ENSGALT00000023186 | 3 | ENSGALG00000014355 | 3 | 72683440 | 72741445 | - | STIM2 | protein\_coding | STIM2-201 | protein\_coding |
| 4 | 75154549 | 75155039 | 246306 | ENSGALT00000028196 | 4 | ENSGALG00000014421 | 4 | 75401346 | 75452145 | + | LCORL | protein\_coding | LCORL-201 | protein\_coding |
| 4 | 78859384 | 78859987 | 11022 | ENSGALT00000025028 | 4 | ENSGALG00000015523 | 4 | 78871010 | 78910488 | - |  | protein\_coding |  | protein\_coding |
| 4 | 78901609 | 78902511 | 0 | ENSGALT00000025028 | 4 | ENSGALG00000015523 | 4 | 78871010 | 78910488 | - |  | protein\_coding |  | protein\_coding |
| 4 | 86220862 | 86221807 | 11607 | ENSGALT00000035352 | 2 | ENSGALG00000021810 | 2 | 85938145 | 86209254 | + |  | protein\_coding |  | protein\_coding |
| 5 | 2705052 | 2706284 | 53760 | ENSGALT00000005949 | 4 | ENSGALG00000003748 | 4 | 2760045 | 2792484 | + | ANO5 | protein\_coding | ANO5-201 | protein\_coding |
| 5 | 4202332 | 4203178 | 179866 | ENSGALT00000019847 | 4 | ENSGALG00000012153 | 4 | 3921314 | 4022465 | + | METTL15 | protein\_coding | METTL15-201 | protein\_coding |
| 5 | 4737109 | 4737429 | 24057 | ENSGALT00000042822 | 1 | ENSGALG00000012136 | 2 | 4595946 | 4713051 | - | MPPED2 | protein\_coding | MPPED2-201 | protein\_coding |
| 5 | 6164269 | 6165523 | 70217 | ENSGALT00000042912 | 1 | ENSGALG00000028923 | 1 | 6235741 | 6237290 | + | C11orf91 | protein\_coding | C11orf91-201 | protein\_coding |
| 5 | 10249946 | 10250591 | 126342 | ENSGALT00000043954 | 1 | ENSGALG00000006059 | 4 | 10010798 | 10123603 | + | INSC | protein\_coding | INSC-203 | protein\_coding |
| 5 | 19126313 | 19126773 | 272913 | ENSGALT00000012899 | 4 | ENSGALG00000007941 | 4 | 18813928 | 18853399 | + | C11orf74 | protein\_coding | C11orf74-201 | protein\_coding |
| 5 | 19302240 | 19303103 | 135245 | ENSGALT00000042056 | 2 | ENSGALG00000025143 | 2 | 19438349 | 19438441 | + |  | miRNA |  | miRNA |
| 5 | 31303866 | 31305068 | 0 | ENSGALT00000015948 | 4 | ENSGALG00000020438 | 3 | 31253451 | 31438936 | + | ATPBD4 | protein\_coding | ATPBD4-201 | protein\_coding |
| 5 | 31525368 | 31526271 | 0 | ENSGALT00000015995 | 4 | ENSGALG00000009838 | 4 | 31481161 | 31528089 | + | AQR | protein\_coding | AQR-201 | protein\_coding |
| 5 | 31898626 | 31899883 | 103055 | ENSGALT00000028640 | 1 | ENSGALG00000017933 | 1 | 32002939 | 32003093 | + | U1 | snRNA | U1-201 | snRNA |
| 5 | 31998141 | 31999034 | 3904 | ENSGALT00000028640 | 1 | ENSGALG00000017933 | 1 | 32002939 | 32003093 | + | U1 | snRNA | U1-201 | snRNA |
| 5 | 39163484 | 39164645 | 0 | ENSGALT00000017123 | 4 | ENSGALG00000010518 | 4 | 38796212 | 39223738 | + | NRXN3 | protein\_coding | NRXN3-201 | protein\_coding |
| 5 | 40463978 | 40464915 | 20135 | ENSGALT00000028641 | 1 | ENSGALG00000017934 | 1 | 40485051 | 40485152 | - | U6 | snRNA | U6-201 | snRNA |
| 5 | 40954947 | 40955227 | 469794 | ENSGALT00000028641 | 1 | ENSGALG00000017934 | 1 | 40485051 | 40485152 | - | U6 | snRNA | U6-201 | snRNA |
| 5 | 41299361 | 41300140 | 160549 | ENSGALT00000017230 | 4 | ENSGALG00000010589 | 4 | 41460690 | 41463889 | + | FLRT2 | protein\_coding | FLRT2-201 | protein\_coding |
| 5 | 55530364 | 55531097 | 32284 | ENSGALT00000042708 | 1 | ENSGALG00000028775 | 1 | 55563382 | 55563503 | + | uc\_338 | misc\_RNA | uc\_338-201 | misc\_RNA |
| 6 | 5291837 | 5292712 | 191655 | ENSGALT00000041089 | 2 | ENSGALG00000024315 | 2 | 5484368 | 5485399 | - |  | protein\_coding |  | protein\_coding |
| 6 | 9811191 | 9816444 | 45176 | ENSGALT00000006047 | 3 | ENSGALG00000003814 | 4 | 9764380 | 9766014 | + | DKK-1 | protein\_coding | DKK-1-201 | protein\_coding |
| 6 | 13724843 | 13726022 | 0 | ENSGALT00000040497 | 2 | ENSGALG00000004990 | 4 | 13474304 | 13762719 | - | C10orf11 | protein\_coding | C10orf11-201 | protein\_coding |
| 6 | 25237263 | 25237909 | 0 | ENSGALT00000013886 | 4 | ENSGALG00000008523 | 4 | 25219899 | 25248494 | - | XPNPEP1 | protein\_coding | XPNPEP1-201 | protein\_coding |
| 6 | 26190297 | 26191167 | 6481 | ENSGALT00000014299 | 3 | ENSGALG00000008795 | 3 | 26158125 | 26183815 | - | GPAM | protein\_coding | GPAM-201 | protein\_coding |
| 6 | 33482245 | 33483060 | 30654 | ENSGALT00000029070 | 3 | ENSGALG00000010454 | 4 | 33513715 | 33654497 | + | MGMT | protein\_coding | MGMT-201 | protein\_coding |
| 6 | 33847042 | 33847694 | 0 | ENSGALT00000017035 | 4 | ENSGALG00000010464 | 4 | 33841581 | 33863088 | + | GLRX3 | protein\_coding | GLRX3-201 | protein\_coding |
| 7 | 2507955 | 2508579 | 0 | ENSGALT00000004407 | 4 | ENSGALG00000002789 | 4 | 2460244 | 2525374 | + | MAP2 | protein\_coding | MAP2-201 | protein\_coding |
| 7 | 8070564 | 8071157 | 186665 | ENSGALT00000012579 | 4 | ENSGALG00000007759 | 4 | 7716439 | 7883898 | - | TMEFF2 | protein\_coding | TMEFF2-201 | protein\_coding |
| 7 | 9022731 | 9023457 | 32796 | ENSGALT00000012618 | 4 | ENSGALG00000007777 | 4 | 9056254 | 9171575 | + | SLC39A10 | protein\_coding | SLC39A10-201 | protein\_coding |
| 7 | 9139826 | 9140674 | 0 | ENSGALT00000012618 | 4 | ENSGALG00000007777 | 4 | 9056254 | 9171575 | + | SLC39A10 | protein\_coding | SLC39A10-201 | protein\_coding |
| 7 | 15504223 | 15504887 | 162224 | ENSGALT00000038715 | 2 | ENSGALG00000009250 | 4 | 15327805 | 15341998 | - | HNRNPA3 | protein\_coding | HNRNPA3-201 | protein\_coding |
| 7 | 17708068 | 17708753 | 0 | ENSGALT00000043162 | 1 | ENSGALG00000009589 | 4 | 17698291 | 17724925 | - | GAD67 | protein\_coding | GAD67-201 | protein\_coding |
| 7 | 26115687 | 26116356 | 0 | ENSGALT00000019105 | 4 | ENSGALG00000011686 | 4 | 26051937 | 26233120 | - | SEMA5B | protein\_coding | SEMA5B-201 | protein\_coding |
| 7 | 29830070 | 29830722 | 0 | ENSGALT00000019898 | 4 | ENSGALG00000012187 | 4 | 29769774 | 29831929 | + | MGAT5 | protein\_coding | MGAT5-201 | protein\_coding |
| 8 | 8899619 | 8900337 | 497890 | ENSGALT00000007757 | 4 | ENSGALG00000004857 | 4 | 8198959 | 8401728 | + | BRINP3 | protein\_coding | BRINP3-201 | protein\_coding |
| 8 | 9208394 | 9209273 | 402427 | ENSGALT00000043816 | 1 | ENSGALG00000028389 | 1 | 9611701 | 9613770 | - |  | protein\_coding |  | protein\_coding |
| 8 | 11644653 | 11645314 | 0 | ENSGALT00000008842 | 4 | ENSGALG00000005509 | 4 | 11522078 | 11839939 | + | DPYD | protein\_coding | DPYD-201 | protein\_coding |
| 8 | 17696796 | 17697651 | 39840 | ENSGALT00000014475 | 4 | ENSGALG00000008902 | 4 | 17737492 | 17759541 | - | GIPC2 | protein\_coding | GIPC2-201 | protein\_coding |
| 8 | 23631239 | 23632111 | 0 | ENSGALT00000017391 | 3 | ENSGALG00000010692 | 4 | 23561280 | 23710577 | - | LRP8 | protein\_coding | LRP8-201 | protein\_coding |
| 9 | 1710352 | 1710900 | 29525 | ENSGALT00000003399 | 3 | ENSGALG00000002169 | 3 | 1740426 | 1751264 | - | PLEKHB2 | protein\_coding | PLEKHB2-201 | protein\_coding |
| 9 | 5602574 | 5603257 | 0 | ENSGALT00000008513 | 4 | ENSGALG00000005310 | 4 | 5566977 | 5861817 | + | CLSTN2 | protein\_coding | CLSTN2-201 | protein\_coding |
| 9 | 18008576 | 18009249 | 17006 | ENSGALT00000039478 | 2 | ENSGALG00000023670 | 2 | 17795977 | 17991569 | - | NAALADL2 | protein\_coding | NAALADL2-201 | protein\_coding |
| 9 | 21346066 | 21346914 | 54112 | ENSGALT00000043367 | 1 | ENSGALG00000026305 | 1 | 21401027 | 21404693 | - | OTOL1 | protein\_coding | OTOL1-201 | protein\_coding |
| AADN03009901.1 | 851 | 1161 |  |  |  |  |  |  |  |  |  |  |  |  |
| JH375157.1 | 356 | 536 |  |  |  |  |  |  |  |  |  |  |  |  |
| JH375968.1 | 8535 | 8818 |  |  |  |  |  |  |  |  |  |  |  |  |
| JH376323.1 | 928 | 1072 | 7117 | ENSGALT00000019766 | 4 | ENSGALG00000012102 | 4 | 8190 | 37322 | + |  | protein\_coding |  | protein\_coding |
| JH376323.1 | 17955 | 18124 | 0 | ENSGALT00000044350 | 1 | ENSGALG00000012102 | 4 | 8190 | 37322 | + |  | protein\_coding |  | protein\_coding |
| Z | 3540391 | 3541231 | 0 | ENSGALT00000045007 | 1 | ENSGALG00000026924 | 1 | 3471917 | 3658223 | + | RIT2 | protein\_coding | RIT2-201 | protein\_coding |
| Z | 15470956 | 15471752 | 158857 | ENSGALT00000032856 | 3 | ENSGALG00000014891 | 4 | 15630610 | 15707360 | + | ITGA1 | protein\_coding | ITGA1-201 | protein\_coding |
| Z | 18807991 | 18809115 | 0 | ENSGALT00000023755 | 3 | ENSGALG00000014732 | 4 | 18788219 | 18829343 | - | ERCC8 | protein\_coding | ERCC8-201 | protein\_coding |
| Z | 30996146 | 31000811 | 36487 | ENSGALT00000045598 | 1 | ENSGALG00000028277 | 1 | 30959484 | 30959658 | + | uc\_338 | misc\_RNA | uc\_338-201 | misc\_RNA |
| Z | 32081154 | 32086293 | 82927 | ENSGALT00000042263 | 1 | ENSGALG00000025350 | 1 | 32169221 | 32169312 | - | gga-mir-1779 | miRNA | gga-mir-1779-201 | miRNA |
| Z | 47227949 | 47228506 | 2233 | ENSGALT00000000344 | 4 | ENSGALG00000000264 | 4 | 47205112 | 47225715 | + |  | protein\_coding |  | protein\_coding |
| Z | 47830813 | 47831469 | 28483 | ENSGALT00000000365 | 4 | ENSGALG00000000276 | 4 | 47510172 | 47802329 | + | FBXL17 | protein\_coding | FBXL17-201 | protein\_coding |
| Z | 49125790 | 49126200 | 94980 | ENSGALT00000045748 | 1 | ENSGALG00000027145 | 1 | 49030729 | 49030809 | + |  | miRNA |  | miRNA |
| Z | 50801143 | 50801818 | 53329 | ENSGALT00000043899 | 1 | ENSGALG00000027849 | 1 | 50620160 | 50747813 | + |  | protein\_coding |  | protein\_coding |
| Z | 50872653 | 50873337 | 124839 | ENSGALT00000043899 | 1 | ENSGALG00000027849 | 1 | 50620160 | 50747813 | + |  | protein\_coding |  | protein\_coding |
| Z | 57973076 | 57973674 | 0 | ENSGALT00000023631 | 4 | ENSGALG00000014661 | 4 | 57916145 | 58180255 | + | FAM172A | protein\_coding | FAM172A-201 | protein\_coding |
| Z | 61304819 | 61306021 | 242003 | ENSGALT00000045264 | 1 | ENSGALG00000027963 | 1 | 61059202 | 61062815 | - | COX7C | protein\_coding | COX7C-201 | protein\_coding |
| Z | 64336432 | 64337248 | 4988 | ENSGALT00000025222 | 3 | ENSGALG00000026946 | 1 | 64342237 | 64344092 | - |  | protein\_coding |  | protein\_coding |
| Z | 64574244 | 64574638 | 22628 | ENSGALT00000042190 | 1 | ENSGALG00000025277 | 1 | 64597267 | 64597386 | + |  | miRNA |  | miRNA |
| Z | 65689607 | 65690259 | 0 | ENSGALT00000025246 | 4 | ENSGALG00000015660 | 4 | 65685568 | 65734943 | - | SNX30 | protein\_coding | SNX30-201 | protein\_coding |
| Z | 69879377 | 69879901 | 279148 | ENSGALT00000034628 | 3 | ENSGALG00000021452 | 3 | 70159050 | 70159861 | - |  | protein\_coding |  | protein\_coding |
| Z | 78844863 | 78846057 | 34354 | ENSGALT00000013404 | 4 | ENSGALG00000008237 | 4 | 78775850 | 78810508 | - | GRAMD3 | protein\_coding | GRAMD3-201 | protein\_coding |
| Z | 79945501 | 79946432 | 2154 | ENSGALT00000044626 | 1 | ENSGALG00000002567 | 3 | 79931937 | 79943346 | + |  | protein\_coding |  | protein\_coding |
| 11 | 7345413 | 7345603 | 5494 | ENSGALT00000007069 | 4 | ENSGALG00000004444 | 4 | 7351098 | 7365112 | + | SHCBP1 | protein\_coding | SHCBP1-201 | protein\_coding |
| 11 | 11174286 | 11174756 | 162817 | ENSGALT00000008459 | 4 | ENSGALG00000005272 | 4 | 10982217 | 11011468 | - | CDH5 | protein\_coding | CDH5-201 | protein\_coding |
| 11 | 18978682 | 18979268 | 0 | ENSGALT00000001009 | 4 | ENSGALG00000000713 | 4 | 18936951 | 19031832 | + | ZFHX3 | protein\_coding | ZFHX3-201 | protein\_coding |
| 15 | 1590630 | 1591272 | 0 | ENSGALT00000003452 | 3 | ENSGALG00000002205 | 3 | 1547558 | 1855522 | - | FBRSL1 | protein\_coding | FBRSL1-201 | protein\_coding |
| 24 | 5789018 | 5789217 | 0 | ENSGALT00000012709 | 4 | ENSGALG00000007833 | 4 | 5786865 | 5800976 | - | TTC12 | protein\_coding | TTC12-201 | protein\_coding |
| 13 | 1868296 | 1868957 | 565 | ENSGALT00000003874 | 4 | ENSGALG00000002457 | 4 | 1869523 | 1957625 | + | SIL1 | protein\_coding | SIL1-201 | protein\_coding |
| 13 | 2970495 | 2971368 | 65060 | ENSGALT00000003332 | 4 | ENSGALG00000002132 | 4 | 2890226 | 2905434 | - | KCNIP1 | protein\_coding | KCNIP1-201 | protein\_coding |
| 13 | 6261859 | 6262487 | 136728 | ENSGALT00000034794 | 3 | ENSGALG00000001706 | 4 | 6399216 | 6470747 | - | GABRG2 | protein\_coding | GABRG2-201 | protein\_coding |
| 13 | 6484735 | 6485598 | 13987 | ENSGALT00000034794 | 3 | ENSGALG00000001706 | 4 | 6399216 | 6470747 | - | GABRG2 | protein\_coding | GABRG2-201 | protein\_coding |
| 13 | 6876434 | 6877337 | 53599 | ENSGALT00000002584 | 4 | ENSGALG00000001690 | 4 | 6690008 | 6822834 | + | GABARB | protein\_coding | GABARB-201 | protein\_coding |
| 13 | 10909806 | 10910958 | 0 | ENSGALT00000006182 | 4 | ENSGALG00000003886 | 4 | 10842858 | 11150740 | - | SGCD | protein\_coding | SGCD-201 | protein\_coding |
| AADN03017712.1 | 157 | 1286 |  |  |  |  |  |  |  |  |  |  |  |  |
| 17 | 180636 | 181570 | 55326 | ENSGALT00000034159 | 3 | ENSGALG00000021211 | 3 | 236897 | 239605 | + | PRF1 | protein\_coding | PRF1-201 | protein\_coding |
| 19 | 2694054 | 2694923 | 0 | ENSGALT00000001925 | 4 | ENSGALG00000021665 | 3 | 2645697 | 2698701 | - | GTF2I | protein\_coding | GTF2I-201 | protein\_coding |
| AADN03018735.1 | 281 | 1293 |  |  |  |  |  |  |  |  |  |  |  |  |
| 14 | 236512 | 236813 | 17560 | ENSGALT00000004615 | 4 | ENSGALG00000002924 | 4 | 141352 | 218951 | + | SNX29 | protein\_coding | SNX29-201 | protein\_coding |
| 14 | 7965871 | 7966753 | 100563 | ENSGALT00000039991 | 2 | ENSGALG00000006757 | 4 | 8067317 | 8147095 | + | XYLT1 | protein\_coding | XYLT1-201 | protein\_coding |
| AADN03024906.1 | 3470 | 4351 | 1021 | ENSGALT00000036188 | 2 | ENSGALG00000022611 | 2 | 5373 | 15649 | - |  | protein\_coding |  | protein\_coding |
| JH375212.1 | 37241 | 37702 | 5714 | ENSGALT00000022685 | 4 | ENSGALG00000014003 | 4 | 9142 | 31526 | + |  | protein\_coding |  | protein\_coding |
| JH375231.1 | 4846 | 8915 |  |  |  |  |  |  |  |  |  |  |  |  |
| JH375237.1 | 49865 | 50701 |  |  |  |  |  |  |  |  |  |  |  |  |
| JH376310.1 | 6513 | 7054 |  |  |  |  |  |  |  |  |  |  |  |  |
| AADN03016632.1 | 765 | 923 | 0 | ENSGALT00000043900 | 1 | ENSGALG00000028058 | 1 | 56 | 1788 | + |  | protein\_coding |  | protein\_coding |
| AADN03019391.1 | 477 | 583 |  |  |  |  |  |  |  |  |  |  |  |  |
| AADN03016003.1 | 257 | 448 |  |  |  |  |  |  |  |  |  |  |  |  |
| AADN03024630.1 | 317 | 887 |  |  |  |  |  |  |  |  |  |  |  |  |
| AADN03025776.1 | 820 | 1020 |  |  |  |  |  |  |  |  |  |  |  |  |
| AADN03018589.1 | 484 | 802 |  |  |  |  |  |  |  |  |  |  |  |  |

  


---

  


# Supplementary Table 7: *S7*

| S7. BLAT results of EAV-HP LTR to Galgal4\* | | | | | | | | | | | | | | | | | |
|  |  |  |  |  |  |  |  |  |  |  |  |  |  |  |  |  |  |
| **matches** | **misMatches** | **repMatches** | **nCount** | **qNumInsert** | **qBaseInsert** | **tNumInsert** | **tBaseInsert** | **strand** | **qName** | **qSize** | **qStart** | **qEnd** | **tName** | **tSize** | **tStart** | **tEnd** | **Detected in RJF [1]** |
| 285 | 2 | 0 | 0 | 0 | 0 | 0 | 0 | + | EAV\_HP\_LTR | 287 | 0 | 287 | 1 | 195276750 | 15902879 | 15904166 | NA |
| 282 | 3 | 0 | 0 | 0 | 0 | 0 | 0 | + | EAV\_HP\_LTR | 287 | 0 | 285 | 1 | 195276750 | 43882599 | 43883884 | 1:43882858-43883632 |
| 285 | 2 | 0 | 0 | 0 | 0 | 0 | 0 | + | EAV\_HP\_LTR | 287 | 0 | 287 | 1 | 195276750 | 96156139 | 96157426 | 1:96156897-96157152 |
| 144 | 2 | 0 | 0 | 0 | 0 | 0 | 0 | - | EAV\_HP\_LTR | 287 | 139 | 285 | 1 | 195276750 | 116918088 | 116919234 | NA |
| 284 | 2 | 0 | 0 | 0 | 0 | 0 | 0 | - | EAV\_HP\_LTR | 287 | 0 | 286 | 1 | 195276750 | 140710334 | 140711620 | 1:140710511-140710872 |
| 286 | 1 | 0 | 0 | 0 | 0 | 0 | 0 | - | EAV\_HP\_LTR | 287 | 0 | 287 | 1 | 195276750 | 140714165 | 140715452 | 1:140714361-140715230 |
| 147 | 1 | 0 | 0 | 0 | 0 | 0 | 0 | + | EAV\_HP\_LTR | 287 | 139 | 287 | 1 | 195276750 | 148564528 | 148565676 | NA |
| 145 | 1 | 0 | 0 | 0 | 0 | 0 | 0 | + | EAV\_HP\_LTR | 287 | 139 | 285 | 1 | 195276750 | 148567522 | 148568668 | 1:148568134-148568461 |
| 283 | 3 | 0 | 0 | 0 | 0 | 0 | 0 | - | EAV\_HP\_LTR | 287 | 0 | 286 | 1 | 195276750 | 149162719 | 149164005 | 1:149162974-149163255 |
| 284 | 3 | 0 | 0 | 0 | 0 | 0 | 0 | - | EAV\_HP\_LTR | 287 | 0 | 287 | 1 | 195276750 | 149164939 | 149166226 | 1:149165178-149166025 |
| 147 | 1 | 0 | 0 | 0 | 0 | 0 | 0 | + | EAV\_HP\_LTR | 287 | 139 | 287 | 1 | 195276750 | 151406450 | 151407598 | NA |
| 145 | 1 | 0 | 0 | 0 | 0 | 0 | 0 | + | EAV\_HP\_LTR | 287 | 139 | 285 | 1 | 195276750 | 151409399 | 151410545 | 1:151410012-151410307 |
| 145 | 1 | 0 | 0 | 0 | 0 | 0 | 0 | - | EAV\_HP\_LTR | 287 | 139 | 285 | 1 | 195276750 | 159293851 | 159294997 | 1:159294092-159294382 |
| 147 | 1 | 0 | 0 | 0 | 0 | 0 | 0 | - | EAV\_HP\_LTR | 287 | 139 | 287 | 1 | 195276750 | 159296850 | 159297998 | NA |
| 144 | 2 | 0 | 0 | 0 | 0 | 0 | 0 | - | EAV\_HP\_LTR | 287 | 139 | 285 | 1 | 195276750 | 160455165 | 160456311 | 1:160455392-160455708 |
| 147 | 1 | 0 | 0 | 0 | 0 | 0 | 0 | - | EAV\_HP\_LTR | 287 | 139 | 287 | 1 | 195276750 | 160458164 | 160459312 | NA |
| 115 | 0 | 0 | 0 | 0 | 0 | 0 | 0 | - | EAV\_HP\_LTR | 287 | 139 | 254 | 1 | 195276750 | 160460150 | 160461265 | NA |
| 146 | 2 | 0 | 0 | 0 | 0 | 0 | 0 | - | EAV\_HP\_LTR | 287 | 139 | 287 | 1 | 195276750 | 160463118 | 160464266 | NA |
| 141 | 7 | 0 | 0 | 0 | 0 | 1 | 1 | - | EAV\_HP\_LTR | 287 | 139 | 287 | 1 | 195276750 | 163499237 | 163500386 | NA |
| 142 | 5 | 0 | 0 | 0 | 0 | 1 | 1 | - | EAV\_HP\_LTR | 287 | 139 | 286 | 1 | 195276750 | 163500690 | 163501838 | NA |
| 147 | 1 | 0 | 0 | 0 | 0 | 0 | 0 | + | EAV\_HP\_LTR | 287 | 139 | 287 | 1 | 195276750 | 163906762 | 163907910 | NA |
| 145 | 1 | 0 | 0 | 0 | 0 | 0 | 0 | + | EAV\_HP\_LTR | 287 | 139 | 285 | 1 | 195276750 | 163909725 | 163910871 | 1:163910330-163910681 |
| 144 | 2 | 0 | 0 | 0 | 0 | 0 | 0 | + | EAV\_HP\_LTR | 287 | 139 | 285 | 1 | 195276750 | 164414954 | 164416100 | 1:164415166-164415884 |
| 145 | 2 | 0 | 0 | 0 | 0 | 0 | 0 | - | EAV\_HP\_LTR | 287 | 139 | 286 | 1 | 195276750 | 180746857 | 180748004 | 1:180747124-180747762 |
| 280 | 6 | 0 | 0 | 0 | 0 | 1 | 1 | - | EAV\_HP\_LTR | 287 | 1 | 287 | 1 | 195276750 | 182832811 | 182834098 | 1:182832996-182833874 |
| 145 | 1 | 0 | 0 | 0 | 0 | 0 | 0 | + | EAV\_HP\_LTR | 287 | 139 | 285 | 2 | 148809762 | 20043579 | 20044725 | 2:20044191-20044405 |
| 145 | 1 | 0 | 0 | 0 | 0 | 0 | 0 | - | EAV\_HP\_LTR | 287 | 139 | 285 | 2 | 148809762 | 21240644 | 21241790 | 2:21240875-21241179 |
| 146 | 1 | 0 | 0 | 0 | 0 | 0 | 0 | - | EAV\_HP\_LTR | 287 | 139 | 286 | 2 | 148809762 | 21243614 | 21244761 | NA |
| 145 | 1 | 0 | 0 | 0 | 0 | 0 | 0 | + | EAV\_HP\_LTR | 287 | 139 | 285 | 2 | 148809762 | 58047034 | 58048180 | 2:58047649-58047977 |
| 285 | 2 | 0 | 0 | 0 | 0 | 0 | 0 | + | EAV\_HP\_LTR | 287 | 0 | 287 | 2 | 148809762 | 81458469 | 81459756 | 2:81458689-81458997 |
| 49 | 2 | 0 | 0 | 0 | 0 | 0 | 0 | + | EAV\_HP\_LTR | 287 | 234 | 285 | 2 | 148809762 | 81461783 | 81462834 | 2:81462137-81462600 |
| 145 | 2 | 0 | 0 | 0 | 0 | 0 | 0 | - | EAV\_HP\_LTR | 287 | 139 | 286 | 2 | 148809762 | 81964951 | 81966098 | 2:81965218-81965871 |
| 283 | 4 | 0 | 0 | 0 | 0 | 0 | 0 | + | EAV\_HP\_LTR | 287 | 0 | 287 | 2 | 148809762 | 82574275 | 82575562 | 2:82574530-82575341 |
| 275 | 8 | 0 | 0 | 0 | 0 | 1 | 1 | - | EAV\_HP\_LTR | 287 | 2 | 285 | 2 | 148809762 | 92856960 | 92858244 | 2:92857191-92857951 |
| 145 | 1 | 0 | 0 | 0 | 0 | 0 | 0 | + | EAV\_HP\_LTR | 287 | 139 | 285 | 2 | 148809762 | 109247987 | 109249133 | NA |
| 145 | 2 | 0 | 0 | 0 | 0 | 0 | 0 | + | EAV\_HP\_LTR | 287 | 139 | 286 | 2 | 148809762 | 115445975 | 115447122 | 2:115446270-115446856 |
| 145 | 1 | 0 | 0 | 0 | 0 | 0 | 0 | - | EAV\_HP\_LTR | 287 | 139 | 285 | 2 | 148809762 | 131563717 | 131564863 | 2:131563946-131564242 |
| 147 | 1 | 0 | 0 | 0 | 0 | 0 | 0 | - | EAV\_HP\_LTR | 287 | 139 | 287 | 2 | 148809762 | 131566678 | 131567826 | NA |
| 144 | 2 | 0 | 0 | 0 | 0 | 0 | 0 | + | EAV\_HP\_LTR | 287 | 139 | 285 | 2 | 148809762 | 142512833 | 142513979 | 2:142513037-142513743 |
| 144 | 2 | 0 | 0 | 0 | 0 | 0 | 0 | - | EAV\_HP\_LTR | 287 | 139 | 285 | 2 | 148809762 | 143463190 | 143464336 | 2:143463458-143463723 |
| 146 | 2 | 0 | 0 | 0 | 0 | 0 | 0 | - | EAV\_HP\_LTR | 287 | 139 | 287 | 2 | 148809762 | 143467768 | 143468916 | NA |
| 277 | 8 | 0 | 0 | 0 | 0 | 1 | 1 | + | EAV\_HP\_LTR | 287 | 0 | 285 | 3 | 110447801 | 19821763 | 19823049 | 3:19822051-19822794 |
| 145 | 1 | 0 | 0 | 0 | 0 | 0 | 0 | - | EAV\_HP\_LTR | 287 | 139 | 285 | 3 | 110447801 | 54250057 | 54251203 | 3:54250303-54250586 |
| 147 | 1 | 0 | 0 | 0 | 0 | 0 | 0 | - | EAV\_HP\_LTR | 287 | 139 | 287 | 3 | 110447801 | 54253057 | 54254205 | NA |
| 284 | 2 | 0 | 0 | 0 | 0 | 0 | 0 | - | EAV\_HP\_LTR | 287 | 0 | 286 | 3 | 110447801 | 55831224 | 55832510 | 3:55831442-55831755 |
| 285 | 2 | 0 | 0 | 0 | 0 | 0 | 0 | - | EAV\_HP\_LTR | 287 | 0 | 287 | 3 | 110447801 | 55835124 | 55836411 | 3:55835868-55836192 |
| 279 | 5 | 0 | 0 | 0 | 0 | 1 | 1 | - | EAV\_HP\_LTR | 287 | 2 | 286 | 3 | 110447801 | 73561835 | 73563120 | 3:73562061-73562884 |
| 128 | 4 | 0 | 0 | 0 | 0 | 1 | 1 | - | EAV\_HP\_LTR | 287 | 139 | 271 | 3 | 110447801 | 83776864 | 83777997 | 3:83777060-83777752 |
| 147 | 1 | 0 | 0 | 0 | 0 | 0 | 0 | + | EAV\_HP\_LTR | 287 | 139 | 287 | 3 | 110447801 | 84889098 | 84890246 | NA |
| 145 | 1 | 0 | 0 | 0 | 0 | 0 | 0 | + | EAV\_HP\_LTR | 287 | 139 | 285 | 3 | 110447801 | 84892061 | 84893207 | 3:84892673-84892945 |
| 145 | 1 | 0 | 0 | 0 | 0 | 0 | 0 | - | EAV\_HP\_LTR | 287 | 139 | 285 | 3 | 110447801 | 96957488 | 96958634 | 3:96957718-96958022 |
| 147 | 1 | 0 | 0 | 0 | 0 | 0 | 0 | - | EAV\_HP\_LTR | 287 | 139 | 287 | 3 | 110447801 | 96960488 | 96961636 | NA |
| 284 | 1 | 0 | 0 | 0 | 0 | 0 | 0 | - | EAV\_HP\_LTR | 287 | 0 | 285 | 4 | 90216835 | 11123151 | 11124436 | 4:11123987-11124226 |
| 141 | 5 | 0 | 0 | 0 | 0 | 1 | 1 | - | EAV\_HP\_LTR | 287 | 139 | 285 | 4 | 90216835 | 26530851 | 26531998 | 4:26531126-26531394 |
| 64 | 0 | 0 | 0 | 0 | 0 | 0 | 0 | - | EAV\_HP\_LTR | 287 | 0 | 64 | 4 | 90216835 | 55419426 | 55420490 | 4:55419659-55420314 |
| 262 | 6 | 0 | 0 | 1 | 16 | 1 | 1 | - | EAV\_HP\_LTR | 287 | 1 | 285 | 5 | 59580361 | 2705027 | 2706296 | 5:2705267-2706061 |
| 147 | 1 | 0 | 0 | 0 | 0 | 0 | 0 | + | EAV\_HP\_LTR | 287 | 139 | 287 | 5 | 59580361 | 4733497 | 4734645 | NA |
| 145 | 1 | 0 | 0 | 0 | 0 | 0 | 0 | + | EAV\_HP\_LTR | 287 | 139 | 285 | 5 | 59580361 | 4736497 | 4737643 | 5:4737109-4737429 |
| 283 | 2 | 0 | 0 | 0 | 0 | 0 | 0 | - | EAV\_HP\_LTR | 287 | 0 | 285 | 5 | 59580361 | 6164228 | 6165513 | 5:6164414-6165265 |
| 261 | 9 | 0 | 0 | 1 | 16 | 2 | 2 | - | EAV\_HP\_LTR | 287 | 1 | 287 | 5 | 59580361 | 31303827 | 31305099 | 5:31304065-31304847 |
| 277 | 8 | 0 | 0 | 0 | 0 | 0 | 0 | - | EAV\_HP\_LTR | 287 | 0 | 285 | 5 | 59580361 | 31898623 | 31899908 | 5:31898868-31899688 |
| 260 | 9 | 0 | 0 | 1 | 16 | 2 | 2 | - | EAV\_HP\_LTR | 287 | 2 | 287 | 5 | 59580361 | 39163416 | 39164687 | 5:39163672-39164482 |
| 277 | 8 | 0 | 0 | 0 | 0 | 1 | 1 | + | EAV\_HP\_LTR | 287 | 2 | 287 | 6 | 34951654 | 9811161 | 9812447 | 6:9811364-9812260 |
| 276 | 7 | 0 | 0 | 0 | 0 | 1 | 1 | + | EAV\_HP\_LTR | 287 | 2 | 285 | 6 | 34951654 | 9815201 | 9816485 | 6:9815557-9816246 |
| 253 | 11 | 0 | 0 | 1 | 16 | 2 | 2 | - | EAV\_HP\_LTR | 287 | 6 | 286 | 6 | 34951654 | 13724803 | 13726069 | 6:13725047-13725814 |
| 146 | 2 | 0 | 0 | 0 | 0 | 0 | 0 | + | EAV\_HP\_LTR | 287 | 139 | 287 | 8 | 28767244 | 8899413 | 8900561 | 8:8899619-8900337 |
| 283 | 2 | 0 | 0 | 0 | 0 | 0 | 0 | - | EAV\_HP\_LTR | 287 | 0 | 285 | 12 | 19897011 | 3277392 | 3278677 | 12:3277795-3278292 |
| 283 | 2 | 0 | 0 | 0 | 0 | 0 | 0 | - | EAV\_HP\_LTR | 287 | 0 | 285 | 12 | 19897011 | 15105969 | 15107254 | 12:15106212-15106979 |
| 282 | 3 | 0 | 0 | 0 | 0 | 0 | 0 | + | EAV\_HP\_LTR | 287 | 0 | 285 | 13 | 17760035 | 10909746 | 10911031 | 13:10909930-10910774 |
| 142 | 5 | 0 | 0 | 0 | 0 | 1 | 1 | + | EAV\_HP\_LTR | 287 | 139 | 286 | AADN03009451.1 | 852 | -1 | 1147 | NA |
| 68 | 1 | 0 | 0 | 0 | 0 | 0 | 0 | + | EAV\_HP\_LTR | 287 | 216 | 285 | AADN03010133.1 | 31673 | -500 | 569 | NA |
| 283 | 2 | 0 | 0 | 0 | 0 | 0 | 0 | - | EAV\_HP\_LTR | 287 | 0 | 285 | AADN03012687.1 | 1295 | 325 | 1610 | NA |
| 147 | 1 | 0 | 0 | 0 | 0 | 0 | 0 | - | EAV\_HP\_LTR | 287 | 139 | 287 | AADN03016226.1 | 1830 | 378 | 1526 | NA |
| 141 | 7 | 0 | 0 | 0 | 0 | 1 | 1 | - | EAV\_HP\_LTR | 287 | 139 | 287 | AADN03016632.1 | 1809 | -41 | 1108 | NA |
| 146 | 2 | 0 | 0 | 0 | 0 | 0 | 0 | + | EAV\_HP\_LTR | 287 | 139 | 287 | AADN03018589.1 | 1026 | -144 | 1004 | NA |
| 275 | 8 | 0 | 0 | 0 | 0 | 1 | 1 | - | EAV\_HP\_LTR | 287 | 2 | 285 | AADN03018735.1 | 1754 | 99 | 1383 | AADN03018735.1:321-1166 |
| 147 | 1 | 0 | 0 | 0 | 0 | 0 | 0 | + | EAV\_HP\_LTR | 287 | 139 | 287 | AADN03019391.1 | 1204 | 270 | 1418 | NA |
| 278 | 7 | 0 | 0 | 0 | 0 | 1 | 1 | + | EAV\_HP\_LTR | 287 | 2 | 287 | AADN03022428.1 | 1093 | -363 | 923 | NA |
| 141 | 7 | 0 | 0 | 0 | 0 | 1 | 1 | + | EAV\_HP\_LTR | 287 | 139 | 287 | AADN03024906.1 | 18168 | 3269 | 4418 | NA |
| 147 | 1 | 0 | 0 | 0 | 0 | 0 | 0 | - | EAV\_HP\_LTR | 287 | 139 | 287 | AADN03025776.1 | 1746 | -81 | 1067 | NA |
| 145 | 1 | 0 | 0 | 0 | 0 | 0 | 0 | + | EAV\_HP\_LTR | 287 | 139 | 285 | AADN03026616.1 | 1372 | 88 | 1234 | NA |
| 146 | 2 | 0 | 0 | 0 | 0 | 0 | 0 | + | EAV\_HP\_LTR | 287 | 139 | 287 | JH375157.1 | 2502 | 204 | 1352 | NA |
| 140 | 6 | 0 | 0 | 0 | 0 | 1 | 1 | + | EAV\_HP\_LTR | 287 | 139 | 285 | JH375212.1 | 52949 | 36637 | 37784 | JH375212.1:37241-37575 |
| 240 | 14 | 0 | 0 | 1 | 17 | 4 | 356 | - | EAV\_HP\_LTR | 287 | 0 | 271 | JH375231.1 | 51492 | 4677 | 6287 | JH375231.1:4893-5626 |
| 247 | 22 | 0 | 0 | 1 | 17 | 3 | 3 | - | EAV\_HP\_LTR | 287 | 1 | 287 | JH375231.1 | 51492 | 7797 | 9069 | JH375231.1:8174-8709 |
| 179 | 15 | 0 | 0 | 3 | 50 | 3 | 51 | - | EAV\_HP\_LTR | 287 | 30 | 274 | JH375237.1 | 53235 | 49664 | 50909 | JH375237.1:49867-50521 |
| 275 | 8 | 0 | 0 | 0 | 0 | 1 | 1 | - | EAV\_HP\_LTR | 287 | 2 | 285 | JH376310.1 | 16848 | 6514 | 7798 | JH376310.1:6805-7054 |
| 188 | 17 | 0 | 0 | 1 | 19 | 3 | 4 | + | EAV\_HP\_LTR | 287 | 30 | 254 | Z | 82363669 | 18807921 | 18809130 | Z:18808285-18808949 |
| 282 | 5 | 0 | 0 | 0 | 0 | 0 | 0 | + | EAV\_HP\_LTR | 287 | 0 | 287 | Z | 82363669 | 30995909 | 30997196 | Z:30996146-30996935 |
| 283 | 4 | 0 | 0 | 0 | 0 | 0 | 0 | + | EAV\_HP\_LTR | 287 | 0 | 287 | Z | 82363669 | 30999756 | 31001043 | Z:31000091-31000811 |
| 285 | 2 | 0 | 0 | 0 | 0 | 0 | 0 | + | EAV\_HP\_LTR | 287 | 0 | 287 | Z | 82363669 | 32081128 | 32082415 | NA |
| 283 | 2 | 0 | 0 | 0 | 0 | 0 | 0 | + | EAV\_HP\_LTR | 287 | 0 | 285 | Z | 82363669 | 32085056 | 32086341 | Z:32085773-32086047 |
| 261 | 7 | 0 | 0 | 1 | 17 | 2 | 2 | + | EAV\_HP\_LTR | 287 | 0 | 285 | Z | 82363669 | 61304789 | 61306059 | Z:61305043-61305820 |
| 284 | 1 | 0 | 0 | 0 | 0 | 0 | 0 | + | EAV\_HP\_LTR | 287 | 0 | 285 | Z | 82363669 | 64336183 | 64337468 | Z:64336432-64337248 |
| 281 | 4 | 0 | 0 | 1 | 2 | 0 | 0 | + | EAV\_HP\_LTR | 287 | 0 | 287 | Z | 82363669 | 78844834 | 78846119 | Z:78845015-78845880 |
|  |  |  |  |  |  |  |  |  |  |  |  |  |  |  |  |  |  |
| \* Alignments from starting at 140 bp could potentially be ART-CH and not EAV-HP as the LTR sequence is near identical from 140 onwards | | | | | | | | | | | | | | | | |  |
| BLAT alignment performed using standalone BLAT, minScore 20 | | | | | | | | | | | | | | | | |  |
| [1] Relates to whether or not the BLAT hit was detected as an interval in the RJF sample (MQ=20, RC=0.25μXi) | | | | | | | | | | | | | | | | | |

  


---

  


# Supplementary Table 8a: *S8a*

| S8a. Intervals at high frequency (≥0.9) across all birds/lines | | | | | | | | | | | | | | | |
|  |  |  |  |  |  |  |  |  |  |  |  |  |  |  |  |
|  |  |  |  | **BLAT: EAV-HP LTR to Galgal4** | | **BLAT: EAV-HP genome (GenBank:KC632578) to Galgal4** | | | | | | | | | |
| **Interval** | **Frequency** | **Absent [1]** | **Breakpoints [2]** | **Identity** | **Span** | **Genomic Location** | **Orientation** | **Query name** | **Query start** | **Query end** | **Query ori** | **Length** | **Score** | **E-val** | **%ID** |
| 1:140710345-140715423 | 1 |  | 1:140710834 | 0.99 | 0-286 | 1:140710835-140711148 | Reverse | KC632578 | 1 | 314 | Forward | 314 | 608 | 2.2E-174 | 99.04 |
| 1:182832847-182834082 | 0.91 | LineN, Wellcome | 1:182833598 1:182833313 | 0.98 | 1-287 | 1:182833314-182833598 | Reverse | KC632578 | 30 | 313 | Forward | 285 | 532 | 2.8E-151 | 97.54 |
| 2:82574297-82575518 | 0.91 | JB1B16A, Silkie | 2:82574775 | 0.99 | 0-287 | 2:82574748-82575060 | Forward | KC632578 | 1 | 313 | Forward | 313 | 604 | 5.2E-173 | 98.72 |
| 2:92856996-92858219 | 0.96 | HB1A16A | 2:92857744 | 0.96 | 2-285 | 2:92857461-92857744 | Reverse | KC632578 | 31 | 313 | Forward | 284 | 520 | 1.2E-147 | 96.48 |
| 3:19821802-19823007 | 1 |  | 3:19822549 | 0.97 | 0-285 | 3:19822266-19822549 | Forward | KC632578 | 31 | 313 | Forward | 284 | 524 | 5.5E-149 | 96.83 |
| 3:73561879-73563101 | 0.96 | Silkie | 3:73562620 | 0.97 | 2-286 | 3:73562336-73562620 | Reverse | KC632578 | 31 | 314 | Forward | 285 | 529 | 1.4E-150 | 97.54 |
| 5:2705052-2706284 | 1 |  | 5:2705527 5:2705796 | 0.91 | 1-285 | 5:2705528-2705738 | Reverse | KC632578 | 103 | 313 | Forward | 211 | 395 | 5.1E-110 | 97.16 |
| 5:31303866-31305068 | 1 |  | 5:31304327 5:31304599 | 0.91 | 1-287 | 5:31304328-31304464 | Reverse | KC632578 | 179 | 315 | Forward | 137 | 250 | 1.9E-066 | 94.89 |
| 5:31898626-31899883 | 1 |  | 5:31899123 5:31899406 | 0.97 | 0-285 | 5:31899124-31899406 | Reverse | KC632578 | 31 | 313 | Forward | 283 | 535 | 2.9E-152 | 97.17 |
| 6:9811191-9816444 | 1 |  | 6:9811661 | 0.97 | 2-287 | 6:9811662-9811708 | Forward | KC632578 | 31 | 77 | Forward | 47 | 88 | 7.1E-018 | 97.87 |
| 6:13724843-13726022 | 1 |  | 6:13725303 6:13725569 | 0.88 | 6-286 | 6:13725304-13725516 | Reverse | KC632578 | 103 | 314 | Forward | 213 | 380 | 1.3E-105 | 95.31 |
| Z:32081154-32086293 | 0.96 | Line15 | Z:32081628 | 0.99 | 0-287 | Z:32081601-32081790 | Forward | KC632578 | 3926 | 4115 | Forward | 190 | 371 | 4.7E-103 | 100 |
| Z:61304819-61306021 | 1 |  | Z:61305289 Z:61305559 | 0.91 | 0-285 | Z:61305340-61305559 | Forward | KC632578 | 94 | 313 | Forward | 220 | 403 | 1.2E-112 | 96.36 |
| JH376310.1:6513-7054 | 0.9 | Taiwanese, NA=12 | JH376310.1:7014 | 0.96 | 2-285 | JH376310.1:7015-7298 | Reverse | KC632578 | 31 | 313 | Forward | 284 | 521 | 3.1E-148 | 96.48 |
|  |  |  |  |  |  |  |  |  |  |  |  |  |  |  |  |
| [1] NA indicates number of birds/lines for which the presence of the interval was not determined due to lack of coverage | | | | | | | | | | | | | | | |
| [2] Breakpoints observed in soft-clipping data | | | | | | | | | | | | | | | |
|  |  |  |  |  |  |  |  |  |  |  |  |  |  |  |  |
|  |  |  |  |  |  |  |  |  |  |  |  |  |  |  |  |

  


---

  


# Supplementary Table 8b: *S8b*

| S8b. BLAT results of EAV-HP (GenBank:KC632578) to Galgal4 | | | | | | | | | | |  |  |  |  |  |
|  |  |  |  |  |  |  |  |  |  |  |  |  |  |  |  |
| **Genomic Location** | **Orientation** | **Query name** | **Query start** | **Query end** | **Query ori** | **Length** | **Score** | **E-val** | **%ID** | **Interval [1]** |  |  |  |  |  |
| 3:55831849-55835134 | Reverse | KC632578 | 830 | 4115 | Forward | 3286 | 6297 | 0 | 98.54 | - |  |  |  |  |  |
| Z:32082494-32085718 | Forward | KC632578 | 891 | 4115 | Forward | 3225 | 6214 | 0 | 98.85 | Y |  |  |  |  |  |
| 2:81459732-81462282 | Forward | KC632578 | 827 | 3377 | Forward | 2551 | 4874 | 0 | 98.31 | - |  |  |  |  |  |
| 6:9812439-9814532 | Forward | KC632578 | 855 | 2948 | Forward | 2094 | 3934 | 0 | 97.28 | Y |  |  |  |  |  |
| Z:30997187-30998869 | Forward | KC632578 | 794 | 2476 | Forward | 1683 | 3199 | 0 | 97.8 | - |  |  |  |  |  |
| JH376310.1:8793-10459 | Reverse | KC632578 | 817 | 2483 | Forward | 1667 | 3149 | 0 | 97.36 | - |  |  |  |  |  |
| 1:140712534-140714188 | Reverse | KC632578 | 829 | 2483 | Forward | 1655 | 3115 | 0 | 97.16 | Y |  |  |  |  |  |
| 1:149163344-149164982 | Reverse | KC632578 | 2477 | 4115 | Forward | 1639 | 3150 | 0 | 99.02 | - |  |  |  |  |  |
| 1:96155163-96156801 | Forward | KC632578 | 2477 | 4115 | Forward | 1639 | 3128 | 0 | 98.54 | - |  |  |  |  |  |
| Z:30998870-31000418 | Forward | KC632578 | 2567 | 4115 | Forward | 1549 | 2952 | 0 | 98.39 | - |  |  |  |  |  |
| 1:15904233-15905709 | Forward | KC632578 | 891 | 2366 | Forward | 1477 | 2839 | 0 | 98.65 | - |  |  |  |  |  |
| JH376310.1:7543-8775 | Reverse | KC632578 | 2558 | 3790 | Forward | 1233 | 2348 | 0 | 98.38 | - |  |  |  |  |  |
| JH376310.1:7543-8775 | Reverse | KC632578 | 2558 | 3790 | Forward | 1233 | 2348 | 0 | 98.38 | - |  |  |  |  |  |
| AADN03018457.1:1-1150 | Reverse | KC632578 | 841 | 1992 | Forward | 1152 | 2077 | 0 | 94.79 | - |  |  |  |  |  |
| JH375231.1:5430-6548 | Reverse | KC632578 | 2810 | 3928 | Forward | 1119 | 1966 | 0 | 93.3 | - |  |  |  |  |  |
| 6:9814638-9815733 | Forward | KC632578 | 2862 | 3957 | Forward | 1096 | 2064 | 0 | 97.72 | Y |  |  |  |  |  |
| 8:23383004-23384098 | Reverse | KC632578 | 1386 | 2480 | Forward | 1095 | 1838 | 0 | 90.23 | - |  |  |  |  |  |
| JH375237.1:50954-51957 | Reverse | KC632578 | 1156 | 2159 | Forward | 1004 | 1811 | 0 | 94.42 | - |  |  |  |  |  |
| JH375231.1:6570-7511 | Reverse | KC632578 | 1199 | 2140 | Forward | 942 | 1708 | 0 | 94.8 | - |  |  |  |  |  |
| AADN03018735.1:852-1754 | Reverse | KC632578 | 3055 | 3957 | Forward | 903 | 1712 | 0 | 98.01 | - |  |  |  |  |  |
| AADN03022428.1:138-937 | Forward | KC632578 | 31 | 829 | Forward | 800 | 1500 | 0 | 97.25 | - |  |  |  |  |  |
| Z:30996382-30997174 | Forward | KC632578 | 1 | 793 | Forward | 793 | 1528 | 0 | 98.74 | - |  |  |  |  |  |
| 6:9811710-9812438 | Forward | KC632578 | 78 | 806 | Forward | 729 | 1367 | 0 | 96.98 | Y |  |  |  |  |  |
| AADN03016632.1:1-608 | Reverse | KC632578 | 168 | 774 | Forward | 608 | 1126 | 0 | 96.71 | - |  |  |  |  |  |
| 6:22476446-22477011 | Reverse | KC632578 | 2598 | 3163 | Forward | 566 | 923 | 3.7E-269 | 88.87 | - |  |  |  |  |  |
| 1:163499335-163499886 | Reverse | KC632578 | 168 | 718 | Forward | 552 | 1029 | 6.8E-301 | 96.74 | - |  |  |  |  |  |
| 1:148565029-148565579 | Forward | KC632578 | 168 | 718 | Forward | 551 | 1068 | 8.6e-313 | 98.91 | - |  |  |  |  |  |
| 1:159296948-159297498 | Reverse | KC632578 | 168 | 718 | Forward | 551 | 1063 | 3.1e-311 | 98.55 | - |  |  |  |  |  |
| 1:160458262-160458812 | Reverse | KC632578 | 168 | 718 | Forward | 551 | 1065 | 5.5e-312 | 98.73 | - |  |  |  |  |  |
| 1:160463216-160463766 | Reverse | KC632578 | 168 | 718 | Forward | 551 | 1055 | 5.6e-309 | 98 | - |  |  |  |  |  |
| 1:163907263-163907813 | Forward | KC632578 | 168 | 718 | Forward | 551 | 1056 | 3.7e-309 | 98.19 | - |  |  |  |  |  |
| 2:131566776-131567326 | Reverse | KC632578 | 168 | 718 | Forward | 551 | 1060 | 1.8e-310 | 98.37 | - |  |  |  |  |  |
| 2:21243712-21244261 | Reverse | KC632578 | 168 | 718 | Forward | 551 | 1055 | 1.0e-308 | 98.55 | - |  |  |  |  |  |
| 3:54253155-54253705 | Reverse | KC632578 | 168 | 718 | Forward | 551 | 1066 | 4.8e-312 | 98.73 | - |  |  |  |  |  |
| 3:84889599-84890149 | Forward | KC632578 | 168 | 718 | Forward | 551 | 1056 | 4.2e-309 | 98.19 | - |  |  |  |  |  |
| 3:96960586-96961136 | Reverse | KC632578 | 168 | 718 | Forward | 551 | 1054 | 1.8e-308 | 98.19 | - |  |  |  |  |  |
| 5:4733998-4734548 | Forward | KC632578 | 168 | 718 | Forward | 551 | 1054 | 1.8e-308 | 98.19 | - |  |  |  |  |  |
| AADN03016226.1:476-1026 | Reverse | KC632578 | 168 | 718 | Forward | 551 | 1058 | 1.3e-309 | 98.19 | - |  |  |  |  |  |
| AADN03025776.1:41-567 | Reverse | KC632578 | 168 | 694 | Forward | 527 | 1018 | 9.9E-298 | 98.67 | - |  |  |  |  |  |
| 8:23384109-23384630 | Reverse | KC632578 | 854 | 1375 | Forward | 522 | 847 | 2.6E-246 | 88.7 | - |  |  |  |  |  |
| 1:149165272-149165754 | Reverse | KC632578 | 1 | 483 | Forward | 483 | 932 | 1E-271 | 98.76 | - |  |  |  |  |  |
| Z:78845307-78845748 | Forward | KC632578 | 1 | 444 | Forward | 444 | 844 | 2.6E-245 | 98.2 | - |  |  |  |  |  |
| AADN03019391.1:771-1204 | Forward | KC632578 | 168 | 601 | Forward | 434 | 840 | 4E-244 | 98.85 | - |  |  |  |  |  |
| 4:22223302-22223704 | Forward | KC632578 | 2600 | 3002 | Forward | 403 | 592 | 1.5E-169 | 84.12 | - |  |  |  |  |  |
| 2:55802775-55803150 | Reverse | KC632578 | 2598 | 2973 | Forward | 377 | 535 | 1.9E-152 | 83.29 | - |  |  |  |  |  |
| 8:23384700-23385072 | Reverse | KC632578 | 364 | 736 | Forward | 373 | 618 | 3.8E-177 | 89.54 | - |  |  |  |  |  |
| JH375231.1:7872-8236 | Reverse | KC632578 | 427 | 791 | Forward | 365 | 656 | 1E-188 | 94.25 | - |  |  |  |  |  |
| 8:23382023-23382384 | Reverse | KC632578 | 3450 | 3811 | Forward | 362 | 538 | 2.6E-153 | 84.81 | - |  |  |  |  |  |
| JH375231.1:7513-7870 | Reverse | KC632578 | 841 | 1198 | Forward | 358 | 652 | 1.7E-187 | 94.69 | - |  |  |  |  |  |
| 4:23249754-23250109 | Reverse | KC632578 | 2598 | 2953 | Forward | 356 | 551 | 4.7E-157 | 86.8 | - |  |  |  |  |  |
| 8:23382385-23382740 | Reverse | KC632578 | 3091 | 3446 | Forward | 356 | 557 | 4.6E-159 | 86.8 | - |  |  |  |  |  |
| JH375964.1:3390-3740 | Reverse | KC632578 | 2598 | 2948 | Forward | 351 | 514 | 7.6E-146 | 83.19 | - |  |  |  |  |  |
| Z:17371134-17371484 | Reverse | KC632578 | 2598 | 2948 | Forward | 351 | 512 | 2.1E-145 | 83.48 | - |  |  |  |  |  |
| AADN03012687.1:949-1295 | Reverse | KC632578 | 3769 | 4115 | Forward | 347 | 662 | 1.7E-190 | 98.56 | - |  |  |  |  |  |
| JH375237.1:50435-50775 | Reverse | KC632578 | 3588 | 3928 | Forward | 341 | 570 | 9.7E-163 | 90.91 | - |  |  |  |  |  |
| 2:143468080-143468416 | Reverse | KC632578 | 168 | 505 | Forward | 338 | 648 | 2.6E-186 | 98.82 | - |  |  |  |  |  |
| Z:31000229-31000561 | Forward | KC632578 | 1 | 333 | Forward | 333 | 637 | 4.5E-183 | 98.5 | - |  |  |  |  |  |
| 1:96156612-96156929 | Forward | KC632578 | 1 | 321 | Forward | 321 | 600 | 7.1E-172 | 97.82 | - |  |  |  |  |  |
| 1:140710835-140711148 | Reverse | KC632578 | 1 | 314 | Forward | 314 | 608 | 2.2E-174 | 99.04 | Y |  |  |  |  |  |
| 1:149163220-149163533 | Reverse | KC632578 | 1 | 314 | Forward | 314 | 600 | 1E-171 | 98.41 | - |  |  |  |  |  |
| 3:55831725-55832038 | Reverse | KC632578 | 1 | 314 | Forward | 314 | 614 | 6.1E-176 | 99.68 | - |  |  |  |  |  |
| 12:15106470-15106782 | Reverse | KC632578 | 1 | 313 | Forward | 313 | 605 | 2.6E-173 | 99.04 | - |  |  |  |  |  |
| 12:3277893-3278205 | Reverse | KC632578 | 1 | 313 | Forward | 313 | 607 | 7.2E-174 | 99.04 | - |  |  |  |  |  |
| 13:10910219-10910531 | Forward | KC632578 | 1 | 313 | Forward | 313 | 610 | 7.3E-175 | 99.68 | - |  |  |  |  |  |
| 1:43883072-43883384 | Forward | KC632578 | 1 | 313 | Forward | 313 | 602 | 2.3E-172 | 98.72 | - |  |  |  |  |  |
| 2:82574748-82575060 | Forward | KC632578 | 1 | 313 | Forward | 313 | 604 | 5.2E-173 | 98.72 | Y |  |  |  |  |  |
| 4:11123652-11123964 | Reverse | KC632578 | 1 | 313 | Forward | 313 | 609 | 1.3E-174 | 99.36 | - |  |  |  |  |  |
| 5:6164729-6165041 | Reverse | KC632578 | 1 | 313 | Forward | 313 | 609 | 1.3E-174 | 99.36 | - |  |  |  |  |  |
| AADN03012687.1:826-1138 | Reverse | KC632578 | 1 | 313 | Forward | 313 | 598 | 3.6E-171 | 98.4 | - |  |  |  |  |  |
| Z:32085529-32085841 | Forward | KC632578 | 1 | 313 | Forward | 313 | 612 | 2.2E-175 | 99.68 | Y |  |  |  |  |  |
| Z:64336656-64336968 | Forward | KC632578 | 1 | 313 | Forward | 313 | 605 | 3.1E-173 | 99.04 | - |  |  |  |  |  |
| AADN03025131.1:3752-4056 | Forward | KC632578 | 2598 | 2902 | Forward | 305 | 484 | 5.7E-137 | 87.54 | - |  |  |  |  |  |
| 4:11123775-11124072 | Reverse | KC632578 | 3818 | 4115 | Forward | 298 | 573 | 1.2E-163 | 98.99 | - |  |  |  |  |  |
| AADN03018735.1:594-883 | Reverse | KC632578 | 31 | 325 | Forward | 296 | 520 | 1E-147 | 94.59 | - |  |  |  |  |  |
| 1:182833314-182833598 | Reverse | KC632578 | 30 | 313 | Forward | 285 | 532 | 2.8E-151 | 97.54 | Y |  |  |  |  |  |
| 3:73562336-73562620 | Reverse | KC632578 | 31 | 314 | Forward | 285 | 529 | 1.4E-150 | 97.54 | Y |  |  |  |  |  |
| 2:92857461-92857744 | Reverse | KC632578 | 31 | 313 | Forward | 284 | 520 | 1.2E-147 | 96.48 | Y |  |  |  |  |  |
| 3:19822266-19822549 | Forward | KC632578 | 31 | 313 | Forward | 284 | 524 | 5.5E-149 | 96.83 | Y |  |  |  |  |  |
| AADN03022602.1:689-972 | Reverse | KC632578 | 2619 | 2902 | Forward | 284 | 427 | 6.6E-120 | 84.86 | - |  |  |  |  |  |
| JH376310.1:7015-7298 | Reverse | KC632578 | 31 | 313 | Forward | 284 | 521 | 3.1E-148 | 96.48 | Y |  |  |  |  |  |
| 5:31899124-31899406 | Reverse | KC632578 | 31 | 313 | Forward | 283 | 535 | 2.9E-152 | 97.17 | Y |  |  |  |  |  |
| 1:151407170-151407451 | Forward | KC632578 | 437 | 718 | Forward | 282 | 529 | 1.3E-150 | 97.16 | - |  |  |  |  |  |
| JH376310.1:2729-3006 | Reverse | KC632578 | 2598 | 2875 | Forward | 278 | 447 | 5.9E-126 | 88.13 | - |  |  |  |  |  |
| 6:9815750-9815985 | Forward | KC632578 | 78 | 313 | Forward | 236 | 446 | 1.8E-125 | 97.88 | Y |  |  |  |  |  |
| JH376310.1:10461-10687 | Reverse | KC632578 | 565 | 791 | Forward | 227 | 425 | 4.1E-119 | 96.92 | - |  |  |  |  |  |
| Z:61305340-61305559 | Forward | KC632578 | 94 | 313 | Forward | 220 | 403 | 1.2E-112 | 96.36 | Y |  |  |  |  |  |
| 6:13725304-13725516 | Reverse | KC632578 | 103 | 314 | Forward | 213 | 380 | 1.3E-105 | 95.31 | Y |  |  |  |  |  |
| 5:2705528-2705738 | Reverse | KC632578 | 103 | 313 | Forward | 211 | 395 | 5.1E-110 | 97.16 | Y |  |  |  |  |  |
| JH375231.1:5178-5375 | Reverse | KC632578 | 103 | 299 | Forward | 198 | 348 | 5.4E-096 | 94.44 | - |  |  |  |  |  |
| JH375231.1:8237-8431 | Reverse | KC632578 | 182 | 376 | Forward | 195 | 353 | 1.2E-097 | 94.36 | - |  |  |  |  |  |
| 1:15903350-15903541 | Forward | KC632578 | 3924 | 4115 | Forward | 192 | 363 | 1.3E-100 | 97.92 | - |  |  |  |  |  |
| 12:15106593-15106782 | Reverse | KC632578 | 3926 | 4115 | Forward | 190 | 365 | 5.5E-101 | 98.95 | - |  |  |  |  |  |
| 12:3278016-3278205 | Reverse | KC632578 | 3926 | 4115 | Forward | 190 | 369 | 2.6E-102 | 99.47 | - |  |  |  |  |  |
| 13:10910219-10910408 | Forward | KC632578 | 3926 | 4115 | Forward | 190 | 367 | 8.6E-102 | 99.47 | - |  |  |  |  |  |
| 1:140714791-140714980 | Reverse | KC632578 | 3926 | 4115 | Forward | 190 | 369 | 2.6E-102 | 99.47 | Y |  |  |  |  |  |
| 1:149165565-149165754 | Reverse | KC632578 | 3926 | 4115 | Forward | 190 | 365 | 4.9E-101 | 98.95 | - |  |  |  |  |  |
| 1:43883072-43883261 | Forward | KC632578 | 3926 | 4115 | Forward | 190 | 362 | 4.2E-100 | 98.42 | - |  |  |  |  |  |
| 2:81458942-81459131 | Forward | KC632578 | 3926 | 4115 | Forward | 190 | 371 | 4.7E-103 | 100 | - |  |  |  |  |  |
| 2:82574748-82574937 | Forward | KC632578 | 3926 | 4115 | Forward | 190 | 366 | 1.7E-101 | 98.95 | Y |  |  |  |  |  |
| 3:55835750-55835939 | Reverse | KC632578 | 3926 | 4115 | Forward | 190 | 371 | 4.7E-103 | 100 | - |  |  |  |  |  |
| 5:6164852-6165041 | Reverse | KC632578 | 3926 | 4115 | Forward | 190 | 369 | 2.6E-102 | 99.47 | - |  |  |  |  |  |
| Z:30996382-30996571 | Forward | KC632578 | 3926 | 4115 | Forward | 190 | 362 | 3.6E-100 | 98.42 | - |  |  |  |  |  |
| Z:32081601-32081790 | Forward | KC632578 | 3926 | 4115 | Forward | 190 | 371 | 4.7E-103 | 100 | Y |  |  |  |  |  |
| Z:64336656-64336845 | Forward | KC632578 | 3926 | 4115 | Forward | 190 | 364 | 6.5E-101 | 98.95 | - |  |  |  |  |  |
| Z:78845307-78845494 | Forward | KC632578 | 3926 | 4115 | Forward | 190 | 351 | 5.8E-097 | 97.89 | - |  |  |  |  |  |
| AADN03011164.1:16670-16852 | Forward | KC632578 | 2598 | 2780 | Forward | 183 | 314 | 7.6E-086 | 91.26 | - |  |  |  |  |  |
| Z:18808450-18808630 | Forward | KC632578 | 103 | 282 | Forward | 181 | 295 | 5.1E-080 | 90.61 | - |  |  |  |  |  |
| JH375237.1:50778-50950 | Reverse | KC632578 | 3414 | 3586 | Forward | 173 | 313 | 1.3E-085 | 94.8 | - |  |  |  |  |  |
| JH376310.1:7267-7433 | Reverse | KC632578 | 3791 | 3957 | Forward | 167 | 304 | 1.2E-082 | 96.41 | - |  |  |  |  |  |
| 1:182833437-182833598 | Reverse | KC632578 | 3955 | 4115 | Forward | 162 | 296 | 1.9E-080 | 97.53 | Y |  |  |  |  |  |
| 2:92857584-92857744 | Reverse | KC632578 | 3956 | 4115 | Forward | 161 | 292 | 4.9E-079 | 96.89 | Y |  |  |  |  |  |
| 3:19822266-19822426 | Forward | KC632578 | 3956 | 4115 | Forward | 161 | 289 | 3.2E-078 | 96.27 | Y |  |  |  |  |  |
| 3:73562460-73562620 | Reverse | KC632578 | 3956 | 4115 | Forward | 161 | 290 | 1.6E-078 | 96.89 | Y |  |  |  |  |  |
| AADN03022428.1:138-298 | Forward | KC632578 | 3956 | 4115 | Forward | 161 | 292 | 4.9E-079 | 96.89 | - |  |  |  |  |  |
| 5:31899247-31899406 | Reverse | KC632578 | 3956 | 4115 | Forward | 160 | 305 | 5.2E-083 | 98.12 | Y |  |  |  |  |  |
| AADN03022428.1:938-1093 | Forward | KC632578 | 854 | 1010 | Forward | 157 | 293 | 2.4E-079 | 98.09 | - |  |  |  |  |  |
| 1:163501191-163501338 | Reverse | KC632578 | 168 | 314 | Forward | 148 | 266 | 3.4E-071 | 95.95 | - |  |  |  |  |  |
| AADN03009451.1:500-647 | Forward | KC632578 | 168 | 314 | Forward | 148 | 266 | 3.4E-071 | 95.95 | - |  |  |  |  |  |
| JH375157.1:705-852 | Forward | KC632578 | 168 | 315 | Forward | 148 | 286 | 2.1E-077 | 98.65 | - |  |  |  |  |  |
| 1:180747358-180747504 | Reverse | KC632578 | 168 | 314 | Forward | 147 | 282 | 3.2E-076 | 98.64 | - |  |  |  |  |  |
| 2:115446476-115446622 | Forward | KC632578 | 168 | 314 | Forward | 147 | 287 | 1.5E-077 | 99.32 | - |  |  |  |  |  |
| 2:81965452-81965598 | Reverse | KC632578 | 168 | 314 | Forward | 147 | 282 | 2.8E-076 | 98.64 | - |  |  |  |  |  |
| 4:26531352-26531498 | Reverse | KC632578 | 168 | 313 | Forward | 147 | 264 | 1.3E-070 | 95.92 | - |  |  |  |  |  |
| AADN03024906.1:3770-3916 | Forward | KC632578 | 168 | 313 | Forward | 147 | 261 | 8.1E-070 | 95.24 | - |  |  |  |  |  |
| JH375212.1:37138-37284 | Forward | KC632578 | 168 | 313 | Forward | 147 | 261 | 8.1E-070 | 95.24 | - |  |  |  |  |  |
| 1:116918589-116918734 | Reverse | KC632578 | 168 | 313 | Forward | 146 | 282 | 3.1E-076 | 98.63 | - |  |  |  |  |  |
| 1:148568023-148568168 | Forward | KC632578 | 168 | 313 | Forward | 146 | 287 | 9.7E-078 | 100 | - |  |  |  |  |  |
| 1:151409900-151410045 | Forward | KC632578 | 168 | 313 | Forward | 146 | 285 | 5.5E-077 | 99.32 | - |  |  |  |  |  |
| 1:159294352-159294497 | Reverse | KC632578 | 168 | 313 | Forward | 146 | 285 | 5.5E-077 | 99.32 | - |  |  |  |  |  |
| 1:160455666-160455811 | Reverse | KC632578 | 168 | 313 | Forward | 146 | 282 | 3.1E-076 | 98.63 | - |  |  |  |  |  |
| 1:163910226-163910371 | Forward | KC632578 | 168 | 313 | Forward | 146 | 285 | 5.5E-077 | 99.32 | - |  |  |  |  |  |
| 1:164415455-164415600 | Forward | KC632578 | 168 | 313 | Forward | 146 | 282 | 3.6E-076 | 98.63 | - |  |  |  |  |  |
| 2:109248488-109248633 | Forward | KC632578 | 168 | 313 | Forward | 146 | 285 | 5.5E-077 | 99.32 | - |  |  |  |  |  |
| 2:131564218-131564363 | Reverse | KC632578 | 168 | 313 | Forward | 146 | 285 | 5.5E-077 | 99.32 | - |  |  |  |  |  |
| 2:142513334-142513479 | Forward | KC632578 | 168 | 313 | Forward | 146 | 281 | 1E-075 | 98.63 | - |  |  |  |  |  |
| 2:143463691-143463836 | Reverse | KC632578 | 168 | 313 | Forward | 146 | 282 | 3.1E-076 | 98.63 | - |  |  |  |  |  |
| 2:20044080-20044225 | Forward | KC632578 | 168 | 313 | Forward | 146 | 285 | 5.5E-077 | 99.32 | - |  |  |  |  |  |
| 2:21241145-21241290 | Reverse | KC632578 | 168 | 313 | Forward | 146 | 285 | 5.5E-077 | 99.32 | - |  |  |  |  |  |
| 2:58047535-58047680 | Forward | KC632578 | 168 | 313 | Forward | 146 | 285 | 5.5E-077 | 99.32 | - |  |  |  |  |  |
| 3:54250558-54250703 | Reverse | KC632578 | 168 | 313 | Forward | 146 | 285 | 5.5E-077 | 99.32 | - |  |  |  |  |  |
| 3:84892562-84892707 | Forward | KC632578 | 168 | 313 | Forward | 146 | 285 | 5.5E-077 | 99.32 | - |  |  |  |  |  |
| 3:96957989-96958134 | Reverse | KC632578 | 168 | 313 | Forward | 146 | 287 | 9.7E-078 | 100 | - |  |  |  |  |  |
| 5:4736998-4737143 | Forward | KC632578 | 168 | 313 | Forward | 146 | 287 | 9.7E-078 | 100 | - |  |  |  |  |  |
| 8:8899914-8900059 | Forward | KC632578 | 168 | 313 | Forward | 146 | 285 | 5.5E-077 | 99.32 | - |  |  |  |  |  |
| AADN03018589.1:357-502 | Forward | KC632578 | 168 | 313 | Forward | 146 | 285 | 5.5E-077 | 99.32 | - |  |  |  |  |  |
| AADN03026616.1:589-734 | Forward | KC632578 | 168 | 313 | Forward | 146 | 287 | 9.7E-078 | 100 | - |  |  |  |  |  |
| 1:152943120-152943263 | Reverse | KC632578 | 2600 | 2743 | Forward | 144 | 237 | 1.7E-062 | 88.89 | - |  |  |  |  |  |
| 1:152942962-152943102 | Reverse | KC632578 | 2762 | 2902 | Forward | 141 | 202 | 4.3E-052 | 82.98 | - |  |  |  |  |  |
| 5:31304328-31304464 | Reverse | KC632578 | 179 | 315 | Forward | 137 | 250 | 1.9E-066 | 94.89 | Y |  |  |  |  |  |
| 5:39163919-39164053 | Reverse | KC632578 | 179 | 313 | Forward | 135 | 251 | 7E-067 | 96.3 | - |  |  |  |  |  |
| 3:83777365-83777497 | Reverse | KC632578 | 168 | 299 | Forward | 133 | 239 | 3.5E-063 | 96.24 | - |  |  |  |  |  |
| AADN03009338.1:1-129 | Forward | KC632578 | 590 | 718 | Forward | 129 | 244 | 1.3E-064 | 96.9 | - |  |  |  |  |  |
| 1:160460651-160460765 | Reverse | KC632578 | 168 | 282 | Forward | 115 | 226 | 2.2E-059 | 100 | - |  |  |  |  |  |
| 6:9815748-9815862 | Forward | KC632578 | 4001 | 4115 | Forward | 115 | 214 | 8.8E-056 | 97.39 | Y |  |  |  |  |  |
| JH376310.1:7138-7252 | Reverse | KC632578 | 4001 | 4115 | Forward | 115 | 219 | 4.2E-057 | 98.26 | - |  |  |  |  |  |
| 6:9811710-9811822 | Forward | KC632578 | 4003 | 4115 | Forward | 113 | 210 | 1.5E-054 | 97.35 | Y |  |  |  |  |  |
| 8:23381905-23382014 | Reverse | KC632578 | 3819 | 3928 | Forward | 110 | 175 | 6.3E-044 | 89.09 | - |  |  |  |  |  |
| Z:61305340-61305436 | Forward | KC632578 | 4019 | 4115 | Forward | 97 | 170 | 1.8E-042 | 94.85 | Y |  |  |  |  |  |
| 4:55419927-55420018 | Reverse | KC632578 | 1 | 92 | Forward | 92 | 181 | 8.4E-046 | 100 | - |  |  |  |  |  |
| 4:55419927-55420018 | Reverse | KC632578 | 3926 | 4017 | Forward | 92 | 181 | 8.4E-046 | 100 | - |  |  |  |  |  |
| Z:18808450-18808538 | Forward | KC632578 | 4028 | 4115 | Forward | 89 | 135 | 5.4E-032 | 88.76 | - |  |  |  |  |  |
| 5:2705651-2705738 | Reverse | KC632578 | 4028 | 4115 | Forward | 88 | 166 | 3.7E-041 | 97.73 | Y |  |  |  |  |  |
| JH375237.1:50165-50251 | Reverse | KC632578 | 216 | 302 | Forward | 87 | 167 | 1.5E-041 | 97.7 | - |  |  |  |  |  |
| 5:31304466-31304550 | Reverse | KC632578 | 4019 | 4103 | Forward | 85 | 156 | 4.2E-038 | 96.47 | Y |  |  |  |  |  |
| JH375237.1:50306-50389 | Reverse | KC632578 | 94 | 177 | Forward | 84 | 125 | 8.1E-029 | 84.52 | - |  |  |  |  |  |
| JH376310.1:2450-2531 | Reverse | KC632578 | 3082 | 3163 | Forward | 82 | 130 | 1.8E-030 | 89.02 | - |  |  |  |  |  |
| AADN03018735.1:723-803 | Reverse | KC632578 | 4035 | 4115 | Forward | 81 | 155 | 8.1E-038 | 98.77 | - |  |  |  |  |  |
| 2:55802594-55802673 | Reverse | KC632578 | 3084 | 3163 | Forward | 80 | 118 | 8.4E-027 | 85 | - |  |  |  |  |  |
| 5:31304463-31304541 | Reverse | KC632578 | 103 | 181 | Forward | 79 | 146 | 2.9E-035 | 97.47 | Y |  |  |  |  |  |
| 5:39164052-39164130 | Reverse | KC632578 | 103 | 181 | Forward | 79 | 144 | 1.7E-034 | 96.2 | - |  |  |  |  |  |
| 5:39164055-39164130 | Reverse | KC632578 | 4028 | 4103 | Forward | 76 | 138 | 8E-033 | 96.05 | - |  |  |  |  |  |
| 6:13725441-13725516 | Reverse | KC632578 | 4028 | 4103 | Forward | 76 | 138 | 8E-033 | 96.05 | Y |  |  |  |  |  |
| JH375231.1:5300-5375 | Reverse | KC632578 | 4028 | 4103 | Forward | 76 | 130 | 1.9E-030 | 92.11 | - |  |  |  |  |  |
| JH375237.1:50306-50380 | Reverse | KC632578 | 4028 | 4102 | Forward | 75 | 114 | 1.5E-025 | 85.33 | - |  |  |  |  |  |
| 1:152942714-152942787 | Reverse | KC632578 | 3090 | 3163 | Forward | 74 | 117 | 1.7E-026 | 89.19 | - |  |  |  |  |  |
| 4:23249554-23249627 | Reverse | KC632578 | 3090 | 3163 | Forward | 74 | 109 | 6.5E-024 | 86.49 | - |  |  |  |  |  |
| JH375231.1:8433-8504 | Reverse | KC632578 | 110 | 181 | Forward | 72 | 122 | 4.8E-028 | 91.67 | - |  |  |  |  |  |
| AADN03010133.1:1-69 | Forward | KC632578 | 245 | 313 | Forward | 69 | 133 | 2.3E-031 | 98.55 | - |  |  |  |  |  |
| JH375964.1:3184-3248 | Reverse | KC632578 | 3099 | 3163 | Forward | 65 | 91 | 9.9E-019 | 83.08 | - |  |  |  |  |  |
| 8:23384631-23384683 | Reverse | KC632578 | 746 | 805 | Forward | 60 | 78 | 9.5E-015 | 80 | - |  |  |  |  |  |
| JH375231.1:8513-8569 | Reverse | KC632578 | 30 | 85 | Forward | 57 | 84 | 1.3E-016 | 87.72 | - |  |  |  |  |  |
| 1:152942809-152942862 | Reverse | KC632578 | 3018 | 3071 | Forward | 54 | 88 | 1E-017 | 88.89 | - |  |  |  |  |  |
| AADN03025131.1:4160-4213 | Forward | KC632578 | 3018 | 3071 | Forward | 54 | 90 | 2.1E-018 | 90.74 | - |  |  |  |  |  |
| 4:22223705-22223757 | Forward | KC632578 | 3015 | 3067 | Forward | 53 | 86 | 4.9E-017 | 88.68 | - |  |  |  |  |  |
| JH375964.1:3280-3332 | Reverse | KC632578 | 3018 | 3070 | Forward | 53 | 91 | 1.3E-018 | 92.45 | - |  |  |  |  |  |
| Z:17371024-17371076 | Reverse | KC632578 | 3018 | 3070 | Forward | 53 | 88 | 7.6E-018 | 90.57 | - |  |  |  |  |  |
| 2:81462284-81462334 | Forward | KC632578 | 263 | 313 | Forward | 51 | 95 | 7.9E-020 | 96.08 | - |  |  |  |  |  |
| 8:23385107-23385157 | Reverse | KC632578 | 179 | 229 | Forward | 51 | 86 | 3.6E-017 | 90.2 | - |  |  |  |  |  |
| 4:23249656-23249704 | Reverse | KC632578 | 3015 | 3064 | Forward | 50 | 65 | 8.1E-011 | 84 | - |  |  |  |  |  |
| AADN03011164.1:17177-17226 | Forward | KC632578 | 3114 | 3163 | Forward | 50 | 75 | 9.9E-014 | 86 | - |  |  |  |  |  |
| AADN03022602.1:437-486 | Reverse | KC632578 | 3114 | 3163 | Forward | 50 | 75 | 9.9E-014 | 86 | - |  |  |  |  |  |
| JH376310.1:2545-2594 | Reverse | KC632578 | 3022 | 3071 | Forward | 50 | 80 | 3.5E-015 | 88 | - |  |  |  |  |  |
| 2:55802690-55802738 | Reverse | KC632578 | 3022 | 3070 | Forward | 49 | 80 | 2.2E-015 | 89.8 | - |  |  |  |  |  |
| AADN03011164.1:17082-17130 | Forward | KC632578 | 3022 | 3070 | Forward | 49 | 83 | 3.5E-016 | 91.84 | - |  |  |  |  |  |
| AADN03022602.1:533-581 | Reverse | KC632578 | 3022 | 3070 | Forward | 49 | 83 | 3.5E-016 | 91.84 | - |  |  |  |  |  |
| Z:61305290-61305338 | Forward | KC632578 | 29 | 77 | Forward | 49 | 97 | 2.2E-020 | 100 | Y |  |  |  |  |  |
| Z:61305290-61305338 | Forward | KC632578 | 3954 | 4002 | Forward | 49 | 97 | 2.2E-020 | 100 | Y |  |  |  |  |  |
| 5:31304552-31304599 | Reverse | KC632578 | 3955 | 4002 | Forward | 48 | 95 | 8.1E-020 | 100 | Y |  |  |  |  |  |
| 6:9811662-9811708 | Forward | KC632578 | 31 | 77 | Forward | 47 | 88 | 7.1E-018 | 97.87 | Y |  |  |  |  |  |
| 6:9811662-9811708 | Forward | KC632578 | 3956 | 4002 | Forward | 47 | 88 | 7.1E-018 | 97.87 | Y |  |  |  |  |  |
| 6:9815702-9815748 | Forward | KC632578 | 31 | 77 | Forward | 47 | 86 | 4.5E-017 | 95.74 | Y |  |  |  |  |  |
| 5:31304554-31304599 | Reverse | KC632578 | 30 | 75 | Forward | 46 | 91 | 1.4E-018 | 100 | Y |  |  |  |  |  |
| 1:152942911-152942955 | Reverse | KC632578 | 2913 | 2957 | Forward | 45 | 65 | 1E-010 | 82.22 | - |  |  |  |  |  |
| 6:13725529-13725569 | Reverse | KC632578 | 35 | 75 | Forward | 41 | 76 | 4.8E-014 | 95.12 | Y |  |  |  |  |  |
| JH375231.1:5390-5428 | Reverse | KC632578 | 35 | 73 | Forward | 39 | 69 | 3.6E-012 | 92.31 | - |  |  |  |  |  |
| JH375231.1:5390-5428 | Reverse | KC632578 | 3960 | 3998 | Forward | 39 | 69 | 3.6E-012 | 92.31 | - |  |  |  |  |  |
| JH376310.1:7252-7290 | Reverse | KC632578 | 3964 | 4002 | Forward | 39 | 75 | 9.9E-014 | 97.44 | - |  |  |  |  |  |
| AADN03025131.1:4227-4264 | Forward | KC632578 | 3082 | 3119 | Forward | 38 | 69 | 4.4E-012 | 94.74 | - |  |  |  |  |  |
| 5:2705764-2705795 | Reverse | KC632578 | 1 | 32 | Forward | 32 | 59 | 6.7E-009 | 96.88 | Y |  |  |  |  |  |
| 5:2705764-2705795 | Reverse | KC632578 | 3926 | 3957 | Forward | 32 | 59 | 6.7E-009 | 96.88 | Y |  |  |  |  |  |
| 5:31304567-31304598 | Reverse | KC632578 | 3926 | 3957 | Forward | 32 | 59 | 6.7E-009 | 96.88 | Y |  |  |  |  |  |
| 5:31304567-31304598 | Reverse | KC632578 | 1 | 32 | Forward | 32 | 59 | 6.7E-009 | 96.88 | Y |  |  |  |  |  |
| 5:39164156-39164187 | Reverse | KC632578 | 1 | 32 | Forward | 32 | 59 | 6.7E-009 | 96.88 | - |  |  |  |  |  |
| 5:39164156-39164187 | Reverse | KC632578 | 3926 | 3957 | Forward | 32 | 59 | 6.7E-009 | 96.88 | - |  |  |  |  |  |
| 1:140939292-140939317 | Forward | KC632578 | 308 | 333 | Forward | 26 | 51 | 0.0000011 | 100 | - |  |  |  |  |  |
| 1:163292258-163292283 | Forward | KC632578 | 308 | 333 | Forward | 26 | 51 | 0.0000011 | 100 | - |  |  |  |  |  |
| 2:132715054-132715079 | Forward | KC632578 | 308 | 333 | Forward | 26 | 51 | 0.0000011 | 100 | - |  |  |  |  |  |
| 3:80820598-80820623 | Forward | KC632578 | 308 | 333 | Forward | 26 | 51 | 0.0000011 | 100 | - |  |  |  |  |  |
| Z:18808422-18808447 | Forward | KC632578 | 59 | 83 | Forward | 26 | 38 | 0.01 | 92.31 | - |  |  |  |  |  |
| Z:18808422-18808447 | Forward | KC632578 | 3984 | 4008 | Forward | 26 | 38 | 0.01 | 92.31 | - |  |  |  |  |  |
| 5:2705739-2705763 | Reverse | KC632578 | 63 | 86 | Forward | 25 | 39 | 0.0066 | 96 | Y |  |  |  |  |  |
| 5:2705739-2705763 | Reverse | KC632578 | 3988 | 4011 | Forward | 25 | 39 | 0.0066 | 96 | Y |  |  |  |  |  |
| 2:20044080-20044102 | Forward | KC632578 | 4093 | 4115 | Forward | 23 | 45 | 0.00012 | 100 | - |  |  |  |  |  |
| 8:8899914-8899936 | Forward | KC632578 | 4093 | 4115 | Forward | 23 | 45 | 0.00012 | 100 | - |  |  |  |  |  |
| AADN03025776.1:18-37 | Reverse | KC632578 | 699 | 718 | Forward | 20 | 37 | 0.019 | 95 | - |  |  |  |  |  |
| 2:52627010-52627027 | Forward | KC632578 | 1789 | 1806 | Forward | 18 | 35 | 0.11 | 100 | - |  |  |  |  |  |
| 5:31304535-31304552 | Reverse | KC632578 | 76 | 93 | Forward | 18 | 35 | 0.065 | 100 | Y |  |  |  |  |  |
| 6:13725529-13725545 | Reverse | KC632578 | 3984 | 4000 | Forward | 17 | 34 | 0.24 | 100 | Y |  |  |  |  |  |
| JH375964.1:2936-2951 | Reverse | KC632578 | 3412 | 3427 | Forward | 16 | 32 | 0.98 | 100 | - |  |  |  |  |  |
|  |  |  |  |  |  |  |  |  |  |  |  |  |  |  |  |
| [1] Indicates whether or not the BLAT alignment overlaps with an interval in S8a | | | | | | | | | | |  |  |  |  |  |

  


---

  


# Supplementary Table 9: *S9*

| S9. Intervals at high frequency (≥0.8) in Ethiopian chickens from one region (Horro / Jarso), and low frequency (≤0.2) in the chickens from the other region (Horro / Jarso) | | | | | | | |
|  |  |  |  |  |  |  |  |
| **Interval** | **Sample** | **Observed EAV-HP site** | **Breakpoint [1]** | ***Galgal4*** | **U3 [2]** | **R [2]** | **U5 [2]** |
| chr1:163,694,748-163,695,372 | JA1A17A | chr1:163,694,756-163,695,363 | chr1:163,694,968 | No | 0.05 | 1 | 1 |
|  | JB1A25B | chr1:163,694,748-163,695,372 | chr1:163,694,968 |  | 0.05 | 1 | 1 |
|  | JB1B16A | chr1:163,694,750-163,695,341 | - |  | 0.05 | 1 | 1 |
|  | JB2A04B.1 | chr1:163,694,782-163,695,352 | chr1:163,694,968 |  | 0.05 | 1 | 1 |
|  | JB2A04B.2 | chr1:163,694,818-163,695,334 | - |  | 0.05 | 1 | 1 |
|  | Line15 | 1:163695172-163695275 | - |  | 0 | 0 | 0.83 |
|  | Line7 | 1:163694811-163695236 | - |  | 0.05 | 1 | 1 |
| chr2:51,801,055-51,801,954 | JA1A17A | chr2:51,801,067-51,801,954 | chr2:51,801,494 | No | 0.99 | 1 | 0.99 |
|  | JA2A10B | chr2:51,801,070-51,801,931 | chr2:51,801,494 |  | 0.99 | 1 | 0.99 |
|  | JB1A25B | chr2:51,801,071-51,801,937 | chr2:51,801,494 |  | 0.99 | 1 | 0.99 |
|  | JB2A04B.1 | chr2:51,801,055-51,801,942 | chr2:51,801,494 |  | 0.99 | 1 | 0.99 |
|  | JB2A04B.2 | chr2:51,801,109-51,801,932 | chr2:51,801,494 |  | 0.99 | 1 | 0.99 |
|  | Line 15 | chr2:51,801,179-51,801,817 | - |  | 0.83 | 1 | 0.99 |
|  | Line 6 | chr2:51,801,080-51,801,932 | - |  | 0.99 | 1 | 0.99 |
|  | Line 7 | chr2:51,801,063-51,801,875 | - |  | 0.99 | 1 | 1 |
|  | Line N | chr2:51,801,166-51,801,833 | - |  | 0.95 | 1 | 1 |
|  | Line P | chr2:51,801,136-51,801,839 | - |  | 0.98 | 1 | 0.98 |
|  | Line Zero | chr2:51,801,081-51,801,932 | chr2:51,801,494 |  | 1 | 1 | 0.99 |
|  | Wellcome | chr2:51,801,351-51,801,685 | chr2:51,801,494 |  | 0.68 | 1 | 0.99 |
|  | Taiwanese | chr2:51,801,229-51,801,824 | chr2:51,801,494 |  | 0.99 | 1 | 0.99 |
| chr2:129,140,825-129,141,707 | HA1B25B | chr2:129,140,825-129,141,694 | chr2:129,141,273 | chr2:129,141,279 | No | 0.99 | 1 | 1 |
|  | HA2A10B | chr2:129,140,834-129,141,707 | chr2:129,141,273 | chr2:129,141,279 |  | 0.99 | 1 | 1 |
|  | HA2A25B | chr2:129,140,856-129,141,690 | chr2:129,141,273 | chr2:129,141,279 |  | 0.99 | 1 | 1 |
|  | HB1A16A | chr2:129,140,835-129,141,696 | chr2:129,141,273 | chr2:129,141,279 |  | 0.99 | 1 | 1 |
|  | HB1B21B | chr2:129,140,842-129,141,689 | chr2:129,141,273 | chr2:129,141,279 |  | 0.99 | 1 | 1 |
| chr3:84,550,518-84,551,452 | JA1A17A | chr3:84,550,564-84,551,413 | chr3:84,550,973 | chr3:84,550,979 | No | 1 | 1 | 1 |
|  | JA2A10B | chr3:84,550,520-84,551,425 | chr3:84,550,973 | chr3:84,550,979 |  | 1 | 1 | 1 |
|  | JB1A25B | chr3:84,550,525-84,551,408 | chr3:84,550,973 | chr3:84,550,979 |  | 1 | 1 | 1 |
|  | JB1B16A | chr3:84,550,518-84,551,452 | chr3:84,550,973 | chr3:84,550,979 |  | 1 | 1 | 1 |
|  | JB2A04B.1 | chr3:84,550,557-84,551,402 | chr3:84,550,973 | chr3:84,550,979 |  | 1 | 1 | 1 |
|  | JB2A04B.2 | chr3:84,550,542-84,551,403 | chr3:84,550,973 | chr3:84,550,979 |  | 1 | 1 | 1 |
| chr3:102,777,226-102,778,143 | HA1A22A | chr3:102,777,273-102,778,125 | chr3:102,777,685 | No | 1 | 1 | 1 |
|  | HA2A10B | chr3:102,777,238-102,778,134 | chr3:102,777,685 |  | 1 | 1 | 1 |
|  | HA2A25B | chr3:102,777,298-102,778,102 | chr3:102,777,685 |  | 1 | 1 | 1 |
|  | HB1A16A | chr3:102,777,236-102,778,143 | chr3:102,777,685 |  | 1 | 1 | 1 |
|  | HB1B21B | chr3:102,777,268-102,778,110 | chr3:102,777,685 |  | 1 | 1 | 1 |
|  | JB1A25B | chr3:102,777,226-102,778,117 | chr3:102,777,685 |  | 1 | 1 | 1 |
| chr8:11,644,653-11,645,314 | JA2A10B | chr8:11,644,655-11,645,224 | chr8:11,645,101 | No | 0.05 | 1 | 1 |
|  | JB1B16A | chr8:11,644,724-11,645,314 | chr8:11,645,101 |  | 0.05 | 1 | 0.99 |
|  | JB1A25B | chr8:11,644,671-11,645,291 | chr8:11,645,101 |  | 0.05 | 1 | 0.99 |
|  | JB2A04B.1 | chr8:11,644,653-11,645,312 | chr8:11,645,101 |  | 0.05 | 1 | 0.99 |
|  | JB2A04B.2 | chr8:11,644,677-11,645,263 | chr8:11,645,101 |  | 0.05 | 1 | 0.99 |
| chrZ:50,872,653-50,873,337 | HA1A22A | chrZ:50872687-50873298 | chrZ:50,872,885 | No | 0.05 | 1 | 1 |
|  | HA1B25B | chrZ:50872654-50873335 | - |  | 0.05 | 1 | 1 |
|  | HA2A10B | chrZ:50872653-50873303 | - |  | 0.05 | 1 | 1 |
|  | HA2A25B | chrZ:50872673-50873303 | chrZ:50,872,885 |  | 0.05 | 1 | 1 |
|  | HB1A16A | chrZ:50872717-50873337 | chrZ:50,872,885 |  | 0.05 | 1 | 1 |
|  | HB1B21B | chrZ:50872673-50873314 | chrZ:50,872,885 |  | 0.05 | 1 | 1 |
|  | JA1A17A | chrZ:50872704-50873279 | chrZ:50,872,885 |  | 0.05 | 1 | 1 |
|  | Wellcome | chrZ:50872968-50873178 | - |  | 0.04 | 1 | 0.98 |
|  |  |  |  |  |  |  |  |
| Excludes intervals on unplaced contigs | | | | | | | |
| [1] Only samples with long soft-clipped sequences (≥ 20 bp) were used for the resolution of breakpoints | | | | | | | |
| [2] Fraction of sequence coverage of the LTR U3, R and U5 regions respectively | | | | | | | |

  


---

  


# Supplementary Table 10: *S10*

| S10. Integration site breakpoints identified near to genes in functional annotation enriched clusters | | | | | | | | | | | |
|  |  |  |  |  |  |  |  |  |  |  |  |
| **Ensembl Gene ID** | **Gene** | **Chr** | **Gene Start** | **Gene End** | **Sample** | **Observed EAV-HP site** | **Breakpoint [1]** | ***Galgal4 [2]*** | **U3 [3]** | **R [3]** | **U5 [3]** |
| ENSGALG00000009523 | *CNTN1* | 1 | 28,777,011 | 28,942,648 | HB1B21B | chr1:28,836,094-28,836,942 | chr1:28,836,524 | No | 1 | 1 | 1 |
| ENSGALG00000012559 | *LARGE* | 1 | 52,524,419 | 52,799,322 | Silkie | chr1:52,743,958-52,744,500 | chr1:52,744,294 | No | 0 | 0 | 0.62 |
|  |  |  |  |  | Line P | chr1:52,743,902-52,744,440 | - |  | 0.05 | 1 | 0.98 |
| ENSGALG00000015511 | *ROBO1* | 1 | 95,642,548 | 95,948,471 | JB2A04B.1 | chr1:95,854,597-95,855,207 | chr1:95,854,790 | No | 0.05 | 1 | 1 |
|  |  |  |  |  | JB2A04B.2 | chr1:95,854,651-95,855,224 | chr1:95,854,790 |  | 0.05 | 1 | 0.99 |
| ENSGALG00000017197 | *CNTN5* | 1 | 182,715,565 | 183,138,508 | HA1A22A | Chr1:182,832,868-182,834,031 | - | chr1:182,833,311-182,833,598 | 0.97 | 1 | 0.79 |
|  |  |  |  |  | HA1B25B | chr1:182,832,894-182,834,053 | - |  | 0.88 | 1 | 0.98 |
|  |  |  |  |  | HA2A10B | chr1:182,832,922-182,834,040 | - |  | 0.88 | 1 | 0.96 |
|  |  |  |  |  | HA2A25B | chr1:182,832,896-182,834,057 | - |  | 0.95 | 1 | 0.77 |
|  |  |  |  |  | HB1A16A | chr1:182,832,886-182,834,039 | - |  | 0.99 | 1 | 0.81 |
|  |  |  |  |  | HB1B21B | chr1:182,832,947-182,834,029 | - |  | 0.94 | 1 | 0.98 |
|  |  |  |  |  | JA1A17A | chr1:182,832,884-182,834,049 | - |  | 0.99 | 1 | 0.98 |
|  |  |  |  |  | JA2A10B | chr1:182,832,889-182,834,039 | - |  | 0.99 | 1 | 0.82 |
|  |  |  |  |  | JB1A25B | chr1:182,832,884-182,834,061 | - |  | 0.99 | 1 | 0.82 |
|  |  |  |  |  | JB1B16A | chr1:182,832,885-182,834,052 | - |  | 1 | 1 | 0.81 |
|  |  |  |  |  | JB2A04B.1 | chr1:182,832,907-182,834,057 | - |  | 0.99 | 1 | 0.81 |
|  |  |  |  |  | JB2A04B.2 | chr1:182,832,887-182,834,032 | - |  | 0.99 | 1 | 0.98 |
|  |  |  |  |  | Line 15 | chr1:182,832,958-182,833,933 | - |  | 0.85 | 1 | 0.88 |
|  |  |  |  |  | Line 6 | chr1:182,832,918-182,834,042 | chr1:182,833,313 |  | 0.97 | 1 | 0.98 |
|  |  |  |  |  | Line 7 | chr1:182,832,913-182,834,008 | - |  | 0.99 | 1 | 0.98 |
|  |  |  |  |  | Line C | chr1:182,832,847-182,833,971 | chr1:182,833,598 |  | 0.99 | 1 | 0.93 |
|  |  |  |  |  | Line P | chr1:182,832,958-182,834,011 | chr1:182,833,313 |  | 0.95 | 1 | 0.98 |
|  |  |  |  |  | LineZero | 1:182833163-182833264 | - |  | 0.68 | 1 | 0.37 |
|  |  |  |  |  | RJF | chr1:182,832,996-182,833,874 | - |  | 0.99 | 1 | 0.98 |
|  |  |  |  |  | Silkie | chr1:182,832,905-182,834,082 | - |  | 1 | 1 | 0.99 |
|  |  |  |  |  | Taiwanese | chr1:182,832,952-182,833,960 | chr1:182,833,599 |  | 1 | 1 | 0.98 |
| ENSGALG00000013782 | *CDH7* | 2 | 95,477,061 | 95,554,752 | HA1A22A | chr2:95,480,963-95,481,818 | chr2:95,481,397 | No | 1 | 1 | 0.99 |
|  |  |  |  |  | HA2A10B | chr2:95,480,964-95,481,785 | chr2:95,481,397 |  | 1 | 1 | 0.99 |
|  |  |  |  |  | HA2A25B | chr2:95,481,033-95,481,781 | chr2:95,481,397 |  | 1 | 1 | 0.99 |
|  |  |  |  |  | HB1B21B | chr2:95,480,991-95,481,786 | - |  | 1 | 1 | 1 |
|  |  |  |  |  | JA1A17A | chr2:95,480,957-95,481,781 | chr2:95,481,397 |  | 1 | 1 | 0.99 |
|  |  |  |  |  | JB1B16A | chr2:95,480,969-95,481,814 | chr2:95,481,397 |  | 1 | 1 | 0.99 |
|  |  |  |  |  | JB2A04B.1 | chr2:95,480,982-95,481,804 | chr2:95,481,397 |  | 1 | 1 | 1 |
|  |  |  |  |  | JB2A04B.2 | chr2:95,480,992-95,481,797 | chr2:95,481,397 |  | 1 | 1 | 1 |
|  |  |  |  |  | Line6 | chr2:95,480,970-95,481,836 | - |  | 1 | 1 | 0.99 |
|  |  |  |  |  | LineC | chr2:95,481,141-95,481,827 | chr2:95,481,397 |  | 1 | 1 | 0.99 |
|  |  |  |  |  | LineN | chr2:95,481,247-95,481,522 | - |  | 0.88 | 1 | 0.16 |
|  |  |  |  |  | Wellcome | chr2:95,481,020-95,481,760 | - |  | 1 | 1 | 0.98 |
|  |  |  |  |  | Taiwanese | chr2:95,481,137-95,481,700 | chr2:95,481,397 |  | 1 | 1 | 0.99 |
| ENSGALG00000009239 | *TLR2-2* | 4 | 19,209,334 | 19,210,887 | Silkie | chr4:19,211,542-19,212,312 | chr4:19,211,934 | No | 0.38 | 0 | 0.51 |
| ENSGALG00000010692 | *LRP8* | 8 | 23,561,280 | 23,710,577 | JA2A10B | chr8:23,631,239-23,632,111 | chr8:23,631,660 |  | 1 | 1 | 0.98 |
| ENSGALG00000014661 | *FAM172A* | Z | 57,916,145 | 58,180,255 | Silkie | chrZ:57,973,076-57,973,674 | chrZ:57,973,439 | No | 0 | 0 | 0.63 |
|  |  |  |  |  |  |  |  |  |  |  |  |
| [1] Only samples with long soft-clipped sequences (≥ 20 bp) were used for the resolution of breakpoints | | | | | | | | |  |  |  |
| [2] EAV-HP alignment to Galgal4 confirmed by BLAT | | | | | | | | |  |  |  |
| [3] Fraction of sequence coverage of the LTR U3, R and U5 regions respectively | | | | | | | | |  |  |  |

  


---

  


# Supplementary Table 11a: *S11a*

| S11a. DAVID Functional Annotation Clustering[1] of genes within 5 kb of intervals | | | | | | | | | | | | |
|  |  |  |  |  |  |  |  |  |  |  |  |  |
| **Annotation Cluster 1** | Enrichment Score: 1.98 |  |  |  |  |  |  |  |  |  |  |  |
| **Category** | **Term** | **Count** | **%** | **PValue** | **Genes** | **List Total** | **Pop Hits** | **Pop Total** | **Fold Enrichment** | **Bonferroni** | **Benjamini** | **FDR** |
| UP\_SEQ\_FEATURE | glycosylation site:N-linked (GlcNAc...) | 7 | 9.21 | 0.001 | ENSGALG00000013782, ENSGALG00000014661, ENSGALG00000012559, ENSGALG00000009523, ENSGALG00000009239,  ENSGALG00000010692, ENSGALG00000017197 | 9 | 410 | 2121 | 4.02 | 0.06 | 0.06 | 1.01 |
| SP\_PIR\_KEYWORDS | glycoprotein | 7 | 9.21 | 0.011 | ENSGALG00000013782, ENSGALG00000014661, ENSGALG00000012559, ENSGALG00000009523, ENSGALG00000009239,  ENSGALG00000010692, ENSGALG00000017197 | 17 | 430 | 3361 | 3.22 | 0.44 | 0.17 | 10.16 |
| UP\_SEQ\_FEATURE | signal peptide | 6 | 7.89 | 0.017 | ENSGALG00000013782, ENSGALG00000014661, ENSGALG00000009523, ENSGALG00000009239, ENSGALG00000010692,  ENSGALG00000017197 | 9 | 476 | 2121 | 2.97 | 0.67 | 0.43 | 16.05 |
| SP\_PIR\_KEYWORDS | signal | 6 | 7.89 | 0.065 | ENSGALG00000013782, ENSGALG00000014661, ENSGALG00000009523, ENSGALG00000009239, ENSGALG00000010692,  ENSGALG00000017197 | 17 | 478 | 3361 | 2.48 | 0.97 | 0.39 | 47.93 |
|  |  |  |  |  |  |  |  |  |  |  |  |  |
| **Annotation Cluster 2** | Enrichment Score: 1.57 |  |  |  |  |  |  |  |  |  |  |  |
| **Category** | **Term** | **Count** | **%** | **PValue** | **Genes** | **List Total** | **Pop Hits** | **Pop Total** | **Fold Enrichment** | **Bonferroni** | **Benjamini** | **FDR** |
| INTERPRO | IPR013098:Immunoglobulin I-set | 3 | 3.95 | 0.010 | ENSGALG00000015511, ENSGALG00000009523, ENSGALG00000017197 | 17 | 45 | 4557 | 17.87 | 0.48 | 0.48 | 10.09 |
| INTERPRO | IPR003598:Immunoglobulin subtype 2 | 3 | 3.95 | 0.014 | ENSGALG00000015511, ENSGALG00000009523, ENSGALG00000017197 | 17 | 53 | 4557 | 15.17 | 0.59 | 0.36 | 13.57 |
| SMART | SM00408:IGc2 | 3 | 3.95 | 0.014 | ENSGALG00000015511, ENSGALG00000009523, ENSGALG00000017197 | 10 | 53 | 2507 | 14.19 | 0.18 | 0.18 | 9.36 |
| INTERPRO | IPR007110:Immunoglobulin-like | 3 | 3.95 | 0.073 | ENSGALG00000015511, ENSGALG00000009523, ENSGALG00000017197 | 17 | 128 | 4557 | 6.28 | 0.99 | 0.79 | 53.30 |
| INTERPRO | IPR013783:Immunoglobulin-like fold | 3 | 3.95 | 0.094 | ENSGALG00000015511, ENSGALG00000009523, ENSGALG00000017197 | 17 | 149 | 4557 | 5.40 | 1.00 | 0.79 | 63.28 |
|  |  |  |  |  |  |  |  |  |  |  |  |  |
| [1] Enrichment score > 1.3, stringency HIGH | | | | | | | | | | | | |
|  |  |  |  |  |  |  |  |  |  |  |  |  |
| Although the FDR values reported here are insignificant, likewise annotation clustering performed with g:Profiler returned significant FDR values for the same gene list (S10b). | | | | | | | | | | | | |

  


---

  


# Supplementary Table 11b: *S11b*

| S11b. gProfiler g:GOSt analysis of genes within 5 kb of intervals | | | | | | | | | | | |
|  | | | | | | | | | | | | | | | | | | | |
| **P-value [1]** | **T** | **Q** | **Q&T** | **Q&T/Q** | **Q&T/T** | **term ID** | **t type** | **t group** | **t name** | **t depth in group** | **Q&T list** |
| 0.028 | 2 | 83 | 1 | 0.012 | 0.5 | GO:0010815 | BP | 33 | bradykinin catabolic process | 1 | ENSGALG00000008523 |
| 0.0459 | 37 | 83 | 2 | 0.024 | 0.054 | GO:0050807 | BP | 32 | regulation of synapse organization | 1 | ENSGALG00000016594,ENSGALG00000010518 |
| 0.0419 | 3 | 83 | 1 | 0.012 | 0.333 | GO:0032493 | BP | 7 | response to bacterial lipoprotein | 1 | ENSGALG00000009239 |
| 0.014 | 1 | 83 | 1 | 0.012 | 1 | GO:0042494 | BP | 7 | detection of bacterial lipoprotein | 1 | ENSGALG00000009239 |
| 0.0436 | 36 | 83 | 2 | 0.024 | 0.056 | GO:0021885 | BP | 18 | forebrain cell migration | 1 | ENSGALG00000015511,ENSGALG00000010692 |
| 0.0413 | 35 | 83 | 2 | 0.024 | 0.057 | GO:0022029 | BP | 18 | telencephalon cell migration | 2 | ENSGALG00000015511,ENSGALG00000010692 |
| 0.0269 | 28 | 83 | 2 | 0.024 | 0.071 | GO:0021795 | BP | 18 | cerebral cortex cell migration | 3 | ENSGALG00000015511,ENSGALG00000010692 |
| 0.0168 | 22 | 83 | 2 | 0.024 | 0.091 | GO:0051865 | BP | 16 | protein autoubiquitination | 1 | ENSGALG00000011562,ENSGALG00000014732 |
| 0.028 | 2 | 83 | 1 | 0.012 | 0.5 | GO:0050925 | BP | 55 | negative regulation of negative chemotaxis | 1 | ENSGALG00000015511 |
| 0.0419 | 3 | 83 | 1 | 0.012 | 0.333 | GO:0051931 | BP | 27 | regulation of sensory perception | 1 | ENSGALG00000012297 |
| 0.0419 | 3 | 83 | 1 | 0.012 | 0.333 | GO:0051930 | BP | 27 | regulation of sensory perception of pain | 2 | ENSGALG00000012297 |
| 0.0419 | 3 | 83 | 1 | 0.012 | 0.333 | GO:0070099 | BP | 40 | regulation of chemokine-mediated signaling pathway | 1 | ENSGALG00000015511 |
| 0.0419 | 3 | 83 | 1 | 0.012 | 0.333 | GO:0070100 | BP | 40 | negative regulation of chemokine-mediated signaling pathway | 1 | ENSGALG00000015511 |
| 0.0419 | 3 | 83 | 1 | 0.012 | 0.333 | GO:0070842 | BP | 14 | aggresome assembly | 1 | ENSGALG00000011562 |
| 0.0419 | 3 | 83 | 1 | 0.012 | 0.333 | GO:0002051 | BP | 24 | osteoblast fate commitment | 1 | ENSGALG00000009977 |
| 0.028 | 2 | 83 | 1 | 0.012 | 0.5 | GO:0021825 | BP | 31 | substrate-dependent cerebral cortex tangential migration | 1 | ENSGALG00000015511 |
| 0.028 | 2 | 83 | 1 | 0.012 | 0.5 | GO:0021823 | BP | 31 | cerebral cortex tangential migration using cell-cell interactions | 2 | ENSGALG00000015511 |
| 0.028 | 2 | 83 | 1 | 0.012 | 0.5 | GO:0021827 | BP | 31 | postnatal olfactory bulb interneuron migration | 1 | ENSGALG00000015511 |
| 0.028 | 2 | 83 | 1 | 0.012 | 0.5 | GO:0021836 | BP | 31 | chemorepulsion involved in postnatal olfactory bulb interneuron migration | 2 | ENSGALG00000015511 |
| 0.0419 | 3 | 83 | 1 | 0.012 | 0.333 | GO:0007216 | BP | 22 | G-protein coupled glutamate receptor signaling pathway | 1 | ENSGALG00000017238 |
| 0.0419 | 3 | 83 | 1 | 0.012 | 0.333 | GO:0072385 | BP | 13 | minus-end-directed organelle transport along microtubule | 1 | ENSGALG00000005249 |
| 0.0419 | 3 | 83 | 1 | 0.012 | 0.333 | GO:0051835 | BP | 29 | positive regulation of synapse structural plasticity | 1 | ENSGALG00000016594 |
| 0.00189 | 73 | 83 | 4 | 0.048 | 0.055 | GO:0007156 | BP | 39 | homophilic cell adhesion | 1 | ENSGALG00000015511,ENSGALG00000017229,ENSGALG00000013782,ENSGALG00000005310 |
| 0.028 | 2 | 83 | 1 | 0.012 | 0.5 | GO:0019860 | BP | 9 | uracil metabolic process | 1 | ENSGALG00000005509 |
| 0.028 | 2 | 83 | 1 | 0.012 | 0.5 | GO:0006212 | BP | 9 | uracil catabolic process | 1 | ENSGALG00000005509 |
| 0.0419 | 3 | 83 | 1 | 0.012 | 0.333 | GO:0006145 | BP | 12 | purine nucleobase catabolic process | 1 | ENSGALG00000005509 |
| 0.0385 | 259 | 83 | 5 | 0.06 | 0.019 | GO:0007268 | BP | 47 | synaptic transmission | 1 | ENSGALG00000016594,ENSGALG00000019054,ENSGALG00000017238,ENSGALG00000020143,ENSGALG00000010518 |
| 0.0419 | 3 | 83 | 1 | 0.012 | 0.333 | GO:0042135 | BP | 52 | neurotransmitter catabolic process | 1 | ENSGALG00000019054 |
| 0.014 | 1 | 83 | 1 | 0.012 | 1 | GO:0019859 | BP | 8 | thymine metabolic process | 1 | ENSGALG00000005509 |
| 0.014 | 1 | 83 | 1 | 0.012 | 1 | GO:0006210 | BP | 8 | thymine catabolic process | 1 | ENSGALG00000005509 |
| 0.014 | 1 | 83 | 1 | 0.012 | 1 | GO:0046125 | BP | 4 | pyrimidine deoxyribonucleoside metabolic process | 1 | ENSGALG00000005509 |
| 0.014 | 1 | 83 | 1 | 0.012 | 1 | GO:0046104 | BP | 4 | thymidine metabolic process | 2 | ENSGALG00000005509 |
| 0.028 | 2 | 83 | 1 | 0.012 | 0.5 | GO:0046135 | BP | 4 | pyrimidine nucleoside catabolic process | 1 | ENSGALG00000005509 |
| 0.0419 | 3 | 83 | 1 | 0.012 | 0.333 | GO:0046121 | BP | 4 | deoxyribonucleoside catabolic process | 1 | ENSGALG00000005509 |
| 0.014 | 1 | 83 | 1 | 0.012 | 1 | GO:0046127 | BP | 4 | pyrimidine deoxyribonucleoside catabolic process | 2 | ENSGALG00000005509 |
| 0.014 | 1 | 83 | 1 | 0.012 | 1 | GO:0006214 | BP | 4 | thymidine catabolic process | 3 | ENSGALG00000005509 |
| 0.0306 | 85 | 83 | 3 | 0.036 | 0.035 | GO:0031346 | BP | 56 | positive regulation of cell projection organization | 1 | ENSGALG00000009523,ENSGALG00000015511,ENSGALG00000007647 |
| 0.028 | 2 | 83 | 1 | 0.012 | 0.5 | GO:0035385 | BP | 43 | Roundabout signaling pathway | 1 | ENSGALG00000015511 |
| 0.0288 | 29 | 83 | 2 | 0.024 | 0.069 | GO:0005901 | CC | 2 | caveola | 1 | ENSGALG00000017044,ENSGALG00000010692 |
| 0.028 | 2 | 83 | 1 | 0.012 | 0.5 | GO:0038037 | CC | 44 | G-protein coupled receptor dimeric complex | 1 | ENSGALG00000012297 |
| 0.014 | 1 | 83 | 1 | 0.012 | 1 | GO:0038038 | CC | 44 | G-protein coupled receptor homodimeric complex | 2 | ENSGALG00000012297 |
| 0.00921 | 353 | 83 | 7 | 0.084 | 0.02 | GO:0097458 | CC | 53 | neuron part | 1 | ENSGALG00000015511,ENSGALG00000016594,ENSGALG00000017238,ENSGALG00000007647,ENSGALG00000012297, ENSGALG00000002789,ENSGALG00000021665 |
| 0.0144 | 2135 | 83 | 21 | 0.253 | 0.01 | GO:0031224 | CC | 23 | intrinsic component of membrane | 1 | ENSGALG00000009523,ENSGALG00000011614,ENSGALG00000012559,ENSGALG00000015511,ENSGALG00000017044, ENSGALG00000017197,ENSGALG00000017229,ENSGALG00000019054,ENSGALG00000017234,ENSGALG00000017238, ENSGALG00000006112,ENSGALG00000013782,ENSGALG00000012297,ENSGALG00000007466,ENSGALG00000009239, ENSGALG00000020143,ENSGALG00000010518,ENSGALG00000010692,ENSGALG00000005310,ENSGALG00000004661, ENSGALG00000003886 |
[truncated: 62,860 more chars]
