# Supplementary material for: Genome-wide analysis reveals the extent of EAV-HP integration in domestic chicken
Source: BMC Genomics. 2015 Oct 14;16:784. doi: 10.1186/s12864-015-1954-x (PMC4607243; doi:10.1186/s12864-015-1954-x)
Supplement: Supplementary file 2 — Supplementary methods. (PDF 106 kb) [file 12864_2015_1954_MOESM2_ESM.pdf]

Supplementary Methods

Genome-wide analysis reveals the extent of EAV-HP integration in domestic chicken

The pipeline for mapping EAV-HP intergration sites using paired-end next generation sequence (NGS) data was evaluated using reads downloaded from NCBI's sequence read archive (SRA). The reads used were derived from the DNA of the same bird (Accession: SAMN00216039) that was sequenced to construct the chicken reference genome (*Galgal4*). Specifically, the included the following accession numbers: SRR105787, SRR105790, SRR105791, SRR105793, SRR197983, SRR197984, SRR105788, SRR105789, SRR105792, SRR105794, SRR197985 and SRR197986; totalling 67.6 Gbases of sequence data. Once aligned to Galgal4 using BWA-MEM, the mean depth of coverage was calculated to be 61.3 X with a mean insert size of 204.

To evaluate the effects of using different depths of coverage with the pipeline, the initial mapping to Galgal4 was down-sampled by 50%. The 50% down-sampling was repeated on the resulting BAM file, and again on the BAM file generated from the additional down-sampling, resulting in 4 datasets: the original at 61.3X depth of coverage, followed by coverage at 30.65 X, 15.32 X, and 7.66X. The BAM files were converted to FASTQ files using Picard, which retains only read-pairs, and used as inputs for the EAV-HP mapping pipeline.

In addition to testing the effects of coverage, the filters within the pipeline pertaining to mapping quality (MQ) and read count (RC) were also evaluated. The MQ filter is intended to avoid the risk of generating multiple hits arising from interspersed repeats throughout the genome, for instance CR1 elements. The BWA-MEM aligner marks alignments with equally high mapping scores as having MQ=0, thus, if a read aligns equally well to multiple genomic intervals i.e. due to the presence of CR1 elements, then MQ=0. By filtering out reads with MQ=0 we reduce the risk of false positives arising from intervals located within these interspersed repetitive elements. To assess this, the pipeline was evaluated to include intervals containing reads with MQ=0 and separately to include only intervals with MQ≥20. A further filter within the pipeline, relating to the RC supporting an interval, is correlated with sequencing depth of coverage. To evaluate this filter three different settings were tested: RC=3, RC=10, and RC=$0.25\mu X_i$ in which X_i is the depth of coverage of the bird/line (i) being analysed.

To establish the sensitivity and precision of the analyses, the results were compared to EAV-HP LTR alignments to Galgal4 identified by BLAT (stand-alone version, default parameters) assuming a minimum score of 20 (Supplementary Table S7). Pipeline results were considered true positives (TP) if the interval was within 500 bp of a BLAT alignment, this distance was specified to accommodate read length and insert size. The false negative rate (FNR) was recorded as the fraction of BLAT alignments that were undetected by the mapping pipeline. Sensitivity (SENS) was recorded as the number of TP / (TP + number of false negatives). Precision (PREC) was recorded as the TP / (TP + number of false positives). False discovery rate was recorded as 1-PREC. The results of the different analyses are presented in Table 1.

Setting the MQ filter to include reads with MQ=0 results in the greatest sensitivity, however as previously mentioned there is a risk with this in that a single integration might be associated with multiple genomic intervals. Consequently, the false discovery rate (FDR) increases with increasing depths of coverage whilst the FNR

decreases. The most consistent results across all depths of coverage were observed at RC= 0.25μX_i where at MQ=20 sensitivity and precision were averaged 59% and 98%, respectively, whilst risking MQ=0 would result in an average sensitivity of 97% and precision of 95%.

Table 1. Sensitivity and precision of EAV-HP mapping pipeline subject to different MQ and RC filters

MQ	Coverage	RC	SENS	PREC	FDR	FNR
0	61.3	3	1	0.819	0.181	0
	30.65	3	1	0.888	0.112	0
	15.32	3	1	0.931	0.069	0
	7.66	3	0.874	0.976	0.024	0.126
	61.3	10	1	0.931	0.069	0
	30.65	10	0.947	0.957	0.043	0.053
	15.32	10	0.663	0.969	0.031	0.337
	7.66	10	0.126	1	0	0.874
	61.3	0.25μX_i	0.968	0.958	0.042	0.032
	30.65	0.25μX_i	0.989	0.959	0.041	0.011
	15.32	0.25μX_i	0.989	0.959	0.041	0.011
	7.66	0.25μX_i	0.926	0.936	0.064	0.074
20	61.3	3	1	0.772	0.228	0
	30.65	3	0.768	0.973	0.027	0.232
	15.32	3	0.653	0.984	0.016	0.347
	7.66	3	0.474	0.978	0.022	0.526
	61.3	10	1	0.95	0.05	0
	30.65	10	0.463	0.978	0.022	0.537
	15.32	10	0.2	0.95	0.05	0.8
	7.66	10	0.021	1	0	0.979
	61.3	0.25μX_i	0.663	0.984	0.016	0.337
	30.65	0.25μX_i	0.568	0.982	0.018	0.432
	15.32	0.25μX_i	0.579	0.982	0.018	0.421
	7.66	0.25μX_i	0.558	0.981	0.019	0.442

MQ = Mapping Quality; RC = Read Count
