# Supplementary material for: Genome-wide analysis reveals the extent of EAV-HP integration in domestic chicken
Source: BMC Genomics. 2015 Oct 14;16:784. doi: 10.1186/s12864-015-1954-x (PMC4607243; doi:10.1186/s12864-015-1954-x)

REVIGO GO Biological Process uniqueness treemap (Ggal db)

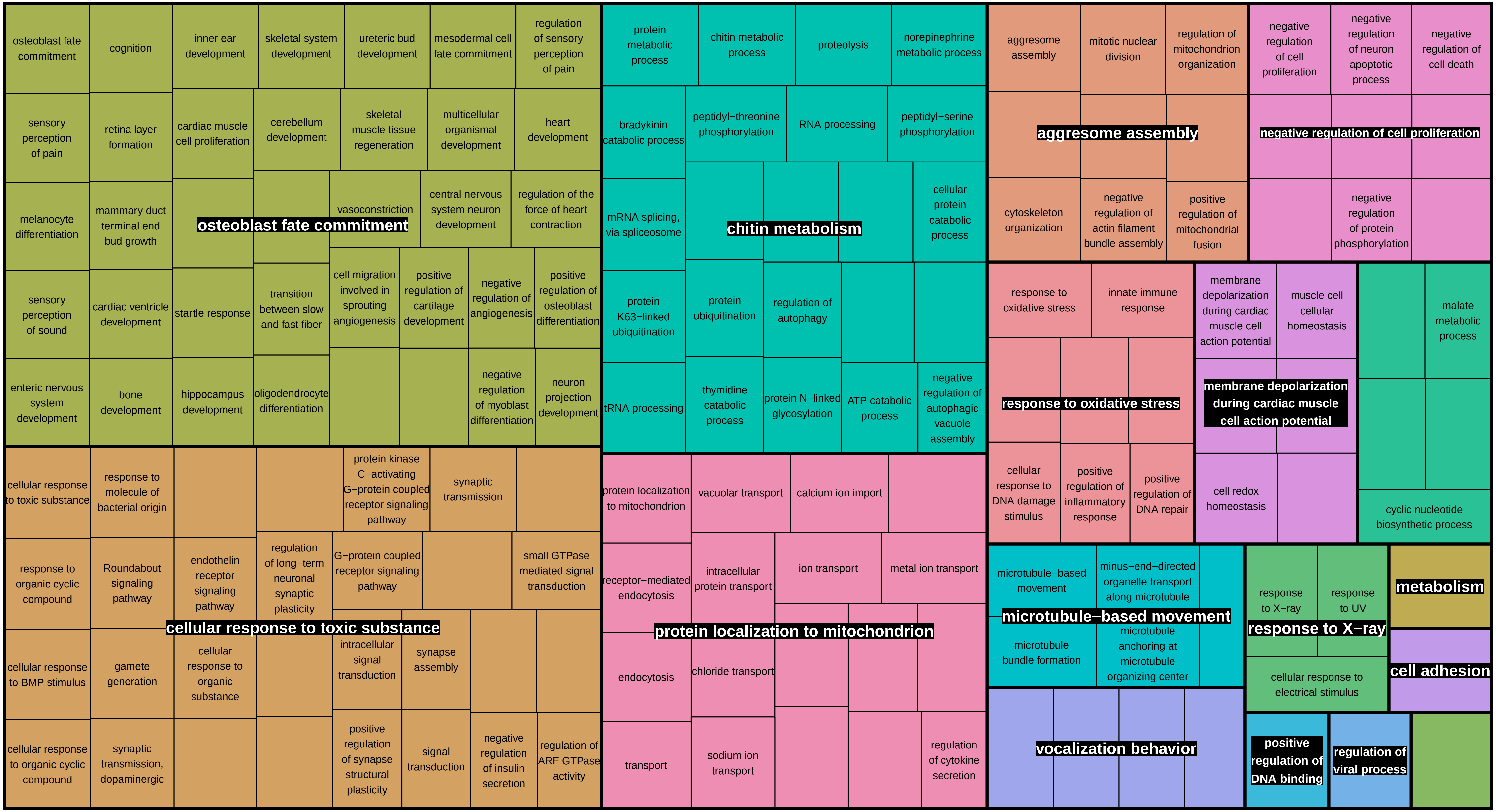

REVIGO GO Cellular Component uniqueness treemap (Ggal db)

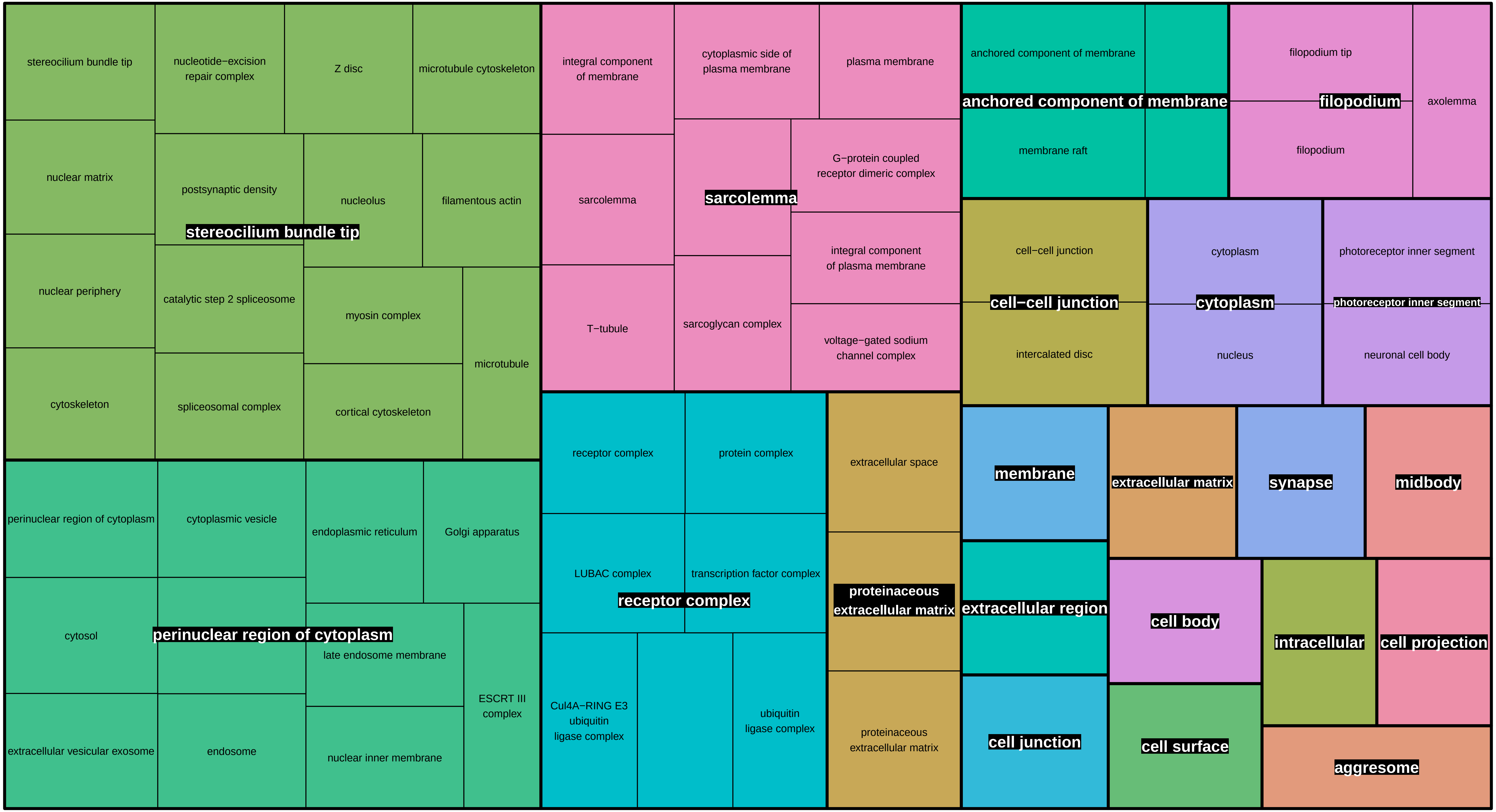

REVIGO GO Molecular Function uniqueness treemap (Ggal db)

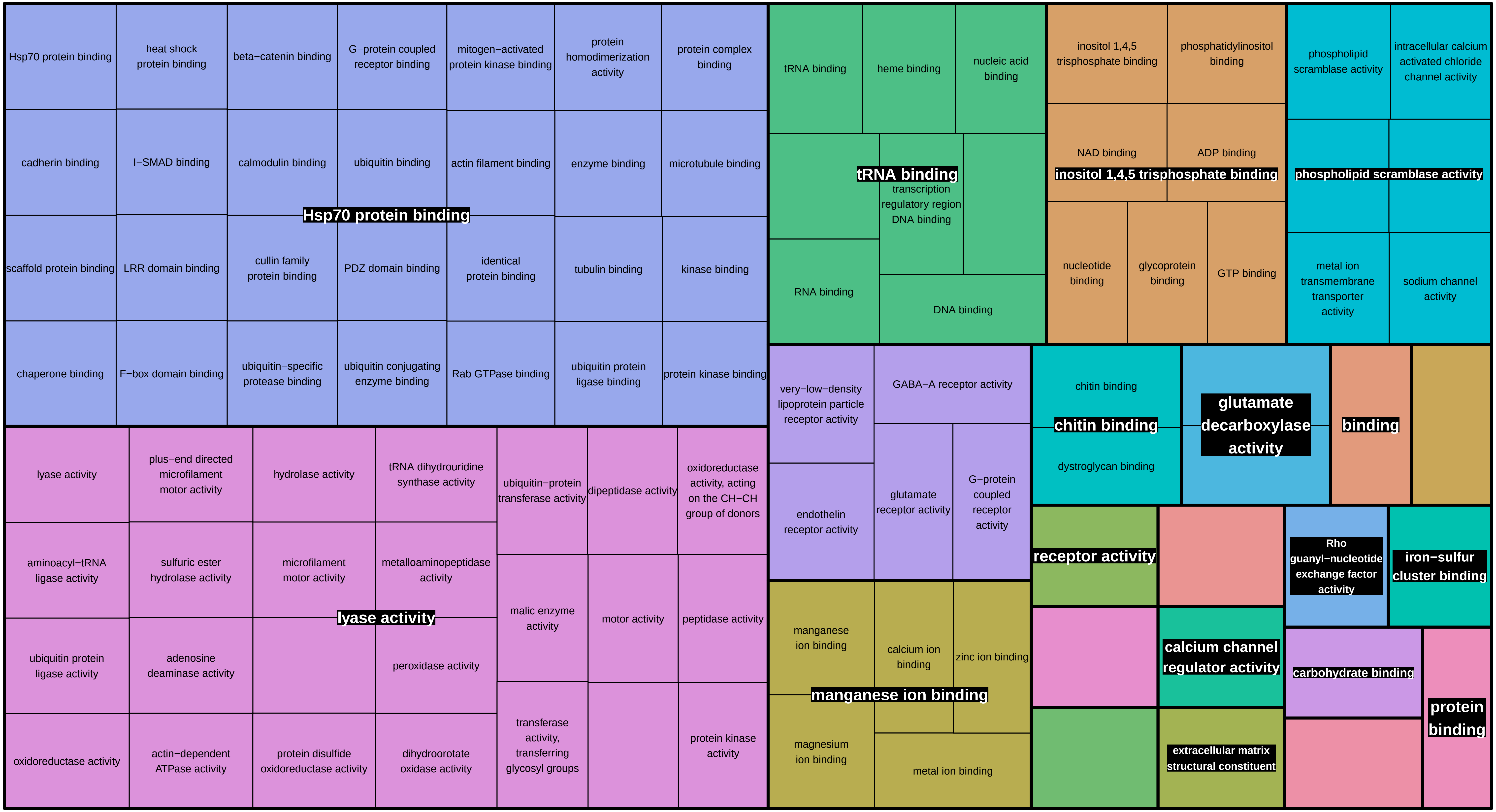

Supplement: Supplementary file 3 — Supplementary figures. SF1. REVIGO GO Biological Process uniqueness treemap. SF2. REVIGO GO Cellular Component uniqueness treemap. SF3. REVIGO GO Molecular Function uniqueness treemap. (PDF 25 kb) [file 12864_2015_1954_MOESM3_ESM.pdf]
